# Expression profile and bioinformatics analysis of circular RNAs in acute ischemic stroke in a South Chinese Han population

**DOI:** 10.1038/s41598-020-66990-y

**Published:** 2020-06-23

**Authors:** Shenghua Li, Lan Chen, Chen Xu, Xiang Qu, Zhenxiu Qin, Jinggui Gao, Jinpin Li, Jingli Liu

**Affiliations:** 1 Department of Neurology, The First Affiliated Hospital of Guangxi Medical University, Nanning, China; 2 Department of Internal Medicine, The Second Affiliated Hospital of Guangxi Medical University, Nanning, China

**Keywords:** Non-coding RNAs, Stroke

## Abstract

Recent studies have found that circular RNAs (circRNAs) play crucial roles not only in the normal growth and the development of different tissues and organs but also in the pathogenesis and progression of various disorders. However, the expression patterns and the function of circRNAs in acute ischemic stroke (AIS) in the South Chinese Han population are unclear. In the present study, RNA sequencing (RNA-seq) data was generated from 3 AIS patients and 3 healthy controls. The circRNAs were detected and identified by CIRI2 and Find_circ software. Quantitative reverse transcription-polymerase chain reaction (qRT-PCR) analyses were used to detect the expression of circRNAs. Meanwhile, the potential diagnostic value of the selected circRNAs for AIS was assessed by generating receiver operating characteristic (ROC) curve with area under curve (AUC). The bioinformatic analysis of the host genes of differentially expressed (DE) circRNAs was performed by gene ontology (GO) enrichment, Kyoto Encyclopedia of Genes and Genomes (KEGG) pathway analysis, KOBAS for pathway analysis and regulatory network analysis. miRNA-circRNA and miRNA-mRNA interactions were predicted by using TargetScan, miRanda and starBase. CircRNA-miRNA-mRNA interaction networks were created with Cytoscape. Our result showed that there were 2270 DE circRNAs between AIS patients and healthy controls. Among them, 659 were found upregulated and 1611 were downregulated. Bioinformatic analysis showed that the DE circRNAs were related to the following biological processes: endocytosis, energy metabolism, apoptosis, FoxO signaling pathway, platelet activation, neurotrophin signaling pathway and VEGF signaling pathway, which may be associated with the pathological of AIS. Three randomly selected circRNAs were successfully validated by qRT‐PCR. The results show that hsa_circ_0005548 was significantly upregulated, while hsa_circ_0000607 and hsa_circ_0002465 were significantly downregulated in AIS. Furthermore, the AUC values for hsa_circ_005548, hsa_circ_0000607 and hsa_circ_0002465 were 0.51, 0.75 and 0.69, respectively, suggesting that hsa_circ_0000607 and hsa_circ_0002465 could be potential biomarkers for AIS. In addition, Bcl2 was predicted to be a direct target of miR-337-3p, and hsa_circRNA_0000607 was predicted to act as a sponge for miR-337-3p. Thus, hsa_circ_0000607 may be involved in AIS by regulating the miR-337-3p/Bcl2 axis. Collectively, our findings indicate that numerous dysregulated circRNAs may play pivotal functional roles in AIS and hsa_circ_0000607 may play a crucial role in the pathogenesis and progression of AIS by regulating the miR-337-3p/Bcl2 axis.

## Introduction

Stroke is one of the leading causes of death and a major cause of long-term disability worldwide. Approximately 87% of all stroke cases are ischemic stroke (IS)^1^. Although major progress in the diagnosis and treatment of acute ischemic stroke (AIS) has been reported in recent years, the mortality and long-term disability rates of IS are still high. Moreover, recombinant tissue plasminogen activator (rt-PA) is currently the only effective agent approved for the treatment of AIS by the US Food and Drug Administration (FDA). Most stroke associations worldwide recommend thrombolysis therapy as the first-line treatment^2^. However, rt-PA thrombolysis is often restricted by a narrow therapeutic time window and insufficient long-term effects^3,4^. Moreover, complications of thrombolysis, including hemorrhage, neuronal damage and arterial reocclusion, are common^5,6^. Effective treatments for AIS are urgently needed, and thus, other unknown mechanisms contributing to the occurrence and development of AIS should be investigated.

Gene alterations are the key to exploring the pathological mechanism of AIS. CircRNAs represent a large new class of endogenous small noncoding RNAs that form covalently closed continuous loops without 5′ caps and 3′ poly(A) tails^7^. CircRNAs are highly homologous and characterized by a stable structure and distinct tissue-specific expression^7^. Recently, an increasing number of research studies have shown that circRNAs play a pivotal role in the modulation of post-transcriptional gene expression^8,9^. The high stability, evolutionary conservation and abundance of circRNAs in various species endow them numerous different potential functions, such as roles as efficient miRNA sponges or interactions with RNA-binding proteins to form complexes and then regulate genes at the post-transcriptional level^10^. Over the past few years, based on the potential function of circRNAs, many subsequent studies have explored the roles of circRNAs in pathological and physiological processes, and the emerging functions and specific roles of circRNAs in the initiation and progression of human diseases have been elucidated. For example, circNT5E was observed to sponge miR-422a, exhibiting tumor suppressor-like features in glioblastoma^11^. CircMTO1 suppresses human hepatocellular carcinoma progression via acting as the sponge of miR-9 to promote the expression level of P21, indicating that circMTO1 may be a potential novel therapeutic target in human hepatocellular carcinoma treatment^12^. Many studies also have demonstrated that circRNAs may be involved in the pathogenesis process of IS. Recent research has shown that the expression of circTLK1 was significantly upregulated in animal brains and circulating blood from animal models of IS and IS patients. Furthermore, knockdown of circTLK1 significantly reduced infarct volumes, decreased ischemic neuronal damage, and improved neurological deficits^13^. Another study manifested that the expression of circRNA HECTD1 was associated with risk, severity and recurrence of AIS^14^. These observations indicate that circRNAs have the potential to serve as novel diagnostic biomarkers and molecular therapeutic targets for AIS. However, as the roles of most circRNAs remain unclear, further specific studies are needed to investigate their functions in AIS.

The purpose of this study was to investigate circRNA expression profiles in AIS in the South Chinese Han population. We applied RNA-seq analysis and conducted extensive bioinformatic analyses to identify DE circRNAs in AIS. The roles and functions of the DE circRNAs were predicted by annotation of the host genes. Potential circRNA-miRNA-mRNA network targeting relationships were created by Cytoscape software based on the RNA-seq data and bioinformatic prediction results.

## Results

### Identification of transcripts in AIS

**Figure 1:**
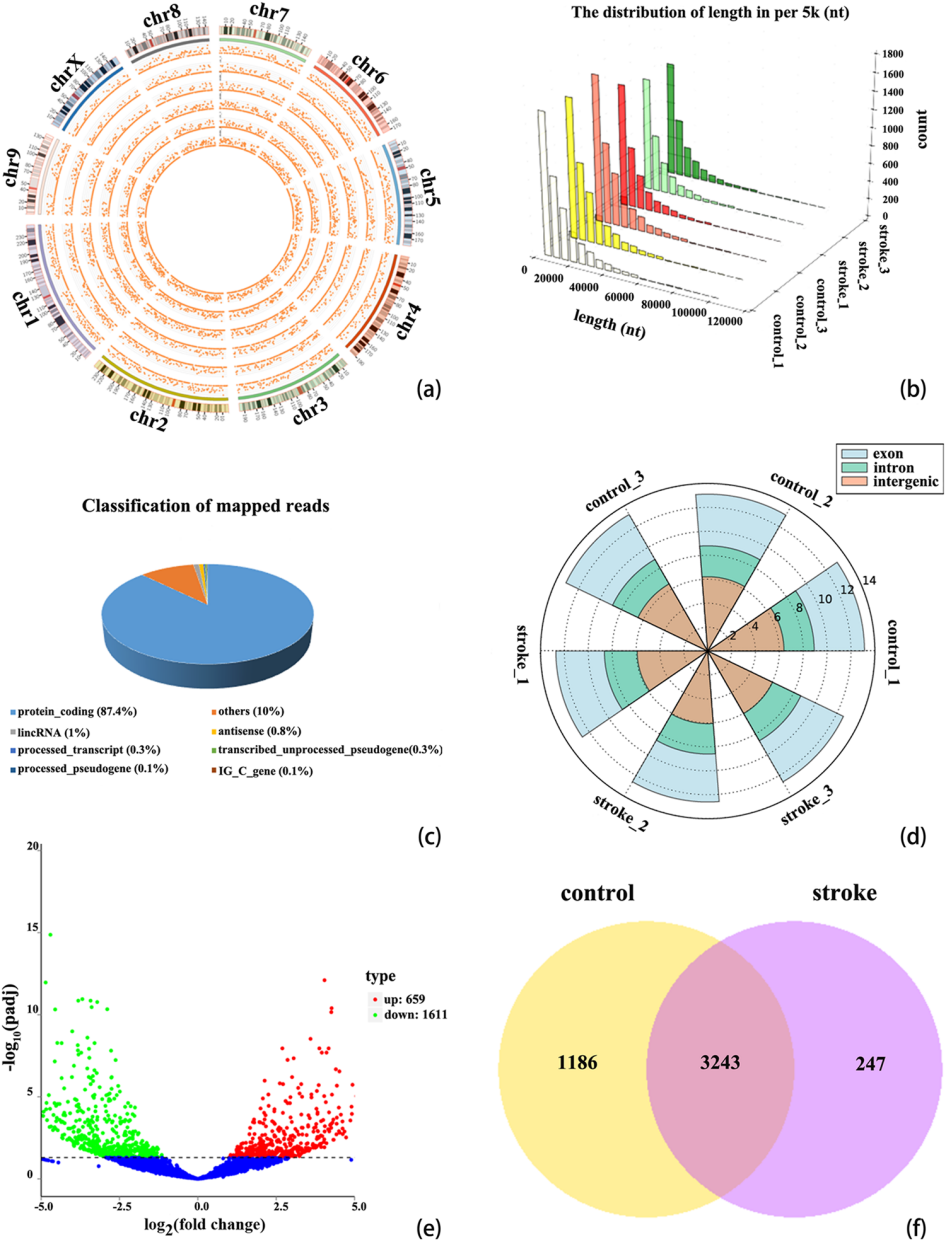
Profiles of circRNAs in patients with AIS and healthy controls. (**a**) Distribution of the DE circRNAs on human chromosomes. A circos plot diagram shows the locations of the circRNAs on human chromosomes. (**b**) The distribution of the lengths of all circRNAs. (**c**) Classification of the known genes based on genomic origin. (**d**) CircRNA classification and distribution in AIS patients and normal healthy controls. The figure shown the number of circRNAs from exon, intron and intergenic circRNA in each sample. (**e**) Volcano plot displaying dysregulated circRNAs between AIS patients and normal healthy controls. The horizontal gray line represents a P value of 0.05. The blue points represent circRNAs with no significant difference. The green points represent significantly downregulated circRNAs in patients with AIS, and the red points represent significantly upregulated circRNAs in patients with AIS. (**f**) Venn diagram analysis presenting the number of unique and common circRNAs found in patients with AIS and healthy controls. The analysis comprises 3490 and 4429 circRNAs identified in patients with AIS and healthy controls, respectively. Of these, 3243 circRNAs overlap between the two groups.

RNA-seq was conducted by using circulating blood from three patients with AIS and three normal healthy controls. The circRNAs were identified by CIRI2 and Find_circ software. After mapping the reference sequence, we identified all the DE circRNAs. These dysregulated circRNAs were widely distributed across almost all human chromosomes, including the sex chromosomes (Fig. 1a). The length distribution of these circRNAs are shown in Fig. 1b and the number of circRNAs from exon, intron and intergenic circRNA in each sample is shown in Fig. 1d. The source composition of circRNAs classified according to the source region is shown too. The statistics for the known genes are shown in Fig. 1c: protein_coding accounts for 87.4%, others account for 10.0%, lincRNA accounts for 1%, antisense accounts for 0.8%, transcribed_unprocessed_pseudogene accounts for 0.3%, processed_transcript accounts for 0.3%, processed_pseudogene accounts for 0.1%, and IG_C_gene accounts for 0.1%. The number of unique and common circRNAs between AIS patients and normal healthy controls is shown in Fig. 1f.

### Expression profiles of circRNA in AIS

**Figure 2:**
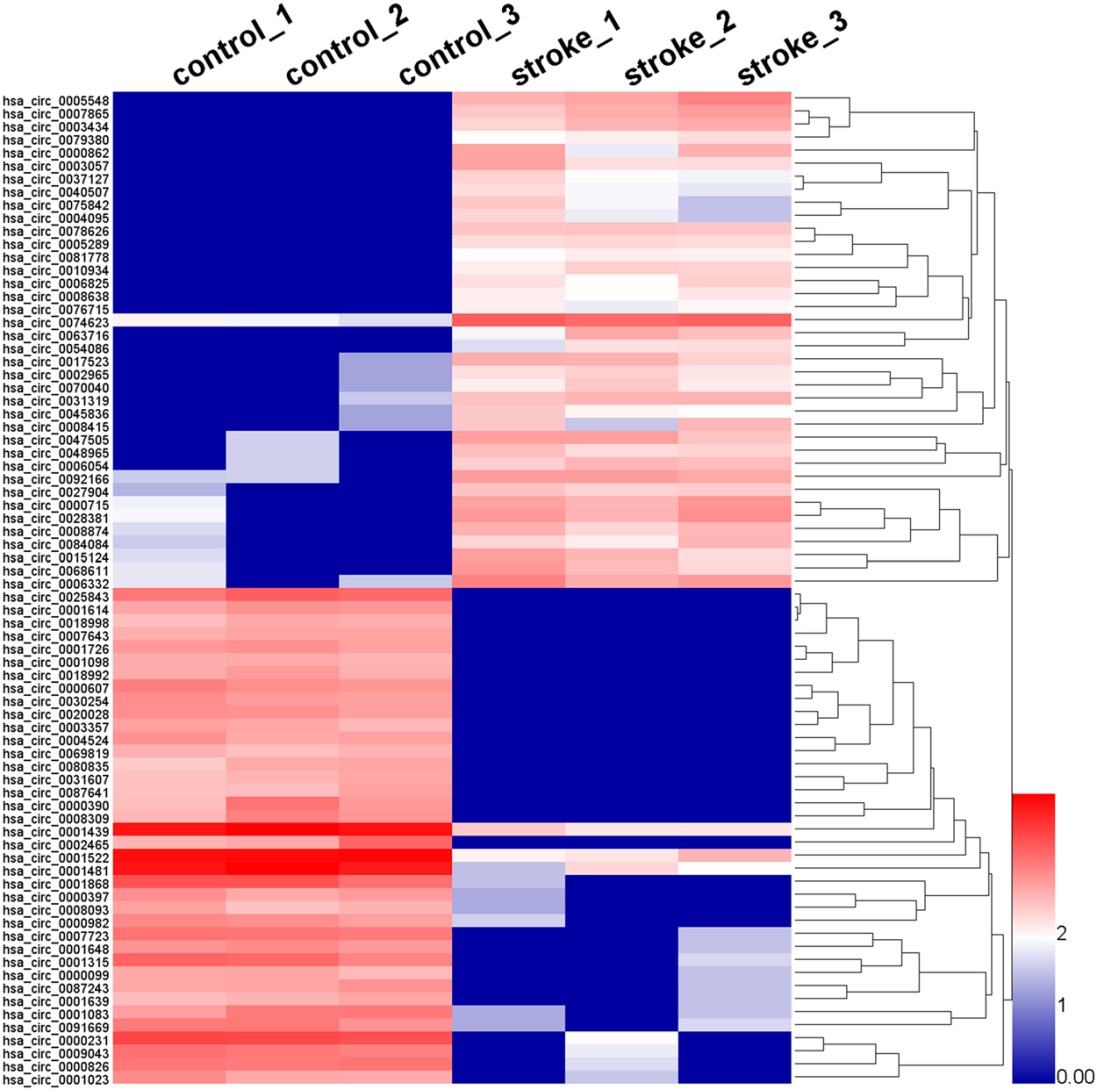
Hierarchical clustering of representative significantly DE circRNAs in patients with AIS. The expression levels of circRNAs were represented by a color scale. “blue” represents the low relative expression level, and “red” represents the high relative expression level. Each column represents a circulating blood sample, and each row represents a single circRNA. The representative DE circiRNAs were clustered with log10 (TPM + 1) values.

The significant DE circRNAs between the AIS patients and normal healthy controls were determined by padj<0.05. After screening DE circRNAs by filtering, we identified 2270 DE circRNAs. Compared with normal healthy controls, AIS patients had 659 significantly upregulated and 1611 significantly downregulated circRNAs. Volcano plots visualized the significant DE circRNAs in AIS. The red points in the plot indicate the significantly upregulated circRNAs. The green points in the plot indicate the downregulated circRNAs (Fig. 1e). Most DE circRNAs were derived from exons (Fig. 1d). Hierarchical clustering analysis manifested that circRNA expression pattern were distinguishable between the AIS patients and normal healthy controls (Fig. 2). The top 30 DE circRNAs are listed in Table 1.

**Table 1:** The top 30 up-regulated and down-regulated circRNAs in patients with AIS. ^a^FC = fold change.

CircRNA ID	Log_2_FC^a^	*P-*value	regulation	chromosome	CircRNA type	Gene name
novel_circ_0021145	8.7368	0.000032709	up	chr9	exon	ZER1
hsa_circ_0005548	8.7262	0.000035321	up	chr11	exon	AMBRA1
novel_circ_0004646	8.7229	0.000034871	up	chr14	exon	NRXN3
novel_circ_0002474	8.3258	0.00010161	up	chr12	exon	HECTD4
hsa_circ_0007865	8.2947	0.00011083	up	chr16	exon	HAGH
novel_circ_0001647	8.259	0.00011926	up	chr11	exon	HBB
novel_circ_0006235	8.1415	0.00015679	up	chr16	exon	RANBP10
hsa_circ_0003434	8.0205	0.00019992	up	chr17	exon	PLEKHM1
novel_circ_0013810	7.996	0.00021557	up	chr2	intron	—
novel_circ_0009865	7.9834	0.00020995	up	chr1	exon	ABCB10
novel_circ_0014956	7.9782	0.00024356	up	chr3	exon	TFRC
hsa_circ_0000862	7.9705	0.00027996	up	chr18	intron	PQLC1
hsa_circ_0003057	7.876	0.00031033	up	chr5	exon	ANKH
hsa_circ_0078626	7.7969	0.00031233	up	chr6	exon	AGPAT4
hsa_circ_0063716	7.7249	0.00041843	up	chr22	exon	ARHGAP8
hsa_circ_0025843	−9.6453	0.000001997	down	chr12	exon	FGD4
novel_circ_0008783	−9.4869	0.000003452	down	chr1	exon	PTPN22
novel_circ_0010086	−8.8979	0.000021524	down	chr1	exon	ZNF124
hsa_circ_0000607	−8.8906	0.000021331	down	chr15	exon	VPS13C
hsa_circ_0002465	−8.8169	NA	down	chr6	exon	CD109
novel_circ_0010762	−8.7518	0.000032106	down	chr1	exon	PRKACB
hsa_circ_0000390	−8.6973	0.000040398	down	chr12	exon	FGD4
hsa_circ_0020028	−8.6678	0.00003959	down	chr10	exon	SHOC2
novel_circ_0021440	−8.6637	0.000039925	down	chr9	exon	ZCCHC7
hsa_circ_0008309	−8.6045	0.000049377	down	chr2	exon	CUL3
hsa_circ_0030254	−8.5949	0.000048769	down	chr13	exon	SETDB2
hsa_circ_0001726	−8.5772	0.000050625	down	chr7	intron	ZNF789
hsa_circ_0001614	−8.5667	0.000052178	down	chr6	exon	SENP6
novel_circ_0007060	−8.5424	0.000089146	down	chr17	exon	EFCAB13
hsa_circ_0004524	−8.4746	0.00006788	down	chr3	exon	CEP70

### GO and KEGG analyses

**Figure 3:**
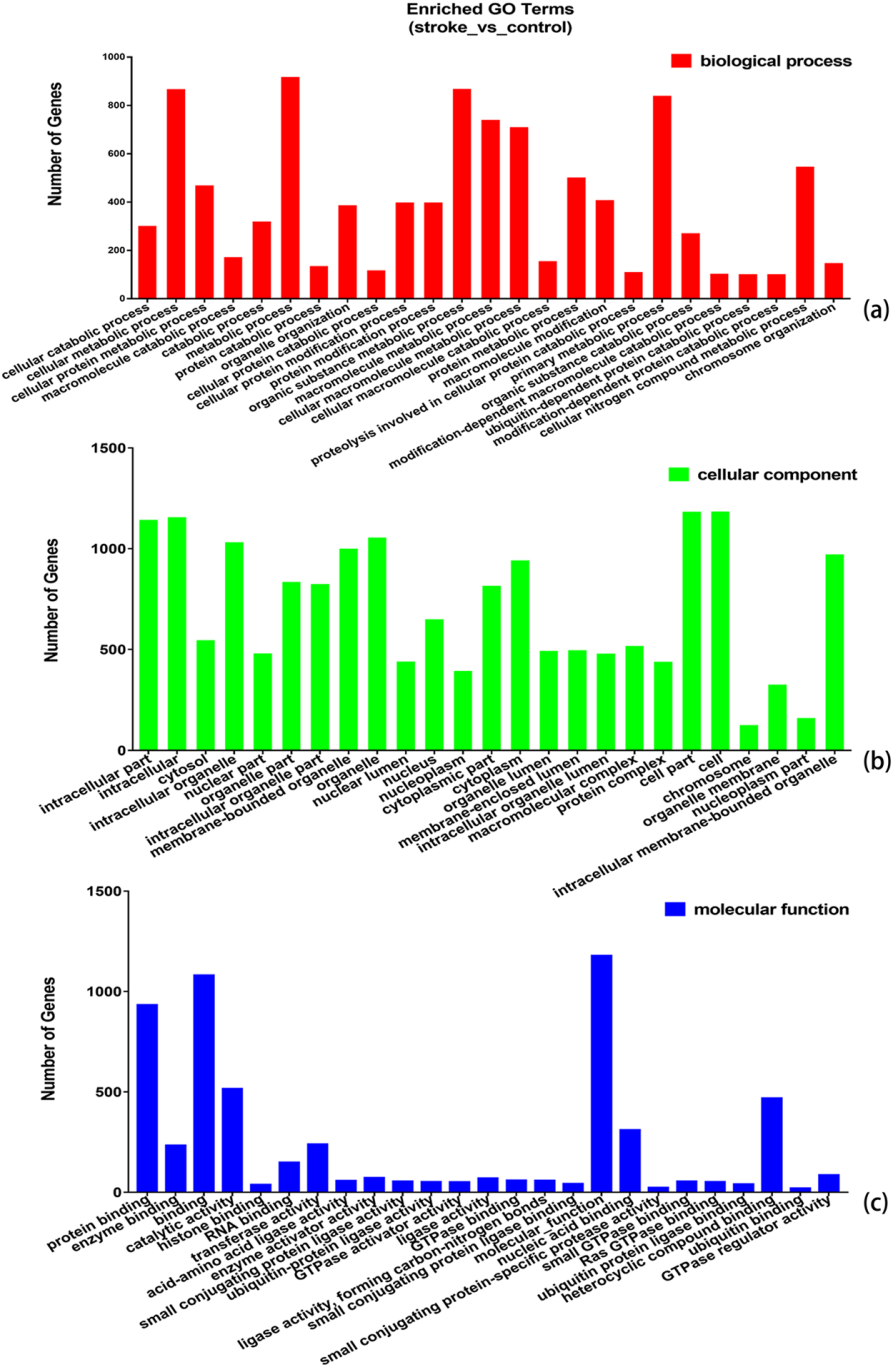
GO annotation enrichment analysis of the host genes of DE circRNAs in AIS patients versus healthy controls. (**a**) Biological process ontology. (**b**) Cellular component ontology. (**c**). Molecular function ontology.

**Figure 4:**
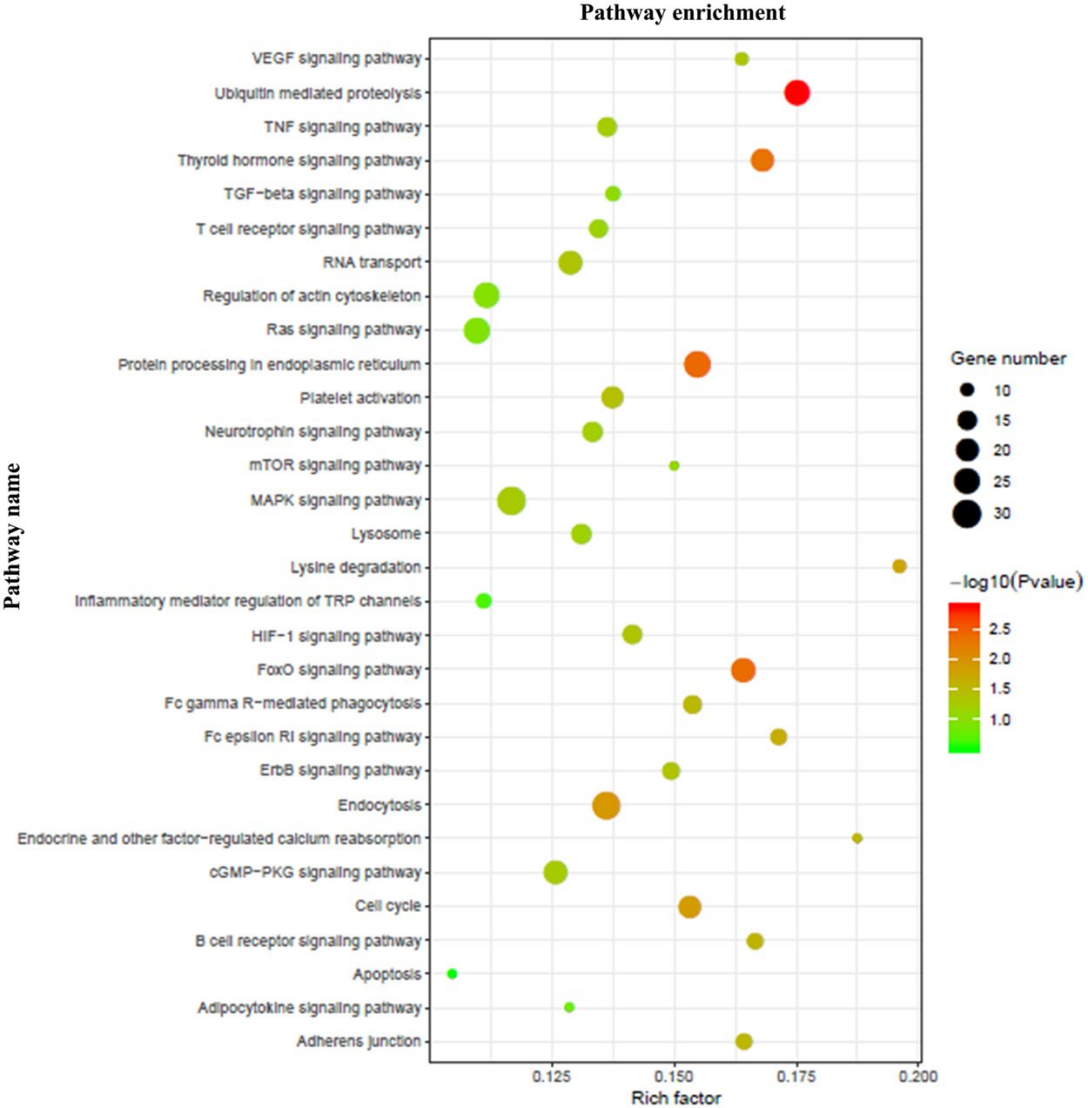
The bubble map of KEGG pathway enrichment analysis of the host genes corresponding to DE circRNAs. Rich factors are presented as enrichment degree of DE genes. The Y axis shows the names of the enriched pathways. Meanwhile, the area of each node represents the number of enriched host genes corresponding to DE circRNAs. The -log_10_(P value) is represented by a color scale. It was shown that the statistical significance increased from green to red (red represents high significance, while green represents low).

GO annotation, enrichment and KEGG pathway analyses of the host genes of significantly dysregulated circRNAs were performed to predict the functions of circRNA and molecular interactions among the genes. Based on sequence homology, the host genes of the DE circRNAs in AIS were assigned GO terms. The GO terms are divided into three categories: biological process, cellular component and molecular function. The results of GO analysis indicated that biological process mainly consisting of catabolic process, metabolic process, cellular macromolecule metabolic process, cellular macromolecule catabolic process and macromolecule modification, whereas cellular component mainly included intracellular, cytosol, cytoplasm, intracellular organelle, cell, nucleoplasm and protein complex, and molecular function mainly included enzyme binding, histone binding, catalytic activity, molecular function, transferase activity, RNA binding, protein binding, heterocyclic compound binding, GTPase activator activity and nucleic acid binding (Fig. 3a–c). The results showed possible changes in the cellular and molecular components and metabolism of peripheral blood in patients with AIS compared with healthy controls. The characteristics of these changes are closely related to the pathological mechanisms of brain damage. We then carried out a pathway enrichment analysis and found that the host genes of the DE circRNAs in AIS were assigned to various pathways. The most significantly enriched KEGG pathways were FoxO signaling pathway, neurotrophin signaling pathway, cell cycle, platelet activation, T cell receptor signaling pathway, endocytosis, lysine degradation, protein processing in the endoplasmic reticulum, VEGF signaling pathway, TNF signaling pathway and apoptosis (Fig. 4).

### Identification of circRNA-miRNA networks

**Figure 5:**
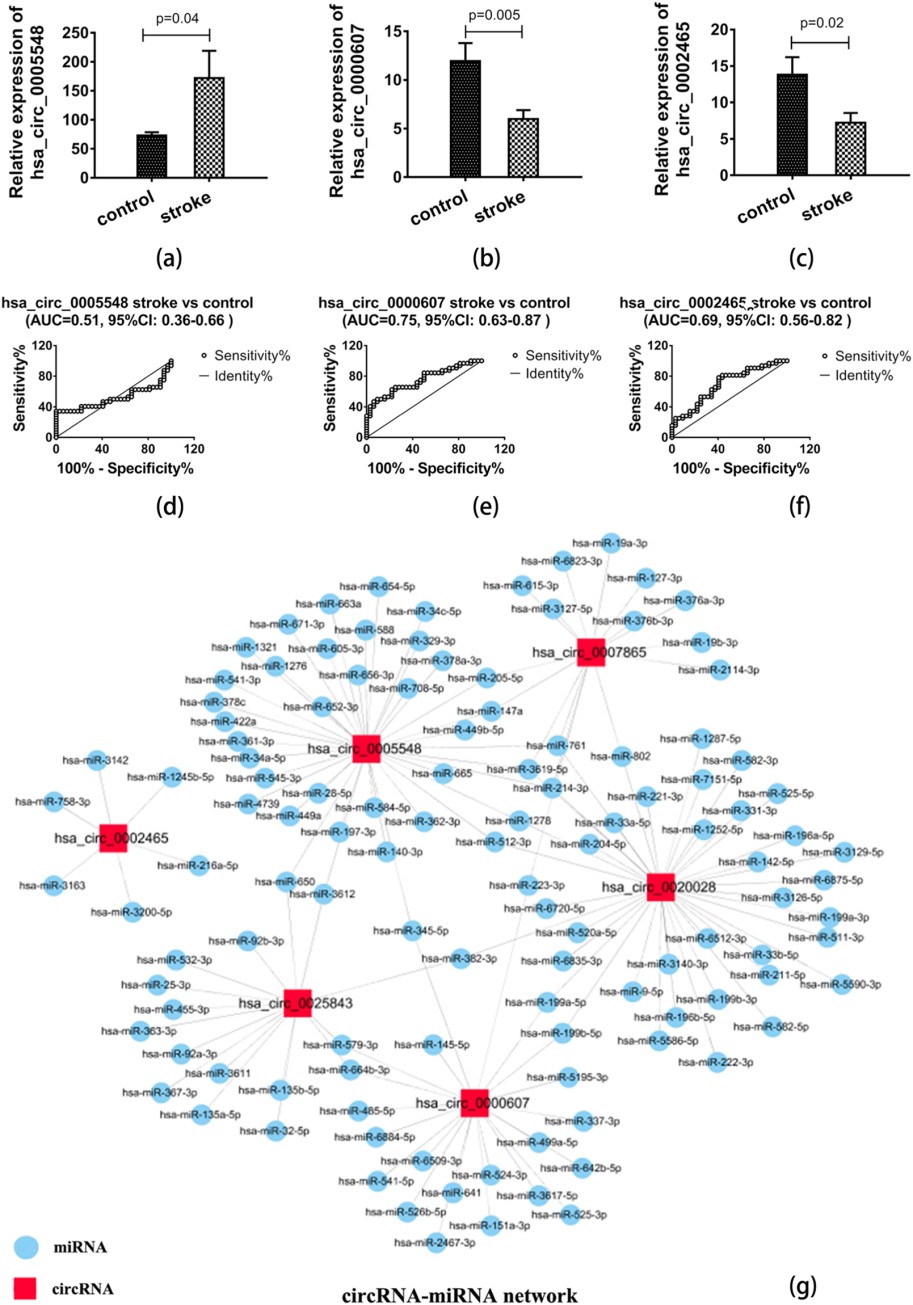
The relative expression and potential diagnostic values of circRNAs and the miRNA-circRNA interaction network in AIS. (**a**) The expression level of hsa_circ_005548 was upregulated in AIS patients compared with normal healthy controls. (**b**) The expression levels of hsa_circ_0000607 were downregulated in AIS. (**c**) AIS patients presented decreased expression of hsa_circ_0002465. (**d–f**) Potential diagnostic values of hsa_circ_005548, hsa_circ_0000607 and hsa_circ_0002465 in AIS. The AUC values for hsa_circ_005548, hsa_circ_0000607 and hsa_circ_0002465 were 0.51 (95% CI 0.36–0.66), 0.75 (95% CI 0.63–0.87) and 0.69 (95% CI 0.56–0.82), respectively, suggesting that hsa_circ_0000607 and hsa_circ_0002465 could be used as potential diagnostic biomarkers for the diagnosis of AIS. (**g**) The diagram shows the interaction network between miRNAs and the top six DE known circRNAs in AIS. Red rectangles indicate circRNAs, blue circles represent miRNAs, and a link between the nodes indicates the target relationship. Data are shown as the mean ± SEM of at three independent experiments.

Remarkably, emerging evidence has manifested that some circRNAs might function as miRNA sponges to regulate gene expression and play a crucial role in various diseases. Some circRNAs have been demonstrated to have multiple binding sites and act as miRNA sponges, functioning as transcriptional regulatory factors in organisms. In the present study, the IRESfinder software was also used for the prediction of the IRES elements of certain circRNA. The interactions between miRNAs and circRNAs were mainly predicted by miRanda and starBase software to assess the potential function of dysregulated circRNAs in AIS. Combined with the results of GO and KEGG pathway analysis indicated that some circRNAs may play an important role in AIS through functioning as miRNA sponges. We focused on the miRNAs with the strongest relationships with the top six DE circRNAs, which were mainly predicted by starBase and miRanda software. The miRNA-circRNA regulatory network is presented in Fig. 5g.

### Construction and analysis of the circRNA-miRNA-mRNA network in AIS

**Figure 6:**
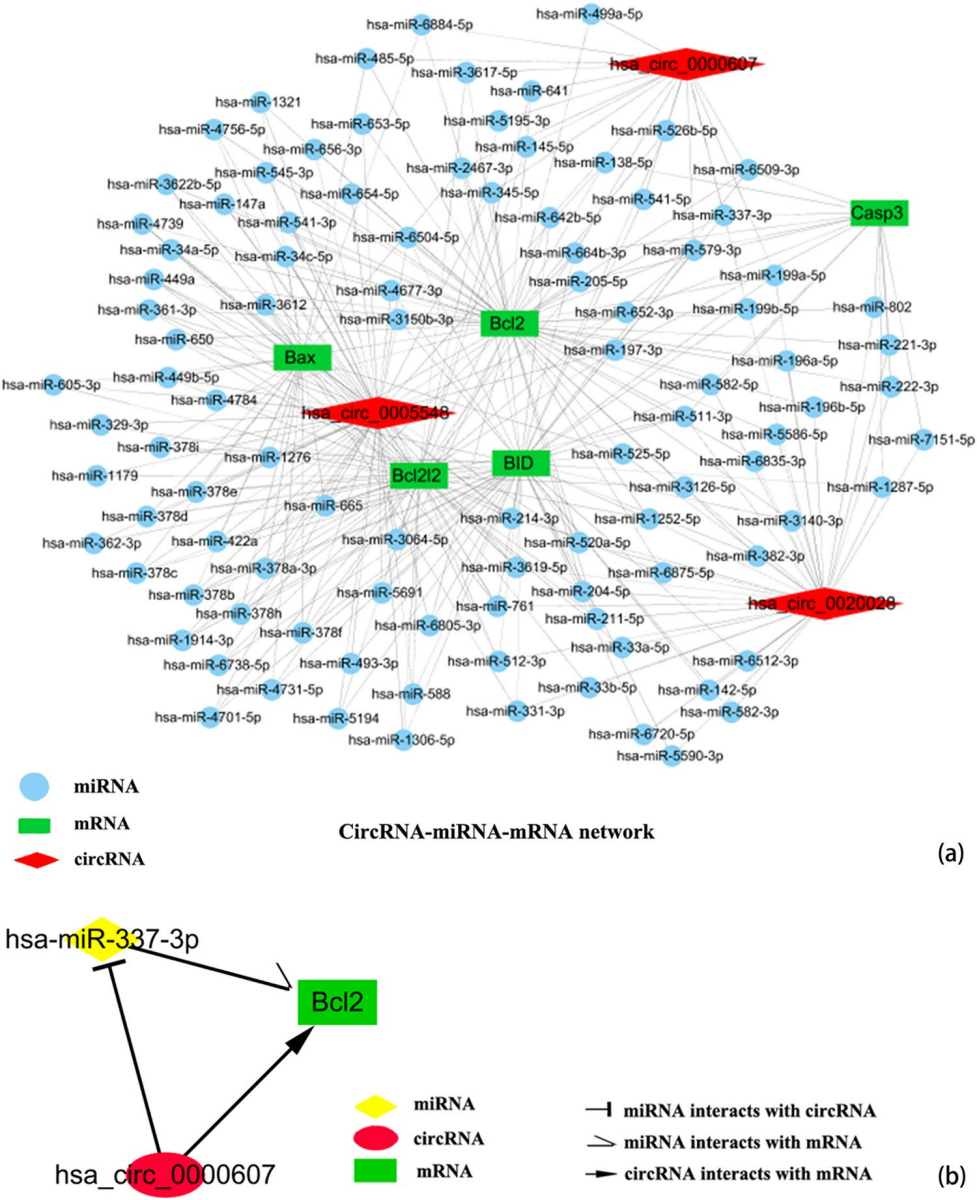
Overall regulatory networks of miRNAs, circRNAs and mRNAs. (**a**) CircRNA-miRNA-mRNA interactions are potentially associated with the pathological process of AIS. ceRNA network prediction tools showed that some significant circRNA-miRNA-mRNA pairs may be involved in AIS associated with apoptotic pathways; these molecules included hsa_circ_0005548, hsa_circ_0000607 and hsa_circ_0020028, 101 miRNAs, and five mRNAs (Bax, Bcl2, Bcl2l2, BID, and Casp3). In the figure, blue circles represent miRNAs, red diamonds represent circRNAs and green rectangles represent mRNAs. (**b**) The possible binding miRNAs and mRNAs in hsa_circ_0000607-related AIS. One direct target of miR-337-3p was Bcl2, and hsa_circ_0000607 may potentially bind to hsa-miR-337-3p. Pathway analysis based on the hsa_circ_0000607/hs-miR-337-3p/Bcl2 network. The bioinformatic prediction of hsa_circ_0000607 target genes associated with AIS and apoptosis pathways and the miRNA-targeted mRNAs were predicted via TargetScan, miRanda and starBase.

Recently, increasing number of studies have demonstrated that circRNAs can function as efficient miRNA sponge molecules, regulators of splicing and transcription, and modifiers of host genes expression via adsorbing specific miRNAs. In this study, the potential target miRNAs of the top ten most significantly dysregulated circRNAs involved in AIS were predicted by starBase (http://starbase.sysu.edu.cn/) and miRanda. The target genes of the miRNAs were predicted by TargetScan. The functions of the circRNAs were elucidated on the basis of the functions of the potential target genes of miRNAs. To further investigate the potential functional role of circRNA-miRNA interactions in patients with AIS, we screened miRNAs may be related to the apoptosis pathway. The TargetScan, starBase and miRanda databases were used to predict whether there was a regulatory relationship among these molecules. The results shared by starBase, miRanda and TargetScan were selected as the final miRNA-gene relationships. From the analysis, 3 of the top significantly DE circRNAs and 5 genes associated with AIS and 101 miRNAs were obtained. Combined with the conventional analysis of miRNA-circRNA regulatory relationships and miRNA-mRNA regulatory relationships, miRNA-circRNA relationships were integrated into the miRNA-AIS-related genes, and a network diagram of circRNA-miRNAs-AIS-related genes was obtained. The predicted results were used to generate the circRNA-miRNA-mRNA network diagram (Fig. 6). The network was based on the 3 significantly DE circRNAs and target genes with experimentally supported or predicted miRNAs that may involve in apoptosis. Upregulated (or downregulated) circRNAs targeting miRNAs in the apoptosis pathway were determined as potential functional circRNAs related to apoptosis. We found that among the top 10 DE circRNAs, hsa_circ_0005548, hsa_circ_0000607 and hsa_circ_0020028 could potentially bind to the miRNAs may be involved in the apoptosis pathway (Fig. 6a). Collectively, the above results suggested that these three DE circRNAs might play crucial functional roles in the initiation and development of AIS by interacting with miRNAs associated with the apoptosis pathway.

### Validation of selected circRNA expression levels

Combined with bioinformatic analysis and for verification of the accuracy of the RNA-Seq data, RT-PCR was conducted for one upregulated (hsa_circ_0005548) and three downregulated (hsa_circ_0000607, hsa_circ_0020028 and hsa_circ_0002465) circRNAs selected from the top thirty DE circRNAs. However, the melt curve for hsa_circ_0020028 showed nonspecific amplification, so we removed it. The expression level of the remaining three circRNAs was detected in 32 AIS patients and 32 healthy controls. Our results manifested that the expression level of hsa_circ_0005548 was significantly upregulated ((Fig. 5a), while the expression level of hsa_circ_0000607 and hsa_circ_0002465 was significantly downregulated in AIS (Fig. 5b,c). Our results manifested that the expression trends of these three circRNAs are consistent with RNA-Seq data, further confirming the reliability of our RNA-Seq data. As shown in Fig. 5b, the further qRT-PCR validation found that hsa_circ_0000607 was the most significantly DE circRNA, and furthermore bioinformatic analyses implied that it could potentially bind to miR-337-3p, which was associated with both in the apoptosis pathways and miRNAs in AIS with experimentally supported. Hence, to further explore the functional roles of circRNA in AIS, we selected hsa_circ_0000607 as a candidate circRNA for further investigation. These results suggested that hsa_circ_0000607 might play a key functional role in the progression of AIS (Fig. 6b).

### Evaluation of diagnostic value of circRNAs in AIS with ROC analysis

ROC curve analysis was performed to assess the potential diagnostic value of the circulating significantly dysregulated circRNAs. The AUC values for hsa_circ_005548, hsa_circ_0000607 and hsa_circ_0002465 were 0.51 (95% CI 0.36–0.66), 0.75 (95% CI 0.63–0.87) and 0.69 (95% CI 0.56–0.82), respectively (Fig. 5d–f). The ROC curves of the three confirmed DE circRNAs manifested that the expression levels of hsa_circ_0000607 and hsa_circ_0002465 could differentiate the AIS patients and healthy controls. The highest AUC value was found for hsa_circ_0000607 (AUC 0.75, 95% CI 0.63–0.87). Thus, hsa_circ_0000607 may be the most valuable circRNA among these three circRNAs as a potential biomarker for AIS diagnosis (Fig. 5e).

## Discussion

CircRNAs were initially thought to be molecular flukes or products of aberrant RNA splicing or splicing errors with little functional potential due to their low expression^10^. However, with the rapid development of novel high-throughput deep sequencing technology and bioinformatics, accumulating evidence has shown that numerous circRNAs are abundant, endogenous, stable and evolutionarily conserved in mammalian cells^7,15^. The high stability and specificity of circRNAs indicate that they can be used as potential diagnostic biomarkers for various disorders; moreover, certain circRNAs have been suggested as translational and transcriptional regulators in the progress and pathological changes of many diseases^16–19^. Notably, an increasing number of research studies have demonstrated that circRNAs can influence gene expression via different regulatory mechanisms, including competing with linear splicing, acting as miRNA sponges, binding to mRNA-related proteins and regulating gene expression at epigenetic, transcriptional and post-transcriptional levels^8,20,21^. Based on the evidence currently available, circRNAs can play a pivotal regulatory role in different disorders through different mechanisms.

Recent studies have manifested that many circRNAs are highly abundant, dynamically expressed and conserved in the mammalian brain^22^. Moreover, previous studies have shown that circRNAs might involve in the pathological process of several nervous system diseases, including neuropathic pain, multiple system atrophy, Alzheimer’s disease, and IS^23–26^. Furthermore, it is known that the expression level of circRNAs is altered after AIS, but the functional importance of circRNAs in AIS is still unclear. The present study aimed to elucidate the potential function of circRNAs in the pathological process of neuronal injury after AIS. Our results regarding the expression profile and potential function of circRNAs indicated that they are promising biomarkers for evaluating the neuronal injury caused by AIS.

To the best of our knowledge, this is the first study to analyze the circRNA expression profile in AIS in a South Chinese Han population. In this study, RNA-seq data was generated from 3 AIS patients and 3 healthy controls^27^. The circRNAs were detected and identified by CIRI2 and Find_circ software. Our results showed that a total of 2270 circRNAs, including 659 upregulated and 1611 downregulated circRNAs, which displayed significant differential expression in AIS patients compared with normal healthy controls. Furthermore, we investigated the potential functions of the significantly DE circRNAs in AIS via GO enrichment and KEGG pathway analyses, which indicated that DE circRNAs are associated with many pathologic processes of AIS. The results of GO enrichment analysis manifested that the main significantly enriched GO terms were metabolic process, catabolic process, intracellular, organelle, cell, molecular function, protein binding and transferase activity. This result showed possible changes in the cellular and molecular components and their function and metabolism of circulating blood in patients with AIS compared with healthy controls. The characteristics of these changes are tightly linked the pathological mechanisms of AIS. As we all know, normal cerebral function is closely related to adequate oxygen and glucose availability and to maintain energy metabolism homeostasis. AIS can lead to extensive perturbation in various metabolisms including glycogen metabolism, oxygen metabolism, lipoprotein metabolism, cellular metabolism, neuronal metabolism and so on, which may contribute to neuronal dysfunction and cell death^28–33^. AIS is the combinatorial effect of various pathological processes including the oxidative stress, perturbation in energy metabolism nervous excitability toxicity, intracellular calcium overload, inflammatory responses and apoptosis. To sum up, the results of GO analysis suggest that DE circRNA may play an important role in the occurrence and development of AIS. The most significantly enriched KEGG pathways were ubiquitin-mediated proteolysis, FoxO signaling pathway, endocytosis, lysine degradation, protein processing in the endoplasmic reticulum, T cell receptor signaling pathway, cell cycle, platelet activation, VEGF signaling pathway, TNF signaling pathway and apoptosis. These results are consistent with previous studies showing that the pathological mechanism of IS may involve excitotoxicity, oxidative stress, microvascular injury, inflammation and apoptosis^34,35^. We then carried out a pathway enrichment analysis and found that some host genes of the DE circRNAs in AIS were assigned to apoptotic pathways. In this study, the potential target genes of miRNA were predicted by using TargetScan, miRanda, TargetFinder and starBase software and a circRNA-miRNA interaction network for the top six known DE circRNAs was constructed by using Cytoscape software. Furthermore, miRNAs might involve in the apoptosis pathway were mainly screened by using TargetScan. Based on the analysis of combining the results from TargetScan, miRanda, TargetFinder and starBase, a circRNA-miRNA-mRNA interaction network potentially related to the apoptosis was generated by using Cytoscape. The interaction network analysis indicated that most miRNAs targeted by the top six DE circRNAs were related to apoptosis. Finally, we screened the target miRNAs of the top six DE circRNAs in accordance with the apoptosis pathway, and their target genes associated with AIS in the apoptosis pathway were identified. Among the top ten dysregulated known circRNAs, hsa_circ_0005548, hsa_circ_0000607, hsa_circ_0007865, hsa_circ_0020028, hsa_circ_0025843 and hsa_circ_0002465 may potentially bind to miRNAs associated with the apoptosis pathway. Collectively, our results indicated that the significantly known DE circRNAs might play a key functional role in the initiation and development of AIS via interacting with miRNAs which might associate with the apoptosis pathway. However, further studies are necessary to focus on these DE circRNAs and their potential related miRNAs and mRNAs. In summary, GO annotation, enrichment analysis and KEGG pathway analysis indicated that the significantly DE circRNAs in AIS might involve in the pathologic process of AIS. However, these findings are preliminary, and future research is needed to confirm them.

Our results also support previous findings that circRNAs can serve as novel diagnostic biomarkers and promising therapeutic targets for IS^26,36–39^. In the present study, based on the results of bioinformatic analyses, three of the top ten significantly DE circRNAs were selected for PCR validation in AIS patients and healthy controls. Our results manifested that hsa_circ_0005548 was significantly upregulated, while hsa_circ_0000607 and hsa_circ_0002465 were significantly downregulated in AIS. The expression trend of these three circRNAs is consistent with RNA-seq data results, therefore confirm the reliability of the RNA-Seq data. ROC analysis suggested that hsa_circ_0000607 and hsa_circ_0002465 could potentially serve as circulating diagnostic biomarkers of AIS. Among them, hsa_circ_0000607 was the most significantly DE circRNA, and bioinformatic analysis demonstrated that hsa_circ_0000607 contains multiple miRNA-binding sites that may be related to apoptosis. Further analysis found that miR-337-3p could potentially bind to hsa_circ_0000607; moreover, miR-337-3p expression level was found to increase following focal cerebral ischemia and may be involved in the apoptosis pathways in IS^40^. Hence, in order to explore the potential functions of circRNAs in the pathogenesis and progression of AIS, we selected hsa_circ_0000607 for further study. Furthermore, we predict the potential target gene of miR-337-3p is Bcl2 by TargetScan and starBase software and that hsa_circ_0000607 may be derived from the newly discovered apoptosis-related gene VPS13C^41^. Studies have shown that Bcl2 is a apoptosis-related factor and that Bcl2 can reduce brain damage induced by ischemia/reperfusion injury and enhance the survival of hippocampal neurons after ischemic damage^42,43^. A previous study also showed that Bcl2 inhibition induces apoptosis, prevents platelet activation and disrupts cellular calcium homeostasis^44^. Therefore, we speculate that hsa_circ_0000607 might involve in the pathobiological process of apoptosis via regulating the miR-337-3p/Bcl2 axis, which further impacts the progression of AIS. However, the exact regulatory mechanism of hsa_circ_0000607 in AIS is still unclear. Further integrating functional and mechanistic researches of hsa_circ_0000607 are necessary.

## Conclusions

Our finding revealed the expression profile and potential roles of circRNAs in AIS in the South Chinese Han population. Bioinformatic analyses implied that numerous abnormal expression circRNAs might play pivotal functional roles in the pathogenesis and progression of AIS. Our results manifested that the expression of hsa_circ_0000607 and hsa_circ_0002465 was decreased in AIS. ROC analysis suggested that hsa_circ_0000607 and hsa_circ_0002465 could be served as potential novel circulating biomarkers for AIS. Further bioinformatic analyses indicated that hsa_circ_0000607 may function as a miRNA sponge by regulating the miR-337-3p/Bcl2 axis in the progression of AIS and could serve as a potential therapeutic target for AIS. However, the precise roles of hsa_circ_0000607 associated with AIS and its target miRNAs and mRNAs should be validated in further studies. Taken together, our findings help elucidate the molecular mechanisms underlying AIS. The finding of this study may help future researchers to identify new circulating diagnostic biomarkers, understand the pathologic process of AIS and identify therapeutic targets for AIS. However, these findings are based on only 32 AIS patients and 32 healthy controls, and future analyses with more clinical blood samples are necessary to assess and validate the results.

## Materials and Methods

### Patients and specimens

The ethics committee of the first Affiliated Hospital of Guangxi Medical University approved this study protocol (approval ID: 2018-KY-E-063). Written consent forms were obtained from all participants or their legally authorized representatives prior to their enrollment in this study. All of the methods were carried out in accordance with the relevant guidelines and regulations. A total of 64 circulating blood samples were collected (32 AIS patients and 32 healthy subjects) from September 2018 to June 2019 in the first Affiliated Hospital of Guangxi Medical University (Nanning, China). Patients with anterior circulation large vessel occlusion who had not experienced interventional or thrombolysis therapy within three days of the onset were include. Age-and gender-matched healthy peoples who received a regular physical examination at the physical examination Center of the first affiliated Hospital of Guangxi Medical University were recruited as the normal controls. Patients were excluded if they had cancer, cerebral hemorrhage, cardiovascular disease, severe kidney or liver dysfunction and/or any other serious disorders. 4 mL of fresh circulating blood samples were collected from all of the participants in ethylenediaminetetraacetic acid (EDTA) tubes.

### Total RNA isolation

Total RNA was extracted from whole blood samples by using TRIzol® reagent (Life Technologies, Waltham, MA, USA) following the manufacturer’s recommendation. The concentration and purity of the total RNA were evaluated with a NanoDrop 2000 spectrophotometer (Thermo Scientific, MA, USA). RNA contamination and degradation were assessed with 1% agarose gel electrophoresis. The RNA integrity was evaluated by using the RNA Nano 6000 Assay Kit of the Agilent 2100 Bioanalyzer system (Agilent Technologies, CA, USA).

### Library preparation for lncRNA sequencing

Total RNA extracted from blood that met the following requirements were used to construct transcriptome sequence libraries and in downstream experiments: the RIN (RNA integrity number) ≥7.5 and the 28S:18S rRNA Ratio ≥1.5. RNA sequencing libraries were constructed and sequenced by Beijing Novogene Bioinformatics Technology Co., Ltd., Beijing, China (www.novogene.com/). Five micrograms of RNA from each circulating blood sample was used as initial material for the RNA sample preparations. First, we used the Epicentre Ribo-Zero™ rRNA Removal Kit (Epicentre, Madison, WI, USA) to remove ribosomal RNA and then removed the rRNA-free residue by using ethanol precipitation. Second, the linear RNA in one µg of RNA was digested with 3 U of RNase R (Epicentre, Madison, WI, USA). The RNA sequencing libraries were created via the NEBNext® Ultra™ Directional RNA Library Prep Kit for Illumina® (NEB, Ispawich, USA) according to the manufacturer’s protocols. Briefly, fragmentation was carried out in NEBNext First Strand Synthesis Reaction Buffer (5×) by using divalent cations with elevated temperature. The first-strand cDNA was synthesized by using the Random hexamer primers and M-MuLV Reverse Transcriptase (RNase H-). Subsequently, RNase H and DNA polymerase I were used to synthesize the second-strand cDNA. Then, in the reaction buffer, dNTPs with dTTP were replaced by dUTP. Furthermore, the remaining overhangs were transformed into blunt ends through polymerase and exonuclease activities. NEBNext Adaptor with hairpin loop structure was ligated to prepare for hybridization after adenylation of the 3’ ends of the DNA fragments. To obtain cDNA fragments with lengths of 150~200 bp, we first purified the library fragments via the AMPure XP system (Beckman Coulter, Beverly, MA, USA). And then, three μl of USER Enzyme (NEB, Ispawich, USA) was incubated with size-selected, adaptor-ligated cDNA fragments at 37 °C for 15 minutes followed by 5 minutes at 95 °C before PCR. Next, PCR amplification was carried out with Universal PCR primers, Index (X) Primer and Phusion High-Fidelity DNA polymerase. Finally, the PCR products were purified with the AMPure XP system. The library quality was evaluated by using the Agilent 2100 Bioanalyzer system.

### Clustering and sequencing

The clustering of the index-coded samples was carried out on a cBot Cluster Generation System by using TruSeq PE Cluster Kit v3-cBot-HS (Illumina) following the manufacturer’s recommendations. After cluster generation, the library preparations were sequenced by using the Illumina HiSeq™ 4000 sequencing platform (Illumina, San Diego, CA, USA) with a single-ended read length of 150 bp. Raw reads in the FASTQ format were first processed via in-house perlscripts. The quality of the subsequent analysis is affected by low-quality reading segments, sequencing joints, adapters, short-length sequences and high-N-rate sequences in the raw sequencing data. To ensure that the follow-up analysis was performed smoothly, we filtered the original sequencing data to obtain high-quality clean data to insure the accuracy of all the subsequent analyses. Meanwhile, the Q20, Q30 and GC-contents of the clean data were calculated. The selected high-quality clean data were used for downstream analysis.

### Mapping to reference genome and circRNA identification

Gene model annotation files and reference genome were downloaded from genome website. Bowtie2 v2.2.8 was used to index the reference genome and the paired-end clean reads were aligned to the reference genome by using Bowtie^45^. The circRNAs were detected and identified by using CIRI2^46^ and find_circ^7^ software package, separately. We refer to CIRI^47^ and find_circ^7^ to design the basis and pipeline of circRNA identification and optimal choice of parameters, respectively. First, combining the circRNAs identified by find_circ and CIRI2, and then intersection of the two identified results based on the location of the circRNAs in the chromosome. Additionally, ensure that at least in one group the junction reads in all samples are greater than or equal to 2. Calculating the expression of known and novel circRNAs in each sample, raw counts were first normalized using TPM^48^. Normalized expression level = (readCount*1,000,000)/libsize (libsize is the sum of the circRNA read count).

### Differential expression analysis

Differential expression analysis was performed with the DESeq R package (1.10.1). DESeq offers statistical routines for measuring differential expression in digital gene expression (DGE) data via a model according to the negative binomial distribution. We adjusted the resulting P values by using the Benjamini & Hochberg method for controlling the rate of false discovery. All the genes with adjusted P-values (padj) < 0.05 found by DESeq were assigned as DE. The analysis was conducted with DESeq using default parameters.

### Bioinformatic analysis of the DE circRNAs

GO enrichment analysis for host genes of DE circRNAs was implemented using the GOseq R package, in which the gene length bias was properly corrected^49^. CircRNAs can regulate gene expression by interacting with miRNAs. The host gene functions were then predicted by GO functional annotation enrichment analysis. Gene function is divided into three separate subgroups: biological process (BP), molecular function (MF) and cellular component (CC). The results of the GO annotation enrichment analysis are presented in a barplot. The pathways related to the host genes of the DE circRNAs were performed by KEGG. MiRNA response elements as regulators of mRNAs were predicted through TargetScan software (http://www.targetscan.org/vert_72/). Target miRNAs of circRNAs were predicted by using miRanda (http://www.microrna.org/microrna/home.do) and starBase software (http://starbase.sysu.edu.cn/). We used KOBAS 3.0 to detect the statistical enrichment of DE genes or circRNA host genes in KEGG pathways^50^. The maps of the circRNA-miRNA-mRNA regulatory interaction networks were generated by using Cytoscape software.

### Validation of DE circRNAs using qPCR

To validate the DE circRNAs identified by RNA-seq was conducted using qRT-PCR analyses of 64 total RNA samples. A Thermo Scientific RevertAid First Strand cDNA Synthesis Kit (Thermo Fisher Scientific, MA, USA) was used to synthesize cDNA templates from 2 μg of total RNA. The RT-PCR amplification was performed on the ABI 7500 real-time PCR system (Applied Biosystems, CA, USA) by using TB Green™ Premix Ex Taq™ II (TaKaRa, Dalian, China). The optimum reaction conditions were as follows: 95 °C for 30 secs for predenaturation, followed by 40 cycles of 95 °C for 5 secs and 60 °C for 30 secs. The melting curves exhibited a single peak, indicating the specificity of the amplification systems and analysis of relative gene expression. All primers for circRNAs were designed and synthesized by Sangon Biotech (Shanghai, China). GAPDH was used as the internal control. The RT-PCR results were analyzed by using the 2^−∆∆Ct^ method.

### Receiver operating characteristic curve analysis

ROC curve analysis was carried out to evaluate circRNAs as diagnostic biomarkers for AIS. The expression of circRNAs was applied to generate ROC curves to differentiate AIS patients from normal healthy controls. The area under the ROC curve (AUC) and confidence intervals were calculated to assess the predictive value of the selected circRNAs for AIS diagnosis. The solid lines in the figures indicates the diagnostic threshold value of circRNAs for AIS (AUC = 0.5).

### Statistical analysis

All statistical analyses were carried out by using SPSS software (version 22.0, IBM SPSS, Chicago, IL, USA), and figures were generated by using GraphPad Prism version 7.0 (GraphPad software, Inc., La Jolla, CA, USA), Cytoscape_v3.5.1. and Adobe Photoshop CS5. Comparisons between two independent groups were performed by Student’s t test. The potential diagnostic value of circRNAs for AIS was evaluated by ROC curves and the AUC. All data are represented as the mean ± SEM. All the experiments were repeated for three times. P values < 0.05 were considered statistically significant.

## Supplementary information

Supplementary information.

## Data Availability

The transcriptomics datasets generated during the current study have been deposited in the NCBI Gene Expression Omnibus (GEO) repository and are accessible via GEO series accession number GSE140275. (https://www.ncbi.nlm.nih.gov/geo/query/acc.cgi?acc=GSE140275).
