# Supplementary material for: Expression profile and bioinformatics analysis of circular RNAs in acute ischemic stroke in a South Chinese Han population
Source: Sci Rep. 2020 Jun 23;10:10138. doi: 10.1038/s41598-020-66990-y (PMC7311391; doi:10.1038/s41598-020-66990-y)
Supplement: Supplementary file 1 — Supplementary information. [file 41598_2020_66990_MOESM1_ESM.pdf]

Supplementary information

Expression profile and bioinformatics analysis of circular RNAs in acute ischemic stroke in a South Chinese Han population

Shenghua Li, Lan Chen, Chen Xu, Xiang Qu, Zhenxiu Qin, Jinggui Gao, Jinpin Li, Jingli Liu

stroke_vs_control.DE circRNAs

ID	stroke_readcount	control_readcount	log2(FC)	pval	padj
hsa_circ_0001439	14.7896824	629.2338212	-5.3498	6.3472E-26	2.964E-22
hsa_circ_0001481	8.065262512	601.4670998	-6.1868	7.9807E-22	1.456E-18
hsa_circ_0001522	16.74131407	666.2746833	-5.2781	9.3568E-22	1.456E-18
hsa_circ_0087391	7.976769331	215.1937078	-4.7032	1.1499E-18	1.342E-15
novel_circ_0020133	209.7459284	12.46375399	4.0399	8.4267E-16	7.869E-13
hsa_circ_0001085	5.853662677	177.8263166	-4.8569	1.389E-15	1.081E-12
hsa_circ_0000839	16.71093888	219.4278249	-3.6843	1.6754E-14	1.118E-11
hsa_circ_0001451	18.88382572	205.8928587	-3.423	2.5875E-14	1.393E-11
hsa_circ_0091382	13.93547749	197.6087774	-3.8156	2.6854E-14	1.393E-11
hsa_circ_0001707	24.33664074	228.120169	-3.2113	3.6064E-14	1.684E-11
hsa_circ_0001346	20.95572583	222.442467	-3.3893	8.1311E-14	3.451E-11
hsa_circ_0074623	131.0282593	6.766348687	4.2665	1.0305E-13	4.009E-11
hsa_circ_0000471	48.52263785	362.9973433	-2.8914	1.2876E-13	4.624E-11
hsa_circ_0004089	5.636686549	137.1418743	-4.5521	1.4271E-13	4.76E-11
novel_circ_0010890	146.8435772	7.563005115	4.2562	2.2301E-13	6.942E-11
hsa_circ_0001868	0.874939251	151.2306097	-7.01	3.5687E-13	1.041E-10
hsa_circ_0000231	2.446554613	206.2053504	-6.3474	1.814E-12	4.982E-10
novel_circ_0002839	2.346994228	104.2811922	-5.4702	1.9628E-12	5.091E-10
hsa_circ_0003632	6.085888048	99.97379625	-4.0077	4.1821E-12	1.028E-09
hsa_circ_0000396	14.08612112	164.0239523	-3.523	1.0681E-11	2.494E-09
hsa_circ_0004036	114.1530298	9.371758295	3.5944	1.3338E-11	2.965E-09
novel_circ_0007625	4.511543278	102.1670589	-4.4852	2.4631E-11	5.227E-09
hsa_circ_0001550	29.20210419	292.7339351	-3.2961	2.6575E-11	5.333E-09
hsa_circ_0067735	16.21195719	343.4462705	-4.3465	2.7413E-11	5.333E-09
hsa_circ_0001315	1.148607018	106.0757439	-6.4258	3.3044E-11	6.171E-09
hsa_circ_0000826	1.223277306	89.97055856	-6.1972	3.8399E-11	6.64E-09
novel_circ_0001345	152.7984469	3.716014939	5.2933	3.7447E-11	6.64E-09
hsa_circ_0001524	6.145309094	91.23958603	-3.8319	4.3202E-11	7.204E-09
novel_circ_0001660	75.94308828	1.00724855	6.216	5.6637E-11	9.119E-09
hsa_circ_0005720	1589.427047	105.4324708	3.8768	7.658E-11	1.117E-08
hsa_circ_0043837	700.7504989	107.2124834	2.6959	7.5425E-11	1.117E-08
novel_circ_0016587	140.938805	7.666361895	4.1723	7.455E-11	1.117E-08
hsa_circ_0001663	7.867568092	88.46153924	-3.4728	8.4568E-11	1.197E-08
hsa_circ_0007723	0.765738012	92.21665031	-6.7746	8.7934E-11	1.208E-08
hsa_circ_0091669	1.731899852	76.21711768	-5.3283	9.1008E-11	1.214E-08
hsa_circ_0000398	5.56201626	81.95133673	-3.8278	1.1439E-10	1.484E-08
hsa_circ_0001492	49.71411362	342.3460313	-2.769	1.2205E-10	1.54E-08
hsa_circ_0006660	157.9537342	8.927803796	4.1044	1.6451E-10	2.021E-08
hsa_circ_0040356	144.2312267	9.137426278	3.954	1.6939E-10	2.028E-08
hsa_circ_0007765	7.968431535	108.6220109	-3.7219	1.7524E-10	2.046E-08
hsa_circ_0009043	1.631036409	88.86561307	-5.7655	2.0394E-10	2.267E-08
novel_circ_0010889	94.15770782	1.683595786	5.778	2.0282E-10	2.267E-08
hsa_circ_0001263	7.267722958	103.4956704	-3.8097	2.4454E-10	2.655E-08
hsa_circ_0001083	1.349030846	71.30989734	-5.5456	2.8865E-10	3.063E-08
hsa_circ_0007292	14.34466294	160.9818903	-3.4625	4.055E-10	4.207E-08
hsa_circ_0058494	129.205436	15.11664339	3.0573	4.5342E-10	4.504E-08
novel_circ_0006144	1.148607018	71.93278894	-5.895	4.4393E-10	4.504E-08
hsa_circ_0008179	1021.066571	138.8228446	2.8618	5.7956E-10	5.637E-08
hsa_circ_0002538	36.12697418	225.1123088	-2.6237	6.0632E-10	5.777E-08
hsa_circ_0003764	3.75544612	95.04410263	-4.5647	7.5006E-10	7.004E-08
novel_circ_0009889	83.64729077	1.00724855	6.3068	7.7831E-10	7.125E-08
hsa_circ_0072437	5.161168604	81.22704118	-3.9534	1.4321E-09	1.286E-07
hsa_circ_0001661	6.318113418	72.82326375	-3.5291	2.121E-09	1.869E-07

hsa_circ_0000118	1.398811038	56.97794514	-5.2191	2.4703E-09	2.097E-07
novel_circ_0016791	50.40660769	2.16436403	4.4027	2.4536E-09	2.097E-07
hsa_circ_0000160	25.6387441	259.0333465	-3.2998	2.8933E-09	2.412E-07
novel_circ_0009875	55.8345589	2.268690451	4.5157	3.4523E-09	2.828E-07
hsa_circ_0001769	12.68600703	118.9918225	-3.2271	4.442E-09	3.576E-07
hsa_circ_0001460	16.36963556	171.1100542	-3.3501	6.8809E-09	5.445E-07
novel_circ_0014827	49.85772252	0.694269288	5.7291	7.0772E-09	5.507E-07
novel_circ_0002974	5.087924666	59.56399453	-3.4804	8.1005E-09	6.2E-07
hsa_circ_0000567	38.25972169	261.0772298	-2.7488	8.6539E-09	6.313E-07
hsa_circ_0004379	133.8355748	16.57915256	3.0051	8.5211E-09	6.313E-07
hsa_circ_0007411	3.13071089	53.09042066	-4.018	8.5228E-09	6.313E-07
hsa_circ_0000982	1.166585668	56.60167487	-5.2598	9.187E-09	6.599E-07
hsa_circ_0001535	58.39562399	401.7510232	-2.7598	9.6005E-09	6.792E-07
hsa_circ_0001648	0.765738012	55.54145825	-6.0615	1.0264E-08	7.153E-07
hsa_circ_0001577	2.315192685	51.87550246	-4.3459	1.0863E-08	7.246E-07
hsa_circ_0004592	5.470793671	60.94654371	-3.4205	1.0747E-08	7.246E-07
novel_circ_0015652	0.815518204	54.87366622	-6.0376	1.0776E-08	7.246E-07
hsa_circ_0058493	1030.497071	233.6923708	2.1315	1.58E-08	1.039E-06
hsa_circ_0001358	6.602848389	69.16490707	-3.3427	1.6877E-08	1.094E-06
hsa_circ_0004833	150.4221602	24.18317954	2.6141	2.3642E-08	1.512E-06
hsa_circ_0001518	8.334748241	92.13814203	-3.4197	2.699E-08	1.68E-06
hsa_circ_0002988	101.6811462	15.28751284	2.7172	2.6695E-08	1.68E-06
hsa_circ_0007313	114.8397659	11.34733082	3.2968	2.7454E-08	1.687E-06
novel_circ_0009625	38.3204458	1.134345225	4.9337	3.05E-08	1.849E-06
novel_circ_0010112	85.5048997	5.755221575	3.8558	3.2347E-08	1.936E-06
hsa_circ_0092166	42.38148452	2.017406022	4.435	3.546E-08	2.096E-06
hsa_circ_0001772	66.59512741	391.4910073	-2.5413	3.7183E-08	2.17E-06
hsa_circ_0004771	20.18165003	180.3963371	-3.1328	4.5167E-08	2.604E-06
hsa_circ_0035943	46.65246463	4.983274682	3.2003	5.0634E-08	2.883E-06
novel_circ_0020660	1.93232368	41.83498249	-4.2658	5.3856E-08	3.03E-06
novel_circ_0021918	18.41391617	124.5668213	-2.7535	5.8455E-08	3.249E-06
hsa_circ_0000397	0.583292834	47.77123845	-5.8969	5.9322E-08	3.259E-06
hsa_circ_0002024	8.307128736	101.7715729	-3.5987	6.7435E-08	3.661E-06
hsa_circ_0087243	0.765738012	44.74012596	-5.7584	7.9579E-08	4.271E-06
hsa_circ_0025006	39.06559904	2.816001731	3.797	8.3467E-08	4.429E-06
hsa_circ_0001789	35.72339712	201.9669378	-2.4882	9.1283E-08	4.789E-06
hsa_circ_0068610	106.8492707	11.07230649	3.2463	1.2118E-07	6.287E-06
hsa_circ_0006848	8.359638337	60.1016094	-2.813	1.234E-07	6.331E-06
hsa_circ_0001023	0.815518204	45.41146699	-5.7487	1.4012E-07	7.111E-06
hsa_circ_0000782	9.350690273	69.67426342	-2.8607	1.4481E-07	7.193E-06
hsa_circ_0004552	59.10622001	4.833407753	3.6218	1.4375E-07	7.193E-06
hsa_circ_0014613	124.7655608	25.96140535	2.2497	1.4743E-07	7.246E-06
hsa_circ_0006332	53.6229065	2.604439968	4.2786	1.5167E-07	7.377E-06
hsa_circ_0006556	4.212985415	42.58706805	-3.2948	1.5804E-07	7.607E-06
hsa_circ_0005379	11.6976845	71.04133094	-2.587	1.6515E-07	7.868E-06
hsa_circ_0004293	2.038795511	54.84410848	-4.7541	1.6744E-07	7.897E-06
novel_circ_0016279	7.544120133	58.85163163	-2.9194	1.7238E-07	8.048E-06
hsa_circ_0003441	7.834340199	81.84619868	-3.3482	2.0001E-07	9.07E-06
novel_circ_0009897	34.3051548	1.932940934	4.0296	2.0008E-07	9.07E-06
novel_circ_0021213	33.35281585	1.00724855	5.0532	1.9937E-07	9.07E-06
novel_circ_0005300	45.06702638	4.415132429	3.3238	2.2103E-07	9.923E-06
hsa_circ_0008803	61.82216334	7.818168105	2.9525	2.3581E-07	1.049E-05
hsa_circ_0007185	40.80559011	3.174521502	3.624	2.4783E-07	1.092E-05
hsa_circ_0000497	7.042409034	70.63822675	-3.3173	2.6481E-07	1.156E-05
hsa_circ_0007379	168.1422793	39.65269641	2.0743	2.9096E-07	1.258E-05
hsa_circ_0001434	10.35829451	67.28800114	-2.6657	3.0378E-07	1.301E-05
hsa_circ_0008833	17.53897692	125.4250336	-2.8317	3.1623E-07	1.342E-05
hsa_circ_0004127	7.108741526	58.66090084	-3.0517	3.3171E-07	1.383E-05
novel_circ_0009164	3.162512432	47.25348491	-3.9222	3.3121E-07	1.383E-05
hsa_circ_0005505	2.389862974	37.29372303	-3.8689	3.8333E-07	1.584E-05
hsa_circ_0000347	18.73045268	130.7789132	-2.7911	4.0187E-07	1.633E-05
hsa_circ_0047505	36.38824541	1.010157472	5.152	4.0219E-07	1.633E-05
hsa_circ_0087357	13.5000726	100.6305791	-2.8436	4.4214E-07	1.78E-05
hsa_circ_0006417	145.9102471	20.53417616	2.7995	4.5229E-07	1.805E-05
novel_circ_0021473	39.74281759	0.694269288	5.4109	4.637E-07	1.835E-05

hsa_circ_0001684	6.250354575	114.3186317	-4.1466	5.1263E-07	2.011E-05
novel_circ_0009890	30.95055634	1.701517838	4.0817	5.4248E-07	2.111E-05
hsa_circ_0000236	1.223277306	34.28522278	-4.8284	6.0946E-07	2.352E-05
hsa_circ_0001829	56.61967563	267.9171322	-2.2263	6.2437E-07	2.39E-05
hsa_circ_0017586	1.166585668	34.77568743	-4.5749	6.4965E-07	2.427E-05
hsa_circ_0037130	135.8964785	17.27518957	2.9357	6.4457E-07	2.427E-05
hsa_circ_0046178	58.29921545	7.821077026	2.8624	6.4597E-07	2.427E-05
hsa_circ_0000099	0.765738012	34.24162156	-5.3764	6.5784E-07	2.438E-05
hsa_circ_0007803	1.398811038	33.64716005	-4.4697	7.3264E-07	2.694E-05
hsa_circ_0085439	2.333171336	58.71580819	-4.4104	7.4705E-07	2.725E-05
hsa_circ_0001798	2.406415274	35.99449775	-3.8083	7.8951E-07	2.858E-05
hsa_circ_0008230	1.166585668	33.06738211	-4.5036	8.7561E-07	3.145E-05
hsa_circ_0000475	2.389862974	37.49461875	-3.8724	9.8153E-07	3.498E-05
hsa_circ_0000110	20.52044424	514.0703751	-4.5261	1.0192E-06	3.551E-05
hsa_circ_0003179	12.48973896	99.15307037	-2.9504	1.0179E-06	3.551E-05
hsa_circ_0050119	144.9014631	30.161269	2.2589	1.0039E-06	3.551E-05
hsa_circ_0001112	24.87849118	111.1132723	-2.1444	0.000001084	3.749E-05
hsa_circ_0007641	6.188177841	63.0506972	-3.2588	1.1399E-06	3.914E-05
novel_circ_0010906	30.49304332	1.00724855	4.9032	1.1754E-06	4.006E-05
hsa_circ_0000344	44.54448393	225.0150669	-2.3198	1.2028E-06	4.069E-05
hsa_circ_0001639	0.765738012	30.99022054	-5.244	0.000001315	4.417E-05
hsa_circ_0004368	44.44647318	180.3157912	-2.0066	1.3437E-06	4.481E-05
hsa_circ_0031319	27.00017149	0.925692384	4.4972	1.3766E-06	4.537E-05
novel_circ_0016663	2.565396706	39.29206581	-3.7911	1.3798E-06	4.537E-05
hsa_circ_0008093	0.583292834	32.36341642	-5.3506	1.4736E-06	4.811E-05
novel_circ_0013936	541.6111883	134.5398286	2.0005	1.5772E-06	5.114E-05
novel_circ_0002419	59.07043856	4.07453471	3.7839	1.6528E-06	5.322E-05
hsa_circ_0003652	1.148607018	31.42174126	-4.7159	1.6809E-06	5.375E-05
hsa_circ_0000072	4.56132347	51.5544691	-3.4874	1.7752E-06	5.638E-05
hsa_circ_0014611	66.42517579	12.11105206	2.4427	1.8092E-06	5.708E-05
novel_circ_0000163	1.148607018	34.60933662	-4.8395	1.8372E-06	5.757E-05
hsa_circ_0017523	27.39553405	0.462846192	5.3934	1.8585E-06	5.785E-05
hsa_circ_0025843	0	115.767585	-9.6453	0.000001997	6.175E-05
hsa_circ_0024193	3.372577114	48.02286599	-3.7259	2.2593E-06	6.94E-05
novel_circ_0011611	5.761013738	86.22498589	-3.8353	2.5038E-06	7.641E-05
hsa_circ_0071311	76.38938451	14.01734418	2.4383	2.5277E-06	7.663E-05
hsa_circ_0009034	6.659540028	64.96342015	-3.2694	2.5457E-06	7.668E-05
hsa_circ_0023923	10.59194623	83.07986411	-2.9249	0.000002602	7.788E-05
novel_circ_0009216	1.223277306	37.81696485	-4.9203	2.6962E-06	8.018E-05
novel_circ_0003380	2.315192685	32.09420994	-3.6616	2.9408E-06	8.69E-05
hsa_circ_0003395	26.34363471	1.809722821	3.819	3.0589E-06	8.882E-05
hsa_circ_0005288	0.815518204	27.07685888	-5.0433	3.0529E-06	8.882E-05
novel_circ_0022226	22.54804925	1.134345225	4.1847	3.0627E-06	8.882E-05
novel_circ_0004795	1.349030846	28.73363429	-4.2612	3.2317E-06	9.314E-05
hsa_circ_0000793	91.13634774	21.01996416	2.1136	0.000003298	9.447E-05
novel_circ_0002098	90.91934533	13.76121155	2.7132	3.3417E-06	9.514E-05
novel_circ_0008783	0	102.8521329	-9.4869	3.4523E-06	9.769E-05
hsa_circ_0008967	1.581256216	26.17490639	-4.0754	3.5687E-06	0.0001004
hsa_circ_0006258	31.16062102	150.7625451	-2.2503	4.0169E-06	0.0001104
hsa_circ_0008717	25.6469586	1.701517838	3.8157	3.9793E-06	0.0001104
novel_circ_0005872	87.70976395	2.685996134	4.914	4.0177E-06	0.0001104
novel_circ_0010089	22.26331428	0.462846192	5.102	3.9878E-06	0.0001104
hsa_circ_0008207	22.90329876	97.34671145	-2.0812	4.2249E-06	0.0001154
hsa_circ_0000095	7710.941587	756.2260368	3.301	4.4411E-06	0.0001206
hsa_circ_0000612	0.583292834	25.5820546	-5.0249	4.5052E-06	0.0001216
hsa_circ_0006054	23.71881696	1.010157472	4.5617	4.5537E-06	0.0001222
hsa_circ_0003045	1.166585668	28.35719247	-4.2902	4.7871E-06	0.0001277
novel_circ_0016751	0.874939251	27.43361092	-4.6059	4.8336E-06	0.0001282
hsa_circ_0003142	112.1182667	24.83578688	2.1582	5.0478E-06	0.0001332
hsa_circ_0001222	52.01836239	4.12395378	3.5289	5.1151E-06	0.0001342
hsa_circ_0032965	1.166585668	27.20298591	-4.2301	5.1905E-06	0.0001354
hsa_circ_0000211	9.065955302	56.85876359	-2.6427	0.000005392	0.0001391
hsa_circ_0001617	4.645634613	34.28910135	-2.8592	5.3748E-06	0.0001391
hsa_circ_0000711	29.98166509	122.8732322	-2.0226	5.6444E-06	0.0001448
hsa_circ_0019170	0.765738012	24.90958593	-4.9352	5.7114E-06	0.0001457

hsa_circ_0001551	3.788674013	40.83710079	-3.3587	6.3346E-06	0.0001607
hsa_circ_0025887	3.795585459	34.83641263	-3.2055	6.7815E-06	0.0001712
novel_circ_0007826	93.43896803	11.76560614	2.9577	0.000007193	0.0001806
novel_circ_0018445	60.73585635	2.685996134	4.4337	7.4194E-06	0.0001853
hsa_circ_0001746	13.41576146	77.09207825	-2.479	7.7193E-06	0.000191
hsa_circ_0007646	0.874939251	25.23015076	-4.5004	7.7309E-06	0.000191
novel_circ_0002443	27.57512652	2.06197689	3.569	8.0979E-06	0.000199
hsa_circ_0006208	3.896448901	31.2017823	-2.9682	9.2144E-06	0.0002253
hsa_circ_0002058	541.6259256	122.5037032	2.13	9.8404E-06	0.0002393
hsa_circ_0008620	0.583292834	30.04353917	-5.2294	0.000009918	0.0002399
hsa_circ_0001937	4.796278249	35.51275986	-2.8282	0.000010085	0.0002415
hsa_circ_0003184	143.0522708	41.11794295	1.7909	0.000010086	0.0002415
hsa_circ_0000715	41.36541919	2.014497101	4.3226	0.00001019	0.0002427
hsa_circ_0001520	8.508978915	101.2730215	-3.5106	0.000010354	0.0002454
hsa_circ_0000566	3.98767149	38.01351349	-3.2149	0.000010688	0.000252
novel_circ_0021868	2.505975659	35.94170123	-3.7699	0.000010763	0.0002525
novel_circ_0021952	10.68174247	53.71478301	-2.3133	0.000010859	0.0002535
hsa_circ_0000280	1.148607018	25.2632575	-4.4075	0.000011005	0.0002556
novel_circ_0012021	31.51444417	1.619961672	3.9992	0.000011152	0.0002578
hsa_circ_0001924	0.815518204	23.08581962	-4.8107	0.00001148	0.0002641
hsa_circ_0078336	2.973155808	33.88891963	-3.3956	0.000011555	0.0002645
hsa_circ_0000175	21.86937807	98.59348333	-2.166	0.000011659	0.0002655
hsa_circ_0027904	19.88451851	0.671499034	4.8664	0.000012138	0.0002751
hsa_circ_0017092	2.515616513	31.93480461	-3.5075	0.000012425	0.0002803
hsa_circ_0005325	1.93232368	31.93851162	-3.8844	0.000013136	0.0002949
hsa_circ_0005374	12.43162097	58.45870592	-2.2058	0.000013565	0.0003028
hsa_circ_0017521	49.52470443	2.9668383	3.9307	0.00001362	0.0003028
hsa_circ_0001314	14.64452386	98.56634694	-2.7201	0.000014009	0.0003071
hsa_circ_0004720	54.77857454	10.42357771	2.3712	0.000013986	0.0003071
hsa_circ_0006801	1.223277306	28.18294557	-4.5217	0.000013955	0.0003071
hsa_circ_0002103	21.11197786	107.4300075	-2.34	0.000014456	0.000315
novel_circ_0018049	1.223277306	24.2787792	-4.3348	0.000014503	0.000315
novel_circ_0001642	79.76216376	3.021745651	4.6129	0.000014668	0.0003171
hsa_circ_0006856	40.30818434	216.4883602	-2.4111	0.000014769	0.0003178
hsa_circ_0007088	3.830116409	29.83230697	-2.9059	0.000015434	0.0003306
hsa_circ_0000091	13.47518251	77.5922393	-2.474	0.000015638	0.0003334
hsa_circ_0005204	180.3509822	22.10973852	2.9956	0.000016008	0.0003397
hsa_circ_0044177	490.4702298	144.4028958	1.7581	0.000016415	0.0003468
hsa_circ_0002926	139.8206257	35.62531193	1.9627	0.000016709	0.0003468
hsa_circ_0005630	1.223277306	23.85485759	-4.3042	0.000016607	0.0003468
novel_circ_0010988	0.874939251	22.81547194	-4.3567	0.00001652	0.0003468
novel_circ_0013051	3.190131936	38.85650851	-3.469	0.000016714	0.0003468
hsa_circ_0002940	5.88689057	62.66793642	-3.3441	0.000017102	0.0003533
hsa_circ_0017310	8.421788791	110.3686008	-3.6018	0.000017444	0.0003588
hsa_circ_0000914	190.953933	47.80581595	1.9913	0.000017636	0.0003612
hsa_circ_0006345	163.6721966	30.2626865	2.4208	0.000017963	0.0003662
hsa_circ_0003472	790.5756557	225.6901204	1.7986	0.000018129	0.000367
hsa_circ_0003644	6.568317439	44.26243819	-2.7398	0.000018156	0.000367
novel_circ_0001346	23.44099344	0.925692384	4.2949	0.00001853	0.0003729
hsa_circ_0000209	1.166585668	27.72769849	-4.2496	0.000020068	0.0004021
hsa_circ_0001264	7.933900584	44.12062536	-2.4665	0.000021162	0.0004204
novel_circ_0013934	81.18566276	18.99964922	2.0891	0.000021142	0.0004204
hsa_circ_0000607	0	63.6173687	-8.8906	0.000021331	0.000422
novel_circ_0010086	0	64.64077158	-8.8979	0.000021524	0.000424
hsa_circ_0015124	29.60568125	1.342998067	4.4279	0.000022322	0.0004379
hsa_circ_0001436	92.43948129	16.48047243	2.455	0.000022817	0.0004458
novel_circ_0020459	19.04138081	1.00724855	4.2552	0.000022973	0.0004469
hsa_circ_0012883	22.42359877	1.680686865	3.7756	0.000023342	0.0004522
hsa_circ_0008874	25.36495304	1.342998067	4.2314	0.000025321	0.0004885
hsa_circ_0002775	2.223970097	27.13079659	-3.4435	0.000026179	0.000503
novel_circ_0005095	2.315192685	28.43116307	-3.4847	0.000026364	0.0005045
hsa_circ_0028381	45.54660307	2.350246618	4.2201	0.000027982	0.0005333
hsa_circ_0008935	2.315192685	28.33909887	-3.4932	0.00002825	0.0005362
novel_circ_0000610	57.29416477	4.58292141	3.517	0.0000284	0.0005368
novel_circ_0010762	0	56.90447565	-8.7518	0.000032106	0.0006044

novel_circ_0021145	46.22399747	0	8.7368	0.000032709	0.0006133
hsa_circ_0003788	2.389862974	24.23711725	-3.254	0.000033035	0.0006164
novel_circ_0009884	22.12373785	1.342998067	4.0517	0.000033134	0.0006164
hsa_circ_0002490	475.4364726	104.4666063	2.1714	0.000034067	0.0006312
hsa_circ_0008951	0.583292834	20.1985982	-4.6851	0.000034741	0.0006408
hsa_circ_0054886	7.792897803	48.17900574	-2.6037	0.000034996	0.0006408
novel_circ_0004646	45.98616371	0	8.7229	0.000034871	0.0006408
hsa_circ_0005548	46.57912368	0	8.7262	0.000035321	0.0006442
novel_circ_0001335	26.41269661	2.352185898	3.5194	0.000035735	0.0006492
hsa_circ_0004877	1.166585668	22.27849714	-3.9455	0.000036288	0.0006567
hsa_circ_0001501	1.80657014	24.12714454	-3.6676	0.000037549	0.0006769
hsa_circ_0026233	0.815518204	22.15219855	-4.731	0.000038636	0.0006938
hsa_circ_0020028	0	52.9064909	-8.6678	0.00003959	0.0007082
novel_circ_0021440	0	52.52051068	-8.6637	0.000039925	0.0007115
hsa_circ_0000390	0	56.01256268	-8.6973	0.000040398	0.0007118
hsa_circ_0039100	16.76763052	1.134345225	3.7573	0.000040344	0.0007118
novel_circ_0004161	0.583292834	18.45718614	-4.5689	0.00004026	0.0007118
hsa_circ_0001073	40.50848487	194.0598843	-2.2469	0.000041565	0.0007296
hsa_circ_0003571	133.3409429	21.84778805	2.5921	0.000042015	0.0007347
hsa_circ_0000295	28.58831287	2.630119143	3.3126	0.000043324	0.0007548
hsa_circ_0019607	1.166585668	23.01556957	-3.9877	0.000043504	0.0007551
hsa_circ_0000672	90.97997243	8.522133797	3.3443	0.00004442	0.0007681
novel_circ_0018060	2.114768857	23.69853274	-3.4135	0.000044966	0.0007747
hsa_circ_0023919	630.7706262	197.7066269	1.6671	0.000045478	0.0007806
hsa_circ_0059074	73.82268475	10.06117938	2.8403	0.000045721	0.0007819
novel_circ_0020615	1.148607018	29.67388483	-4.5726	0.000045927	0.0007826
hsa_circ_0048965	19.17820155	1.010157472	4.2528	0.000046187	0.0007839
novel_circ_0007268	30.24969819	2.06197689	3.7073	0.000046338	0.0007839
hsa_circ_0030254	0	49.97937527	-8.5949	0.000048769	0.000822
hsa_circ_0008309	0	50.97872773	-8.6045	0.000049377	0.0008293
hsa_circ_0001726	0	49.08614954	-8.5772	0.000050625	0.0008472
hsa_circ_0001153	0.874939251	18.71137949	-4.0773	0.000051014	0.0008507
hsa_circ_0001614	0	48.61102759	-8.5667	0.000052178	0.000858
hsa_circ_0047270	1.223277306	21.19744955	-4.1325	0.00005219	0.000858
novel_circ_0007091	1.148607018	20.89868533	-4.1293	0.00005219	0.000858
novel_circ_0009562	16.77035992	0.671499034	4.6162	0.000051752	0.000858
hsa_circ_0006978	0.815518204	19.12383697	-4.5366	0.000053932	0.0008835
hsa_circ_0008998	26.98634859	4.100213885	2.6428	0.000054146	0.0008839
novel_circ_0015059	23.06228019	2.500113547	3.1101	0.00005597	0.0009105
hsa_circ_0000831	6.702408774	64.5853957	-3.208	0.000059282	0.0009512
hsa_circ_0002457	5.186058701	34.22723496	-2.7236	0.000059055	0.0009512
novel_circ_0006472	16.22029498	0.694269288	4.1559	0.00005872	0.0009512
novel_circ_0006637	43.87960936	2.350246618	4.1566	0.000059174	0.0009512
hsa_circ_0000814	33.21311613	5.549477654	2.5538	0.000060096	0.0009609
hsa_circ_0085440	2.515616513	28.6824475	-3.3465	0.000063882	0.001018
hsa_circ_0003871	0.583292834	19.11737905	-4.6222	0.000067064	0.001065
hsa_circ_0004524	0	45.37545133	-8.4746	0.00006788	0.0010743
hsa_circ_0007554	1.458232085	24.75229144	-3.8102	0.000068273	0.0010769
hsa_circ_0007332	0.583292834	17.22045378	-4.4721	0.000069207	0.001088
novel_circ_0008769	0	44.80827872	-8.4595	0.000070606	0.0011062
hsa_circ_0005689	1.166585668	19.847664	-3.7878	0.000071005	0.0011088
novel_circ_0009955	1.166585668	19.72153697	-3.7773	0.00007328	0.0011405
novel_circ_0002520	21.46301904	1.367707602	3.8224	0.000076862	0.0011923
hsa_circ_0072758	27.67741632	4.62281563	2.59	0.000078338	0.0012111
hsa_circ_0000652	4.413409243	29.65030294	-2.6807	0.000078927	0.0012122
novel_circ_0019508	0	46.64124032	-8.4604	0.00007872	0.0012122
hsa_circ_0068611	31.7978498	1.678747584	4.1914	0.000079699	0.0012201
hsa_circ_0002965	14.49530658	0.462846192	4.501	0.000081779	0.0012437
hsa_circ_0008774	52.92365056	9.981562495	2.3747	0.000081669	0.0012437
novel_circ_0007532	18.79951458	1.932940934	3.1696	0.00008357	0.0012668
hsa_circ_0084084	18.57550371	1.00724855	4.1913	0.000084669	0.0012752
novel_circ_0009871	18.92526812	1.342998067	3.8325	0.000084598	0.0012752
hsa_circ_0002968	20.54939308	83.22471129	-1.9987	0.000085273	0.0012761
hsa_circ_0025148	33.94014115	4.984244322	2.7361	0.000085274	0.0012761
hsa_circ_0005496	15.00392912	0.694269288	4.044	0.000086659	0.0012927

hsa_circ_0005372	1.640677263	18.87740075	-3.3687	0.00008823	0.0013036
novel_circ_0001253	17.85679021	0.673438314	4.6779	0.000088226	0.0013036
novel_circ_0011197	17.33437105	73.29999542	-2.0637	0.000087929	0.0013036
novel_circ_0007060	0	56.95603265	-8.5424	0.000089146	0.001313
hsa_circ_0001445	1.166585668	20.09603951	-3.8084	0.000089965	0.0013209
hsa_circ_0006373	9.083933952	45.8185942	-2.3119	0.000090527	0.001325
novel_circ_0016384	50.32903214	3.716014939	3.6893	0.000091195	0.0013306
hsa_circ_0006687	1.631036409	22.80691673	-3.8206	0.000092294	0.0013424
novel_circ_0005061	1.223277306	18.71428841	-3.964	0.000093695	0.0013586
novel_circ_0003781	11.18906196	51.0292554	-2.1796	0.00010044	0.0014518
novel_circ_0002474	33.31267652	0	8.3258	0.00010161	0.0014642
novel_circ_0019609	15.97557606	0.671499034	4.5222	0.00010722	0.0015403
hsa_circ_0007865	32.73356572	0	8.2947	0.00011083	0.0015874
hsa_circ_0007643	0	38.20052372	-8.2716	0.00011165	0.0015942
hsa_circ_0018992	0	38.4519797	-8.2735	0.000112	0.0015943
hsa_circ_0005227	4.529521928	28.36946824	-2.5993	0.00011296	0.0016024
novel_circ_0021439	0	38.32197411	-8.2688	0.00011326	0.0016024
hsa_circ_0000854	14.01287719	52.47109161	-1.8887	0.00011496	0.0016167
hsa_circ_0084143	4.612406721	39.1583532	-3.0307	0.00011475	0.0016167
hsa_circ_0074371	17.3357974	88.78197866	-2.3273	0.00011568	0.001622
novel_circ_0001647	31.78407947	0	8.259	0.00011926	0.0016672
novel_circ_0010113	44.1201988	6.427690249	2.7537	0.00012146	0.0016928
novel_circ_0010353	9.291269226	40.50522983	-2.1114	0.00012425	0.0017266
hsa_circ_0032969	1.964125222	21.75509731	-3.4679	0.00012553	0.0017392
hsa_circ_0003270	193.196102	55.00739237	1.8068	0.00013484	0.0018626
novel_circ_0020469	15.6826003	0.462846192	4.6206	0.00013568	0.0018687
hsa_circ_0003357	0	36.15179224	-8.1931	0.00013685	0.0018793
hsa_circ_0007099	854.4750214	271.62198	1.6457	0.00013826	0.0018931
hsa_circ_0001654	7.44325669	37.86865277	-2.3267	0.00014061	0.0019196
hsa_circ_0008417	13.80554191	53.06217567	-1.915	0.00014191	0.0019318
hsa_circ_0070040	13.76137011	0.462846192	4.4211	0.00014258	0.0019352
novel_circ_0011198	15.55269101	1.470094742	3.333	0.00014557	0.0019701
hsa_circ_0005616	190.7784518	27.11060569	2.7795	0.00014655	0.0019719
hsa_circ_0030253	28.83578748	122.8021514	-2.0658	0.0001462	0.0019719
novel_circ_0009887	19.77674362	2.037267355	3.2153	0.00014742	0.0019779
hsa_circ_0006887	0.765738012	15.43350121	-4.2614	0.00014976	0.0020036
novel_circ_0018694	0	34.45398141	-8.1435	0.00015164	0.0020229
hsa_circ_0000274	64.11271251	14.10454663	2.1482	0.00015479	0.0020591
novel_circ_0006235	28.74586795	0	8.1415	0.00015679	0.0020797
hsa_circ_0002138	1.631036409	20.9432562	-3.7033	0.00016051	0.002123
hsa_circ_0015335	20.77052497	1.809722821	3.4697	0.00016205	0.0021373
hsa_circ_0001098	0	33.48662708	-8.1056	0.00016558	0.0021716
novel_circ_0018138	7.133631622	35.38452199	-2.3245	0.00016518	0.0021716
novel_circ_0018167	0	34.62308314	-8.1246	0.00016653	0.002178
hsa_circ_0000019	33.61135767	6.197236793	2.4401	0.00016893	0.0021809
hsa_circ_0000023	7.334055451	37.5543743	-2.3505	0.00016728	0.0021809
hsa_circ_0001635	1.690457455	17.59883488	-3.2709	0.00016821	0.0021809
hsa_circ_0003258	6.461845608	32.77540538	-2.2881	0.00016909	0.0021809
hsa_circ_0007383	1.964125222	19.16937748	-3.3116	0.00016871	0.0021809
hsa_circ_0003945	43.71526612	10.42454735	2.0513	0.00016991	0.0021854
hsa_circ_0054853	0.583292834	15.40491311	-4.3159	0.00017051	0.0021871
novel_circ_0014471	192.1834433	22.82353958	3.024	0.00017512	0.0022401
novel_circ_0002316	13.78756326	0.694269288	3.9226	0.00017877	0.0022805
novel_circ_0017886	0	32.03736331	-8.0531	0.00018658	0.0023737
hsa_circ_0001367	1.458232085	21.88413327	-3.6276	0.00018746	0.0023784
novel_circ_0010426	34.84973465	4.305957806	2.9585	0.00019418	0.002457
hsa_circ_0080835	0	32.02702682	-8.0454	0.00019589	0.002472
hsa_circ_0008426	1.989015318	17.99627923	-3.2172	0.0001973	0.0024764
novel_circ_0018542	15.92449281	1.00724855	3.9675	0.00019692	0.0024764
hsa_circ_0003434	26.19847617	0	8.0205	0.00019992	0.0024957
hsa_circ_0057657	1.148607018	16.33351442	-3.7951	0.00019974	0.0024957
hsa_circ_0018998	0	31.05482429	-8.0142	0.00020524	0.0025553
novel_circ_0009865	25.15215895	0	7.9834	0.00020995	0.0026071
hsa_circ_0004860	22.80243531	4.079382912	2.4436	0.00021207	0.0026264
hsa_circ_0004843	47.84657279	8.30184527	2.4735	0.00021364	0.0026389

hsa_circ_0001685	0.874939251	15.49034784	-3.8051	0.00021522	0.0026487
novel_circ_0013810	25.57371466	0	7.996	0.00021557	0.0026487
hsa_circ_0007798	2.098216557	21.99878263	-3.3154	0.00021682	0.0026571
hsa_circ_0001693	7.958790681	46.79547338	-2.5395	0.00022149	0.0027001
hsa_circ_0031607	0	30.3187215	-7.9849	0.0002212	0.0027001
novel_circ_0003231	16.67222589	0.925692384	3.8356	0.00022362	0.0027189
hsa_circ_0087641	0	30.36038345	-7.9824	0.00022597	0.0027404
hsa_circ_0075501	26.45413901	3.044515905	3.0844	0.00022951	0.0027761
novel_circ_0020352	13.94654469	1.00724855	3.8103	0.00023285	0.0028093
hsa_circ_0003028	20.52307664	87.71040977	-2.0809	0.00023474	0.0028248
hsa_circ_0007716	2.038795511	24.27798111	-3.5785	0.00023753	0.002851
novel_circ_0013679	0	29.19956096	-7.9379	0.00024295	0.0029084
novel_circ_0014956	25.71053541	0	7.9782	0.00024356	0.0029084
hsa_circ_0008415	17.20431218	0.462846192	4.7288	0.00025079	0.0029795
hsa_circ_0069819	0	29.00689089	-7.9288	0.00025022	0.0029795
novel_circ_0019354	1.148607018	18.14146951	-3.9096	0.00025257	0.002993
hsa_circ_0009036	0.874939251	14.82078809	-3.7438	0.00025703	0.0030382
hsa_circ_0004270	0.583292834	15.76457408	-4.3304	0.00026455	0.0031113
novel_circ_0010101	46.26398724	3.021745651	3.8591	0.00026414	0.0031113
hsa_circ_0008250	0	29.08667933	-7.9092	0.00027224	0.0031937
hsa_circ_0000862	26.45959783	0	7.9705	0.00027996	0.0032404
hsa_circ_0001936	0	27.75029719	-7.877	0.00027838	0.0032404
hsa_circ_0004001	13.03837755	59.82802324	-2.173	0.00028038	0.0032404
novel_circ_0008880	0	28.12286045	-7.8864	0.00027924	0.0032404
novel_circ_0018673	0.765738012	13.80966098	-4.1	0.00027765	0.0032404
novel_circ_0020274	36.0328989	4.514610648	2.9522	0.0002774	0.0032404
novel_circ_0020176	14.27272206	1.00724855	3.8377	0.00028143	0.0032445
hsa_circ_0008836	117.4537367	29.75606753	1.968	0.00028439	0.0032704
hsa_circ_0002458	0.583292834	15.26941924	-4.3104	0.00029616	0.0033975
novel_circ_0001763	504.1967779	33.04959902	3.8064	0.00029994	0.0034324
hsa_circ_0003978	164.7200453	23.74923196	2.7547	0.00030099	0.003436
hsa_circ_0001603	58.95690571	17.34076297	1.751	0.0003051	0.003469
hsa_circ_0030608	32.01342586	6.891506081	2.2067	0.00030537	0.003469
novel_circ_0019823	0	26.85028398	-7.8333	0.00030847	0.0034958
hsa_circ_0060762	1.458232085	21.20035847	-3.5906	0.0003093	0.0034967
hsa_circ_0003057	23.83763277	0	7.876	0.00031033	0.0034998
hsa_circ_0001414	15.96317952	74.048848	-2.1833	0.00031135	0.0035029
hsa_circ_0078626	21.60677751	0	7.7969	0.00031233	0.0035054
hsa_circ_0001051	0	26.38065031	-7.8132	0.00031924	0.0035744
novel_circ_0003666	1.989015318	17.65374223	-3.183	0.00032224	0.0035994
hsa_circ_0000119	2.91646417	39.81032046	-3.5657	0.00032588	0.0036313
hsa_circ_0007162	0	27.07896971	-7.8229	0.00032842	0.003651
hsa_circ_0000704	97.38792654	24.00569416	2.0074	0.0003316	0.0036721
hsa_circ_0001181	9.692116882	41.85695466	-2.0788	0.000333	0.0036721
hsa_circ_0003234	1.631036409	20.45165036	-3.6594	0.0003332	0.0036721
hsa_circ_0028410	24.95173512	5.211788857	2.2264	0.00033347	0.0036721
hsa_circ_0080285	12.64586769	0.694269288	3.7956	0.00033618	0.0036932
hsa_circ_0005092	0	25.73676973	-7.7815	0.00034397	0.003762
hsa_circ_0050334	4.670524709	31.23310776	-2.7157	0.00034405	0.003762
hsa_circ_0007547	0.874939251	14.58548643	-3.7209	0.00034591	0.0037735
novel_circ_0002425	32.03280458	4.647525165	2.7463	0.00035555	0.0038696
novel_circ_0000608	28.90209369	3.741694114	2.8981	0.00035808	0.0038881
hsa_circ_0005398	0.815518204	13.52042161	-4.0604	0.0003599	0.0038988
hsa_circ_0005046	0.583292834	13.36667612	-4.1183	0.00036186	0.0039019
novel_circ_0005241	16.79940578	1.00724855	4.0323	0.00036181	0.0039019
hsa_circ_0003513	8.886239532	43.41795887	-2.234	0.00036904	0.003959
hsa_circ_0008571	1.166585668	16.05752046	-3.4843	0.0003697	0.003959
hsa_circ_0087220	0	25.15893108	-7.7506	0.00036936	0.003959
hsa_circ_0006607	0.874939251	14.48019037	-3.7123	0.00037148	0.003969
novel_circ_0004757	0.874939251	14.4593594	-3.7062	0.00038354	0.0040885
novel_circ_0021217	13.87603016	0.671499034	4.3229	0.00038532	0.0040981
hsa_circ_0002673	0	24.53023518	-7.7182	0.00038948	0.0041049
hsa_circ_0005593	33.35278957	6.578526819	2.3129	0.00038941	0.0041049
hsa_circ_0025850	0	24.38601454	-7.7185	0.00038819	0.0041049
novel_circ_0004250	0.874939251	15.69512212	-3.8219	0.00038732	0.0041049

hsa_circ_0006473	0.765738012	14.59113272	-4.1701	0.00039068	0.0041083
hsa_circ_0045836	12.73433459	0.462846192	4.3337	0.00039431	0.0041279
novel_circ_0001865	37.32378547	6.03315482	2.5686	0.00039351	0.0041279
hsa_circ_0001402	8.26425999	79.44199531	-3.2123	0.00040216	0.0042006
hsa_circ_0006784	0	24.00375488	-7.6951	0.00040783	0.0042503
hsa_circ_0005501	0.815518204	13.26913718	-4.0275	0.00041698	0.0043361
hsa_circ_0063716	21.40780631	0	7.7249	0.00041843	0.0043415
hsa_circ_0006318	27.42315355	3.092965335	3.0194	0.00042679	0.0044184
novel_circ_0008779	0	23.52492591	-7.6679	0.00042995	0.0044412
hsa_circ_0001079	0	23.23083834	-7.6586	0.00043522	0.0044826
novel_circ_0012550	1.349030846	14.90605126	-3.3404	0.00043587	0.0044826
novel_circ_0003043	20.27560202	0	7.6823	0.00044232	0.0045389
hsa_circ_0061395	0	25.09467043	-7.706	0.00044402	0.0045463
novel_circ_0015779	0.583292834	12.44583194	-4.0146	0.00045299	0.0046281
hsa_circ_0000279	0	23.70305138	-7.6608	0.00045516	0.00464
novel_circ_0006514	18.7524638	0	7.6162	0.00046487	0.0047287
hsa_circ_0023883	33.97063964	6.553817284	2.3537	0.00046656	0.0047356
hsa_circ_0004901	3.903360348	28.95295318	-2.8929	0.0004731	0.0047806
hsa_circ_0035228	0	23.51232059	-7.6484	0.00047406	0.0047806
novel_circ_0019368	1.80657014	16.70624924	-3.1483	0.00047307	0.0047806
hsa_circ_0007710	16.53540515	56.9210985	-1.7705	0.00048484	0.0048682
hsa_circ_0050386	17.20992057	2.942128765	2.5182	0.00048428	0.0048682
novel_circ_0003933	0	22.77169916	-7.6249	0.00048952	0.0049047
hsa_circ_0001487	0	23.02589251	-7.632	0.00049336	0.0049326
novel_circ_0014749	5.846751231	30.43920225	-2.297	0.00049639	0.0049523
hsa_circ_0067864	38.06763566	9.49885497	1.9932	0.00050183	0.0049959
hsa_circ_0026235	1.166585668	21.3715249	-3.8912	0.00050405	0.0050072
novel_circ_0003751	0	21.84036049	-7.5791	0.00051042	0.0050597
novel_circ_0022288	0	21.65059934	-7.5695	0.00052435	0.0051869
hsa_circ_0004961	41.10427126	9.685707198	2.0903	0.000528	0.0052119
novel_circ_0012131	1.964125222	18.73723022	-3.2481	0.00053056	0.0052261
hsa_circ_0005583	0	21.69437212	-7.5638	0.000532	0.0052293
hsa_circ_0000660	0	22.35617475	-7.5845	0.00053708	0.0052681
hsa_circ_0012823	0	21.39640599	-7.5516	0.00054444	0.0053291
hsa_circ_0004314	0	21.56824508	-7.5525	0.00054872	0.0053598
hsa_circ_0000701	0	21.25429618	-7.5406	0.00055475	0.0054073
hsa_circ_0003428	0.815518204	12.76172012	-3.9588	0.00056214	0.005468
hsa_circ_0009033	32.82343269	5.548508014	2.5357	0.00056587	0.0054928
hsa_circ_0031608	0	21.38703914	-7.5471	0.00056863	0.0054967
novel_circ_0019854	1.166585668	15.34903612	-3.4335	0.0005685	0.0054967
novel_circ_0010367	17.70056446	0	7.5258	0.00057567	0.0055533
hsa_circ_0009110	0	20.77335638	-7.5207	0.00057762	0.0055606
hsa_circ_0003755	1.166585668	14.92705379	-3.3807	0.00058012	0.0055732
hsa_circ_0004417	0	20.6651514	-7.5133	0.00058905	0.0056459
novel_circ_0016280	0	20.51722375	-7.5055	0.0005901	0.0056459
novel_circ_0002466	16.86027946	0	7.4843	0.00059285	0.005649
novel_circ_0020460	10.23254097	0.462846192	4.0157	0.00059172	0.005649
novel_circ_0011151	17.18645682	2.81503209	2.6253	0.00059885	0.0056946
novel_circ_0009862	16.63769494	0	7.4706	0.00061817	0.0058663
novel_circ_0010555	48.62492765	14.71161347	1.717	0.00062567	0.0059221
novel_circ_0013925	70.8066602	22.23586556	1.6729	0.00062658	0.0059221
novel_circ_0013244	2.515616513	18.94280259	-2.7649	0.00063092	0.0059511
hsa_circ_0007848	0.583292834	12.08925145	-3.9685	0.0006341	0.005969
novel_circ_0011162	8.127412966	32.9023305	-1.9768	0.00063567	0.0059717
hsa_circ_0005347	1.166585668	14.59033463	-3.3489	0.00064377	0.0060357
novel_circ_0007773	0	19.8428158	-7.4618	0.00064867	0.0060695
novel_circ_0001665	16.67367852	0	7.4495	0.00065379	0.006105
hsa_circ_0008952	2.098216557	16.16766472	-2.8725	0.00066692	0.0062152
hsa_circ_0001788	197.7738808	68.10680129	1.5342	0.00068425	0.0063514
hsa_circ_0035292	0	19.47025254	-7.4341	0.00068367	0.0063514
hsa_circ_0008794	0	19.505127	-7.4367	0.00068904	0.0063832
hsa_circ_0001519	48.46319053	151.7923924	-1.6325	0.00069449	0.0064209
hsa_circ_0007683	15.19328575	59.28457698	-1.9589	0.00069727	0.0064244
hsa_circ_0074944	1.223277306	14.88054364	-3.6096	0.00069762	0.0064244
novel_circ_0017379	0	19.21588763	-7.4214	0.00071342	0.006557

hsa_circ_0045095	16.31700267	2.037267355	2.9284	0.00071526	0.006561
hsa_circ_0000251	0.583292834	11.64335767	-3.9192	0.00072363	0.0066248
novel_circ_0007561	15.55960245	0	7.3789	0.00072589	0.0066324
hsa_circ_0000972	2828.116454	1036.466067	1.4409	0.00073433	0.0066875
hsa_circ_0005918	0	18.98463609	-7.3973	0.00073765	0.0066875
novel_circ_0001733	15.9687879	0	7.3976	0.00073715	0.0066875
novel_circ_0014960	11.78472505	0.671499034	4.1011	0.00073615	0.0066875
hsa_circ_0000356	0	18.87352219	-7.3918	0.00074408	0.0067004
hsa_circ_0002722	14.13863073	50.69206772	-1.8145	0.00074533	0.0067004
hsa_circ_0006783	1.166585668	14.59227392	-3.3498	0.00074625	0.0067004
hsa_circ_0008381	0	18.98252526	-7.4033	0.0007439	0.0067004
novel_circ_0011368	13.23880138	0.925692384	3.5044	0.00074113	0.0067004
hsa_circ_0003525	12.0888913	1.134345225	3.288	0.00074982	0.0067196
hsa_circ_0004870	0.583292834	11.71263807	-3.9439	0.00075145	0.0067213
hsa_circ_0001350	0	18.96186584	-7.3895	0.00075766	0.006758
hsa_circ_0001430	0	18.79972314	-7.3863	0.00076098	0.006758
hsa_circ_0006378	0	19.27387546	-7.4	0.00075992	0.006758
hsa_circ_0079449	1.148607018	17.66875536	-3.8537	0.00076135	0.006758
novel_circ_0013990	0.815518204	11.54194017	-3.8304	0.00077201	0.0068397
novel_circ_0019990	0	18.75013252	-7.3856	0.00077375	0.0068421
hsa_circ_0001322	0.874939251	12.44583194	-3.4987	0.00077656	0.006854
hsa_circ_0091365	0.583292834	11.4555358	-3.9072	0.00077895	0.0068621
hsa_circ_0017854	1.458232085	16.28894355	-3.2151	0.0007862	0.0069129
hsa_circ_0002553	0	18.12143663	-7.3515	0.00079104	0.0069389
hsa_circ_0069608	0	18.16406821	-7.3559	0.00079361	0.0069389
novel_circ_0013850	24.50253362	5.863426557	2.0726	0.00079269	0.0069389
hsa_circ_0005289	14.7371728	0	7.322	0.00080345	0.0070118
novel_circ_0014456	0	18.50757485	-7.3643	0.00081069	0.0070618
hsa_circ_0008621	1.148607018	13.41415591	-3.5154	0.00081407	0.0070648
hsa_circ_0010934	14.86019693	0	7.321	0.00081262	0.0070648
hsa_circ_0077495	0	18.33573576	-7.3592	0.00081886	0.0070801
novel_circ_0000116	0	17.89098317	-7.3347	0.00081747	0.0070801
hsa_circ_0052877	25.94967222	5.110371357	2.3085	0.00082215	0.0070954
novel_circ_0012462	0	18.16018965	-7.3469	0.00082616	0.0071037
novel_circ_0019152	10.39843385	0.671499034	3.9289	0.00082517	0.0071037
novel_circ_0000126	1.223277306	13.24636693	-3.4681	0.00084288	0.0072342
hsa_circ_0054171	1.631036409	15.72274058	-3.2988	0.00084455	0.0072352
hsa_circ_0012144	29.60152549	7.590623571	1.9499	0.00084779	0.0072497
hsa_circ_0007137	0	17.59786524	-7.3172	0.0008533	0.0072835
hsa_circ_0007192	14.8145725	1.346876629	3.468	0.00086719	0.0073886
hsa_circ_0004431	0	18.10819122	-7.335	0.0008754	0.0074178
hsa_circ_0008350	3.221933479	19.52031168	-2.5562	0.00087325	0.0074178
hsa_circ_0029926	20.03112968	3.742663755	2.3716	0.00087407	0.0074178
hsa_circ_0040414	181.8992207	69.28086918	1.3895	0.00087865	0.0074319
novel_circ_0003047	12.92226486	1.00724855	3.6718	0.00088567	0.0074777
novel_circ_0001353	27.79495535	3.484591843	2.9671	0.00088986	0.0074909
novel_circ_0022222	10.18967222	0.671499034	3.8983	0.00089043	0.0074909
novel_circ_0009396	0	17.43201554	-7.294	0.00090103	0.0075664
hsa_circ_0000564	0	17.33979333	-7.292	0.00090326	0.0075715
hsa_circ_0003823	0	17.32671948	-7.2891	0.00090573	0.0075786
hsa_circ_0037010	2.854313715	29.38253463	-3.339	0.00091183	0.0075888
hsa_circ_0077096	0	17.37032071	-7.2935	0.00090879	0.0075888
novel_circ_0016588	14.24783196	0	7.2754	0.0009105	0.0075888
hsa_circ_0000605	0.583292834	12.06066335	-3.9592	0.00091787	0.0076255
hsa_circ_0007242	27.3084935	6.558665486	2.0454	0.00092213	0.0076473
novel_circ_0013684	0	17.21269665	-7.2805	0.00092456	0.0076538
novel_circ_0021666	1.631036409	16.66635502	-3.3724	0.00092816	0.0076701
hsa_circ_0000592	0	17.06767793	-7.2698	0.00093093	0.0076793
novel_circ_0003610	21.61511531	4.307897087	2.2873	0.00093781	0.0077225
novel_circ_0017571	0	17.50290566	-7.2852	0.00094365	0.0077569
hsa_circ_0024960	43.44432776	6.12343775	2.7727	0.00095067	0.0078009
hsa_circ_0000051	0	16.79636061	-7.2586	0.00095429	0.0078168
hsa_circ_0004561	4.206073969	23.00329381	-2.355	0.00095701	0.0078253
hsa_circ_0066971	0	17.08463034	-7.2645	0.00096631	0.0078876
novel_circ_0001652	13.96036759	0	7.2342	0.00097576	0.0079094

novel_circ_0004426	0	16.65231152	-7.2452	0.00097214	0.0079094
novel_circ_0008252	14.04883449	0	7.2519	0.00097305	0.0079094
novel_circ_0014536	0	17.85884607	-7.2992	0.00097532	0.0079094
novel_circ_0013280	0	16.62663235	-7.2404	0.00097954	0.0079263
hsa_circ_0007976	0	16.63051091	-7.2395	0.00098466	0.007954
hsa_circ_0004132	1.640677263	14.06094541	-2.9603	0.00099351	0.0079849
hsa_circ_0008881	9.448824308	37.21781315	-1.9799	0.00099363	0.0079849
novel_circ_0017708	0	16.62469307	-7.2375	0.00099289	0.0079849
hsa_circ_0001317	0	17.0240767	-7.2494	0.0010009	0.0080299
hsa_circ_0002789	6.286311876	26.79149807	-2.0657	0.0010136	0.0081082
novel_circ_0017402	0	17.54295789	-7.2791	0.0010142	0.0081082
hsa_circ_0000502	0	18.87998011	-7.3179	0.0010346	0.0082576
hsa_circ_0003322	0	16.10500024	-7.2078	0.0010408	0.0082925
novel_circ_0022156	0	16.37631756	-7.2131	0.0010445	0.0083076
hsa_circ_0001177	0	16.50227304	-7.2258	0.0010595	0.0083984
novel_circ_0021516	9.958873203	0.673438314	3.8768	0.0010579	0.0083984
hsa_circ_0001360	26.73899727	5.003136015	2.3634	0.0010791	0.0085392
novel_circ_0014791	0	15.85274618	-7.1841	0.0010872	0.0085888
novel_circ_0004890	20.54666367	4.645585884	2.1092	0.0010891	0.0085895
hsa_circ_0008282	57.43079149	12.18388146	2.218	0.0010961	0.0086303
hsa_circ_0072780	0	16.91021188	-7.2353	0.0011095	0.0087065
novel_circ_0001732	13.0121844	0	7.1566	0.0011083	0.0087065
hsa_circ_0006825	13.14757879	0	7.1704	0.0011122	0.008713
novel_circ_0015097	7.385138701	32.51151562	-2.104	0.0011237	0.0087882
hsa_circ_0047705	1.166585668	13.13913158	-3.2055	0.0011266	0.0087963
hsa_circ_0001200	69.95240272	19.52806881	1.8395	0.0011421	0.0089022
novel_circ_0009840	14.30176792	0	7.2315	0.0011447	0.0089073
novel_circ_0020038	0	16.51357916	-7.2018	0.001148	0.0089182
hsa_circ_0000690	15.1173124	49.57370527	-1.7165	0.0011544	0.0089388
novel_circ_0015972	12.85462931	0	7.1406	0.0011544	0.0089388
novel_circ_0016991	17.52776014	1.342998067	3.6617	0.0011584	0.0089546
novel_circ_0022085	14.65140903	0	7.2448	0.0011603	0.0089546
hsa_circ_0023891	9.274716926	46.21280006	-2.2894	0.0011628	0.0089587
novel_circ_0010818	0	17.10288195	-7.2288	0.0011689	0.0089763
novel_circ_0021264	16.52576429	50.03928883	-1.5981	0.0011677	0.0089763
hsa_circ_0069443	0	16.01101031	-7.1694	0.0011769	0.0090233
hsa_circ_0001516	0.815518204	13.15528591	-3.9577	0.0011789	0.0090234
novel_circ_0017847	12.6071547	0	7.1276	0.0011879	0.0090775
hsa_circ_0005946	25.17429336	4.747973024	2.3861	0.0011986	0.009086
novel_circ_0000570	0	15.88779219	-7.1589	0.0011967	0.009086
novel_circ_0002465	12.77031817	0	7.1243	0.0011988	0.009086
novel_circ_0009629	12.67348719	0	7.1291	0.001196	0.009086
novel_circ_0021529	15.24449229	2.016436382	2.9489	0.0011981	0.009086
hsa_circ_0000418	22.70417799	105.9568403	-2.1926	0.0012011	0.0090872
hsa_circ_0002528	1.166585668	15.67074215	-3.4588	0.0012028	0.0090872
hsa_circ_0001246	0	15.15556797	-7.126	0.0012096	0.0090945
hsa_circ_0009144	0	15.25892475	-7.1304	0.0012091	0.0090945
hsa_circ_0044241	0	15.0740118	-7.1257	0.0012073	0.0090945
hsa_circ_0006374	0	15.37003865	-7.1337	0.0012165	0.0091317
novel_circ_0009893	13.02743364	0	7.1309	0.0012326	0.0092375
novel_circ_0006234	13.0508711	0	7.1455	0.0012469	0.0093301
hsa_circ_0001377	44.41454835	10.80971594	2.0017	0.0012684	0.0094453
hsa_circ_0005656	9.150266445	0.671499034	3.7632	0.001268	0.0094453
hsa_circ_0008382	0	14.96483718	-7.1107	0.0012657	0.0094453
hsa_circ_0070680	0	14.736323	-7.0979	0.0012722	0.0094582
hsa_circ_0000849	6.121845348	38.36266642	-2.5611	0.0012767	0.0094768
hsa_circ_0000024	28.97406085	88.04795411	-1.5924	0.001284	0.0095157
hsa_circ_0001405	0	14.63296622	-7.0901	0.0012923	0.0095322
hsa_circ_0007375	0	14.63296622	-7.0901	0.0012923	0.0095322
novel_circ_0001878	47.98754929	11.47636677	2.0307	0.0012923	0.0095322
hsa_circ_0067772	0	14.61116561	-7.0857	0.0012976	0.0095409
novel_circ_0014737	1.148607018	15.40605431	-3.6549	0.0012957	0.0095409
novel_circ_0003507	0	15.56705581	-7.1265	0.0013077	0.0096004
hsa_circ_0003206	0.765738012	10.51095172	-3.7065	0.0013154	0.0096086
hsa_circ_0007542	0	14.50586955	-7.0783	0.0013159	0.0096086

hsa_circ_0029703	4.255854161	26.15795398	-2.5293	0.0013154	0.0096086
novel_circ_0011710	12.89046332	0	7.1201	0.0013171	0.0096086
novel_circ_0012423	0	14.69660034	-7.082	0.0013259	0.009658
hsa_circ_0069227	0	14.5656251	-7.0678	0.0013633	0.0099067
hsa_circ_0079799	0	14.57030175	-7.0794	0.0013643	0.0099067
hsa_circ_0004189	0	14.4642076	-7.0619	0.0013729	0.0099082
hsa_circ_0005653	0.583292834	11.95245837	-3.9464	0.0013675	0.0099082
novel_circ_0013486	38.10216661	11.09701603	1.7677	0.00137	0.0099082
novel_circ_0015468	6.202000733	26.4974105	-2.0973	0.001373	0.0099082
novel_circ_0007941	11.97277862	0	7.0504	0.0013837	0.0099697
hsa_circ_0013252	0	14.16915039	-7.0497	0.0013873	0.0099721
hsa_circ_0020506	0.874939251	11.35217902	-3.381	0.0013883	0.0099721
hsa_circ_0004405	0	14.14638014	-7.0429	0.0014061	0.010054
hsa_circ_0007180	3.937891298	25.90569991	-2.6664	0.0014043	0.010054
novel_circ_0013192	11.64790431	0	7.0266	0.0014061	0.010054
hsa_circ_0026228	0	14.04302335	-7.0366	0.0014181	0.010124
novel_circ_0015098	1.690457455	14.75344697	-2.9951	0.0014257	0.010142
novel_circ_0018444	12.05839282	0	7.0562	0.0014239	0.010142
novel_circ_0019852	0	14.65009019	-7.0596	0.0014271	0.010142
hsa_circ_0001554	0.874939251	11.09992495	-3.3419	0.0014412	0.010226
hsa_circ_0001069	0	13.91786596	-7.0213	0.0014636	0.010319
hsa_circ_0008463	0	15.3280336	-7.1014	0.0014675	0.010319
hsa_circ_0018484	0	13.91495704	-7.0212	0.0014638	0.010319
hsa_circ_0069607	0	13.98132852	-7.0331	0.0014566	0.010319
novel_circ_0004561	12.07637147	0	7.0532	0.0014665	0.010319
novel_circ_0009754	0	15.50844145	-7.0948	0.0014627	0.010319
hsa_circ_0006620	0	13.9188356	-7.0198	0.0014737	0.010334
novel_circ_0013347	11.37423654	0	7.0026	0.0014741	0.010334
hsa_circ_0040813	10.98445609	1.134345225	3.1703	0.001477	0.010339
hsa_circ_0004936	0	14.10471819	-7.0231	0.0014901	0.0104
novel_circ_0016590	11.80961515	0	7.0337	0.0014881	0.0104
novel_circ_0011689	4.138315126	27.42618336	-2.659	0.001495	0.010418
novel_circ_0007111	8.833729932	0.673438314	3.7119	0.0015076	0.010491
hsa_circ_0000033	42.65930804	12.48943317	1.7516	0.0015194	0.010547
hsa_circ_0003770	0	14.0488412	-7.0212	0.0015203	0.010547
hsa_circ_0001580	2.371884324	14.96968538	-2.6998	0.0015352	0.010608
hsa_circ_0002570	0	13.77267568	-7.0116	0.0015359	0.010608
novel_circ_0004925	0	14.64233306	-7.0439	0.0015335	0.010608
hsa_circ_0006681	1.166585668	13.0965	-3.1937	0.0015445	0.010652
novel_circ_0001349	8.984373567	0.671499034	3.7377	0.0015526	0.010692
hsa_circ_0007082	0.765738012	10.4710575	-3.7148	0.0015597	0.010725
novel_circ_0003298	0	13.87135581	-6.9988	0.0015682	0.010767
hsa_circ_0007739	0	13.91010884	-7.0048	0.0015744	0.010794
hsa_circ_0001727	280.5884751	116.3654239	1.2638	0.0016062	0.010964
hsa_circ_0006420	4.410679835	29.06115816	-2.7254	0.0016053	0.010964
hsa_circ_0067896	0	13.34778443	-6.9695	0.0016046	0.010964
novel_circ_0000475	0	14.65944348	-7.0474	0.0016193	0.011037
hsa_circ_0001240	15.80016562	1.388538576	3.2238	0.0016303	0.011096
hsa_circ_0008186	9.360331127	0.673438314	3.7899	0.0016402	0.011147
hsa_circ_0025750	19.54166555	68.05816676	-1.7828	0.0016443	0.011153
novel_circ_0015580	0	13.13816194	-6.9543	0.0016458	0.011153
novel_circ_0004645	10.8158338	0	6.938	0.0016506	0.011169
hsa_circ_0008861	4.470100881	19.80115385	-2.1558	0.0016558	0.011188
hsa_circ_0003922	4.663613263	26.44428442	-2.4257	0.0016685	0.011241
hsa_circ_0006968	94.54901164	29.59489448	1.6629	0.0016672	0.011241
hsa_circ_0027668	0	15.11744147	-7.0598	0.0016762	0.011244
hsa_circ_0064555	0	13.11830061	-6.9463	0.0016756	0.011244
novel_circ_0003779	0	13.44823229	-6.965	0.0016727	0.011244
novel_circ_0004485	0	13.05757542	-6.9489	0.0016802	0.011255
hsa_circ_0000857	0	13.23957944	-6.9534	0.0016865	0.011265
hsa_circ_0005982	0	13.3316301	-6.9679	0.0016841	0.011265
hsa_circ_0001747	21.06217139	80.8024469	-1.9016	0.0016995	0.011287
hsa_circ_0067774	11.60646191	36.56167036	-1.6387	0.0016933	0.011287
novel_circ_0001334	10.73152266	0	6.9215	0.0016971	0.011287
novel_circ_0014264	0.765738012	10.59718454	-3.728	0.0016952	0.011287

hsa_circ_0079380	10.77296506	0	6.9225	0.0017076	0.011293
novel_circ_0016912	11.43223124	0	6.972	0.001703	0.011293
novel_circ_0021980	3.221933479	16.57075536	-2.341	0.0017063	0.011293
hsa_circ_0006127	144.8752962	56.9975095	1.3417	0.0017101	0.011294
hsa_circ_0035999	1.166585668	12.48943317	-3.1415	0.0017197	0.011334
novel_circ_0002428	11.01352822	0	6.9335	0.0017211	0.011334
hsa_circ_0002954	107.756064	14.54027549	2.8197	0.001732	0.011358
novel_circ_0002449	11.37696595	0	6.972	0.001731	0.011358
novel_circ_0019994	0	14.35486142	-7.0124	0.0017318	0.011358
hsa_circ_0000205	2.214329243	15.5538104	-2.7915	0.0017366	0.011372
hsa_circ_0000494	0	13.32113561	-6.9466	0.0017482	0.011428
hsa_circ_0005966	0	13.12217917	-6.9368	0.0017501	0.011428
hsa_circ_0005420	0	12.9484008	-6.9337	0.0017538	0.011433
novel_circ_0016356	10.54489544	0.671499034	3.9315	0.0017558	0.011433
novel_circ_0007942	10.77439141	0	6.9209	0.0017584	0.011434
novel_circ_0018747	0	12.71891698	-6.9173	0.001778	0.011546
hsa_circ_0017995	0	12.84892258	-6.9248	0.0017865	0.011569
novel_circ_0010833	0	12.67628539	-6.9081	0.0017857	0.011569
hsa_circ_0006668	42.83887424	10.23769513	2.0297	0.0017986	0.011631
novel_circ_0008559	10.49799423	0.462846192	4.0368	0.0018339	0.011843
hsa_circ_0037127	10.77712082	0	6.9173	0.0018521	0.011944
hsa_circ_0008164	1.166585668	13.20373534	-3.2052	0.0018631	0.011998
novel_circ_0017753	0	12.4448623	-6.8837	0.0018662	0.012002
hsa_circ_0080849	0	12.54724944	-6.8883	0.0018692	0.012005
hsa_circ_0018273	9.339623069	0.671499034	3.7544	0.0018856	0.012027
hsa_circ_0060521	0	12.36330613	-6.8809	0.001884	0.012027
novel_circ_0013740	0	12.44680158	-6.882	0.0018809	0.012027
novel_circ_0014081	0	13.2076139	-6.9228	0.0018766	0.012027
novel_circ_0016651	0	12.61846912	-6.8985	0.0018849	0.012027
novel_circ_0013287	1.349030846	11.47830605	-2.9712	0.0018996	0.0121
hsa_circ_0001630	1.640677263	13.20373534	-2.8545	0.0019209	0.012136
hsa_circ_0004024	0.874939251	10.6380484	-3.2794	0.0019097	0.012136
hsa_circ_0007455	0	12.23523981	-6.8673	0.001918	0.012136
hsa_circ_0040188	23.58196994	5.757160856	2.037	0.0019205	0.012136
hsa_circ_0060950	19.98265255	5.085661822	1.9697	0.0019192	0.012136
novel_circ_0012705	3.062952047	29.5800529	-3.2015	0.0019121	0.012136
novel_circ_0006921	0	14.07031225	-6.9624	0.0019261	0.012153
hsa_circ_0001649	0	12.36524541	-6.875	0.0019327	0.012178
hsa_circ_0058514	5.360289375	37.01755751	-2.7588	0.0019366	0.012186
hsa_circ_0058346	12.04875196	1.15711548	3.0867	0.001941	0.012189
novel_circ_0010618	10.45094345	0	6.8799	0.0019423	0.012189
hsa_circ_0001432	48.2351472	119.8752264	-1.3024	0.0019628	0.012216
hsa_circ_0003032	0	12.13188303	-6.857	0.0019622	0.012216
hsa_circ_0005789	0	12.12897411	-6.8569	0.0019626	0.012216
hsa_circ_0008638	10.06664809	0	6.8479	0.0019536	0.012216
hsa_circ_0009090	0	12.28078032	-6.8706	0.0019598	0.012216
hsa_circ_0060849	0	12.28078032	-6.8706	0.0019598	0.012216
novel_circ_0001644	10.0003156	0	6.8428	0.001965	0.012216
hsa_circ_0008199	0	12.30064166	-6.8714	0.0019821	0.012307
novel_circ_0020276	10.61268057	0	6.8667	0.0020117	0.012474
hsa_circ_0001470	0	12.97716045	-6.892	0.0020233	0.012529
hsa_circ_0004587	34.13104742	93.02556896	-1.4336	0.0020319	0.012566
hsa_circ_0002607	0	11.92226055	-6.8367	0.0020481	0.0126
hsa_circ_0006717	3.672561327	21.02561045	-2.4432	0.0020407	0.0126
novel_circ_0014238	40.67590111	9.054900471	2.1401	0.0020434	0.0126
novel_circ_0020277	69.91619884	4.700493235	3.75	0.0020483	0.0126
hsa_circ_0072688	0	12.12606519	-6.8456	0.0020563	0.012633
hsa_circ_0004383	1.914345029	17.51049123	-3.1542	0.0020623	0.012653
hsa_circ_0001613	0	11.8757504	-6.824	0.0020787	0.012654
hsa_circ_0003777	11.92299843	1.15711548	3.067	0.0020741	0.012654
hsa_circ_0004322	8.06799192	0.462846192	3.6865	0.0020756	0.012654
hsa_circ_0008042	17.62601747	2.014497101	3.117	0.0020733	0.012654
hsa_circ_0023555	27.09554983	7.102098204	1.9434	0.0020774	0.012654
novel_circ_0007916	0	12.00769528	-6.8341	0.0020785	0.012654
hsa_circ_0002473	0.583292834	9.813773514	-3.6727	0.0020879	0.012677

novel_circ_0013968	0	12.50170893	-6.8558	0.002086	0.012677
hsa_circ_0031657	1.640677263	12.86781427	-2.845	0.002113	0.012796
hsa_circ_0044954	33.80617311	5.648955873	2.5392	0.0021109	0.012796
hsa_circ_0005585	10.87525485	36.30439653	-1.7081	0.0021197	0.012803
hsa_circ_0006871	19.00957926	3.994917824	2.1641	0.0021196	0.012803
novel_circ_0010964	9.725221483	0.694269288	3.4413	0.0021268	0.01283
hsa_circ_0064557	1.223277306	11.09895531	-3.2198	0.0021477	0.012936
novel_circ_0013942	75.89731427	28.56875423	1.4014	0.00215	0.012936
novel_circ_0020721	0	11.68695889	-6.8094	0.0021573	0.012963
hsa_circ_0054086	10.13025118	0	6.8169	0.0021609	0.012968
hsa_circ_0007930	0.583292834	9.312972383	-3.6233	0.0021643	0.012972
hsa_circ_0003054	0	11.75253229	-6.8045	0.0021683	0.012979
novel_circ_0001666	720.7048615	8.099808358	5.7379	0.0021803	0.013034
hsa_circ_0001173	0	11.67097612	-6.8014	0.0021914	0.013084
hsa_circ_0083557	10.19240163	0.694269288	3.5124	0.0022011	0.013125
novel_circ_0011800	9.751537929	0	6.7974	0.0022339	0.013304
hsa_circ_0001478	0	11.53806161	-6.786	0.0022397	0.013321
novel_circ_0010267	0.874939251	11.05923264	-3.3262	0.0022657	0.013459
hsa_circ_0064690	0	11.53709197	-6.7822	0.0022763	0.013505
novel_circ_0012546	0	11.41484349	-6.7714	0.0022823	0.013523
novel_circ_0002521	9.732132929	0	6.7801	0.0022888	0.013545
hsa_circ_0000740	12.34730983	1.932940934	2.5738	0.0022988	0.013582
hsa_circ_0002041	0	11.37397963	-6.7779	0.002301	0.013582
novel_circ_0022167	9.631269486	0	6.7652	0.0023115	0.013627
hsa_circ_0076198	19.46163346	3.277878282	2.5386	0.0023171	0.013642
novel_circ_0022114	9.866224264	1.010157472	3.3046	0.0023213	0.01365
hsa_circ_0001820	0.765738012	11.15386266	-3.7524	0.0023313	0.013692
hsa_circ_0028899	215.7194189	89.40549678	1.2643	0.0023401	0.013726
hsa_circ_0081778	9.241489034	0	6.7379	0.0023474	0.013751
hsa_circ_0006664	17.85133139	3.025624213	2.5906	0.0023713	0.013874
novel_circ_0017946	0	11.63947911	-6.7769	0.002383	0.013925
hsa_circ_0001977	0	11.14449582	-6.7534	0.0023917	0.013944
hsa_circ_0005476	0	11.09798567	-6.7438	0.0023921	0.013944
novel_circ_0013159	0	11.28677718	-6.7498	0.0023952	0.013944
hsa_circ_0000650	71.04164125	11.15225294	2.6064	0.0024122	0.013973
hsa_circ_0006610	1.80657014	12.10814314	-2.7017	0.0024079	0.013973
novel_circ_0002475	9.184797395	0	6.7356	0.0024059	0.013973
novel_circ_0017524	9.175156541	0	6.7275	0.0024111	0.013973
novel_circ_0001382	46.80180521	15.8914992	1.5384	0.0024218	0.013996
novel_circ_0017107	0	11.07812434	-6.7358	0.0024222	0.013996
novel_circ_0017723	0	11.01545986	-6.7392	0.0024323	0.014037
hsa_circ_0075842	10.38734036	0	6.8259	0.0024357	0.01404
hsa_circ_0005115	0	10.99753781	-6.7326	0.0024522	0.014118
hsa_circ_0035197	17.90932609	61.22497807	-1.7571	0.0024627	0.014131
hsa_circ_0035803	11.7391269	1.342998067	3.1382	0.0024667	0.014131
novel_circ_0009051	9.059043856	0	6.7159	0.0024591	0.014131
novel_circ_0010466	9.143354999	0	6.7268	0.0024661	0.014131
hsa_circ_0000042	46.81702817	12.06727928	1.9198	0.0024742	0.014133
hsa_circ_0009118	0.874939251	10.09073712	-3.2122	0.0024791	0.014133
hsa_circ_0060624	33.57955613	10.84670124	1.6331	0.002471	0.014133
novel_circ_0015651	0	11.0401694	-6.739	0.0024784	0.014133
hsa_circ_0012553	0	11.5022175	-6.7537	0.0024848	0.014148
hsa_circ_0006163	0	10.86947149	-6.7183	0.0024886	0.014152
novel_circ_0018334	9.157177891	0	6.7114	0.0024948	0.01417
novel_circ_0005594	0	10.87044113	-6.7165	0.0025074	0.014217
novel_circ_0020239	9.132287795	0	6.7087	0.002509	0.014217
hsa_circ_0003664	0	10.78500641	-6.7147	0.0025205	0.014264
hsa_circ_0003192	0	11.09507675	-6.7299	0.0025338	0.014288
hsa_circ_0007403	14.25616976	2.06197689	2.6449	0.002531	0.014288
novel_circ_0018204	9.202776045	0	6.7292	0.0025319	0.014288
hsa_circ_0003433	0	10.78403677	-6.713	0.0025373	0.01429
novel_circ_0004741	0	10.86559293	-6.7131	0.0025409	0.014293
hsa_circ_0007892	28.16402775	8.074300736	1.7551	0.00255	0.014327
hsa_circ_0015259	1.690457455	17.51615107	-3.2208	0.0025604	0.014368
novel_circ_0005245	8.893150978	0	6.6958	0.0025679	0.014393

novel_circ_0003937	0	10.84476196	-6.7049	0.0025746	0.014408
novel_circ_0013756	0	10.74237482	-6.6989	0.0025778	0.014408
novel_circ_0018366	8.991285013	0	6.6933	0.0025828	0.014408
novel_circ_0020540	0	11.37318155	-6.7348	0.0025802	0.014408
hsa_circ_0057521	3.263375875	19.46540433	-2.5369	0.0025935	0.01445
hsa_circ_0072440	0	10.7443141	-6.6958	0.0026133	0.014509
hsa_circ_0073068	0	10.76708435	-6.7012	0.0026118	0.014509
novel_circ_0001344	8.018211727	0.671499034	3.5759	0.0026107	0.014509
hsa_circ_0003863	4.3125458	23.71371742	-2.4202	0.0026393	0.014582
hsa_circ_0007567	1.223277306	11.14255654	-3.2253	0.0026454	0.014582
hsa_circ_0017311	1.458232085	14.01637454	-3.0047	0.0026371	0.014582
hsa_circ_0031814	0	11.16144823	-6.7323	0.002634	0.014582
novel_circ_0002904	0	10.66178829	-6.6925	0.0026443	0.014582
novel_circ_0019752	0	10.82860763	-6.7123	0.002635	0.014582
hsa_circ_0024872	13.869145	2.141593776	2.6597	0.0026623	0.014632
hsa_circ_0070930	0	10.53372198	-6.6797	0.0026638	0.014632
novel_circ_0002870	9.954691165	0	6.7751	0.0026614	0.014632
novel_circ_0008152	0	10.88545426	-6.7059	0.0026675	0.014635
hsa_circ_0001147	4.188095318	20.99170563	-2.2813	0.0026788	0.014663
novel_circ_0020836	0	11.47072048	-6.732	0.0026777	0.014663
hsa_circ_0079422	0	10.55164403	-6.6826	0.0026866	0.014671
novel_circ_0012759	0	10.5773232	-6.6878	0.0026852	0.014671
hsa_circ_0006993	1.398811038	11.37785819	-2.944	0.0027137	0.014742
hsa_circ_0007349	0	10.50998208	-6.6692	0.0027183	0.014742
hsa_circ_0020822	5.636686549	23.24311411	-2.0081	0.0027128	0.014742
novel_circ_0009577	8.933290317	0	6.677	0.0027185	0.014742
novel_circ_0014967	9.528953413	0	6.739	0.0027107	0.014742
novel_circ_0015850	0	11.68422153	-6.7414	0.0027118	0.014742
hsa_circ_0005868	0	11.53224376	-6.7385	0.002727	0.014748
hsa_circ_0008153	3.669831919	33.46676574	-3.142	0.0027323	0.014748
novel_circ_0004661	0	10.97961576	-6.7026	0.0027257	0.014748
novel_circ_0020480	0	10.50901244	-6.6681	0.0027307	0.014748
hsa_circ_0008934	0	10.68455855	-6.6879	0.0027445	0.01478
novel_circ_0017697	0	10.55746187	-6.6778	0.0027415	0.01478
hsa_circ_0039264	0	10.32312985	-6.6599	0.0027558	0.014823
novel_circ_0016484	0	11.46215173	-6.7435	0.0027655	0.014859
novel_circ_0000527	0	10.42454735	-6.6641	0.0027709	0.01487
hsa_circ_0000117	0	10.34396083	-6.663	0.0027784	0.014894
hsa_circ_0001290	14.79529079	56.23345869	-1.9017	0.0027831	0.014902
hsa_circ_0006146	0	10.34590011	-6.6611	0.0027999	0.014974
hsa_circ_0002179	74.66024035	29.49444662	1.3284	0.0028085	0.014986
novel_circ_0004660	0	10.82005242	-6.68	0.0028074	0.014986
hsa_circ_0040507	8.81990704	0	6.6715	0.0028347	0.015092
novel_circ_0005025	0	10.42357771	-6.6586	0.002833	0.015092
hsa_circ_0085441	0	10.61042994	-6.6644	0.0028474	0.015142
novel_circ_0009053	8.785376089	0	6.662	0.002853	0.015154
hsa_circ_0001675	0	10.4017771	-6.6512	0.0028598	0.015164
hsa_circ_0002301	0	11.02870527	-6.6878	0.0028646	0.015164
hsa_circ_0008058	27.03470244	68.46982615	-1.3323	0.0028634	0.015164
hsa_circ_0004619	13.63000818	1.683595786	3.0258	0.0028796	0.015226
hsa_circ_0008345	0	10.27758935	-6.6376	0.0028859	0.015241
hsa_circ_0067163	16.72057973	2.604439968	2.6238	0.0028912	0.015241
novel_circ_0016211	0	10.53760054	-6.6599	0.0028921	0.015241
hsa_circ_0009140	6.412065415	29.79840215	-2.1543	0.002907	0.015289
hsa_circ_0088095	0	10.08976748	-6.6294	0.0029079	0.015289
hsa_circ_0001776	135.7319675	328.2582052	-1.2692	0.0029129	0.015299
hsa_circ_0001380	103.0440706	254.4633519	-1.2949	0.0029433	0.015441
hsa_circ_0005202	0	10.37997649	-6.6384	0.0029526	0.015472
hsa_circ_0003400	3.828690059	35.29908726	-3.1287	0.0029634	0.015478
hsa_circ_0004995	0	10.17132364	-6.6252	0.0029619	0.015478
novel_circ_0006127	0	9.987380337	-6.6189	0.0029637	0.015478
hsa_circ_0070997	9.30091008	1.010157472	3.2283	0.0030003	0.015652
hsa_circ_0035575	0	9.962670802	-6.6091	0.0030118	0.015694
novel_circ_0013505	8.275327194	0	6.5944	0.0030198	0.015718
hsa_circ_0002922	18.19954615	64.38643926	-1.803	0.00303	0.015732

hsa_circ_0003351	0	10.25772801	-6.6206	0.0030352	0.015732
novel_circ_0017110	7.45980899	0.462846192	3.5728	0.0030327	0.015732
novel_circ_0021693	8.532442661	0	6.6104	0.0030358	0.015732
novel_circ_0020020	20.28263676	5.548508014	1.8519	0.0030447	0.01576
hsa_circ_0046843	0	9.861253303	-6.5999	0.0030533	0.01577
novel_circ_0021665	0	9.861253303	-6.5999	0.0030533	0.01577
hsa_circ_0007477	10.91396784	33.57092061	-1.6258	0.0030743	0.015826
hsa_circ_0017924	3.372577114	17.68329997	-2.3152	0.0030722	0.015826
hsa_circ_0023694	6.293223322	23.38264455	-1.9053	0.0030725	0.015826
hsa_circ_0008500	0.583292834	9.35754325	-3.6383	0.0030801	0.015838
hsa_circ_0005954	51.88827724	144.9942497	-1.4666	0.0031075	0.015943
hsa_circ_0006648	0	10.46444157	-6.6283	0.003107	0.015943
hsa_circ_0007334	370.7042238	156.5192184	1.2381	0.0031107	0.015943
hsa_circ_0087249	0	11.00787429	-6.6641	0.0031156	0.01595
hsa_circ_0035949	39.28936332	12.29887393	1.6789	0.0031259	0.015969
novel_circ_0002442	8.200656905	0	6.5813	0.0031292	0.015969
novel_circ_0021690	1.166585668	12.19631524	-3.1183	0.0031295	0.015969
novel_circ_0003027	0	9.982532135	-6.6045	0.0031333	0.015971
hsa_circ_0004179	4.95383333	20.03160731	-1.9878	0.0031462	0.015986
hsa_circ_0007911	0	9.732217347	-6.5802	0.0031468	0.015986
novel_circ_0014464	0.874939251	10.72428121	-3.3129	0.00315	0.015986
novel_circ_0020284	8.043101824	0	6.5696	0.003144	0.015986
hsa_circ_0004098	0	9.856405101	-6.5914	0.0031561	0.016
hsa_circ_0005199	3.246823575	15.9929167	-2.2826	0.0031622	0.016013
novel_circ_0018427	0.815518204	8.979162147	-3.4801	0.0031673	0.016022
hsa_circ_0076715	8.10252287	0	6.5788	0.0031758	0.016048
novel_circ_0020289	8.349997483	0	6.5869	0.0031794	0.016048
hsa_circ_0003273	2.446554613	19.57651824	-2.995	0.0031912	0.016085
hsa_circ_0007449	0	9.627890926	-6.5705	0.0031936	0.016085
novel_circ_0001355	20.74563487	3.275939001	2.6218	0.003199	0.016095
hsa_circ_0004217	0	9.629830207	-6.5691	0.003213	0.016148
novel_circ_0022121	7.951879235	0	6.5572	0.0032253	0.016192
hsa_circ_0001851	10.24779021	1.010157472	3.3271	0.0032373	0.016212
hsa_circ_0002832	0	9.864162224	-6.5849	0.0032437	0.016212
hsa_circ_0003585	0	9.673431434	-6.5767	0.0032582	0.016212
hsa_circ_0006448	7.926989138	0	6.5534	0.0032545	0.016212
hsa_circ_0006705	0	9.647752259	-6.5714	0.0032544	0.016212
hsa_circ_0079813	0	9.647752259	-6.5714	0.0032544	0.016212
novel_circ_0001310	0	9.947657671	-6.5848	0.0032604	0.016212
novel_circ_0007329	7.685122914	0.671499034	3.5136	0.0032569	0.016212
novel_circ_0014721	0	9.922948136	-6.5794	0.0032555	0.016212
hsa_circ_0005667	0	10.1296617	-6.5914	0.003275	0.016267
hsa_circ_0006965	0	9.709447093	-6.5642	0.0032798	0.016273
hsa_circ_0003164	0	9.501763891	-6.5488	0.0033148	0.016411
hsa_circ_0035654	0	9.631769488	-6.5612	0.003318	0.016411
novel_circ_0009866	13.8442549	1.678747584	3.0353	0.0033112	0.016411
hsa_circ_0004813	0	9.5473044	-6.5603	0.0033222	0.016414
novel_circ_0002242	0	9.567165733	-6.564	0.0033405	0.016487
hsa_circ_0006987	2.456195467	12.8469833	-2.3507	0.0033616	0.016565
novel_circ_0020244	21.42160293	5.400580365	2.0041	0.0033634	0.016565
novel_circ_0014976	49.26345949	11.46781156	2.0964	0.0033751	0.016605
hsa_circ_0000439	0.583292834	9.148092322	-3.5756	0.0033867	0.016645
hsa_circ_0006324	30.43908109	81.69472203	-1.4169	0.0033941	0.016663
hsa_circ_0001268	7.944967789	0	6.5493	0.0034128	0.016703
hsa_circ_0004245	9.526224005	34.09529008	-1.8088	0.0034097	0.016703
hsa_circ_0005643	0	9.632739128	-6.5544	0.0034106	0.016703
hsa_circ_0001146	6.821250867	36.95409495	-2.3625	0.0034307	0.016773
novel_circ_0010913	8.143965267	0	6.5608	0.003439	0.016796
novel_circ_0014660	76.75033942	21.32421666	1.8366	0.0034487	0.016826
hsa_circ_0006677	0	9.68958576	-6.5464	0.0034558	0.016843
novel_circ_0016647	0.874939251	9.651630821	-3.1545	0.0034602	0.016846
novel_circ_0018693	0	9.583320058	-6.5388	0.0034647	0.016851
hsa_circ_0005565	0	9.461869671	-6.547	0.0034776	0.016861
novel_circ_0000694	1.398811038	10.21880343	-2.7931	0.0034703	0.016861
novel_circ_0018100	0	9.462839312	-6.5471	0.0034774	0.016861

hsa_circ_0000776	0	10.37512828	-6.5978	0.0034897	0.01687
hsa_circ_0003622	0	9.336712277	-6.5335	0.0035083	0.01687
hsa_circ_0003730	0	9.422147005	-6.5353	0.0034971	0.01687
hsa_circ_0023918	6.226890829	24.94075338	-1.9896	0.0035069	0.01687
hsa_circ_0025570	0	9.336712277	-6.5335	0.0035083	0.01687
hsa_circ_0046420	7.660232818	0	6.5095	0.0034863	0.01687
novel_circ_0015940	0.765738012	8.99902348	-3.5	0.0035012	0.01687
novel_circ_0022148	7.826125696	0	6.519	0.0035064	0.01687
hsa_circ_0004095	8.521349176	0	6.5945	0.0035133	0.016876
hsa_circ_0003937	8.701064946	0.694269288	3.2843	0.003517	0.016877
hsa_circ_0002505	17.45321314	4.518489209	1.9549	0.0035392	0.016914
hsa_circ_0016102	8.949842617	1.010157472	3.1647	0.0035392	0.016914
hsa_circ_0069285	0	9.289232488	-6.5198	0.003538	0.016914
novel_circ_0014593	1.166585668	11.81211629	-3.0665	0.0035372	0.016914
novel_circ_0016257	0.765738012	9.169892936	-3.5036	0.0035622	0.017006
hsa_circ_0004669	0	9.809894952	-6.5505	0.0035698	0.017025
hsa_circ_0003073	0.583292834	8.282953578	-3.4573	0.0035773	0.017043
hsa_circ_0001313	1.223277306	11.95342801	-3.281	0.003583	0.017053
hsa_circ_0006171	15.72679839	2.708766389	2.5003	0.0035906	0.017072
hsa_circ_0004303	0	9.061687954	-6.4943	0.003631	0.017229
hsa_circ_0067582	0	9.288262848	-6.5132	0.0036307	0.017229
hsa_circ_0025039	7.593900325	0	6.4931	0.0036483	0.017293
hsa_circ_0001368	0	9.475115075	-6.5182	0.0036708	0.017332
hsa_circ_0079481	35.04324703	10.29842032	1.7608	0.0036713	0.017332
hsa_circ_0081028	0	9.062657594	-6.4918	0.0036673	0.017332
hsa_circ_0088281	0	9.370788655	-6.5116	0.0036672	0.017332
novel_circ_0018326	0	9.799730023	-6.5393	0.0036751	0.017332
hsa_circ_0000842	0	8.955422252	-6.4831	0.0036981	0.017406
hsa_circ_0037353	8.391439879	0.694269288	3.2176	0.0036945	0.017406
hsa_circ_0007282	7.625701867	0	6.4827	0.0037023	0.017408
novel_circ_0009187	0	8.977222866	-6.4887	0.003708	0.017417
hsa_circ_0008177	16.91136271	48.96840616	-1.5179	0.0037413	0.017521
hsa_circ_0018274	7.990592223	0	6.5082	0.0037391	0.017521
novel_circ_0013054	10.8323861	48.278345	-2.1234	0.0037396	0.017521
hsa_circ_0000219	22.73752917	71.29375656	-1.635	0.0037753	0.017574
hsa_circ_0002782	11.2899254	37.82761735	-1.7359	0.0037613	0.017574
hsa_circ_0042802	0	8.934591279	-6.4712	0.0037742	0.017574
novel_circ_0007263	12.95549276	2.353155539	2.5102	0.0037621	0.017574
novel_circ_0009700	0	9.380485059	-6.5054	0.0037701	0.017574
novel_circ_0011698	7.47778764	0	6.477	0.0037654	0.017574
hsa_circ_0015779	0	8.852065471	-6.4697	0.0038002	0.017637
hsa_circ_0040357	13.28858157	1.678747584	2.9862	0.0037983	0.017637
hsa_circ_0054322	0	8.932651998	-6.4696	0.0037972	0.017637
hsa_circ_0004894	0	9.379343864	-6.5191	0.0038234	0.01771
hsa_circ_0005649	0	9.331864075	-6.51	0.0038218	0.01771
novel_circ_0005440	0	9.892420759	-6.5352	0.0038348	0.017745
hsa_circ_0029767	0	8.936530559	-6.467	0.0038419	0.017749
novel_circ_0015406	12.43580301	44.84815939	-1.8566	0.0038444	0.017749
novel_circ_0021895	0	8.853035112	-6.4665	0.0038472	0.017749
hsa_circ_0007609	50.40400158	15.79202098	1.6478	0.0038514	0.017752
hsa_circ_0084575	7.501251386	0	6.4645	0.0038555	0.017753
novel_circ_0021401	34.98655539	9.64969154	1.8369	0.0038596	0.017754
hsa_circ_0066187	0	8.959300814	-6.4716	0.0038708	0.017771
novel_circ_0014947	7.410028797	0	6.4571	0.0038674	0.017771
hsa_circ_0001333	0	9.128059435	-6.4936	0.0038789	0.01779
hsa_circ_0005567	6.124574756	70.37367788	-3.3455	0.0039162	0.017904
hsa_circ_0007366	7.51231859	0	6.4765	0.0039132	0.017904
novel_circ_0019364	0	9.390649988	-6.5004	0.003916	0.017904
novel_circ_0020463	7.361674955	0	6.4581	0.0039229	0.017904
novel_circ_0021430	45.15967531	14.7600629	1.5972	0.0039225	0.017904
hsa_circ_0009173	29.5103029	9.14324412	1.699	0.0039302	0.017919
novel_circ_0003930	0	8.725938437	-6.4475	0.0039339	0.017919
hsa_circ_0077084	0	8.911821024	-6.4531	0.0039546	0.017996
hsa_circ_0001680	0	8.960270454	-6.463	0.0040025	0.018071
hsa_circ_0002474	0	9.146951128	-6.4884	0.0040153	0.018071

hsa_circ_0004815	0	8.702198542	-6.4354	0.0040121	0.018071
hsa_circ_0018881	3.556448642	19.21605919	-2.3276	0.0040035	0.018071
hsa_circ_0047720	0	8.743860489	-6.4489	0.0040077	0.018071
hsa_circ_0056838	0	9.123382787	-6.4661	0.0039851	0.018071
hsa_circ_0071175	0	8.641473349	-6.4418	0.0040183	0.018071
novel_circ_0001653	7.227583619	0	6.4366	0.0039801	0.018071
novel_circ_0008620	0	8.766630743	-6.4539	0.0040215	0.018071
novel_circ_0013568	0.815518204	9.380485059	-3.5031	0.0039915	0.018071
novel_circ_0014604	0.583292834	8.304754192	-3.4689	0.0039875	0.018071
novel_circ_0019977	0	9.138395918	-6.4701	0.0040103	0.018071
novel_circ_0020551	0	8.620642376	-6.436	0.0040078	0.018071
hsa_circ_0084547	0	8.704137823	-6.4342	0.0040347	0.018079
novel_circ_0011356	0	8.704137823	-6.4342	0.0040347	0.018079
novel_circ_0013366	0.583292834	8.052500122	-3.416	0.004034	0.018079
novel_circ_0015547	0	8.640503709	-6.4404	0.0040389	0.01808
hsa_circ_0067474	0	8.621612016	-6.4329	0.0040566	0.018142
hsa_circ_0004650	0	9.223830647	-6.4679	0.0040764	0.018179
hsa_circ_0024235	0	8.598841762	-6.4247	0.004075	0.018179
hsa_circ_0029853	0.583292834	8.158765824	-3.4296	0.0040767	0.018179
hsa_circ_0003812	90.9037525	26.74659764	1.7528	0.0040837	0.018194
hsa_circ_0021570	0.583292834	8.784724349	-3.5119	0.0040898	0.018203
novel_circ_0009318	0	8.639534068	-6.4361	0.0041023	0.018241
hsa_circ_0003263	0	8.973344304	-6.461	0.004107	0.018245
novel_circ_0012449	0	8.790370638	-6.4515	0.0041354	0.018354
hsa_circ_0001316	1.989015318	11.64626659	-2.5885	0.0041506	0.018404
hsa_circ_0002876	11.15307838	1.010157472	3.4396	0.0041634	0.01841
hsa_circ_0005308	7.052049888	0	6.402	0.0041705	0.01841
hsa_circ_0006588	1.166585668	10.99171997	-2.9593	0.0041718	0.01841
hsa_circ_0016646	7.270452366	0	6.4348	0.0041715	0.01841
hsa_circ_0025005	7.052049888	0	6.402	0.0041705	0.01841
novel_circ_0002214	0	8.95057405	-6.4499	0.0041975	0.018506
novel_circ_0009060	7.111470934	0	6.4132	0.0042143	0.018563
hsa_circ_0006348	0	8.387279999	-6.4009	0.0042373	0.018632
hsa_circ_0054144	6.985717395	27.11012362	-1.9355	0.0042419	0.018632
novel_circ_0015910	0	8.680397928	-6.4152	0.0042402	0.018632
hsa_circ_0006355	0	8.575101867	-6.4068	0.0042575	0.018665
hsa_circ_0023233	0	8.575101867	-6.4068	0.0042575	0.018665
hsa_circ_0007272	7.027159791	0	6.3946	0.0042653	0.018682
hsa_circ_0084443	0	8.471745087	-6.3984	0.0042777	0.018718
novel_circ_0013763	0	8.574132227	-6.4048	0.0042912	0.01876
hsa_circ_0000702	0	8.472714727	-6.3971	0.0043016	0.018769
novel_circ_0012915	7.360248605	0	6.4261	0.0043031	0.018769
novel_circ_0021006	7.094918634	0	6.4057	0.0043054	0.018769
hsa_circ_0055019	0	8.511467753	-6.4076	0.004346	0.018929
hsa_circ_0013093	0	8.364509745	-6.3853	0.0043779	0.019039
hsa_circ_0036768	19.13260339	5.548508014	1.7653	0.0043796	0.019039
hsa_circ_0001192	0	9.262583672	-6.4585	0.0043966	0.019061
hsa_circ_0005399	10.28923261	31.03011476	-1.5991	0.0043976	0.019061
hsa_circ_0054877	0	8.261152964	-6.3774	0.004392	0.019061
novel_circ_0022124	7.633916371	0	6.4413	0.004401	0.019061
hsa_circ_0070039	11.37281019	37.58957833	-1.7131	0.0044104	0.019084
hsa_circ_0015262	28.6782324	83.96275336	-1.5293	0.0044342	0.01917
hsa_circ_0006012	7.017518937	0	6.3722	0.0044568	0.019178
hsa_circ_0055904	0	8.685246131	-6.404	0.0044505	0.019178
novel_circ_0007896	0	8.281014297	-6.381	0.0044521	0.019178
novel_circ_0017219	0.815518204	8.803616042	-3.4212	0.0044555	0.019178
novel_circ_0021920	0	8.178627157	-6.3741	0.0044549	0.019178
hsa_circ_0002101	0	8.488697498	-6.3939	0.0044703	0.019219
hsa_circ_0001423	156.6614476	66.41544838	1.2357	0.0044759	0.019226
hsa_circ_0000609	0	8.264061885	-6.3723	0.0044853	0.019248
novel_circ_0014385	0	8.155856903	-6.3646	0.0044908	0.019254
hsa_circ_0002692	112.5867762	17.23288755	2.6328	0.0045109	0.019323
hsa_circ_0034414	0	8.392128201	-6.3846	0.0045155	0.019324
hsa_circ_0007967	4.494990978	18.22673269	-2.0304	0.0045238	0.019335
hsa_circ_0071106	0	8.326554805	-6.3886	0.0045303	0.019335

hsa_circ_0087385	2.75475333	14.14734978	-2.3922	0.0045274	0.019335
hsa_circ_0003694	10.28232116	31.97099182	-1.6184	0.0045364	0.019343
hsa_circ_0003275	3.855006505	16.47773506	-2.0483	0.0045717	0.019406
hsa_circ_0004671	2.041524919	18.24676557	-2.933	0.004572	0.019406
hsa_circ_0007402	0	8.449944473	-6.3756	0.0045663	0.019406
novel_circ_0001348	6.942848648	0	6.361	0.0045635	0.019406
novel_circ_0003178	0	8.345618052	-6.3678	0.0045684	0.019406
novel_circ_0014753	0	8.240321991	-6.3596	0.0045773	0.019411
novel_circ_0017295	0	8.448005192	-6.3744	0.0045883	0.01944
novel_circ_0015854	0	8.328494086	-6.3846	0.0045965	0.019457
hsa_circ_0012265	7.358945547	0.462846192	3.5408	0.0046073	0.019458
hsa_circ_0021604	0.765738012	8.896636339	-3.4742	0.0046175	0.019458
hsa_circ_0047870	0	8.074300736	-6.3578	0.0046119	0.019458
hsa_circ_0073371	0	8.342709131	-6.3657	0.0046046	0.019458
hsa_circ_0083045	0	8.413928815	-6.3849	0.0046136	0.019458
novel_circ_0001678	6.835073759	0	6.3483	0.004628	0.019467
novel_circ_0013209	6.835073759	0	6.3483	0.004628	0.019467
hsa_circ_0083619	2.572308152	13.69014987	-2.4127	0.0046389	0.019495
novel_circ_0009320	0.815518204	8.304754192	-3.3715	0.0046445	0.019501
hsa_circ_0056589	0	8.242261272	-6.3557	0.0046546	0.019512
novel_circ_0015732	6.669180882	0	6.3344	0.0046554	0.019512
hsa_circ_0002021	23.19910093	62.6614785	-1.4109	0.0046789	0.019575
novel_circ_0001088	0	8.029729868	-6.3392	0.0046774	0.019575
hsa_circ_0023900	39.82560537	7.80201378	2.2674	0.0046859	0.019587
hsa_circ_0000478	9.143354999	34.40665962	-1.8787	0.0047034	0.0196
hsa_circ_0002860	0	8.028760228	-6.3374	0.0047099	0.0196
hsa_circ_0003362	6.728601928	0	6.3466	0.004698	0.0196
novel_circ_0012987	6.917958552	0	6.3519	0.0046994	0.0196
novel_circ_0016434	0	8.028760228	-6.3374	0.0047099	0.0196
novel_circ_0009602	9.168245095	1.00724855	3.1936	0.0047207	0.019627
hsa_circ_0004802	0.765738012	8.053469763	-3.3406	0.0047372	0.019678
hsa_circ_0039349	0	8.137934851	-6.3434	0.004745	0.019693
hsa_circ_0001922	8.243525652	26.77712502	-1.6607	0.0047643	0.019699
hsa_circ_0002092	0	7.969004675	-6.3407	0.0047825	0.019699
hsa_circ_0006200	10.75223072	1.470094742	2.8137	0.0047608	0.019699
hsa_circ_0012964	0	8.28780178	-6.3623	0.0047801	0.019699
hsa_circ_0079440	0	8.027790587	-6.3338	0.004777	0.019699
hsa_circ_0088058	1.166585668	10.8087463	-2.9457	0.0047767	0.019699
novel_circ_0003415	0	8.152947982	-6.3479	0.0047845	0.019699
novel_circ_0004279	0	7.924433807	-6.3267	0.0047728	0.019699
novel_circ_0005890	6.577958293	0	6.3203	0.0047824	0.019699
novel_circ_0014475	40.00556773	9.114656024	2.119	0.0047943	0.019722
novel_circ_0012700	0	7.927342728	-6.324	0.0048271	0.01984
hsa_circ_0089254	2.572308152	12.42306168	-2.2541	0.0048618	0.019915
novel_circ_0008764	0	7.821077026	-6.3147	0.0048625	0.019915
novel_circ_0010072	0	8.182505719	-6.3506	0.0048578	0.019915
novel_circ_0017271	7.403117351	0.671499034	3.4584	0.0048547	0.019915
hsa_circ_0003548	0	7.94429514	-6.3254	0.00493	0.020033
hsa_circ_0003880	13.26511782	3.047424827	2.1309	0.0049187	0.020033
hsa_circ_0006796	0	7.950112982	-6.3259	0.0049273	0.020033
hsa_circ_0008887	0	8.44412663	-6.3571	0.0049207	0.020033
hsa_circ_0013298	0	8.007929254	-6.319	0.0049182	0.020033
novel_circ_0007969	0	7.94429514	-6.3254	0.00493	0.020033
novel_circ_0017998	0	7.950112982	-6.3259	0.0049273	0.020033
novel_circ_0020287	6.619400689	0	6.3172	0.0049032	0.020033
novel_circ_0021533	6.627738485	0	6.3161	0.0049232	0.020033
hsa_circ_0006609	5.360166082	59.74419823	-3.3472	0.0049792	0.020216
hsa_circ_0002157	0	7.715780965	-6.3008	0.0049847	0.020218
novel_circ_0009616	6.461845608	0	6.3002	0.0049885	0.020218
hsa_circ_0084756	0	7.799276413	-6.2995	0.0049973	0.020236
hsa_circ_0001364	51.74730074	20.8132506	1.3137	0.0050214	0.020294
hsa_circ_0052943	0.583292834	7.902633193	-3.3721	0.0050247	0.020294
novel_circ_0017758	0	8.130177727	-6.3285	0.0050246	0.020294
novel_circ_0002623	0	8.882263287	-6.3851	0.0050779	0.020492
novel_circ_0007824	6.436955511	0	6.2919	0.0051072	0.020592

hsa_circ_0084622	44.50592051	11.69923466	1.9199	0.0051213	0.020613
novel_circ_0002885	0	8.109346754	-6.317	0.0051207	0.020613
novel_circ_0013592	0	7.943325499	-6.3143	0.0051316	0.020637
hsa_circ_0068174	0	7.817198465	-6.2998	0.0051374	0.020642
hsa_circ_0005406	0	7.988866008	-6.3255	0.0051465	0.020644
novel_circ_0009182	0	7.695919632	-6.2846	0.0051448	0.020644
hsa_circ_0007225	0	7.73952086	-6.2981	0.0051681	0.020677
hsa_circ_0080069	9.968514057	1.470094742	2.7101	0.0051625	0.020677
novel_circ_0016592	6.685733182	0	6.3087	0.0051668	0.020677
hsa_circ_0035336	0	7.589653931	-6.2755	0.0051735	0.020681
hsa_circ_0013608	0	8.222399939	-6.323	0.0051782	0.020682
novel_circ_0016286	8.808839835	0.925692384	2.9292	0.0051828	0.020682
novel_circ_0010893	56.03077441	7.177836528	2.8922	0.0051921	0.020702
hsa_circ_0069396	0	7.609515264	-6.2817	0.0052025	0.020726
hsa_circ_0005900	0	8.004050693	-6.3045	0.0052138	0.020753
novel_circ_0020297	6.86817836	0	6.3206	0.0052302	0.0208
novel_circ_0021441	0	8.118073518	-6.3118	0.0052446	0.02084
novel_circ_0020035	0	7.69107143	-6.279	0.0052502	0.020844
novel_circ_0003687	0	8.651809832	-6.3554	0.005267	0.020894
hsa_circ_0001538	1.640677263	10.30132924	-2.5141	0.0053055	0.020941
hsa_circ_0004343	6.521266654	0	6.2971	0.0052926	0.020941
hsa_circ_0008004	0	7.591593211	-6.2688	0.0053079	0.020941
hsa_circ_0010926	6.521266654	0	6.2971	0.0052926	0.020941
hsa_circ_0061510	0	7.777475799	-6.2766	0.0053085	0.020941
hsa_circ_0073608	7.634039664	38.89770193	-2.3145	0.0052904	0.020941
novel_circ_0020645	0	7.586745009	-6.2684	0.0053104	0.020941
novel_circ_0021865	0	7.483388229	-6.2605	0.0053193	0.020959
novel_circ_0000701	0	7.775536518	-6.2751	0.0053381	0.021015
novel_circ_0019109	1.631036409	11.43470483	-2.8438	0.0053434	0.021018
hsa_circ_0000845	0	7.898754632	-6.2889	0.0053721	0.021031
hsa_circ_0001058	0	8.534409561	-6.3374	0.0053829	0.021031
hsa_circ_0002685	6.327754272	0	6.2579	0.0053947	0.021031
hsa_circ_0005519	6.90413566	25.39714165	-1.8181	0.0053942	0.021031
hsa_circ_0005658	0	7.48629715	-6.2568	0.0053954	0.021031
hsa_circ_0008059	0	7.567853317	-6.2579	0.0053594	0.021031
hsa_circ_0010458	0	7.99371421	-6.3118	0.0053995	0.021031
hsa_circ_0047552	0	7.48629715	-6.2568	0.0053954	0.021031
hsa_circ_0078200	0.874939251	8.827355936	-3.0198	0.0054008	0.021031
novel_circ_0008689	0	7.568822957	-6.2564	0.0053941	0.021031
novel_circ_0014401	0	7.568822957	-6.2564	0.0053941	0.021031
novel_circ_0016290	0	7.670240457	-6.2649	0.0053802	0.021031
hsa_circ_0000038	0	7.462557255	-6.247	0.0054224	0.021063
hsa_circ_0000409	0	7.462557255	-6.247	0.0054224	0.021063
hsa_circ_0080826	0	7.462557255	-6.247	0.0054224	0.021063
hsa_circ_0062649	0	8.107407473	-6.3023	0.0054273	0.021064
hsa_circ_0002154	0	7.463526896	-6.2461	0.0054417	0.021102
hsa_circ_0001632	0	7.569792598	-6.2531	0.0054668	0.021176
novel_circ_0000370	0	7.564944396	-6.2527	0.0054696	0.021176
hsa_circ_0005668	0	7.400862422	-6.249	0.0055473	0.021458
hsa_circ_0002605	0	7.356291554	-6.2321	0.0055688	0.021497
hsa_circ_0005221	9.177885949	1.00724855	3.1939	0.0055894	0.021497
hsa_circ_0073239	0	7.356291554	-6.2321	0.0055688	0.021497
novel_circ_0001098	1.148607018	10.76999327	-3.1696	0.0055793	0.021497
novel_circ_0009484	6.145309094	0	6.2338	0.0055821	0.021497
novel_circ_0011616	0	7.676058299	-6.2561	0.0055853	0.021497
novel_circ_0021214	6.145309094	0	6.2338	0.0055821	0.021497
hsa_circ_0001355	0	7.274735387	-6.2297	0.0056328	0.021512
hsa_circ_0004317	0	7.503249562	-6.2511	0.0056486	0.021512
hsa_circ_0005540	0	7.274735387	-6.2297	0.0056328	0.021512
hsa_circ_0024766	15.22508729	2.731536643	2.3737	0.0056408	0.021512
hsa_circ_0032939	0	7.377122527	-6.2367	0.0056417	0.021512
hsa_circ_0035216	0	7.252934773	-6.2211	0.0056391	0.021512
hsa_circ_0073486	0	7.274735387	-6.2297	0.0056328	0.021512
hsa_circ_0083004	0	7.509067404	-6.2516	0.005645	0.021512
novel_circ_0002427	6.692644628	0	6.2779	0.0056355	0.021512

novel_circ_0008484	6.177110637	0	6.2215	0.0056436	0.021512
novel_circ_0009626	6.177110637	0	6.2215	0.0056436	0.021512
novel_circ_0015952	0	7.381970729	-6.2371	0.0056388	0.021512
novel_circ_0010900	6.304290526	0	6.2513	0.0056715	0.021581
novel_circ_0006318	6.085888048	0	6.2119	0.0056832	0.021591
novel_circ_0011372	6.085888048	0	6.2119	0.0056832	0.021591
novel_circ_0002418	8.240796244	0.694269288	3.1878	0.0056945	0.021599
novel_circ_0008169	6.251780926	0	6.2254	0.0056917	0.021599
hsa_circ_0002175	19.65920459	5.990523232	1.7183	0.0057069	0.021628
novel_circ_0014734	0.765738012	7.694949992	-3.2689	0.0057389	0.021732
novel_circ_0006249	0	7.545083063	-6.2317	0.0057631	0.021774
novel_circ_0008565	0	7.546052703	-6.2318	0.0057624	0.021774
novel_circ_0018436	6.244869479	0	6.2323	0.0057641	0.021774
hsa_circ_0008425	0	7.336430221	-6.2143	0.0057719	0.021786
hsa_circ_0004437	0	7.147638712	-6.2057	0.0057999	0.021821
hsa_circ_0008358	0	7.782324001	-6.2552	0.0057961	0.021821
hsa_circ_0080635	5.994665459	0	6.1992	0.0057915	0.021821
hsa_circ_0080674	5.994665459	0	6.1992	0.0057915	0.021821
hsa_circ_0057089	0.815518204	7.716750606	-3.2659	0.0058071	0.02183
hsa_circ_0003919	0	7.23113416	-6.2037	0.0058229	0.021837
hsa_circ_0009030	0	7.438817361	-6.2209	0.0058135	0.021837
hsa_circ_0076800	6.243443129	0	6.2198	0.005821	0.021837
hsa_circ_0004985	3.14868954	17.70703986	-2.3695	0.0058377	0.021847
hsa_circ_0007504	0	7.649409483	-6.2375	0.0058333	0.021847
novel_circ_0003395	0	7.230164519	-6.2027	0.0058443	0.021847
novel_circ_0007064	0	7.2321038	-6.2029	0.005843	0.021847
hsa_circ_0004874	0	7.547991984	-6.2282	0.0058534	0.021852
hsa_circ_0008472	0	7.376152887	-6.2262	0.0058549	0.021852
hsa_circ_0078224	0	7.169439326	-6.2107	0.0058641	0.021869
novel_circ_0002636	0	7.875984377	-6.2595	0.0058688	0.021869
hsa_circ_0001345	0	7.3335213	-6.2092	0.0058884	0.021882
hsa_circ_0005373	0	7.255843695	-6.2095	0.0058907	0.021882
hsa_circ_0020915	6.783867217	0	6.2742	0.0058957	0.021882
novel_circ_0009319	0	7.250025852	-6.209	0.0058943	0.021882
novel_circ_0019569	1.148607018	9.779697136	-3.0568	0.0058788	0.021882
novel_circ_0009987	0	7.125838098	-6.1915	0.0059153	0.021937
hsa_circ_0072351	0.583292834	7.921524886	-3.3852	0.0059328	0.021967
novel_circ_0021839	8.566973611	0.925692384	2.8854	0.0059327	0.021967
hsa_circ_0079812	0	7.124868458	-6.1893	0.0059639	0.022047
novel_circ_0021248	0	7.124868458	-6.1893	0.0059639	0.022047
hsa_circ_0000483	0	7.753735904	-6.2404	0.0059718	0.022059
hsa_circ_0008664	0	7.021511677	-6.1785	0.0060267	0.022174
hsa_circ_0012134	5.962863916	0	6.1973	0.0060208	0.022174
hsa_circ_0054894	0	7.562035474	-6.2282	0.0060103	0.022174
hsa_circ_0057523	0	7.021511677	-6.1785	0.0060267	0.022174
novel_circ_0002856	0	7.457709053	-6.2192	0.0060248	0.022174
hsa_circ_0077083	0	7.43687808	-6.2115	0.0060341	0.022183
hsa_circ_0024234	0	7.022481318	-6.1774	0.0060534	0.022202
novel_circ_0003412	0	7.022481318	-6.1774	0.0060534	0.022202
novel_circ_0003860	0	7.022481318	-6.1774	0.0060534	0.022202
hsa_circ_0031615	0	7.123898817	-6.1848	0.0060653	0.022228
hsa_circ_0068630	11.40603808	2.606379249	2.0836	0.0060725	0.022237
hsa_circ_0001696	17.23766337	71.54696673	-2.0159	0.006086	0.022269
hsa_circ_0007917	0.583292834	7.379061808	-3.2987	0.0060983	0.022297
hsa_circ_0012824	0	7.479509667	-6.2224	0.0061052	0.022305
novel_circ_0012923	14.18568151	3.61653672	1.9553	0.0061111	0.022309
novel_circ_0022044	2.779643426	13.78883	-2.3432	0.0061423	0.022405
hsa_circ_0002931	1.148607018	9.481902558	-2.9984	0.0061545	0.022432
hsa_circ_0005395	0	7.319306255	-6.2165	0.0061724	0.022445
hsa_circ_0006501	19.08009379	3.698092887	2.3762	0.0061687	0.022445
hsa_circ_0025853	0	7.319306255	-6.2165	0.0061724	0.022445
hsa_circ_0006393	0.874939251	8.641473349	-3.0056	0.0061958	0.022501
hsa_circ_0006958	20.43876549	5.907027785	1.7446	0.0061975	0.022501
hsa_circ_0077078	0	6.93898587	-6.1708	0.006214	0.022543
hsa_circ_0035944	110.4333914	37.37494964	1.5536	0.0062213	0.022552

hsa_circ_0008433	0	7.210303186	-6.1768	0.0062628	0.022652
novel_circ_0007749	0	7.210303186	-6.1768	0.0062628	0.022652
novel_circ_0018382	10.21456232	2.039206636	2.2638	0.0062634	0.022652
hsa_circ_0070934	0	7.103067844	-6.1667	0.0062877	0.022722
hsa_circ_0000213	0	6.937046589	-6.1665	0.0063035	0.022727
hsa_circ_0012439	0	6.937046589	-6.1665	0.0063035	0.022727
novel_circ_0010822	0	7.018602756	-6.1666	0.0062943	0.022727
hsa_circ_0015264	1.458232085	11.41484349	-2.7129	0.006325	0.022787
hsa_circ_0083536	5.819131727	0	6.1471	0.0063328	0.022797
hsa_circ_0085124	6.5281781	0.462846192	3.4001	0.0063517	0.022847
hsa_circ_0006007	5.787330185	19.59153137	-1.7138	0.0063802	0.022932
novel_circ_0016564	0	6.812858836	-6.1459	0.0064018	0.022992
novel_circ_0000921	0	6.896354283	-6.1446	0.00641	0.023004
hsa_circ_0000936	8.608416007	0.925692384	2.8927	0.0064446	0.023055
hsa_circ_0008083	0	6.960786484	-6.1682	0.0064301	0.023055
hsa_circ_0031610	0	7.173317887	-6.1833	0.006449	0.023055
novel_circ_0005183	0	7.417986388	-6.188	0.0064354	0.023055
novel_circ_0020306	6.247598887	0	6.2165	0.0064452	0.023055
hsa_circ_0030508	0	6.790088581	-6.1338	0.0064645	0.023093
novel_circ_0001635	5.727909138	0	6.1332	0.0064706	0.023097
hsa_circ_0002688	0	7.417016747	-6.1863	0.0064775	0.023104
hsa_circ_0002207	0	6.897323924	-6.1405	0.0065153	0.023107
hsa_circ_0003617	5.860574123	0	6.1451	0.0064914	0.023107
hsa_circ_0007235	0	6.809949914	-6.1404	0.0065227	0.023107
hsa_circ_0031757	0	6.892475722	-6.14	0.0065189	0.023107
hsa_circ_0083125	6.11768959	0	6.1689	0.0064995	0.023107
hsa_circ_0083458	0	6.789118941	-6.1325	0.0064939	0.023107
novel_circ_0012275	5.837110377	0	6.1538	0.006497	0.023107
novel_circ_0019761	0	7.213040553	-6.1927	0.0065073	0.023107
novel_circ_0019870	0	6.897323924	-6.1405	0.0065153	0.023107
hsa_circ_0047378	0	7.025390239	-6.157	0.006533	0.023125
hsa_circ_0001578	10.31685211	29.6396369	-1.4921	0.0065757	0.023178
hsa_circ_0004212	51.34372368	16.70140103	1.6127	0.0065778	0.023178
hsa_circ_0061722	0	6.961756124	-6.1625	0.0065556	0.023178
novel_circ_0000371	0	6.792027862	-6.1292	0.0065811	0.023178
novel_circ_0006989	5.86891192	0	6.1422	0.0065627	0.023178
novel_circ_0010369	5.6712175	0	6.1364	0.0065825	0.023178
novel_circ_0011221	7.951879235	1.00724855	3.0047	0.0065772	0.023178
novel_circ_0009524	0	6.685762161	-6.1191	0.0066191	0.023289
novel_circ_0018567	5.586906357	0	6.1139	0.0066449	0.023345
novel_circ_0020462	5.586906357	0	6.1139	0.0066449	0.023345
hsa_circ_0005587	11.55395231	2.017406022	2.5389	0.00667	0.023415
hsa_circ_0081006	0	6.995832502	-6.1425	0.0066759	0.023418
hsa_circ_0025619	0	6.898293564	-6.1337	0.0066882	0.023442
novel_circ_0005365	0.583292834	8.004050693	-3.3865	0.0066928	0.023442
hsa_circ_0002663	0	7.214979834	-6.1838	0.0067009	0.023443
hsa_circ_0041695	5.653238849	0	6.1177	0.006703	0.023443
novel_circ_0003862	2.371884324	12.86975355	-2.478	0.0067222	0.02349
novel_circ_0007454	5.636686549	0	6.1146	0.0067265	0.02349
hsa_circ_0000085	0	6.78717966	-6.1222	0.0067458	0.023504
hsa_circ_0013171	0	6.808980274	-6.1308	0.0067439	0.023504
hsa_circ_0066241	0	6.78717966	-6.1222	0.0067458	0.023504
hsa_circ_0027851	1.148607018	8.932651998	-2.926	0.0067844	0.023621
novel_circ_0005703	8.434308626	29.46488888	-1.7827	0.0067964	0.023646
hsa_circ_0086190	0	6.768287968	-6.1106	0.0068069	0.023647
novel_circ_0009760	0	6.768287968	-6.1106	0.0068069	0.023647
hsa_circ_0004496	0	6.874553669	-6.1187	0.0068416	0.023705
hsa_circ_0031449	0	6.962725765	-6.1496	0.0068435	0.023705
hsa_circ_0046909	0	6.871644748	-6.1184	0.0068438	0.023705
novel_circ_0016750	0	6.871644748	-6.1184	0.0068438	0.023705
hsa_circ_0066970	0	6.662991906	-6.0992	0.0068706	0.02378
hsa_circ_0073396	0.583292834	7.273765747	-3.2826	0.0069052	0.023864
novel_circ_0010661	5.777689331	0	6.1203	0.006902	0.023864
hsa_circ_0015263	0.815518204	7.461587615	-3.205	0.0069273	0.02387
hsa_circ_0086296	12.73160518	2.354125179	2.4721	0.0069324	0.02387

novel_circ_0000673	5.520573864	0	6.0964	0.0069214	0.02387
novel_circ_0005616	0	6.662022266	-6.0971	0.0069248	0.02387
novel_circ_0021292	0	6.662022266	-6.0971	0.0069248	0.02387
novel_circ_0008784	0	7.519403887	-6.1773	0.0069401	0.023879
hsa_circ_0000357	22.01311026	4.776561121	2.1465	0.0069508	0.023885
hsa_circ_0015211	0	6.558665486	-6.0869	0.0069623	0.023885
hsa_circ_0035094	0	6.558665486	-6.0869	0.0069623	0.023885
hsa_circ_0082583	0	7.082236871	-6.133	0.0069623	0.023885
hsa_circ_0013012	0	6.557695845	-6.0857	0.0069933	0.023956
hsa_circ_0085611	0	6.557695845	-6.0857	0.0069933	0.023956
hsa_circ_0006056	0	6.975001529	-6.1214	0.0070159	0.024015
hsa_circ_0000087	2.797622076	13.39235529	-2.2156	0.0070394	0.024078
novel_circ_0000577	5.719571342	0	6.11	0.0070561	0.024118
novel_circ_0002883	0	6.87649295	-6.1108	0.0070639	0.024127
hsa_circ_0055945	0	6.688671082	-6.1009	0.0070754	0.02414
hsa_circ_0089902	0	6.560604766	-6.0824	0.0070851	0.02414
novel_circ_0013220	0	6.556726205	-6.082	0.0070883	0.02414
novel_circ_0017178	0	6.560604766	-6.0824	0.0070851	0.02414
hsa_circ_0018478	0	6.454339065	-6.0726	0.0071106	0.024181
novel_circ_0009886	5.555104814	0	6.1033	0.0071159	0.024181
novel_circ_0021296	0	6.453369424	-6.0725	0.0071113	0.024181
hsa_circ_0002063	5.54546396	0	6.0889	0.0071584	0.024307
novel_circ_0016792	5.618707899	0	6.0828	0.0071721	0.024336
hsa_circ_0011536	0	6.455308705	-6.0699	0.0071813	0.02434
novel_circ_0003736	0	6.452399784	-6.0696	0.0071837	0.02434
hsa_circ_0065052	5.404461179	0	6.0744	0.0072132	0.024387
novel_circ_0003031	0	6.496970652	-6.0867	0.0072101	0.024387
novel_circ_0020275	5.404461179	0	6.0744	0.0072132	0.024387
hsa_circ_0024843	0	6.731302669	-6.1097	0.0072341	0.02444
hsa_circ_0008038	0	6.561574407	-6.0762	0.0072537	0.024471
novel_circ_0009183	0	6.710471696	-6.1019	0.0072503	0.024471
hsa_circ_0001997	3.520491342	17.82267241	-2.3393	0.0072644	0.024489
hsa_circ_0008272	29.92212075	10.15146231	1.5646	0.0072727	0.0245
hsa_circ_0002059	0	6.456278345	-6.0643	0.0073331	0.024523
hsa_circ_0004456	0	6.456278345	-6.0643	0.0073331	0.024523
hsa_circ_0006323	10.76605361	31.6182899	-1.5243	0.0073005	0.024523
hsa_circ_0030763	0	6.456278345	-6.0643	0.0073331	0.024523
hsa_circ_0054391	0	6.348073363	-6.0555	0.007328	0.024523
novel_circ_0000242	0	6.456278345	-6.0643	0.0073331	0.024523
novel_circ_0003273	16.87264972	3.763494728	2.0944	0.0072863	0.024523
novel_circ_0011966	5.734820584	0	6.0892	0.0073222	0.024523
novel_circ_0017330	0	6.348073363	-6.0555	0.007328	0.024523
novel_circ_0021773	0	6.451430143	-6.0638	0.0073374	0.024523
novel_circ_0021860	0	6.348073363	-6.0555	0.007328	0.024523
hsa_circ_0006456	1.223277306	10.89708995	-3.144	0.0073995	0.024703
hsa_circ_0007178	8.500641118	25.22450447	-1.5529	0.0074043	0.024703
novel_circ_0001877	9.530406043	1.344937348	2.8226	0.0074073	0.024703
novel_circ_0019205	0	6.536864872	-6.0602	0.0074226	0.024737
hsa_circ_0007559	0	6.605175634	-6.086	0.007434	0.024757
hsa_circ_0007418	0	6.350982284	-6.0508	0.0074565	0.024806
hsa_circ_0044708	0	6.43156881	-6.0494	0.0074594	0.024806
hsa_circ_0006554	12.37768502	42.42492536	-1.7749	0.0074794	0.024854
hsa_circ_0008809	5.354680986	0	6.0594	0.0074845	0.024854
novel_circ_0002437	5.693378188	0	6.0787	0.0074922	0.024862
hsa_circ_0005645	11.40603808	1.678747584	2.7684	0.0075008	0.02487
novel_circ_0019803	0	6.640221652	-6.067	0.0075053	0.02487
hsa_circ_0005328	2.023546268	12.30452022	-2.514	0.0075412	0.024912
hsa_circ_0009172	2.857043123	15.48743892	-2.3106	0.0075344	0.024912
hsa_circ_0016601	0	6.32724239	-6.0375	0.007533	0.024912
novel_circ_0001581	5.452815021	0	6.0537	0.0075447	0.024912
novel_circ_0011104	0	6.32724239	-6.0375	0.007533	0.024912
hsa_circ_0071176	0	6.368904336	-6.0559	0.0075537	0.024925
novel_circ_0003021	0	6.746487354	-6.0749	0.0075656	0.024929
novel_circ_0004900	0	6.746487354	-6.0749	0.0075656	0.024929
hsa_circ_0003209	0	6.576587538	-6.0719	0.0075825	0.024949

novel_circ_0018392	0	6.392644231	-6.0632	0.0075789	0.024949
hsa_circ_0006565	0	6.450460503	-6.0541	0.0075971	0.024979
hsa_circ_0000371	0	6.222915969	-6.0235	0.0076709	0.025014
hsa_circ_0003501	1.982103872	10.76417543	-2.3303	0.0076522	0.025014
hsa_circ_0003715	6.163287745	22.93772041	-1.8285	0.0076393	0.025014
hsa_circ_0014156	5.204037351	0	6.0264	0.0076337	0.025014
hsa_circ_0047869	0	6.245686223	-6.0355	0.0076298	0.025014
hsa_circ_0087828	0	6.221946328	-6.0234	0.0076718	0.025014
novel_circ_0000571	0	6.222915969	-6.0235	0.0076709	0.025014
novel_circ_0010075	8.826818486	30.79076298	-1.7686	0.0076344	0.025014
novel_circ_0010554	5.204037351	0	6.0264	0.0076337	0.025014
novel_circ_0016252	0	6.265547556	-6.0431	0.0076654	0.025014
novel_circ_0018033	0	6.539773793	-6.0526	0.0076553	0.025014
novel_circ_0019110	0	6.325303109	-6.0328	0.0076665	0.025014
hsa_circ_0008938	1.982103872	11.24591332	-2.4003	0.0076831	0.025023
novel_circ_0018852	2.505975659	14.66977997	-2.5042	0.0076853	0.025023
hsa_circ_0006359	23.75477426	6.332090592	1.8957	0.0077094	0.025083
hsa_circ_0062804	11.61337336	1.683595786	2.7846	0.0077147	0.025083
novel_circ_0007750	0	6.220976688	-6.0207	0.0077482	0.02514
novel_circ_0015698	0	6.223885609	-6.021	0.0077454	0.02514
novel_circ_0022009	5.179147255	0	6.0201	0.0077471	0.02514
hsa_circ_0031419	1.398811038	9.392589268	-2.6584	0.0078043	0.025269
hsa_circ_0042986	0	6.139420521	-6.0202	0.0078026	0.025269
novel_circ_0021146	5.369930229	0	6.0358	0.0077937	0.025269
hsa_circ_0001906	3.613140281	16.21029631	-2.144	0.0078454	0.025369
hsa_circ_0006267	0	6.140390162	-6.018	0.0078623	0.025369
hsa_circ_0008637	0	6.241807661	-6.0267	0.0078565	0.025369
hsa_circ_0068090	11.55668172	2.354125179	2.3446	0.007848	0.025369
novel_circ_0012898	0	6.540743433	-6.0459	0.0078586	0.025369
hsa_circ_0040364	8.036190378	1.00724855	3.0155	0.0078892	0.025421
hsa_circ_0086466	0	6.116650267	-6.0068	0.0078846	0.025421
hsa_circ_0001020	18.35722453	50.57466745	-1.454	0.0078984	0.025433
novel_circ_0010827	0	6.220007048	-6.015	0.0079103	0.025454
novel_circ_0003985	5.253817543	0	6.0193	0.0079442	0.025545
novel_circ_0002306	23.98281759	3.021745651	2.9316	0.0079542	0.02556
hsa_circ_0003576	0	6.119559188	-6.0023	0.0080197	0.025752
hsa_circ_0002165	0	6.854692336	-6.0695	0.0080313	0.025772
hsa_circ_0027477	0	6.28734817	-6.0353	0.008065	0.025862
novel_circ_0019762	0	6.013293487	-5.9909	0.0080826	0.025901
novel_circ_0016541	17.73521871	4.389453253	2.0215	0.0080998	0.025938
hsa_circ_0001807	0	6.323363828	-6.0167	0.0081335	0.026
novel_circ_0003474	0	6.323363828	-6.0167	0.0081335	0.026
novel_circ_0011617	0	6.162190775	-6.0164	0.0081357	0.026
hsa_circ_0002186	0	6.0350941	-5.9977	0.008169	0.026074
hsa_circ_0020252	0	6.03412446	-5.9976	0.00817	0.026074
hsa_circ_0004058	59.64251462	148.2771342	-1.309	0.0081778	0.026075
novel_circ_0002878	0	6.304472135	-6.0056	0.0081817	0.026075
hsa_circ_0079375	5.119726208	0	5.983	0.0081987	0.026094
novel_circ_0007253	5.119726208	0	5.983	0.0081987	0.026094
hsa_circ_0031641	0	6.199176074	-5.9946	0.0082099	0.026112
novel_circ_0002575	5.197125905	0	6.0158	0.0082318	0.026128
novel_circ_0004484	0	6.094849653	-5.9842	0.0082289	0.026128
novel_circ_0007939	5.197125905	0	6.0158	0.0082318	0.026128
hsa_circ_0070190	27.96357764	80.49025218	-1.5048	0.0082418	0.026142
hsa_circ_0070268	0	6.307381057	-6.0035	0.0082608	0.026159
hsa_circ_0084941	0	6.240838021	-6.0121	0.0082529	0.026159
novel_circ_0015060	5.172235808	0	6.0125	0.0082639	0.026159
hsa_circ_0006893	0	6.114710986	-5.9928	0.008281	0.02616
novel_circ_0013647	0	6.114710986	-5.9928	0.008281	0.02616
novel_circ_0013931	6.794934421	0.673438314	3.3228	0.0082775	0.02616
hsa_circ_0000712	0	5.991492873	-5.9718	0.008315	0.026182
hsa_circ_0006693	2.041524919	14.35988118	-2.5989	0.0082967	0.026182
hsa_circ_0042098	0	6.408798556	-6.0117	0.0083134	0.026182
hsa_circ_0067880	0	5.990523232	-5.9717	0.008316	0.026182
novel_circ_0012529	0.874939251	9.390649988	-3.108	0.0083037	0.026182

novel_circ_0013903	5.028503619	0	5.9695	0.0083288	0.026187
novel_circ_0014298	5.327061482	0	6.0008	0.0083253	0.026187
hsa_circ_0000786	1.749878502	13.33066046	-2.7126	0.0083504	0.026237
hsa_circ_0002871	25.19906016	4.895900673	2.292	0.0083735	0.026287
hsa_circ_0026297	16.77178627	4.159969438	2.0383	0.0083854	0.026287
hsa_circ_0049303	13.19735898	3.300648536	1.9314	0.0083946	0.026287
novel_circ_0000114	0	5.992462513	-5.9694	0.0083938	0.026287
novel_circ_0019916	0	5.992462513	-5.9694	0.0083938	0.026287
hsa_circ_0082688	0	6.308350697	-5.9992	0.0084023	0.026294
novel_circ_0017143	0	6.010384565	-5.9786	0.0084277	0.026338
novel_circ_0021863	0	5.907027785	-5.9697	0.0084241	0.026338
novel_circ_0003543	0	6.1365116	-5.9964	0.0084402	0.026347
novel_circ_0008321	5.053393715	0	5.9679	0.0084476	0.026347
novel_circ_0011583	0	6.845965573	-6.0551	0.0084432	0.026347
hsa_circ_0002682	1.914345029	12.9484008	-2.7292	0.0084644	0.026376
hsa_circ_0045220	0	5.886196812	-5.9571	0.0084737	0.026376
hsa_circ_0067480	1.349030846	9.355775524	-2.6495	0.0084735	0.026376
hsa_circ_0001495	112.5219145	42.68865711	1.3949	0.0084881	0.026403
hsa_circ_0002083	0	6.514094618	-6.0158	0.0085153	0.02647
novel_circ_0011213	4.978723426	0	5.9578	0.0085487	0.026538
novel_circ_0014963	4.978723426	0	5.9578	0.0085487	0.026538
hsa_circ_0001771	0.874939251	8.802646401	-3.0103	0.0085634	0.026567
hsa_circ_0000698	111.0209188	296.7400913	-1.41	0.0086312	0.02667
hsa_circ_0003288	0	6.22679453	-5.9917	0.0086152	0.02667
hsa_circ_0069226	0	6.22679453	-5.9917	0.0086152	0.02667
hsa_circ_0084904	0	5.906058144	-5.9624	0.0086309	0.02667
novel_circ_0009670	0	5.909936706	-5.9629	0.0086266	0.02667
novel_circ_0013358	4.93728103	0	5.9509	0.0086171	0.02667
hsa_circ_0002029	0	5.78090075	-5.9398	0.0087142	0.026732
hsa_circ_0002275	0	6.512155337	-6.0099	0.0087002	0.026732
hsa_circ_0002601	0	5.781870391	-5.94	0.0087132	0.026732
hsa_circ_0002793	0	5.888136092	-5.9511	0.0086635	0.026732
hsa_circ_0032135	0	5.781870391	-5.94	0.0087132	0.026732
hsa_circ_0083234	0.815518204	7.022481318	-3.1259	0.0087029	0.026732
novel_circ_0002759	0	5.884257531	-5.9507	0.0086679	0.026732
novel_circ_0003679	0	6.037033381	-5.9795	0.0086721	0.026732
novel_circ_0012468	0	5.78090075	-5.9398	0.0087142	0.026732
novel_circ_0019985	0	5.781870391	-5.94	0.0087132	0.026732
novel_circ_0021739	0	5.781870391	-5.94	0.0087132	0.026732
hsa_circ_0084464	8.619483211	27.80617418	-1.6353	0.0087347	0.026762
novel_circ_0020802	0	6.183991389	-6.0014	0.0087353	0.026762
novel_circ_0012267	0	6.091940732	-5.965	0.0088319	0.02704
hsa_circ_0063397	19.39663031	6.117619907	1.6339	0.0088539	0.02709
hsa_circ_0009061	2.098216557	11.09604639	-2.3408	0.0089608	0.027399
hsa_circ_0004616	9.515156801	1.342998067	2.8228	0.0089871	0.027461
novel_circ_0000526	0	5.865365838	-5.9292	0.0090041	0.027495
hsa_circ_0000111	23.62629131	64.3121391	-1.4377	0.0090127	0.027504
novel_circ_0000823	4.862610741	0	5.9305	0.009037	0.02756
hsa_circ_0077409	59.21259483	24.9722504	1.245	0.0090457	0.027568
hsa_circ_0002051	0	5.759100136	-5.917	0.009064	0.027588
hsa_circ_0011075	0	5.759100136	-5.917	0.009064	0.027588
hsa_circ_0007549	0	5.778961469	-5.9279	0.0090775	0.027611
novel_circ_0014575	4.413409243	20.68341656	-2.1511	0.009093	0.02764
hsa_circ_0007302	7.47778764	23.35308682	-1.6062	0.009104	0.027656
novel_circ_0019500	0	5.862456917	-5.9255	0.0091206	0.027688
hsa_circ_0062794	7.958790681	0.925692384	2.7793	0.0091337	0.02771
novel_circ_0008884	0	5.966783338	-5.9345	0.0091729	0.027811
hsa_circ_0009133	24.74712926	63.23464051	-1.3315	0.0091903	0.027845
hsa_circ_0091291	7.368586401	1.00724855	2.8929	0.0092137	0.027898
hsa_circ_0013148	0	5.699344584	-5.9231	0.0092512	0.02795
hsa_circ_0030154	0	5.653804075	-5.9013	0.0092548	0.02795
hsa_circ_0090160	0.874939251	8.448974833	-2.9447	0.0092394	0.02795
novel_circ_0015849	0	5.655743356	-5.9015	0.0092524	0.02795
novel_circ_0012142	0	5.67851361	-5.9124	0.0092725	0.027985
novel_circ_0017475	0	5.826441259	-5.9374	0.0093582	0.028226

novel_circ_0013991	0	5.572247909	-5.8996	0.009375	0.028244
novel_circ_0021266	0	5.571278268	-5.8995	0.0093762	0.028244
hsa_circ_0001911	0	5.965813697	-5.9281	0.009394	0.028279
hsa_circ_0005236	0	5.549477654	-5.8859	0.0094379	0.028301
hsa_circ_0008040	0	5.550447295	-5.886	0.0094366	0.028301
novel_circ_0011140	0	5.549477654	-5.8859	0.0094379	0.028301
novel_circ_0014158	0	5.550447295	-5.886	0.0094366	0.028301
novel_circ_0014272	0	5.549477654	-5.8859	0.0094379	0.028301
novel_circ_0021094	7.716924456	1.00724855	2.9342	0.0094295	0.028301
novel_circ_0009565	22.2383009	3.738785193	2.4982	0.0094464	0.028309
hsa_circ_0004888	0.874939251	7.949143342	-2.8842	0.0094538	0.028313
hsa_circ_0001496	0	5.570308628	-5.8937	0.0095502	0.028492
hsa_circ_0086694	4.803189695	0	5.8929	0.0095345	0.028492
novel_circ_0008771	0	6.077897242	-5.9351	0.0095494	0.028492
novel_circ_0014410	4.711967106	0	5.883	0.0095293	0.028492
novel_circ_0017951	2.273750289	11.7694847	-2.2516	0.0095435	0.028492
novel_circ_0018544	4.944192476	0	5.9084	0.0095334	0.028492
hsa_circ_0012440	0	5.700314224	-5.9121	0.0095798	0.028562
hsa_circ_0076401	4.761747298	0	5.8854	0.0096175	0.028638
novel_circ_0019805	4.761747298	0	5.8854	0.0096175	0.028638
hsa_circ_0000836	6.468757054	20.87300616	-1.6692	0.0096385	0.028665
novel_circ_0015874	0	5.868274759	-5.9112	0.009639	0.028665
hsa_circ_0000106	6.700982424	22.34891874	-1.7355	0.0096672	0.028731
hsa_circ_0004327	0	5.651864794	-5.888	0.0096998	0.028758
hsa_circ_0005265	7.428007447	22.6389562	-1.562	0.0096866	0.028758
hsa_circ_0022601	0.583292834	7.002619985	-3.2029	0.009707	0.028758
hsa_circ_0065244	0	5.594048522	-5.8986	0.0097056	0.028758
novel_circ_0013554	4.620744517	0	5.8686	0.0096915	0.028758
hsa_circ_0005704	4.388519147	15.62035344	-1.7703	0.0097267	0.028798
hsa_circ_0001486	1.148607018	9.35560397	-2.988	0.0097697	0.028907
hsa_circ_0002961	0	5.444181593	-5.8656	0.0097846	0.028932
hsa_circ_0030567	0	5.466951847	-5.8762	0.0098058	0.028977
hsa_circ_0003438	4.595854421	0	5.862	0.0098347	0.029025
novel_circ_0002208	4.595854421	0	5.862	0.0098347	0.029025
hsa_circ_0005164	0	5.721145197	-5.9119	0.0098435	0.029033
novel_circ_0016915	0	6.174466539	-5.9357	0.0098785	0.029118
novel_circ_0019973	0	5.632003461	-5.87	0.0099342	0.029264
novel_circ_0018704	0	5.527677041	-5.8589	0.0099436	0.029273
hsa_circ_0091244	0	5.737299523	-5.8801	0.0099741	0.029344
hsa_circ_0002500	8.707976392	26.256291	-1.5975	0.010028	0.029439
hsa_circ_0084188	0	5.634912383	-5.8672	0.010051	0.029439
novel_circ_0000441	0	5.5267074	-5.8563	0.010036	0.029439
novel_circ_0009646	0	5.42335062	-5.8452	0.010056	0.029439
novel_circ_0012561	0	5.631033821	-5.8667	0.010057	0.029439
novel_circ_0018087	0	5.634912383	-5.8672	0.010051	0.029439
novel_circ_0018430	0	5.529616321	-5.8567	0.010032	0.029439
novel_circ_0020183	4.670524709	0	5.8631	0.01005	0.029439
hsa_circ_0035298	0	5.42432026	-5.844	0.010107	0.029567
novel_circ_0012766	1.458232085	10.7205742	-2.6227	0.01017	0.029732
novel_circ_0001510	0	5.735360242	-5.8742	0.010195	0.029787
hsa_circ_0001621	0	5.841625944	-5.8847	0.010227	0.02986
novel_circ_0020606	0	5.52573776	-5.851	0.010232	0.02986
hsa_circ_0001666	0	5.635882023	-5.8616	0.010269	0.02988
hsa_circ_0002168	4.554412024	0	5.8444	0.010272	0.02988
hsa_circ_0005965	0	5.318054558	-5.8288	0.010275	0.02988
hsa_circ_0019172	0	5.421411339	-5.8391	0.010276	0.02988
novel_circ_0007987	0	5.553356216	-5.8614	0.010282	0.02988
novel_circ_0009645	0	5.341794453	-5.841	0.010284	0.02988
novel_circ_0017292	0	5.319024199	-5.8289	0.010274	0.02988
hsa_circ_0002535	22.66130924	6.451430143	1.7862	0.010298	0.029883
hsa_circ_0066535	7.058961334	23.82853833	-1.7594	0.010294	0.029883
hsa_circ_0001542	0	5.488752461	-5.8695	0.010311	0.029901
hsa_circ_0004374	25.69400939	9.629830207	1.4042	0.01037	0.030053
hsa_circ_0004730	0.583292834	6.682853239	-3.1525	0.010441	0.030242
hsa_circ_0005624	1.914345029	12.63833045	-2.6945	0.01052	0.030453

hsa_circ_0031749	0	5.235528751	-5.8232	0.010546	0.030469
hsa_circ_0047700	0	5.213728138	-5.8111	0.010543	0.030469
novel_circ_0013617	0.815518204	8.091424702	-3.2611	0.010542	0.030469
novel_circ_0017999	0	5.426259541	-5.8316	0.010575	0.030535
novel_circ_0006422	0	5.420441698	-5.8308	0.010584	0.030543
hsa_circ_0077705	0	5.62909454	-5.8516	0.010626	0.030605
novel_circ_0005366	0	5.214697778	-5.8091	0.010617	0.030605
novel_circ_0019384	0	5.212758497	-5.8089	0.01062	0.030605
hsa_circ_0003502	4.771388152	16.39714853	-1.7259	0.010667	0.03067
novel_circ_0002881	0	5.32096348	-5.8181	0.010676	0.03067
novel_circ_0005680	1.690457455	9.416329163	-2.3842	0.010694	0.03067
novel_circ_0009151	0	5.236498392	-5.82	0.010657	0.03067
novel_circ_0009185	0.583292834	6.602266713	-3.1532	0.010694	0.03067
novel_circ_0010942	0	5.360686146	-5.8392	0.010677	0.03067
novel_circ_0014063	1.989015318	9.754987601	-2.3461	0.010681	0.03067
hsa_circ_0003679	6.420403211	0.671499034	3.2415	0.010735	0.030769
hsa_circ_0071189	0.583292834	6.475170038	-3.1199	0.010754	0.030804
hsa_circ_0000853	9.03415376	25.53651409	-1.4696	0.01077	0.03083
novel_circ_0019046	2.371884324	15.27217015	-2.654	0.010821	0.030958
novel_circ_0012469	0	5.215667418	-5.8025	0.010858	0.031021
novel_circ_0013724	0	5.215667418	-5.8025	0.010858	0.031021
novel_circ_0016386	7.694763768	1.00724855	2.9469	0.01087	0.031021
novel_circ_0021861	0	5.211788857	-5.802	0.010865	0.031021
hsa_circ_0081813	42.60679844	14.93287163	1.5065	0.010907	0.031109
hsa_circ_0006956	27.87929277	71.39453398	-1.3547	0.010928	0.03114
novel_circ_0017404	1.148607018	8.134056289	-2.7881	0.010931	0.03114
hsa_circ_0006990	18.53130563	59.03233646	-1.6309	0.010998	0.031196
hsa_circ_0008073	0	5.296253945	-5.7968	0.010996	0.031196
hsa_circ_0060739	8.457772372	1.470094742	2.4478	0.010982	0.031196
novel_circ_0000303	0	5.297223585	-5.797	0.010995	0.031196
novel_circ_0000416	0	5.296253945	-5.7968	0.010996	0.031196
novel_circ_0003493	0	5.296253945	-5.7968	0.010996	0.031196
novel_circ_0008889	0	5.297223585	-5.797	0.010995	0.031196
novel_circ_0014955	4.420320689	0	5.7919	0.011016	0.031228
hsa_circ_0001383	0	5.237468032	-5.809	0.011032	0.031256
novel_circ_0017944	0	5.23358947	-5.8085	0.011039	0.031256
hsa_circ_0000414	0	5.295284304	-5.7943	0.011095	0.031318
hsa_circ_0007378	16.17051479	5.085661822	1.6721	0.011077	0.031318
hsa_circ_0022306	4.3290981	0	5.779	0.011107	0.031318
hsa_circ_0042880	0	5.192897164	-5.7827	0.011115	0.031318
hsa_circ_0064622	0	5.192897164	-5.7827	0.011115	0.031318
hsa_circ_0067479	0	5.419472058	-5.817	0.011101	0.031318
hsa_circ_0077292	0	5.295284304	-5.7943	0.011095	0.031318
novel_circ_0000727	0	5.400580365	-5.8066	0.01107	0.031318
hsa_circ_0053882	0	5.38442604	-5.8339	0.011153	0.031388
hsa_circ_0085323	0	5.38442604	-5.8339	0.011153	0.031388
novel_circ_0007914	0	5.315145637	-5.8039	0.011185	0.03144
novel_circ_0016502	0	5.315145637	-5.8039	0.011185	0.03144
hsa_circ_0004558	2.333171336	16.01488887	-2.5756	0.011266	0.031514
hsa_circ_0005612	0	5.086631462	-5.768	0.011254	0.031514
hsa_circ_0007183	0	5.506846067	-5.814	0.011259	0.031514
hsa_circ_0019006	0	5.086631462	-5.768	0.011254	0.031514
hsa_circ_0043975	4.304208004	0	5.7723	0.011272	0.031514
novel_circ_0000755	0	5.086631462	-5.768	0.011254	0.031514
novel_circ_0004168	0	5.086631462	-5.768	0.011254	0.031514
novel_circ_0009052	4.304208004	0	5.7723	0.011272	0.031514
novel_circ_0018284	0	5.087601103	-5.7681	0.011253	0.031514
hsa_circ_0005589	0	5.294314664	-5.7892	0.011305	0.031551
hsa_circ_0024242	0	5.294314664	-5.7892	0.011305	0.031551
hsa_circ_0081897	0	5.299162866	-5.7899	0.011297	0.031551
novel_circ_0005873	8.991285013	1.342998067	2.747	0.011319	0.031569
novel_circ_0016603	0	5.210819216	-5.789	0.011343	0.031619
hsa_circ_0001723	0	5.088570743	-5.7649	0.011387	0.031699
hsa_circ_0049291	6.360982165	0.671499034	3.2146	0.011392	0.031699
novel_circ_0019060	0	5.085661822	-5.7645	0.011392	0.031699

hsa_circ_0008546	38.90100922	15.80623603	1.307	0.011421	0.031741
novel_circ_0001873	10.55038054	1.15711548	2.9102	0.011416	0.031741
hsa_circ_0027528	0	5.404458927	-5.7984	0.011431	0.03175
novel_circ_0011282	6.011217759	0.462846192	3.2745	0.01146	0.031812
hsa_circ_0029633	0	4.982305042	-5.7507	0.011509	0.031899
hsa_circ_0032097	0	4.982305042	-5.7507	0.011509	0.031899
hsa_circ_0084642	0	5.003136015	-5.7637	0.011512	0.031899
hsa_circ_0005058	0	4.981335401	-5.7486	0.01159	0.032021
hsa_circ_0007442	0	4.983274682	-5.7488	0.011587	0.032021
hsa_circ_0039348	0	4.983274682	-5.7488	0.011587	0.032021
hsa_circ_0071234	1.223277306	8.030699509	-2.7498	0.011587	0.032021
novel_circ_0000124	0	4.983274682	-5.7488	0.011587	0.032021
hsa_circ_0007251	0	5.194836445	-5.7707	0.011614	0.03205
hsa_circ_0060527	0	5.194836445	-5.7707	0.011614	0.03205
hsa_circ_0012618	20.65716797	4.746033744	2.088	0.01164	0.032101
hsa_circ_0058728	4.262765607	0	5.7565	0.011681	0.032195
novel_circ_0013163	29.54628648	10.42357771	1.4803	0.011725	0.032297
hsa_circ_0049540	1.223277306	8.578980429	-2.8226	0.011734	0.032304
novel_circ_0013863	12.5380928	33.03250765	-1.389	0.011749	0.032325
novel_circ_0018679	0	5.502967506	-5.8016	0.011761	0.032338
hsa_circ_0002416	0	4.984244322	-5.7425	0.011844	0.032453
hsa_circ_0016040	4.188095318	0	5.7447	0.011845	0.032453
hsa_circ_0065623	4.188095318	0	5.7447	0.011845	0.032453
novel_circ_0006163	0	4.984244322	-5.7425	0.011844	0.032453
novel_circ_0010801	0	4.980365761	-5.7419	0.011851	0.032453
novel_circ_0011717	4.188095318	0	5.7447	0.011845	0.032453
novel_circ_0013764	0	4.980365761	-5.7419	0.011851	0.032453
hsa_circ_0071122	0	5.006044936	-5.7532	0.011907	0.032473
hsa_circ_0088048	0	4.87700898	-5.7299	0.011905	0.032473
hsa_circ_0091570	4.121762826	0	5.7353	0.011909	0.032473
novel_circ_0003570	0	5.002166375	-5.7526	0.011914	0.032473
novel_circ_0006159	0	4.877978621	-5.73	0.011903	0.032473
novel_circ_0006316	11.2815876	2.060037609	2.3073	0.011905	0.032473
novel_circ_0010861	0	5.002166375	-5.7526	0.011914	0.032473
novel_circ_0013948	4.121762826	0	5.7353	0.011909	0.032473
novel_circ_0018569	7.828855103	1.00724855	2.9663	0.011942	0.03253
hsa_circ_0007168	1.631036409	11.17663291	-2.7758	0.011994	0.032652
hsa_circ_0000349	0	4.898809594	-5.7393	0.012028	0.032727
hsa_circ_0020340	16.9722101	2.314230959	2.6759	0.012035	0.032727
hsa_circ_0055906	5.045055919	18.49708036	-1.8454	0.012064	0.032785
hsa_circ_0001148	1.80657014	9.269371155	-2.316	0.012099	0.032803
hsa_circ_0003761	0	4.87603934	-5.7245	0.012112	0.032803
hsa_circ_0077296	0	4.878948261	-5.7249	0.012107	0.032803
novel_circ_0001028	0	4.87603934	-5.7245	0.012112	0.032803
novel_circ_0002247	6.402424561	0.694269288	2.8481	0.012093	0.032803
novel_circ_0013334	0	4.87603934	-5.7245	0.012112	0.032803
novel_circ_0021685	1.223277306	8.431850867	-2.8242	0.012154	0.032897
hsa_circ_0076178	4.09687273	0	5.7262	0.012163	0.032902
hsa_circ_0007335	2.315192685	10.76320579	-2.1157	0.012255	0.033132
novel_circ_0005567	0	4.960504428	-5.7183	0.012265	0.03314
hsa_circ_0004092	0	5.064830849	-5.7299	0.012287	0.033143
hsa_circ_0008278	0	5.064830849	-5.7299	0.012287	0.033143
novel_circ_0005131	0	5.064830849	-5.7299	0.012287	0.033143
hsa_circ_0000235	0	4.900748875	-5.7307	0.012353	0.033177
hsa_circ_0003572	1.458232085	10.48721183	-2.5916	0.012363	0.033177
hsa_circ_0004556	0	4.900748875	-5.7307	0.012353	0.033177
hsa_circ_0027836	0	4.961474068	-5.7171	0.012324	0.033177
hsa_circ_0066273	0	4.897839954	-5.7303	0.012359	0.033177
novel_circ_0006566	0	4.772682559	-5.7075	0.012364	0.033177
novel_circ_0007662	0	4.772682559	-5.7075	0.012364	0.033177
novel_circ_0020700	0	4.772682559	-5.7075	0.012364	0.033177
novel_circ_0020947	0	4.772682559	-5.7075	0.012364	0.033177
hsa_circ_0001647	0	4.856178007	-5.7032	0.012412	0.033234
hsa_circ_0004977	0	4.855208367	-5.703	0.012414	0.033234
novel_circ_0010903	14.52983753	3.023684932	2.2856	0.012409	0.033234

novel_circ_0020728	0	4.855208367	-5.703	0.012414	0.033234
hsa_circ_0002231	0	4.7736522	-5.7044	0.012489	0.033369
hsa_circ_0007123	0	4.962443709	-5.713	0.012513	0.033369
hsa_circ_0007698	0.874939251	7.252934773	-2.7494	0.012504	0.033369
hsa_circ_0056590	0	4.958565147	-5.7124	0.012521	0.033369
novel_circ_0004837	0	4.958565147	-5.7124	0.012521	0.033369
novel_circ_0005560	0	4.771712919	-5.7041	0.012493	0.033369
novel_circ_0011347	10.58087903	2.606379249	1.9656	0.012504	0.033369
novel_circ_0015477	0.874939251	7.674119018	-2.8135	0.012515	0.033369
hsa_circ_0044698	0	4.857147647	-5.7001	0.012555	0.03344
hsa_circ_0032116	0	4.879917902	-5.7132	0.012578	0.033462
novel_circ_0008767	0	4.879917902	-5.7132	0.012578	0.033462
hsa_circ_0001955	0	4.750881946	-5.6863	0.012642	0.033504
hsa_circ_0004773	1.166585668	10.15323004	-2.864	0.01265	0.033504
hsa_circ_0005342	0	5.06773977	-5.7231	0.012617	0.033504
hsa_circ_0006126	0	4.750881946	-5.6863	0.012642	0.033504
hsa_circ_0007493	93.78315033	39.72649546	1.2359	0.012618	0.033504
novel_circ_0006335	4.030540237	0	5.706	0.012651	0.033504
novel_circ_0006778	0	4.750881946	-5.6863	0.012642	0.033504
novel_circ_0016581	4.030540237	0	5.706	0.012651	0.033504
hsa_circ_0002901	0	4.749912305	-5.6842	0.01273	0.033694
hsa_circ_0006805	0	4.920610208	-5.7299	0.012767	0.033773
hsa_circ_0004271	13.87057135	3.743633395	1.8479	0.012802	0.033847
hsa_circ_0000703	1.458232085	10.38676397	-2.5799	0.012815	0.033861
hsa_circ_0003131	0	4.858117288	-5.6931	0.012873	0.033968
hsa_circ_0004407	2.333171336	15.60340103	-2.5421	0.012884	0.033968
novel_circ_0016062	0	4.963413349	-5.7054	0.012865	0.033968
novel_circ_0018029	0	4.853269086	-5.6923	0.012883	0.033968
hsa_circ_0004700	0	4.77462184	-5.6938	0.012912	0.034002
novel_circ_0002430	34.04661299	6.99195394	2.2414	0.012908	0.034002
hsa_circ_0002835	0	4.795452814	-5.7049	0.012935	0.034025
novel_circ_0018245	0.815518204	6.814798116	-3.0731	0.012935	0.034025
hsa_circ_0000417	3.903360348	16.98321284	-2.1297	0.012946	0.034035
hsa_circ_0002513	44.08158283	18.71234913	1.2278	0.01297	0.034035
hsa_circ_0003748	0	4.667386498	-5.68	0.013025	0.034035
hsa_circ_0003873	0	4.645585884	-5.6659	0.013022	0.034035
hsa_circ_0007997	0	4.752821226	-5.6783	0.013	0.034035
hsa_circ_0064460	21.89151248	7.672179738	1.5292	0.013026	0.034035
hsa_circ_0076402	4.128674272	0	5.6908	0.012986	0.034035
novel_circ_0009780	0	4.645585884	-5.6659	0.013022	0.034035
novel_circ_0015262	4.062341779	0	5.6819	0.012986	0.034035
novel_circ_0018607	0	4.668356139	-5.6801	0.013023	0.034035
novel_circ_0019826	0	4.752821226	-5.6783	0.013	0.034035
novel_circ_0022043	0	4.752821226	-5.6783	0.013	0.034035
hsa_circ_0001701	19.08006751	5.189018603	1.8824	0.013133	0.034205
hsa_circ_0004015	1.148607018	7.904572474	-2.7428	0.013119	0.034205
hsa_circ_0015455	13.8193648	3.845050895	1.8164	0.013135	0.034205
novel_circ_0003434	4.012561587	0	5.6735	0.013116	0.034205
novel_circ_0013865	2.297214035	14.48988678	-2.6125	0.013135	0.034205
novel_circ_0022275	12.88773391	3.614597439	1.8227	0.013116	0.034205
hsa_circ_0037052	3.921338998	0	5.6593	0.013246	0.034457
novel_circ_0003647	0	4.647525165	-5.6611	0.013247	0.034457
hsa_circ_0002972	22.5549607	5.154942225	2.0388	0.013269	0.034494
novel_circ_0018378	15.46980621	3.194382835	2.1766	0.01331	0.034582
novel_circ_0004269	0	4.669325779	-5.6715	0.013371	0.034649
novel_circ_0004449	9.332711623	1.932940934	2.17	0.013373	0.034649
novel_circ_0011955	4.146652922	15.00570104	-1.8004	0.013366	0.034649
novel_circ_0019984	0	4.669325779	-5.6715	0.013371	0.034649
novel_circ_0022217	0	4.669325779	-5.6715	0.013371	0.034649
hsa_circ_0000412	130.1367678	63.00207622	1.0457	0.013434	0.03477
hsa_circ_0001017	292.8414287	654.7951056	-1.1532	0.013451	0.03477
hsa_circ_0001736	0	4.852299445	-5.6795	0.013457	0.03477
hsa_circ_0032849	3.97111919	0	5.6601	0.013457	0.03477
hsa_circ_0073177	0	4.859086928	-5.6806	0.013441	0.03477
hsa_circ_0007365	0	4.541259464	-5.6438	0.013467	0.034776

hsa_circ_0005952	1.148607018	7.58868429	-2.7011	0.013529	0.034919
hsa_circ_0004495	0	4.753790867	-5.6663	0.01354	0.034928
novel_circ_0015126	1.914345029	11.22799126	-2.5304	0.013564	0.034969
hsa_circ_0035185	0	4.540289823	-5.6405	0.01361	0.035051
novel_circ_0003022	0	4.540289823	-5.6405	0.01361	0.035051
novel_circ_0012369	3.98767149	0	5.6594	0.013627	0.035075
novel_circ_0016790	3.830116409	0	5.6392	0.013651	0.035098
novel_circ_0020281	3.830116409	0	5.6392	0.013651	0.035098
hsa_circ_0000619	25.44929046	8.640503709	1.5148	0.013785	0.035268
hsa_circ_0001431	0	4.648494806	-5.6497	0.01375	0.035268
hsa_circ_0047348	0	4.624754911	-5.6335	0.013783	0.035268
hsa_circ_0068013	0	4.623785271	-5.6333	0.013785	0.035268
novel_circ_0004242	0	4.643646604	-5.6489	0.013761	0.035268
novel_circ_0005028	0	4.623785271	-5.6333	0.013785	0.035268
novel_circ_0009989	0	4.563060077	-5.6507	0.013768	0.035268
novel_circ_0016851	0	4.689187112	-5.6738	0.013727	0.035268
novel_circ_0020914	0	4.648494806	-5.6497	0.01375	0.035268
hsa_circ_0008823	19.06066251	5.547538374	1.7634	0.01382	0.035317
hsa_circ_0075854	0	4.728111691	-5.646	0.013816	0.035317
hsa_circ_0000847	11.43092818	29.70327101	-1.3641	0.013892	0.035483
novel_circ_0004470	0	4.834377393	-5.6567	0.01394	0.035585
hsa_circ_0017306	0	4.564029718	-5.6455	0.013985	0.035662
hsa_circ_0060735	0	4.564029718	-5.6455	0.013985	0.035662
novel_circ_0006417	0.583292834	6.554786924	-3.1169	0.014	0.035681
hsa_circ_0007779	0	4.52042849	-5.6154	0.014025	0.035684
hsa_circ_0030632	0	4.727142051	-5.6417	0.014024	0.035684
novel_circ_0019769	0	4.52042849	-5.6154	0.014025	0.035684
hsa_circ_0058495	220.8159017	100.0239673	1.1318	0.014053	0.035738
novel_circ_0003932	0	4.539320183	-5.63	0.014087	0.035785
novel_circ_0016104	0	4.539320183	-5.63	0.014087	0.035785
hsa_circ_0016867	1.166585668	8.365479385	-2.568	0.014118	0.035844
hsa_circ_0012720	0	4.435963402	-5.6166	0.014139	0.035858
hsa_circ_0088744	7.211031319	1.010157472	2.8471	0.014139	0.035858
hsa_circ_0003936	0	4.414162788	-5.5972	0.014273	0.03606
hsa_circ_0042881	0	4.415132429	-5.5973	0.014271	0.03606
novel_circ_0008086	0	4.415132429	-5.5973	0.014271	0.03606
novel_circ_0011786	0	4.414162788	-5.5972	0.014273	0.03606
novel_circ_0012278	0	4.415132429	-5.5973	0.014271	0.03606
novel_circ_0013305	0	4.415132429	-5.5973	0.014271	0.03606
novel_circ_0019998	0	4.415132429	-5.5973	0.014271	0.03606
novel_circ_0015995	0	4.626694192	-5.6237	0.014305	0.036122
hsa_circ_0001944	0	4.521398131	-5.6095	0.014338	0.036126
hsa_circ_0002687	0	4.62184599	-5.6229	0.014317	0.036126
novel_circ_0004482	0	4.521398131	-5.6095	0.014338	0.036126
novel_circ_0018886	0	4.521398131	-5.6095	0.014338	0.036126
novel_circ_0008781	0	4.517519569	-5.6089	0.014346	0.036129
novel_circ_0005279	3.73889382	0	5.6108	0.01442	0.036294
novel_circ_0006018	13.76828155	36.69167595	-1.4201	0.014447	0.036343
hsa_circ_0006535	11.89537892	2.500113547	2.1538	0.014532	0.036404
hsa_circ_0008126	0	4.416102069	-5.5926	0.014516	0.036404
hsa_circ_0008406	0	4.416102069	-5.5926	0.014516	0.036404
hsa_circ_0040528	10.58909353	1.830553794	2.3798	0.014492	0.036404
hsa_circ_0085321	0	4.434993762	-5.6079	0.014533	0.036404
novel_circ_0003333	0	4.416102069	-5.5926	0.014516	0.036404
novel_circ_0004708	0	4.413193148	-5.5921	0.014522	0.036404
novel_circ_0020671	0	4.437902683	-5.6083	0.014527	0.036404
novel_circ_0010675	1.749878502	10.97282827	-2.4227	0.01465	0.036676
novel_circ_0021679	0	4.309836368	-5.5755	0.014707	0.036798
hsa_circ_0004705	8.999622809	2.143533057	2.0578	0.014775	0.036938
hsa_circ_0004849	20.0255213	52.16552636	-1.3696	0.014779	0.036938
novel_circ_0014552	0	4.457764016	-5.6143	0.014819	0.03702
novel_circ_0010292	0	4.310806008	-5.5726	0.014856	0.037083
novel_circ_0021658	0	4.308866727	-5.5723	0.01486	0.037083
hsa_circ_0003118	0	4.331636981	-5.5875	0.014905	0.037175
hsa_circ_0085362	0	4.627663832	-5.6115	0.01492	0.037194

hsa_circ_0068360	0	4.620876349	-5.6104	0.014939	0.037221
hsa_circ_0002081	0	4.522367771	-5.5979	0.014953	0.037234
hsa_circ_0072973	12.78268843	2.014497101	2.6514	0.015016	0.037351
novel_circ_0012800	5.93797382	19.38675709	-1.6456	0.01501	0.037351
hsa_circ_0001236	5.228927447	0.462846192	3.0862	0.015041	0.037395
hsa_circ_0007695	0	4.41707171	-5.5816	0.015084	0.037482
hsa_circ_0001961	0	4.332606622	-5.5822	0.015156	0.03762
novel_circ_0004751	0	4.332606622	-5.5822	0.015156	0.03762
hsa_circ_0002359	14.60162884	68.64360453	-2.1651	0.015233	0.037791
hsa_circ_0004820	33.95408734	12.4162742	1.4501	0.0153	0.037937
hsa_circ_0003030	0	4.205509947	-5.5489	0.015389	0.038043
hsa_circ_0007995	0	4.311775648	-5.5627	0.015363	0.038043
hsa_circ_0056040	0	4.204540306	-5.5487	0.015391	0.038043
hsa_circ_0082988	0	4.204540306	-5.5487	0.015391	0.038043
novel_circ_0010709	0	4.307897087	-5.562	0.015372	0.038043
novel_circ_0019181	0	4.311775648	-5.5627	0.015363	0.038043
hsa_circ_0001974	3.770695362	0	5.5725	0.015407	0.038062
hsa_circ_0002039	0	4.393331815	-5.5579	0.015438	0.038063
novel_circ_0004808	2.779643426	11.54097053	-2.0928	0.015434	0.038063
novel_circ_0012816	0	4.392362175	-5.5577	0.015441	0.038063
novel_circ_0020548	0	4.392362175	-5.5577	0.015441	0.038063
hsa_circ_0015365	0.815518204	6.116650267	-2.9303	0.015458	0.038065
novel_circ_0015633	3.654582677	0	5.556	0.01545	0.038065
hsa_circ_0007081	3.570271534	16.5109998	-2.2242	0.015475	0.038089
hsa_circ_0008380	5.411372625	0.671499034	3.0012	0.015492	0.03811
hsa_circ_0000944	21.57497597	7.339339142	1.566	0.015504	0.038118
hsa_circ_0002468	0	4.287066113	-5.5406	0.015588	0.038204
hsa_circ_0005044	1.223277306	8.540055849	-2.8209	0.015574	0.038204
hsa_circ_0017850	0	4.287066113	-5.5406	0.015588	0.038204
hsa_circ_0060530	0	4.287066113	-5.5406	0.015588	0.038204
novel_circ_0012341	0	4.289005394	-5.5409	0.015583	0.038204
novel_circ_0019395	0	4.289005394	-5.5409	0.015583	0.038204
novel_circ_0017949	2.346994228	14.09211286	-2.5522	0.015734	0.038542
novel_circ_0019366	0	4.182739693	-5.5232	0.015772	0.038615
hsa_circ_0001513	0	4.206479587	-5.5406	0.015805	0.038616
hsa_circ_0018761	0	4.206479587	-5.5406	0.015805	0.038616
hsa_circ_0072700	0	4.206479587	-5.5406	0.015805	0.038616
novel_circ_0005014	0	4.206479587	-5.5406	0.015805	0.038616
hsa_circ_0014878	3.795585459	0	5.5681	0.015821	0.038635
hsa_circ_0007440	26.53441769	9.774848934	1.4064	0.015836	0.03865
hsa_circ_0012828	0.765738012	6.012323846	-2.9299	0.01588	0.038737
hsa_circ_0057980	0	4.286096473	-5.5345	0.015932	0.038844
hsa_circ_0001564	2.857043123	12.82518268	-2.0521	0.01599	0.038908
hsa_circ_0012545	0	4.184678973	-5.5188	0.016034	0.038908
hsa_circ_0029909	0	4.395271096	-5.5483	0.01602	0.038908
hsa_circ_0035572	0	4.181770052	-5.5183	0.016042	0.038908
hsa_circ_0078162	0	4.22731056	-5.5512	0.01602	0.038908
novel_circ_0002269	0	4.390422894	-5.5475	0.016033	0.038908
novel_circ_0002890	0	4.22731056	-5.5512	0.01602	0.038908
novel_circ_0003848	0	4.184678973	-5.5188	0.016034	0.038908
novel_circ_0004120	0	4.184678973	-5.5188	0.016034	0.038908
novel_circ_0011518	0	4.181770052	-5.5183	0.016042	0.038908
hsa_circ_0006810	9.549687751	2.375925793	1.9812	0.016087	0.038998
hsa_circ_0000115	0	4.100213885	-5.5198	0.016183	0.03909
hsa_circ_0006747	0	4.078413272	-5.5021	0.016181	0.03909
hsa_circ_0038241	0	4.100213885	-5.5198	0.016183	0.03909
hsa_circ_0052508	1.166585668	8.78569399	-2.6298	0.016156	0.03909
novel_circ_0002876	0	4.078413272	-5.5021	0.016181	0.03909
novel_circ_0007333	0	4.100213885	-5.5198	0.016183	0.03909
novel_circ_0018396	0	4.078413272	-5.5021	0.016181	0.03909
hsa_circ_0010539	2.333171336	14.0202531	-2.391	0.016235	0.039194
hsa_circ_0006140	0	4.077443631	-5.4989	0.016346	0.039381
hsa_circ_0024323	0	4.077443631	-5.4989	0.016346	0.039381
novel_circ_0005120	0	4.077443631	-5.4989	0.016346	0.039381
novel_circ_0007778	0	4.079382912	-5.4993	0.016341	0.039381

hsa_circ_0006434	0	4.099244245	-5.5142	0.016457	0.039547
hsa_circ_0009137	0	4.101183526	-5.5146	0.016452	0.039547
hsa_circ_0053860	0	4.099244245	-5.5142	0.016457	0.039547
hsa_circ_0077276	0	4.101183526	-5.5146	0.016452	0.039547
novel_circ_0011107	0	4.101183526	-5.5146	0.016452	0.039547
hsa_circ_0004663	1.148607018	7.464496536	-2.6661	0.016537	0.039717
hsa_circ_0000603	0	4.285126833	-5.5229	0.016603	0.039814
hsa_circ_0009098	3.447247403	0	5.5057	0.016653	0.039814
hsa_circ_0029787	5.404461179	0.671499034	3.0159	0.016649	0.039814
hsa_circ_0082401	0	4.180800412	-5.5073	0.016662	0.039814
hsa_circ_0084083	5.369930229	0.462846192	3.1117	0.016645	0.039814
novel_circ_0007661	0	4.290944675	-5.524	0.016586	0.039814
novel_circ_0011929	3.447247403	0	5.5057	0.016653	0.039814
novel_circ_0017865	0	4.180800412	-5.5073	0.016662	0.039814
novel_circ_0018291	3.447247403	0	5.5057	0.016653	0.039814
novel_circ_0020178	3.447247403	0	5.5057	0.016653	0.039814
hsa_circ_0005050	0	3.974086851	-5.4758	0.016868	0.04023
hsa_circ_0029638	0	3.974086851	-5.4758	0.016868	0.04023
hsa_circ_0075402	0	3.97311721	-5.4757	0.016871	0.04023
novel_circ_0004300	0	3.97311721	-5.4757	0.016871	0.04023
hsa_circ_0009084	32.79165743	12.95130972	1.3218	0.016886	0.040246
hsa_circ_0002626	0.765738012	6.096788934	-2.9243	0.016997	0.040489
hsa_circ_0008608	25.67460439	5.954507574	2.0225	0.017024	0.040534
hsa_circ_0002015	42.75198326	16.97933428	1.3197	0.017066	0.040598
hsa_circ_0030585	1.93232368	9.313942023	-2.1458	0.017069	0.040598
hsa_circ_0001400	29.9318849	7.39601422	1.9597	0.017079	0.040602
hsa_circ_0061509	0	4.493779674	-5.5432	0.017098	0.040604
novel_circ_0011234	6.103866698	0.694269288	2.8013	0.017094	0.040604
novel_circ_0018613	0.765738012	6.428659889	-2.9873	0.017241	0.040923
hsa_circ_0000365	6.539245304	0.673438314	3.2398	0.017266	0.04093
hsa_circ_0008520	0	4.122014499	-5.5125	0.017278	0.04093
novel_circ_0000176	0	4.122014499	-5.5125	0.017278	0.04093
novel_circ_0005013	0	4.122014499	-5.5125	0.017278	0.04093
hsa_circ_0002004	0	3.975056491	-5.4678	0.017314	0.040952
hsa_circ_0044793	0	3.975056491	-5.4678	0.017314	0.040952
novel_circ_0009843	0	3.97214757	-5.4673	0.017323	0.040952
novel_circ_0016860	0	3.97214757	-5.4673	0.017323	0.040952
novel_circ_0016838	0	4.056612658	-5.4616	0.01741	0.041138
hsa_circ_0000130	0	4.055643017	-5.4596	0.01753	0.041254
hsa_circ_0003399	0	4.055643017	-5.4596	0.01753	0.041254
hsa_circ_0005419	0	4.055643017	-5.4596	0.01753	0.041254
hsa_circ_0006768	0	4.057582298	-5.46	0.017524	0.041254
novel_circ_0004738	0	4.055643017	-5.4596	0.01753	0.041254
novel_circ_0007639	0	4.055643017	-5.4596	0.01753	0.041254
novel_circ_0013372	0	4.055643017	-5.4596	0.01753	0.041254
novel_circ_0018086	0	4.057582298	-5.46	0.017524	0.041254
hsa_circ_0078198	0	4.161908719	-5.4748	0.017584	0.041327
novel_circ_0002159	0	4.160939079	-5.4746	0.017588	0.041327
novel_circ_0014433	0	4.161908719	-5.4748	0.017584	0.041327
hsa_circ_0002153	0	3.952286237	-5.4434	0.0176	0.041334
hsa_circ_0007271	0	3.868790789	-5.4471	0.017685	0.041514
novel_circ_0017690	3.331134718	0	5.4698	0.017735	0.04161
hsa_circ_0008131	12.87951941	2.9668383	2.0225	0.017759	0.041645
hsa_circ_0004836	0	4.159969438	-5.4713	0.017795	0.04171
hsa_circ_0001337	0	4.058551939	-5.4544	0.017896	0.041786
hsa_circ_0001826	0	3.953255877	-5.439	0.017887	0.041786
hsa_circ_0043893	11.06876723	1.678747584	2.7056	0.017839	0.041786
hsa_circ_0054960	0	4.054673377	-5.4537	0.017908	0.041786
hsa_circ_0060665	0	3.953255877	-5.439	0.017887	0.041786
hsa_circ_0067773	0	3.953255877	-5.439	0.017887	0.041786
novel_circ_0009921	0	3.953255877	-5.439	0.017887	0.041786
novel_circ_0016013	0	3.950346956	-5.4384	0.017897	0.041786
novel_circ_0019047	0	4.054673377	-5.4537	0.017908	0.041786
hsa_circ_0007473	0	3.846990176	-5.4226	0.01796	0.041823
hsa_circ_0008478	0	3.846990176	-5.4226	0.01796	0.041823

hsa_circ_0038705	0	3.846990176	-5.4226	0.01796	0.041823
novel_circ_0007776	0	3.846990176	-5.4226	0.01796	0.041823
novel_circ_0000979	0	3.867821149	-5.4416	0.017979	0.041846
hsa_circ_0001483	0	3.846020535	-5.4196	0.018139	0.04201
hsa_circ_0001993	0	3.847959816	-5.4199	0.018133	0.04201
hsa_circ_0003991	0	3.846020535	-5.4196	0.018139	0.04201
hsa_circ_0004243	2.446554613	15.62793901	-2.6554	0.018062	0.04201
hsa_circ_0004768	0	3.846020535	-5.4196	0.018139	0.04201
hsa_circ_0005493	0	3.846020535	-5.4196	0.018139	0.04201
hsa_circ_0037000	0.583292834	6.197236793	-3.0277	0.018113	0.04201
hsa_circ_0052867	0	3.847959816	-5.4199	0.018133	0.04201
novel_circ_0015368	0	3.847959816	-5.4199	0.018133	0.04201
novel_circ_0019797	0	3.847959816	-5.4199	0.018133	0.04201
hsa_circ_0087264	20.81890509	51.14795488	-1.3017	0.018218	0.042172
novel_circ_0016018	0	4.158999798	-5.4644	0.018247	0.042218
hsa_circ_0002847	19.85689901	44.72575291	-1.1648	0.018266	0.042241
novel_circ_0022113	5.260728989	0.462846192	3.0653	0.018362	0.042442
novel_circ_0013833	1.148607018	7.880832579	-2.7175	0.018393	0.042492
hsa_circ_0008731	26.63528113	4.028994202	2.6579	0.018488	0.04267
hsa_circ_0047719	0.815518204	5.907027785	-2.8874	0.018486	0.04267
novel_circ_0002488	3.36293626	0	5.4354	0.018506	0.042691
hsa_circ_0003939	0	3.949377316	-5.4278	0.018571	0.042773
hsa_circ_0039935	0	3.741694114	-5.3967	0.018643	0.042773
hsa_circ_0061137	0	3.742663755	-5.3968	0.018639	0.042773
novel_circ_0002760	0	4.059521579	-5.4434	0.018629	0.042773
novel_circ_0002877	0	3.741694114	-5.3967	0.018643	0.042773
novel_circ_0006731	0	3.741694114	-5.3967	0.018643	0.042773
novel_circ_0007211	6.662269436	1.00724855	2.7472	0.018625	0.042773
novel_circ_0009757	0	3.742663755	-5.3968	0.018639	0.042773
novel_circ_0012430	0	4.059521579	-5.4434	0.018629	0.042773
novel_circ_0015554	0	4.059521579	-5.4434	0.018629	0.042773
novel_circ_0019521	0	3.741694114	-5.3967	0.018643	0.042773
hsa_circ_0002657	0	3.890591403	-5.4444	0.018701	0.042848
hsa_circ_0008755	0	3.848929457	-5.4106	0.018721	0.042848
novel_circ_0012505	0	3.848929457	-5.4106	0.018721	0.042848
novel_circ_0017736	0	3.890591403	-5.4444	0.018701	0.042848
novel_circ_0019966	0	3.848929457	-5.4106	0.018721	0.042848
hsa_circ_0001921	5.928332966	0.694269288	2.75	0.018741	0.042852
novel_circ_0020740	0	3.845050895	-5.4099	0.018735	0.042852
hsa_circ_0037409	3.246823575	0	5.414	0.018791	0.042945
novel_circ_0009055	3.387826356	0	5.4332	0.018852	0.043062
hsa_circ_0003250	0	3.764464369	-5.4105	0.018938	0.043141
hsa_circ_0020001	0	3.764464369	-5.4105	0.018938	0.043141
hsa_circ_0030582	7.685122914	25.98012548	-1.7356	0.018927	0.043141
hsa_circ_0030586	11.33279415	32.35775658	-1.4958	0.018942	0.043141
novel_circ_0005542	0	3.764464369	-5.4105	0.018938	0.043141
novel_circ_0013658	0	3.763494728	-5.4103	0.018942	0.043141
hsa_circ_0030594	18.2618199	4.015748798	2.077	0.018984	0.043217
hsa_circ_0000724	2.913734762	11.7743329	-1.9951	0.019041	0.043283
novel_circ_0002757	0	4.158030158	-5.4523	0.019038	0.043283
novel_circ_0008552	0	4.158030158	-5.4523	0.019038	0.043283
hsa_circ_0001344	0	3.743633395	-5.3891	0.01912	0.043295
hsa_circ_0003930	0	3.743633395	-5.3891	0.01912	0.043295
hsa_circ_0018054	0	3.743633395	-5.3891	0.01912	0.043295
hsa_circ_0029636	0	3.740724474	-5.3885	0.01913	0.043295
hsa_circ_0035958	0	3.743633395	-5.3891	0.01912	0.043295
hsa_circ_0039932	0	3.740724474	-5.3885	0.01913	0.043295
hsa_circ_0087765	13.39778281	3.275939001	2.0065	0.019083	0.043295
novel_circ_0013219	0	3.743633395	-5.3891	0.01912	0.043295
novel_circ_0021240	0	3.743633395	-5.3891	0.01912	0.043295
hsa_circ_0033180	3.221933479	0	5.4042	0.019189	0.043386
novel_circ_0014232	3.221933479	0	5.4042	0.019189	0.043386
hsa_circ_0061009	3.313156068	0	5.4175	0.0192	0.04339
hsa_circ_0005594	22.93782971	9.144213761	1.3357	0.01936	0.043732
hsa_circ_0006209	17.38415124	5.531555602	1.6527	0.019444	0.043897

hsa_circ_0006914	0	3.637367694	-5.3684	0.019471	0.043897
hsa_circ_0027244	0	3.637367694	-5.3684	0.019471	0.043897
novel_circ_0012039	0	3.637367694	-5.3684	0.019471	0.043897
novel_circ_0001225	3.155600986	0	5.3884	0.019542	0.043993
novel_circ_0007831	3.155600986	0	5.3884	0.019542	0.043993
novel_circ_0016545	3.155600986	0	5.3884	0.019542	0.043993
hsa_circ_0001980	15.03154863	4.625724551	1.7197	0.01959	0.04408
hsa_circ_0007832	0	3.636398053	-5.363	0.019788	0.044503
hsa_circ_0000166	0	3.719893501	-5.3559	0.019895	0.044617
hsa_circ_0056362	4.128674272	14.10083963	-1.771	0.01988	0.044617
hsa_circ_0067233	0	3.720863141	-5.3561	0.019892	0.044617
novel_circ_0006799	0	3.719893501	-5.3559	0.019895	0.044617
novel_circ_0011459	0	3.719893501	-5.3559	0.019895	0.044617
novel_circ_0017182	0	3.719893501	-5.3559	0.019895	0.044617
hsa_circ_0008460	3.714003724	13.68918023	-1.8386	0.019969	0.04476
hsa_circ_0009353	46.3913167	20.4348695	1.1858	0.019989	0.044782
hsa_circ_0056714	0	3.824219922	-5.3704	0.020056	0.04489
novel_circ_0018416	0	3.826159202	-5.3708	0.020048	0.04489
novel_circ_0000522	0	3.61556708	-5.3361	0.020148	0.045052
novel_circ_0015259	0	3.61556708	-5.3361	0.020148	0.045052
hsa_circ_0020181	1.398811038	7.274735387	-2.3173	0.020176	0.045093
hsa_circ_0024230	0	3.721832781	-5.3518	0.020208	0.045101
hsa_circ_0030509	0	3.721832781	-5.3518	0.020208	0.045101
novel_circ_0016187	0	3.721832781	-5.3518	0.020208	0.045101
novel_circ_0012153	0	3.71892386	-5.3512	0.02022	0.045105
hsa_circ_0009096	0	3.614597439	-5.3332	0.020344	0.045161
hsa_circ_0039388	0	3.614597439	-5.3332	0.020344	0.045161
hsa_circ_0039389	0	3.61653672	-5.3336	0.020336	0.045161
hsa_circ_0070552	8.6581962	1.932940934	2.0614	0.020361	0.045161
hsa_circ_0071580	0	3.659168307	-5.3713	0.020355	0.045161
novel_circ_0007834	0	3.61653672	-5.3336	0.020336	0.045161
novel_circ_0010820	0	3.614597439	-5.3332	0.020344	0.045161
novel_circ_0011240	0	3.614597439	-5.3332	0.020344	0.045161
novel_circ_0011248	0	3.61653672	-5.3336	0.020336	0.045161
novel_circ_0016528	0	3.614597439	-5.3332	0.020344	0.045161
novel_circ_0018622	0	3.614597439	-5.3332	0.020344	0.045161
novel_circ_0019367	0	3.61653672	-5.3336	0.020336	0.045161
novel_circ_0013019	0.583292834	5.445151233	-2.8738	0.020392	0.045208
hsa_circ_0004599	1.690457455	8.264061885	-2.1892	0.020404	0.045213
hsa_circ_0087247	1.531476023	8.494515341	-2.4518	0.020432	0.045238
hsa_circ_0091072	0.874939251	7.107916046	-2.7066	0.020434	0.045238
novel_circ_0009379	0	3.823250281	-5.3645	0.020482	0.045323
hsa_circ_0003673	23.57505849	8.473684368	1.4843	0.020504	0.04535
hsa_circ_0007108	2.680083041	15.26668187	-2.4615	0.020523	0.045371
hsa_circ_0062885	26.30910376	10.71960456	1.3023	0.020622	0.045568
hsa_circ_0002801	28.25652711	9.370788655	1.5918	0.020757	0.045588
hsa_circ_0003832	12.19248415	35.77356914	-1.5135	0.02068	0.045588
hsa_circ_0004227	0	3.511240659	-5.311	0.020793	0.045588
hsa_circ_0004550	5.828772581	17.45187687	-1.5436	0.020666	0.045588
hsa_circ_0005116	0	3.510271019	-5.3108	0.020797	0.045588
hsa_circ_0005394	0	3.532071632	-5.3319	0.020791	0.045588
hsa_circ_0007046	0	3.533041273	-5.3321	0.020787	0.045588
hsa_circ_0008434	0	3.511240659	-5.311	0.020793	0.045588
hsa_circ_0008945	0	3.511240659	-5.311	0.020793	0.045588
hsa_circ_0009063	0	3.532071632	-5.3319	0.020791	0.045588
hsa_circ_0044650	0	3.532071632	-5.3319	0.020791	0.045588
hsa_circ_0055002	0	3.510271019	-5.3108	0.020797	0.045588
hsa_circ_0056248	0.765738012	6.302532855	-2.9463	0.020756	0.045588
novel_circ_0003627	0	3.510271019	-5.3108	0.020797	0.045588
novel_circ_0004278	0	3.511240659	-5.311	0.020793	0.045588
novel_circ_0008088	0	3.532071632	-5.3319	0.020791	0.045588
novel_circ_0016135	2.023546268	9.610938514	-2.1456	0.020735	0.045588
hsa_circ_0000179	74.12397202	35.50015454	1.0632	0.020808	0.045589
hsa_circ_0008212	11.10748022	1.851384767	2.3667	0.02089	0.045748
hsa_circ_0001163	0	3.613627799	-5.3238	0.020994	0.04575

hsa_circ_0002212	0	3.613627799	-5.3238	0.020994	0.04575
hsa_circ_0002669	0	3.613627799	-5.3238	0.020994	0.04575
hsa_circ_0005904	32.99210754	13.07549747	1.3004	0.020998	0.04575
hsa_circ_0054178	0	3.613627799	-5.3238	0.020994	0.04575
hsa_circ_0074486	0	3.71795422	-5.3409	0.020961	0.04575
novel_circ_0004577	5.144616304	0.673438314	2.9141	0.020947	0.04575
novel_circ_0005774	0	3.617506361	-5.3246	0.020978	0.04575
novel_circ_0016494	0	3.613627799	-5.3238	0.020994	0.04575
novel_circ_0018050	0	3.617506361	-5.3246	0.020978	0.04575
novel_circ_0021647	0	3.613627799	-5.3238	0.020994	0.04575
hsa_circ_0007430	2.839064473	10.78985461	-1.9004	0.021075	0.045896
hsa_circ_0003017	0.874939251	7.1306863	-2.7216	0.021181	0.046105
hsa_circ_0000234	0	3.828098483	-5.3543	0.021278	0.04612
hsa_circ_0001985	0	3.512210299	-5.3035	0.021314	0.04612
hsa_circ_0002802	3.372577114	12.95130972	-1.8798	0.021245	0.04612
hsa_circ_0007737	0	3.512210299	-5.3035	0.021314	0.04612
hsa_circ_0019610	0	3.512210299	-5.3035	0.021314	0.04612
hsa_circ_0057151	0	3.822280641	-5.3531	0.021305	0.04612
hsa_circ_0083974	0	3.512210299	-5.3035	0.021314	0.04612
novel_circ_0000523	0	3.822280641	-5.3531	0.021305	0.04612
novel_circ_0002294	0	3.509301378	-5.3029	0.021326	0.04612
novel_circ_0011067	0	3.509301378	-5.3029	0.021326	0.04612
novel_circ_0011526	0	3.822280641	-5.3531	0.021305	0.04612
novel_circ_0012193	0	3.509301378	-5.3029	0.021326	0.04612
novel_circ_0012288	0	3.512210299	-5.3035	0.021314	0.04612
novel_circ_0021438	0	3.512210299	-5.3035	0.021314	0.04612
novel_circ_0002422	10.16893789	2.16436403	2.1461	0.02135	0.04615
novel_circ_0000965	0.874939251	6.578526819	-2.6189	0.021362	0.046154
hsa_circ_0054963	21.52116331	6.940925151	1.5848	0.021421	0.04626
hsa_circ_0004608	1.166585668	7.801215694	-2.471	0.021553	0.046523
hsa_circ_0000182	12.08055351	30.01818956	-1.2951	0.021591	0.046577
hsa_circ_0001018	0	3.405944598	-5.2829	0.021628	0.046577
hsa_circ_0004348	0	3.405944598	-5.2829	0.021628	0.046577
novel_circ_0000799	0	3.405944598	-5.2829	0.021628	0.046577
novel_circ_0007635	1.148607018	7.228225238	-2.6122	0.021625	0.046577
novel_circ_0002251	1.349030846	7.528928737	-2.3849	0.021681	0.04667
hsa_circ_0034972	72.98773527	30.37927514	1.2481	0.021703	0.046696
novel_circ_0004888	15.53195667	3.148842326	2.2774	0.021714	0.046699
hsa_circ_0030049	4.212985415	13.30885985	-1.6465	0.021728	0.046707
hsa_circ_0000105	17.42286423	43.14762474	-1.3079	0.021769	0.046755
novel_circ_0001869	13.17116583	3.86976043	1.7014	0.02177	0.046755
hsa_circ_0006428	29.16887629	11.24494368	1.3531	0.021809	0.046816
hsa_circ_0008286	1.398811038	7.149577993	-2.2853	0.021884	0.046956
hsa_circ_0000469	0	3.406914238	-5.2781	0.021962	0.047098
novel_circ_0005232	0	3.404974957	-5.2777	0.02197	0.047098
novel_circ_0016686	0.583292834	5.528646681	-2.8699	0.022028	0.0472
novel_circ_0012925	38.8431641	14.84549763	1.3668	0.022117	0.047369
hsa_circ_0005009	0.815518204	5.550447295	-2.7919	0.022196	0.047481
hsa_circ_0005728	131.4221248	61.11046767	1.1006	0.0222	0.047481
novel_circ_0004309	0.815518204	5.549477654	-2.7918	0.022198	0.047481
novel_circ_0000677	1.531476023	8.367418666	-2.4252	0.022212	0.047484
novel_circ_0018203	10.16893789	2.141593776	2.2321	0.022275	0.047599
novel_circ_0019466	0	3.427745211	-5.2925	0.022305	0.047641
hsa_circ_0001247	24.86742398	60.1287132	-1.2735	0.02232	0.04765
novel_circ_0018658	0.583292834	5.422380979	-2.8495	0.022375	0.047746
hsa_circ_0002566	8.116345762	22.37345672	-1.473	0.022416	0.047812
hsa_circ_0071174	4.083049837	24.15750037	-2.4147	0.022466	0.047897
hsa_circ_0002397	0.874939251	6.709502055	-2.6532	0.022698	0.048368
hsa_circ_0063408	2.955177158	0	5.2792	0.022776	0.048491
hsa_circ_0088910	2.955177158	0	5.2792	0.022776	0.048491
hsa_circ_0001403	0	3.384143984	-5.2413	0.022907	0.048614
hsa_circ_0023940	1.531476023	8.577041148	-2.45	0.022895	0.048614
hsa_circ_0030256	0	3.384143984	-5.2413	0.022907	0.048614
novel_circ_0008579	0	3.488470405	-5.2593	0.022887	0.048614
novel_circ_0012162	0	3.488470405	-5.2593	0.022887	0.048614

novel_circ_0017707	0	3.384143984	-5.2413	0.022907	0.048614
novel_circ_0021053	0	3.489440045	-5.2595	0.022882	0.048614
hsa_circ_0008893	6.508896392	0.694269288	2.8566	0.022997	0.048626
hsa_circ_0066278	0	3.300648536	-5.2467	0.023006	0.048626
novel_circ_0000592	0	3.300648536	-5.2467	0.023006	0.048626
novel_circ_0002750	0	3.300648536	-5.2467	0.023006	0.048626
novel_circ_0003344	2.980067254	0	5.2803	0.022956	0.048626
novel_circ_0012189	2.980067254	0	5.2803	0.022956	0.048626
novel_circ_0012477	0	3.300648536	-5.2467	0.023006	0.048626
novel_circ_0013294	0	3.301618177	-5.2469	0.023001	0.048626
novel_circ_0020707	0	3.300648536	-5.2467	0.023006	0.048626
hsa_circ_0057727	0.765738012	5.445151233	-2.7897	0.02304	0.048677
hsa_circ_0000073	0	3.385113624	-5.2388	0.023115	0.0487
hsa_circ_0002664	0.815518204	5.572247909	-2.807	0.023078	0.0487
novel_circ_0004733	0	3.385113624	-5.2388	0.023115	0.0487
novel_circ_0009586	19.14224425	4.705341437	2.03	0.023124	0.0487
novel_circ_0015998	0	3.383174343	-5.2384	0.023124	0.0487
novel_circ_0021077	0	3.383174343	-5.2384	0.023124	0.0487
novel_circ_0022234	0	3.385113624	-5.2388	0.023115	0.0487
hsa_circ_0010051	4.996702077	0.673438314	2.9052	0.02314	0.04871
novel_circ_0000445	11.07294927	1.851384767	2.3575	0.023203	0.048822
hsa_circ_0049267	2.930287062	0	5.2688	0.023266	0.048887
novel_circ_0002559	2.930287062	0	5.2688	0.023266	0.048887
novel_circ_0017569	0	3.487500764	-5.2547	0.023253	0.048887
hsa_circ_0000819	0	3.279817563	-5.2169	0.023464	0.04905
hsa_circ_0000852	0	3.279817563	-5.2169	0.023464	0.04905
hsa_circ_0002485	0	3.278847923	-5.2167	0.023469	0.04905
hsa_circ_0014191	0	3.278847923	-5.2167	0.023469	0.04905
hsa_circ_0056840	0	3.278847923	-5.2167	0.023469	0.04905
hsa_circ_0081342	0	3.279817563	-5.2169	0.023464	0.04905
hsa_circ_0091576	2.863954569	0	5.2559	0.023394	0.04905
novel_circ_0000585	2.863954569	0	5.2559	0.023394	0.04905
novel_circ_0008817	1.148607018	7.942355859	-2.7346	0.023378	0.04905
novel_circ_0017043	0	3.279817563	-5.2169	0.023464	0.04905
novel_circ_0017806	0	3.278847923	-5.2167	0.023469	0.04905
novel_circ_0018719	0	3.278847923	-5.2167	0.023469	0.04905
hsa_circ_0002976	2.839064473	0	5.2503	0.023548	0.049171
novel_circ_0006331	2.839064473	0	5.2503	0.023548	0.049171
hsa_circ_0000669	11.96717023	1.851384767	2.4818	0.023641	0.049321
novel_circ_0004304	5.778992388	21.03788622	-1.8094	0.023641	0.049321
hsa_circ_0000021	69.08870653	34.33173293	1.0026	0.023653	0.049324
hsa_circ_0058497	52.6002026	21.97213381	1.2377	0.023684	0.049367
hsa_circ_0001811	33.80619939	74.32890563	-1.1371	0.023859	0.049545
hsa_circ_0002542	13.17116583	3.090056414	1.9737	0.023861	0.049545
hsa_circ_0006487	0	3.386083265	-5.2301	0.023826	0.049545
hsa_circ_0027353	0	3.382204703	-5.2293	0.023846	0.049545
hsa_circ_0056558	0	3.386083265	-5.2301	0.023826	0.049545
novel_circ_0002718	0	3.386083265	-5.2301	0.023826	0.049545
novel_circ_0006139	0	3.386083265	-5.2301	0.023826	0.049545
novel_circ_0015202	7.826125696	1.597191417	2.1546	0.023865	0.049545
novel_circ_0015583	0	3.382204703	-5.2293	0.023846	0.049545
hsa_circ_0009104	0	3.280787203	-5.2097	0.024035	0.049774
hsa_circ_0031731	0	3.277878282	-5.2091	0.02405	0.049774
hsa_circ_0076410	0	3.277878282	-5.2091	0.02405	0.049774
novel_circ_0001976	0	3.277878282	-5.2091	0.02405	0.049774
novel_circ_0006252	0	3.277878282	-5.2091	0.02405	0.049774
novel_circ_0008804	0	3.280787203	-5.2097	0.024035	0.049774
novel_circ_0018260	0	3.277878282	-5.2091	0.02405	0.049774
hsa_circ_0001429	0	3.486531124	-5.2443	0.024093	0.049818
hsa_circ_0023806	0	3.486531124	-5.2443	0.024093	0.049818
hsa_circ_0002028	0	3.174521502	-5.1893	0.024275	0.049951
hsa_circ_0002117	0	3.174521502	-5.1893	0.024275	0.049951
hsa_circ_0003856	8.524104864	22.45598253	-1.4091	0.024203	0.049951
hsa_circ_0028201	0	3.174521502	-5.1893	0.024275	0.049951
hsa_circ_0046029	0	3.174521502	-5.1893	0.024275	0.049951

hsa_circ_0054345	0	3.174521502	-5.1893	0.024275	0.049951
novel_circ_0010124	5.969775363	1.00724855	2.5883	0.024225	0.049951
novel_circ_0011308	0	3.174521502	-5.1893	0.024275	0.049951
novel_circ_0015086	0	3.174521502	-5.1893	0.024275	0.049951
novel_circ_0015950	0	3.174521502	-5.1893	0.024275	0.049951
novel_circ_0019996	0	3.174521502	-5.1893	0.024275	0.049951
novel_circ_0013346	1.581256216	7.759382193	-2.3402	0.024303	0.049986

raw Ct data-circRNA-0000607

sample	Raw Cт	Cт Mean	
control-1	28.986166	28.96342468	GAPDH
control-1	27.96869659	28.96342468	GAPDH
control-1	29.93541336	28.96342468	GAPDH
control-2	27.99703979	27.13468361	GAPDH
control-2	26.42168617	27.13468361	GAPDH
control-2	26.98532677	27.13468361	GAPDH
control-3	24.32445526	24.02895927	GAPDH
control-3	23.98655128	24.02895927	GAPDH
control-3	23.77586937	24.02895927	GAPDH
control-4	24.01070595	24.58120155	GAPDH
control-4	24.95363426	24.58120155	GAPDH
control-4	24.77926636	24.58120155	GAPDH
control-5	27.71316719	28.22460938	GAPDH
control-5	28.46683121	28.22460938	GAPDH
control-5	28.49383354	28.22460938	GAPDH
control-6	23.99707031	23.90258789	GAPDH
control-6	23.96064949	23.90258789	GAPDH
control-6	23.75004387	23.90258789	GAPDH
control-7	25.62896347	26.07480049	GAPDH
control-7	25.98989105	26.07480049	GAPDH
control-7	26.60554886	26.07480049	GAPDH
control-8	23.97501373	24.74778175	GAPDH
control-8	24.88467979	24.74778175	GAPDH
control-8	25.38365364	24.74778175	GAPDH
stroke-1	22.81828117	23.25094604	GAPDH
stroke-1	23.6321888	23.25094604	GAPDH
stroke-1	23.30237007	23.25094604	GAPDH
stroke-2	24.24754715	24.11670876	GAPDH
stroke-2	23.96543312	24.11670876	GAPDH
stroke-2	24.13714218	24.11670876	GAPDH
stroke-3	24.44216728	23.7510891	GAPDH
stroke-3	21.98332214	23.7510891	GAPDH
stroke-3	24.82777214	23.7510891	GAPDH
stroke-4	21.99193001	21.82296753	GAPDH
stroke-4	21.22269249	21.82296753	GAPDH
stroke-4	22.254282	21.82296753	GAPDH
stroke-5	23.41270638	23.87949944	GAPDH
stroke-5	24.31710625	23.87949944	GAPDH
stroke-5	23.90868568	23.87949944	GAPDH
stroke-6	21.50953865	22.07132721	GAPDH
stroke-6	22.97553635	22.07132721	GAPDH
stroke-6	21.72891045	22.07132721	GAPDH
stroke-7	21.9685936	22.42323303	GAPDH
stroke-7	21.89948082	22.42323303	GAPDH
stroke-7	23.40161896	22.42323303	GAPDH
stroke-8	24.72301102	24.3835392	GAPDH
stroke-8	23.79771042	24.3835392	GAPDH
stroke-8	24.62989235	24.3835392	GAPDH
control-1	30.2010498	30.12251282	circ-0000607
control-1	29.85174179	30.12251282	circ-0000607
control-1	30.31474876	30.12251282	circ-0000607
control-2	29.86787224	30.4516468	circ-0000607
control-2	30.96028709	30.4516468	circ-0000607

control-2	30.52678108	30.4516468	circ-0000607
control-3	27.002388	26.98674965	circ-0000607
control-3	27.23257637	26.98674965	circ-0000607
control-3	26.72528267	26.98674965	circ-0000607
control-4	26.16671371	26.52879333	circ-0000607
control-4	27.34694672	26.52879333	circ-0000607
control-4	26.07271767	26.52879333	circ-0000607
control-5	29.00901794	29.44142151	circ-0000607
control-5	29.97493172	29.44142151	circ-0000607
control-5	29.34031105	29.44142151	circ-0000607
control-6	26.46099281	26.09449768	circ-0000607
control-6	25.42113495	26.09449768	circ-0000607
control-6	26.40136909	26.09449768	circ-0000607
control-7	27.76145554	27.7904911	circ-0000607
control-7	27.78826141	27.7904911	circ-0000607
control-7	27.82175255	27.7904911	circ-0000607
control-8	26.03160095	26.35666656	circ-0000607
control-8	26.59160995	26.35666656	circ-0000607
control-8	26.44678688	26.35666656	circ-0000607
stroke-1	24.6139946	24.2837677	circ-0000607
stroke-1	23.85692978	24.2837677	circ-0000607
stroke-1	24.38037491	24.2837677	circ-0000607
stroke-2	25.96882057	27.04850769	circ-0000607
stroke-2	27.71820831	27.04850769	circ-0000607
stroke-2	27.45849609	27.04850769	circ-0000607
stroke-3	28.25543785	28.05039215	circ-0000607
stroke-3	28.0123024	28.05039215	circ-0000607
stroke-3	27.8834362	28.05039215	circ-0000607
stroke-4	25.77251244	26.16079712	circ-0000607
stroke-4	26.86099815	26.16079712	circ-0000607
stroke-4	25.84887886	26.16079712	circ-0000607
stroke-5	27.9960022	26.85706139	circ-0000607
stroke-5	26.64691353	26.85706139	circ-0000607
stroke-5	25.92826271	26.85706139	circ-0000607
stroke-6	25.91727257	25.67337036	circ-0000607
stroke-6	25.12128258	25.67337036	circ-0000607
stroke-6	25.98155403	25.67337036	circ-0000607
stroke-7	24.96683693	25.62806511	circ-0000607
stroke-7	24.76462936	25.62806511	circ-0000607
stroke-7	27.15273094	25.62806511	circ-0000607
stroke-8	26.74677086	26.77631378	circ-0000607
stroke-8	26.7318821	26.77631378	circ-0000607
stroke-8	26.85028839	26.77631378	circ-0000607
control-9	27.53953171	28.13417053	GAPDH
control-9	28.65486717	28.13417053	GAPDH
control-9	28.20811653	28.13417053	GAPDH
control-10	26.54609299	26.59030151	GAPDH
control-10	26.84967422	26.59030151	GAPDH
control-10	26.37513542	26.59030151	GAPDH
control-11	25.05536079	24.33853912	GAPDH
control-11	23.45033836	24.33853912	GAPDH
control-11	24.50991631	24.33853912	GAPDH
control-12	24.7355423	24.47111702	GAPDH
control-12	24.09218979	24.47111702	GAPDH
control-12	24.58562279	24.47111702	GAPDH
control-13	27.95845222	28.37546539	GAPDH
control-13	28.39027214	28.37546539	GAPDH
control-13	28.77766991	28.37546539	GAPDH
control-14	23.43803215	24.06204033	GAPDH
control-14	23.77913475	24.06204033	GAPDH
control-14	24.96895409	24.06204033	GAPDH
control-15	25.81467628	25.26215172	GAPDH
control-15	24.80905724	25.26215172	GAPDH
control-15	25.16272354	25.26215172	GAPDH
control-16	24.40066528	24.31707001	GAPDH

control-16	24.26047897	24.31707001	GAPDH
control-16	24.29006767	24.31707001	GAPDH
stroke-9	22.51823997	22.15864372	GAPDH
stroke-9	22.14232445	22.15864372	GAPDH
stroke-9	21.81536484	22.15864372	GAPDH
stroke-10	24.56346893	24.30210304	GAPDH
stroke-10	23.81158829	24.30210304	GAPDH
stroke-10	24.53125191	24.30210304	GAPDH
stroke-11	24.77765274	24.3769989	GAPDH
stroke-11	24.04597473	24.3769989	GAPDH
stroke-11	24.30736732	24.3769989	GAPDH
stroke-12	22.65849876	22.55952263	GAPDH
stroke-12	21.888237	22.55952263	GAPDH
stroke-12	23.1318264	22.55952263	GAPDH
stroke-13	23.57981491	23.95546532	GAPDH
stroke-13	24.11337471	23.95546532	GAPDH
stroke-13	24.17320442	23.95546532	GAPDH
stroke-14	22.18286514	21.92886925	GAPDH
stroke-14	21.3039608	21.92886925	GAPDH
stroke-14	22.29978371	21.92886925	GAPDH
stroke-15	22.67212868	22.87604523	GAPDH
stroke-15	22.58248138	22.87604523	GAPDH
stroke-15	23.37352562	22.87604523	GAPDH
stroke-16	24.93684387	24.96379662	GAPDH
stroke-16	24.99489975	24.96379662	GAPDH
stroke-16	24.95964241	24.96379662	GAPDH
control-9	29.79476547	28.68559265	circ-0000607
control-9	28.9161396	28.68559265	circ-0000607
control-9	27.34587097	28.68559265	circ-0000607
control-10	30.24912453	30.37117004	circ-0000607
control-10	30.08897018	30.37117004	circ-0000607
control-10	30.77541924	30.37117004	circ-0000607
control-11	29.81713867	28.6261692	circ-0000607
control-11	28.21579361	28.6261692	circ-0000607
control-11	27.84558105	28.6261692	circ-0000607
control-12	27.64474678	27.66439629	circ-0000607
control-12	27.62210274	27.66439629	circ-0000607
control-12	27.72634125	27.66439629	circ-0000607
control-13	30.8066864	30.77066803	circ-0000607
control-13	31.57831573	30.77066803	circ-0000607
control-13	29.92700005	30.77066803	circ-0000607
control-14	27.18829536	27.44194603	circ-0000607
control-14	27.49446297	27.44194603	circ-0000607
control-14	27.64307976	27.44194603	circ-0000607
control-15	28.06015396	28.11866188	circ-0000607
control-15	28.44386292	28.11866188	circ-0000607
control-15	27.85197067	28.11866188	circ-0000607
control-16	27.93567276	27.87058449	circ-0000607
control-16	27.75416756	27.87058449	circ-0000607
control-16	27.92190933	27.87058449	circ-0000607
stroke-9	28.08120155	27.51510048	circ-0000607
stroke-9	27.15916443	27.51510048	circ-0000607
stroke-9	27.30493736	27.51510048	circ-0000607
stroke-10	28.92417336	28.55953789	circ-0000607
stroke-10	27.3516407	28.55953789	circ-0000607
stroke-10	29.40280151	28.55953789	circ-0000607
stroke-11	30.40820503	29.67177773	circ-0000607
stroke-11	29.67686081	29.67177773	circ-0000607
stroke-11	28.93027115	29.67177773	circ-0000607
stroke-12	27.23206902	28.00384331	circ-0000607
stroke-12	28.0997982	28.00384331	circ-0000607
stroke-12	28.67965698	28.00384331	circ-0000607
stroke-13	29.16504478	27.81465149	circ-0000607
stroke-13	27.65477943	27.81465149	circ-0000607
stroke-13	26.62412643	27.81465149	circ-0000607

stroke-14	26.91100121	26.65208626	circ-0000607
stroke-14	27.22218323	26.65208626	circ-0000607
stroke-14	25.82307434	26.65208626	circ-0000607
stroke-15	26.37581444	27.00505638	circ-0000607
stroke-15	27.12553787	27.00505638	circ-0000607
stroke-15	27.51381111	27.00505638	circ-0000607
stroke-16	29.12075806	29.09998131	circ-0000607
stroke-16	29.22530174	29.09998131	circ-0000607
stroke-16	28.95388031	29.09998131	circ-0000607
control-17	24.37089729	23.65840721	GAPDH
control-17	23.34911919	23.65840721	GAPDH
control-17	23.25520515	23.65840721	GAPDH
control-18	23.85230255	24.05810547	GAPDH
control-18	24.2588501	24.05810547	GAPDH
control-18	24.06316757	24.05810547	GAPDH
control-19	23.52091026	23.08618927	GAPDH
control-19	22.92596436	23.08618927	GAPDH
control-19	22.81169128	23.08618927	GAPDH
control-20	23.92247963	24.03737068	GAPDH
control-20	24.12704659	24.03737068	GAPDH
control-20	24.06259155	24.03737068	GAPDH
control-21	25.32445526	25.54207039	GAPDH
control-21	25.60078812	25.54207039	GAPDH
control-21	25.70096779	25.54207039	GAPDH
control-22	24.53731346	24.4416275	GAPDH
control-22	24.68003845	24.4416275	GAPDH
control-22	24.10752869	24.4416275	GAPDH
control-23	30.08338928	34.30607986	GAPDH
control-23	35.84223938	34.30607986	GAPDH
control-23	36.99261475	34.30607986	GAPDH
control-24	25.50982666	25.45705605	GAPDH
control-24	25.03460693	25.45705605	GAPDH
control-24	25.82674026	25.45705605	GAPDH
stroke-17	23	22.60113525	GAPDH
stroke-17	22.4994278	22.60113525	GAPDH
stroke-17	22.30397797	22.60113525	GAPDH
stroke-18	22.84053612	22.13555145	GAPDH
stroke-18	22.1178112	22.13555145	GAPDH
stroke-18	21.44830322	22.13555145	GAPDH
stroke-19	23.27849579	23.36006355	GAPDH
stroke-19	23.28265762	23.36006355	GAPDH
stroke-19	23.51904106	23.36006355	GAPDH
stroke-20	26.08442116	25.90619469	GAPDH
stroke-20	25.99759102	25.90619469	GAPDH
stroke-20	25.63656807	25.90619469	GAPDH
stroke-21	23.81067276	23.56395721	GAPDH
stroke-21	23.95981598	23.56395721	GAPDH
stroke-21	22.9213829	23.56395721	GAPDH
stroke-22	22.7163105	22.07079124	GAPDH
stroke-22	21.4705925	22.07079124	GAPDH
stroke-22	22.02547264	22.07079124	GAPDH
stroke-23	22.53122139	22.12565041	GAPDH
stroke-23	21.69670296	22.12565041	GAPDH
stroke-23	22.14902687	22.12565041	GAPDH
stroke-24	25.28538322	25.17610168	GAPDH
stroke-24	25.12193108	25.17610168	GAPDH
stroke-24	25.12099075	25.17610168	GAPDH
control-17	26.08292961	25.84122658	circ-0000607
control-17	26.94491577	25.84122658	circ-0000607
control-17	24.49583626	25.84122658	circ-0000607
control-18	26.46812248	25.55987549	circ-0000607
control-18	23.39419746	25.55987549	circ-0000607
control-18	26.81730843	25.55987549	circ-0000607
control-19	25.63464546	25.08820152	circ-0000607
control-19	26.68480682	25.08820152	circ-0000607

control-19	22.94515228	25.08820152	circ-0000607
control-20	26.65668106	26.79709435	circ-0000607
control-20	26.86416054	26.79709435	circ-0000607
control-20	26.87043953	26.79709435	circ-0000607
control-21	27.31864548	25.19190979	circ-0000607
control-21	23.79074287	25.19190979	circ-0000607
control-21	24.46634674	25.19190979	circ-0000607
control-22	26.63454437	26.64724731	circ-0000607
control-22	26.62876511	26.64724731	circ-0000607
control-22	26.67843056	26.64724731	circ-0000607
control-23	26.01900291	24.91068077	circ-0000607
control-23	26.65623856	24.91068077	circ-0000607
control-23	22.05679893	24.91068077	circ-0000607
control-24	27.06623459	27.12625694	circ-0000607
control-24	27.12368584	27.12625694	circ-0000607
control-24	27.1888504	27.12625694	circ-0000607
stroke-17	25.6360836	24.54557037	circ-0000607
stroke-17	20.94773865	24.54557037	circ-0000607
stroke-17	27.05288506	24.54557037	circ-0000607
stroke-18	27.26455688	26.56749916	circ-0000607
stroke-18	25.59686089	26.56749916	circ-0000607
stroke-18	26.84108353	26.56749916	circ-0000607
stroke-19	26.85122871	26.94378853	circ-0000607
stroke-19	26.94581985	26.94378853	circ-0000607
stroke-19	27.03432083	26.94378853	circ-0000607
stroke-20	27.28665924	27.15337372	circ-0000607
stroke-20	27.25465393	27.15337372	circ-0000607
stroke-20	26.91880608	27.15337372	circ-0000607
stroke-21	27.22332954	27.05043602	circ-0000607
stroke-21	26.91497803	27.05043602	circ-0000607
stroke-21	27.01300049	27.05043602	circ-0000607
stroke-22	26.92191887	26.83640862	circ-0000607
stroke-22	26.65605545	26.83640862	circ-0000607
stroke-22	26.93125153	26.83640862	circ-0000607
stroke-23	26.45792007	26.51346397	circ-0000607
stroke-23	26.50614548	26.51346397	circ-0000607
stroke-23	26.57632637	26.51346397	circ-0000607
stroke-24	27.09989929	26.748312	circ-0000607
stroke-24	27.0061245	26.748312	circ-0000607
stroke-24	26.13891411	26.748312	circ-0000607
control-25	25.09058762	25.78330421	GAPDH
control-25	26.45950699	25.78330421	GAPDH
control-25	25.79981422	25.78330421	GAPDH
control-26	25.61735535	25.61603546	GAPDH
control-26	25.59007454	25.61603546	GAPDH
control-26	25.64067841	25.61603546	GAPDH
control-27	24.85573387	25.14356995	GAPDH
control-27	25.42196655	25.14356995	GAPDH
control-27	25.1530056	25.14356995	GAPDH
control-28	24.89976501	25.32396698	GAPDH
control-28	25.32309341	25.32396698	GAPDH
control-28	25.7490406	25.32396698	GAPDH
control-29	26.63158798	26.85236549	GAPDH
control-29	26.92671967	26.85236549	GAPDH
control-29	26.99878502	26.85236549	GAPDH
control-30	25.79225922	25.69767761	GAPDH
control-30	25.60913086	25.69767761	GAPDH
control-30	25.69164467	25.69767761	GAPDH
control-31	25.68033981	25.92655373	GAPDH
control-31	25.97459984	25.92655373	GAPDH
control-31	26.12472153	25.92655373	GAPDH
control-32	27.30233383	27.23433495	GAPDH
control-32	27.28869057	27.23433495	GAPDH
control-32	27.11198235	27.23433495	GAPDH
stroke-25	23.66721344	24.02459717	GAPDH

stroke-25	24.14888	24.02459717	GAPDH
stroke-25	24.25769997	24.02459717	GAPDH
stroke-26	28.53954315	28.14641571	GAPDH
stroke-26	27.82084656	28.14641571	GAPDH
stroke-26	28.07885933	28.14641571	GAPDH
stroke-27	24.53043365	24.71434784	GAPDH
stroke-27	24.66077805	24.71434784	GAPDH
stroke-27	24.95183182	24.71434784	GAPDH
stroke-28	26.83901787	26.77449036	GAPDH
stroke-28	26.897089	26.77449036	GAPDH
stroke-28	26.58736229	26.77449036	GAPDH
stroke-29	24.98485947	25.11204529	GAPDH
stroke-29	25.23482323	25.11204529	GAPDH
stroke-29	25.11645508	25.11204529	GAPDH
stroke-30	24.8253212	23.73656654	GAPDH
stroke-30	23.14415169	23.73656654	GAPDH
stroke-30	23.24023056	23.73656654	GAPDH
stroke-31	23.63295746	23.68496132	GAPDH
stroke-31	23.33071899	23.68496132	GAPDH
stroke-31	24.0912056	23.68496132	GAPDH
stroke-32	25.65545464	25.71642113	GAPDH
stroke-32	25.8227787	25.71642113	GAPDH
stroke-32	25.67102623	25.71642113	GAPDH
control-25	28.17640877	28.43628693	circ-0000607
control-25	28.97157478	28.43628693	circ-0000607
control-25	28.16087723	28.43628693	circ-0000607
control-26	28.88146973	28.34592628	circ-0000607
control-26	28.09176064	28.34592628	circ-0000607
control-26	28.06455421	28.34592628	circ-0000607
control-27	28.01698685	28.22145081	circ-0000607
control-27	28.32868385	28.22145081	circ-0000607
control-27	28.31868362	28.22145081	circ-0000607
control-28	27.7677002	27.06225014	circ-0000607
control-28	27.12875175	27.06225014	circ-0000607
control-28	26.29029846	27.06225014	circ-0000607
control-29	29.37800217	29.61915398	circ-0000607
control-29	29.80021477	29.61915398	circ-0000607
control-29	29.67924309	29.61915398	circ-0000607
control-30	28.13575935	28.08354187	circ-0000607
control-30	27.95761299	28.08354187	circ-0000607
control-30	28.15725517	28.08354187	circ-0000607
control-31	28.28242493	28.51596642	circ-0000607
control-31	28.58515167	28.51596642	circ-0000607
control-31	28.68031693	28.51596642	circ-0000607
control-32	29.09939957	28.37998009	circ-0000607
control-32	27.01730728	28.37998009	circ-0000607
control-32	29.02323151	28.37998009	circ-0000607
stroke-25	26.92085648	27.01782417	circ-0000607
stroke-25	26.91500854	27.01782417	circ-0000607
stroke-25	27.21760941	27.01782417	circ-0000607
stroke-26	30.30896759	30.4726696	circ-0000607
stroke-26	30.54724693	30.4726696	circ-0000607
stroke-26	30.56179428	30.4726696	circ-0000607
stroke-27	26.89507675	27.0796299	circ-0000607
stroke-27	27.39301491	27.0796299	circ-0000607
stroke-27	26.95079422	27.0796299	circ-0000607
stroke-28	30.1125412	29.84586143	circ-0000607
stroke-28	29.47126198	29.84586143	circ-0000607
stroke-28	29.95377922	29.84586143	circ-0000607
stroke-29	27.34066391	27.41553307	circ-0000607
stroke-29	27.51224709	27.41553307	circ-0000607
stroke-29	27.39368439	27.41553307	circ-0000607
stroke-30	26.21185112	26.31738091	circ-0000607
stroke-30	26.2661972	26.31738091	circ-0000607
stroke-30	26.47409248	26.31738091	circ-0000607

stroke-31	25.87304497	26.08994865	circ-0000607	
stroke-31	26.46668816	26.08994865	circ-0000607	
stroke-31	25.93010712	26.08994865	circ-0000607	
stroke-32	28.64920807	27.4141407	circ-0000607	
stroke-32	28.16283798	27.4141407	circ-0000607	
stroke-32	25.43037987	27.4141407	circ-0000607	

sample	△CT		ΔΔCт	RQ
control-1	1.159088135	5.444320679	4.285232544	19.49770675
control-1	1.159088135	5.444320679	4.285232544	19.49770675
control-1	1.159088135	5.444320679	4.285232544	19.49770675
control-2	3.316962481	5.444320679	2.127358198	4.369166843
control-2	3.316962481	5.444320679	2.127358198	4.369166843
control-2	3.316962481	5.444320679	2.127358198	4.369166843
control-3	2.957790375	5.444320679	2.486530304	5.604284921
control-3	2.957790375	5.444320679	2.486530304	5.604284921
control-3	2.957790375	5.444320679	2.486530304	5.604284921
control-4	1.94759047	5.444320679	3.496730208	11.28809562
control-4	1.94759047	5.444320679	3.496730208	11.28809562
control-4	1.94759047	5.444320679	3.496730208	11.28809562
control-5	1.21680963	5.444320679	4.227511048	18.73301292
control-5	1.21680963	5.444320679	4.227511048	18.73301292
control-5	1.21680963	5.444320679	4.227511048	18.73301292
control-6	2.191910982	5.444320679	3.252409697	9.529560616
control-6	2.191910982	5.444320679	3.252409697	9.529560616
control-6	2.191910982	5.444320679	3.252409697	9.529560616
control-7	1.715688705	5.444320679	3.728631973	13.25653634
control-7	1.715688705	5.444320679	3.728631973	13.25653634
control-7	1.715688705	5.444320679	3.728631973	13.25653634
control-8	1.6088835	5.444320679	3.835437179	14.27518145
control-8	1.6088835	5.444320679	3.835437179	14.27518145
control-8	1.6088835	5.444320679	3.835437179	14.27518145
stroke-1	1.032819748	5.444320679	4.411500931	21.28110165
stroke-1	1.032819748	5.444320679	4.411500931	21.28110165
stroke-1	1.032819748	5.444320679	4.411500931	21.28110165
stroke-2	2.931800842	5.444320679	2.512519836	5.70615856
stroke-2	2.931800842	5.444320679	2.512519836	5.70615856
stroke-2	2.931800842	5.444320679	2.512519836	5.70615856
stroke-3	4.299304962	5.444320679	1.145015717	2.211485398
stroke-3	4.299304962	5.444320679	1.145015717	2.211485398
stroke-3	4.299304962	5.444320679	1.145015717	2.211485398
stroke-4	4.337828159	5.444320679	1.106492519	2.153215207
stroke-4	4.337828159	5.444320679	1.106492519	2.153215207
stroke-4	4.337828159	5.444320679	1.106492519	2.153215207
stroke-5	2.977560043	5.444320679	2.466760635	5.52801157
stroke-5	2.977560043	5.444320679	2.466760635	5.52801157
stroke-5	2.977560043	5.444320679	2.466760635	5.52801157
stroke-6	3.602041245	5.444320679	1.842279434	3.585761254
stroke-6	3.602041245	5.444320679	1.842279434	3.585761254
stroke-6	3.602041245	5.444320679	1.842279434	3.585761254
stroke-7	3.2048347	5.444320679	2.239485979	4.722287832
stroke-7	3.2048347	5.444320679	2.239485979	4.722287832
stroke-7	3.2048347	5.444320679	2.239485979	4.722287832
stroke-8	2.392775774	5.444320679	3.051544905	8.290993018
stroke-8	2.392775774	5.444320679	3.051544905	8.290993018
stroke-8	2.392775774	5.444320679	3.051544905	8.290993018
control-9	0.551420212	5.444320679	4.892900467	29.71048944
control-9	0.551420212	5.444320679	4.892900467	29.71048944
control-9	0.551420212	5.444320679	4.892900467	29.71048944
control-10	3.780870438	5.444320679	1.663450241	3.167731906
control-10	3.780870438	5.444320679	1.663450241	3.167731906
control-10	3.780870438	5.444320679	1.663450241	3.167731906
control-11	4.287632465	5.444320679	1.156688213	2.22945057
control-11	4.287632465	5.444320679	1.156688213	2.22945057
control-11	4.287632465	5.444320679	1.156688213	2.22945057

control-12	3.193278551	5.444320679	2.251042128	4.760265786
control-12	3.193278551	5.444320679	2.251042128	4.760265786
control-12	3.193278551	5.444320679	2.251042128	4.760265786
control-13	2.395202637	5.444320679	3.049118042	8.277057857
control-13	2.395202637	5.444320679	3.049118042	8.277057857
control-13	2.395202637	5.444320679	3.049118042	8.277057857
control-14	3.379905701	5.444320679	2.064414978	4.182643327
control-14	3.379905701	5.444320679	2.064414978	4.182643327
control-14	3.379905701	5.444320679	2.064414978	4.182643327
control-15	2.856510162	5.444320679	2.587810516	6.011856263
control-15	2.856510162	5.444320679	2.587810516	6.011856263
control-15	2.856510162	5.444320679	2.587810516	6.011856263
control-16	3.553512573	5.444320679	1.890808105	3.708428891
control-16	3.553512573	5.444320679	1.890808105	3.708428891
control-16	3.553512573	5.444320679	1.890808105	3.708428891
stroke-9	5.356458187	5.444320679	0.087862492	1.062794371
stroke-9	5.356458187	5.444320679	0.087862492	1.062794371
stroke-9	5.356458187	5.444320679	0.087862492	1.062794371
stroke-10	4.257435322	5.444320679	1.186885357	2.276607141
stroke-10	4.257435322	5.444320679	1.186885357	2.276607141
stroke-10	4.257435322	5.444320679	1.186885357	2.276607141
stroke-11	5.294780731	5.444320679	0.149539948	1.109215704
stroke-11	5.294780731	5.444320679	0.149539948	1.109215704
stroke-11	5.294780731	5.444320679	0.149539948	1.109215704
stroke-12	5.444320679	5.444320679	0	1
stroke-12	5.444320679	5.444320679	0	1
stroke-12	5.444320679	5.444320679	0	1
stroke-13	3.859185457	5.444320679	1.585135221	3.000359184
stroke-13	3.859185457	5.444320679	1.585135221	3.000359184
stroke-13	3.859185457	5.444320679	1.585135221	3.000359184
stroke-14	4.723216534	5.444320679	0.721104145	1.648443163
stroke-14	4.723216534	5.444320679	0.721104145	1.648443163
stroke-14	4.723216534	5.444320679	0.721104145	1.648443163
stroke-15	4.129009247	5.444320679	1.315311432	2.48856045
stroke-15	4.129009247	5.444320679	1.315311432	2.48856045
stroke-15	4.129009247	5.444320679	1.315311432	2.48856045
stroke-16	4.136184692	5.444320679	1.308135986	2.476213975
stroke-16	4.136184692	5.444320679	1.308135986	2.476213975
stroke-16	4.136184692	5.444320679	1.308135986	2.476213975
control-17	2.182820082	5.444320679	3.261500597	9.589799133
control-17	2.182820082	5.444320679	3.261500597	9.589799133
control-17	2.182820082	5.444320679	3.261500597	9.589799133
control-18	1.501769423	5.444320679	3.942551255	15.37539165
control-18	1.501769423	5.444320679	3.942551255	15.37539165
control-18	1.501769423	5.444320679	3.942551255	15.37539165
control-19	2.002012968	5.444320679	3.442307711	10.87020852
control-19	2.002012968	5.444320679	3.442307711	10.87020852
control-19	2.002012968	5.444320679	3.442307711	10.87020852
control-20	2.759721041	5.444320679	2.684599638	6.429023561
control-20	2.759721041	5.444320679	2.684599638	6.429023561
control-20	2.759721041	5.444320679	2.684599638	6.429023561
control-21	-0.350158691	5.444320679	5.79447937	55.50244302
control-21	-0.350158691	5.444320679	5.79447937	55.50244302
control-21	-0.350158691	5.444320679	5.79447937	55.50244302
control-22	2.205619812	5.444320679	3.238700867	9.439437339
control-22	2.205619812	5.444320679	3.238700867	9.439437339
control-22	2.205619812	5.444320679	3.238700867	9.439437339
control-23	-9.395401001	5.444320679	14.83972168	29322.52417
control-23	-9.395401001	5.444320679	14.83972168	29322.52417
control-23	-9.395401001	5.444320679	14.83972168	29322.52417
control-24	1.66919899	5.444320679	3.775121689	13.69067515
control-24	1.66919899	5.444320679	3.775121689	13.69067515
control-24	1.66919899	5.444320679	3.775121689	13.69067515
stroke-17	1.944433808	5.444320679	3.49988687	11.31282136
stroke-17	1.944433808	5.444320679	3.49988687	11.31282136

stroke-17	1.944433808	5.444320679	3.49988687	11.31282136
stroke-18	4.431950092	5.444320679	1.012370586	2.017223009
stroke-18	4.431950092	5.444320679	1.012370586	2.017223009
stroke-18	4.431950092	5.444320679	1.012370586	2.017223009
stroke-19	3.583724976	5.444320679	1.860595703	3.631575825
stroke-19	3.583724976	5.444320679	1.860595703	3.631575825
stroke-19	3.583724976	5.444320679	1.860595703	3.631575825
stroke-20	1.247179627	5.444320679	4.197141051	18.34278825
stroke-20	1.247179627	5.444320679	4.197141051	18.34278825
stroke-20	1.247179627	5.444320679	4.197141051	18.34278825
stroke-21	3.486478806	5.444320679	1.957841873	3.884804164
stroke-21	3.486478806	5.444320679	1.957841873	3.884804164
stroke-21	3.486478806	5.444320679	1.957841873	3.884804164
stroke-22	4.765616894	5.444320679	0.678703785	1.600700931
stroke-22	4.765616894	5.444320679	0.678703785	1.600700931
stroke-22	4.765616894	5.444320679	0.678703785	1.600700931
stroke-23	4.387813568	5.444320679	1.056507111	2.079889828
stroke-23	4.387813568	5.444320679	1.056507111	2.079889828
stroke-23	4.387813568	5.444320679	1.056507111	2.079889828
stroke-24	1.572210908	5.444320679	3.872109771	14.64270077
stroke-24	1.572210908	5.444320679	3.872109771	14.64270077
stroke-24	1.572210908	5.444320679	3.872109771	14.64270077
control-25	2.652983904	5.444320679	2.791336775	6.922709335
control-25	2.652983904	5.444320679	2.791336775	6.922709335
control-25	2.652983904	5.444320679	2.791336775	6.922709335
control-26	2.729892015	5.444320679	2.714428663	6.563333142
control-26	2.729892015	5.444320679	2.714428663	6.563333142
control-26	2.729892015	5.444320679	2.714428663	6.563333142
control-27	3.077882767	5.444320679	2.366437912	5.156663525
control-27	3.077882767	5.444320679	2.366437912	5.156663525
control-27	3.077882767	5.444320679	2.366437912	5.156663525
control-28	1.738283753	5.444320679	3.706036925	13.05053391
control-28	1.738283753	5.444320679	3.706036925	13.05053391
control-28	1.738283753	5.444320679	3.706036925	13.05053391
control-29	2.766789198	5.444320679	2.677531481	6.39760305
control-29	2.766789198	5.444320679	2.677531481	6.39760305
control-29	2.766789198	5.444320679	2.677531481	6.39760305
control-30	2.385864258	5.444320679	3.058456421	8.330807956
control-30	2.385864258	5.444320679	3.058456421	8.330807956
control-30	2.385864258	5.444320679	3.058456421	8.330807956
control-31	2.589410782	5.444320679	2.854909897	7.234583172
control-31	2.589410782	5.444320679	2.854909897	7.234583172
control-31	2.589410782	5.444320679	2.854909897	7.234583172
control-32	1.14564383	5.444320679	4.298676848	19.68025281
control-32	1.14564383	5.444320679	4.298676848	19.68025281
control-32	1.14564383	5.444320679	4.298676848	19.68025281
stroke-25	2.993227005	5.444320679	2.451093674	5.468304851
stroke-25	2.993227005	5.444320679	2.451093674	5.468304851
stroke-25	2.993227005	5.444320679	2.451093674	5.468304851
stroke-26	2.326253176	5.444320679	3.118067503	8.682241203
stroke-26	2.326253176	5.444320679	3.118067503	8.682241203
stroke-26	2.326253176	5.444320679	3.118067503	8.682241203
stroke-27	2.365280867	5.444320679	3.079039812	8.450518197
stroke-27	2.365280867	5.444320679	3.079039812	8.450518197
stroke-27	2.365280867	5.444320679	3.079039812	8.450518197
stroke-28	3.071371078	5.444320679	2.3729496	5.179991032
stroke-28	3.071371078	5.444320679	2.3729496	5.179991032
stroke-28	3.071371078	5.444320679	2.3729496	5.179991032
stroke-29	2.30348587	5.444320679	3.140834808	8.820343299
stroke-29	2.30348587	5.444320679	3.140834808	8.820343299
stroke-29	2.30348587	5.444320679	3.140834808	8.820343299
stroke-30	2.580812454	5.444320679	2.863508224	7.277829356
stroke-30	2.580812454	5.444320679	2.863508224	7.277829356
stroke-30	2.580812454	5.444320679	2.863508224	7.277829356
stroke-31	2.404986143	5.444320679	3.039334536	8.221117627

stroke-31	2.404986143	5.444320679	3.039334536	8.221117627
stroke-31	2.404986143	5.444320679	3.039334536	8.221117627
stroke-32	1.697722077	5.444320679	3.746598601	13.42265911
stroke-32	1.697722077	5.444320679	3.746598601	13.42265911
stroke-32	1.697722077	5.444320679	3.746598601	13.42265911

sample	RQ	sample	RQ
control-1	19.49770675	stroke-1	21.28110165
control-2	4.369166843	stroke-2	5.70615856
control-3	5.604284921	stroke-3	2.211485398
control-4	11.28809562	stroke-4	2.153215207
control-5	18.73301292	stroke-5	5.52801157
control-6	9.529560616	stroke-6	3.585761254
control-7	13.25653634	stroke-7	4.722287832
control-8	14.27518145	stroke-8	8.290993018
control-9	29.71048944	stroke-9	1.062794371
control-10	3.167731906	stroke-10	2.276607141
control-11	2.22945057	stroke-11	1.109215704
control-12	4.760265786	stroke-12	1
control-13	8.277057857	stroke-13	3.000359184
control-14	4.182643327	stroke-14	1.648443163
control-15	6.011856263	stroke-15	2.48856045
control-16	3.708428891	stroke-16	2.476213975
control-17	9.589799133	stroke-17	11.31282136
control-18	15.37539165	stroke-18	2.017223009
control-19	10.87020852	stroke-19	3.631575825
control-20	6.429023561	stroke-20	18.34278825
control-21	55.50244302	stroke-21	3.884804164
control-22	9.439437339	stroke-22	1.600700931
control-23	29322.5241668528 (29.3)	stroke-23	2.079889828
control-24	13.69067515	stroke-24	14.64270077
control-25	6.922709335	stroke-25	5.468304851
control-26	6.563333142	stroke-26	8.682241203
control-27	5.156663525	stroke-27	8.450518197
control-28	13.05053391	stroke-28	5.179991032
control-29	6.39760305	stroke-29	8.820343299
control-30	8.330807956	stroke-30	7.277829356
control-31	7.234583172	stroke-31	8.221117627
control-32	19.68025281	stroke-32	13.42265911

raw Ct data circRNA-0002465

Sample Name	Raw Cᴛ	Cᴛ Mean	
control-1	28.22517967	28.5207653	GAPDH
control-1	28.78348351	28.5207653	GAPDH
control-1	28.55363274	28.5207653	GAPDH
control-2	28.53512192	28.58434296	GAPDH
control-2	28.67719078	28.58434296	GAPDH
control-2	28.54071617	28.58434296	GAPDH
control-3	24.60255814	24.6583004	GAPDH
control-3	24.68700218	24.6583004	GAPDH
control-3	24.68533897	24.6583004	GAPDH
control-4	24.88133621	24.9044857	GAPDH
control-4	24.88339806	24.9044857	GAPDH
control-4	24.94872665	24.9044857	GAPDH
control-5	26.07564163	25.99196815	GAPDH
control-5	25.96985626	25.99196815	GAPDH
control-5	25.93041039	25.99196815	GAPDH
control-6	24.87948418	24.92822075	GAPDH
control-6	25.03196144	24.92822075	GAPDH
control-6	24.87321091	24.92822075	GAPDH
control-7	24.938797	25.27075768	GAPDH
control-7	25.3934288	25.27075768	GAPDH

control-7	25.48004532	25.27075768	GAPDH
control-8	24.70462418	24.73781395	GAPDH
control-8	24.66288757	24.73781395	GAPDH
control-8	24.84592438	24.73781395	GAPDH
stroke-1	23.93505478	24.06849098	GAPDH
stroke-1	24.06428909	24.06849098	GAPDH
stroke-1	24.20613098	24.06849098	GAPDH
stroke-2	25.20811081	25.35446739	GAPDH
stroke-2	25.35016632	25.35446739	GAPDH
stroke-2	25.50512886	25.35446739	GAPDH
stroke-3	23.91085052	24.03520775	GAPDH
stroke-3	24.16567802	24.03520775	GAPDH
stroke-3	24.02909088	24.03520775	GAPDH
stroke-4	23.28930473	23.68898201	GAPDH
stroke-4	23.87956429	23.68898201	GAPDH
stroke-4	23.8980751	23.68898201	GAPDH
stroke-5	23.8413868	24.05538177	GAPDH
stroke-5	24.12804222	24.05538177	GAPDH
stroke-5	24.1967144	24.05538177	GAPDH
stroke-6	24.98008919	24.82170296	GAPDH
stroke-6	24.84412193	24.82170296	GAPDH
stroke-6	24.64089775	24.82170296	GAPDH
stroke-7	23.23210526	23.64855385	GAPDH
stroke-7	23.74567032	23.64855385	GAPDH
stroke-7	23.96788406	23.64855385	GAPDH
stroke-8	23.71293068	23.82270813	GAPDH
stroke-8	23.80846214	23.82270813	GAPDH
stroke-8	23.94673538	23.82270813	GAPDH
control-1	32.56254959	31.86647987	circ-0002465
control-1	31.12167168	31.86647987	circ-0002465
control-1	31.91522026	31.86647987	circ-0002465
control-2	31.7302475	31.79043579	circ-0002465
control-2	31.4820919	31.79043579	circ-0002465
control-2	32.15896606	31.79043579	circ-0002465
control-3	30.06734467	29.73313904	circ-0002465
control-3	29.22234917	29.73313904	circ-0002465
control-3	29.90972328	29.73313904	circ-0002465
control-4	27.47392845	27.44553947	circ-0002465
control-4	27.34608078	27.44553947	circ-0002465
control-4	27.51660728	27.44553947	circ-0002465
control-5	30.56390572	30.46040535	circ-0002465
control-5	30.57001686	30.46040535	circ-0002465
control-5	30.24729919	30.46040535	circ-0002465
control-6	29.00938034	29.14421844	circ-0002465
control-6	29.16273117	29.14421844	circ-0002465
control-6	29.26054382	29.14421844	circ-0002465
control-7	29.80199623	29.8035717	circ-0002465
control-7	30.09585953	29.8035717	circ-0002465
control-7	29.51285934	29.8035717	circ-0002465
control-8	29.44866753	29.22708893	circ-0002465
control-8	29.07068253	29.22708893	circ-0002465
control-8	29.16191483	29.22708893	circ-0002465
stroke-1	28.58267593	28.66102028	circ-0002465
stroke-1	28.76257706	28.66102028	circ-0002465
stroke-1	28.63781166	28.66102028	circ-0002465
stroke-2	31.43391991	31.13836479	circ-0002465
stroke-2	31.16127396	31.13836479	circ-0002465
stroke-2	30.81989861	31.13836479	circ-0002465
stroke-3	27.09321976	27.08148766	circ-0002465
stroke-3	27.10632515	27.08148766	circ-0002465
stroke-3	27.04491425	27.08148766	circ-0002465
stroke-4	28.97231674	28.90931129	circ-0002465
stroke-4	28.94058609	28.90931129	circ-0002465
stroke-4	28.81503868	28.90931129	circ-0002465
stroke-5	28.9219265	28.73053741	circ-0002465

stroke-5	28.53737259	28.73053741	circ-0002465
stroke-5	28.73231506	28.73053741	circ-0002465
stroke-6	29.31085014	29.22802734	circ-0002465
stroke-6	29.4238472	29.22802734	circ-0002465
stroke-6	28.94938087	29.22802734	circ-0002465
stroke-7	28.36186028	28.46089363	circ-0002465
stroke-7	28.50704002	28.46089363	circ-0002465
stroke-7	28.51378059	28.46089363	circ-0002465
stroke-8	28.05480576	27.00875282	circ-0002465
stroke-8	28.20303917	27.00875282	circ-0002465
stroke-8	24.76842117	27.00875282	circ-0002465
control-9	28.24897766	28.61803246	GAPDH
control-9	28.63478661	28.61803246	GAPDH
control-9	28.9703331	28.61803246	GAPDH
control-10	28.25745773	28.01657677	GAPDH
control-10	27.79316902	28.01657677	GAPDH
control-10	27.99910164	28.01657677	GAPDH
control-11	24.97808266	25.19816017	GAPDH
control-11	25.34111404	25.19816017	GAPDH
control-11	25.27528572	25.19816017	GAPDH
control-12	24.94738579	24.6295166	GAPDH
control-12	24.21150589	24.6295166	GAPDH
control-12	24.72965622	24.6295166	GAPDH
control-13	29.87628746	29.13039398	GAPDH
control-13	28.92920303	29.13039398	GAPDH
control-13	28.58568764	29.13039398	GAPDH
control-14	23.95876884	24.14717293	GAPDH
control-14	23.99143791	24.14717293	GAPDH
control-14	24.49131012	24.14717293	GAPDH
control-15	24.61626053	25.06131935	GAPDH
control-15	25.21447182	25.06131935	GAPDH
control-15	25.35322189	25.06131935	GAPDH
control-16	25.26622963	25.04005241	GAPDH
control-16	24.3350544	25.04005241	GAPDH
control-16	25.51887131	25.04005241	GAPDH
stroke-9	23.60490608	22.87086487	GAPDH
stroke-9	22.39182663	22.87086487	GAPDH
stroke-9	22.6158638	22.87086487	GAPDH
stroke-10	23.67581177	24.35485649	GAPDH
stroke-10	25.41589355	24.35485649	GAPDH
stroke-10	23.97286415	24.35485649	GAPDH
stroke-11	23.23627853	23.74555969	GAPDH
stroke-11	24.85750389	23.74555969	GAPDH
stroke-11	23.14290237	23.74555969	GAPDH
stroke-12	23.24978828	23.20983696	GAPDH
stroke-12	23.83935356	23.20983696	GAPDH
stroke-12	22.54037094	23.20983696	GAPDH
stroke-13	24.32772064	24.08715248	GAPDH
stroke-13	23.33868027	24.08715248	GAPDH
stroke-13	24.59505844	24.08715248	GAPDH
stroke-14	22.90438843	22.98325539	GAPDH
stroke-14	23.27542686	22.98325539	GAPDH
stroke-14	22.76995659	22.98325539	GAPDH
stroke-15	22.63058853	23.31741905	GAPDH
stroke-15	24.12521553	23.31741905	GAPDH
stroke-15	23.19645119	23.31741905	GAPDH
stroke-16	25.02811432	25.08237267	GAPDH
stroke-16	25.11139297	25.08237267	GAPDH
stroke-16	25.10761261	25.08237267	GAPDH
control-9	31.45966911	31.48971558	circ-0002465
control-9	31.14132309	31.48971558	circ-0002465
control-9	31.86815071	31.48971558	circ-0002465
control-10	30.9251442	31.16489601	circ-0002465
control-10	31.57998657	31.16489601	circ-0002465
control-10	30.98956108	31.16489601	circ-0002465

control-11	29.98975182	30.38703728	circ-0002465
control-11	30.20396042	30.38703728	circ-0002465
control-11	30.96739578	30.38703728	circ-0002465
control-12	28.88368988	28.81193542	circ-0002465
control-12	28.80916595	28.81193542	circ-0002465
control-12	28.74294662	28.81193542	circ-0002465
control-13	32.39952469	31.89770508	circ-0002465
control-13	31.95591927	31.89770508	circ-0002465
control-13	31.337677	31.89770508	circ-0002465
control-14	29.39094734	29.10651588	circ-0002465
control-14	28.59114075	29.10651588	circ-0002465
control-14	29.33746338	29.10651588	circ-0002465
control-15	29.78352356	29.67912483	circ-0002465
control-15	29.53899002	29.67912483	circ-0002465
control-15	29.71486473	29.67912483	circ-0002465
control-16	30.26912308	30.44482422	circ-0002465
control-16	30.65041161	30.44482422	circ-0002465
control-16	30.4149437	30.44482422	circ-0002465
stroke-9	27.9244175	26.85712242	circ-0002465
stroke-9	26.1763382	26.85712242	circ-0002465
stroke-9	26.47060776	26.85712242	circ-0002465
stroke-10	29.37658501	28.92365074	circ-0002465
stroke-10	29.45667648	28.92365074	circ-0002465
stroke-10	27.93769073	28.92365074	circ-0002465
stroke-11	30.81083679	30.54960632	circ-0002465
stroke-11	30.84845161	30.54960632	circ-0002465
stroke-11	29.98952866	30.54960632	circ-0002465
stroke-12	28.30636978	29.03059959	circ-0002465
stroke-12	29.76253128	29.03059959	circ-0002465
stroke-12	29.02289581	29.03059959	circ-0002465
stroke-13	29.00582695	29.04254723	circ-0002465
stroke-13	29.1603241	29.04254723	circ-0002465
stroke-13	28.96149063	29.04254723	circ-0002465
stroke-14	27.57000542	27.57513237	circ-0002465
stroke-14	27.51561546	27.57513237	circ-0002465
stroke-14	27.63977242	27.57513237	circ-0002465
stroke-15	28.3933754	28.52927208	circ-0002465
stroke-15	28.94805527	28.52927208	circ-0002465
stroke-15	28.24637985	28.52927208	circ-0002465
stroke-16	29.96845055	30.08955574	circ-0002465
stroke-16	29.92323112	30.08955574	circ-0002465
stroke-16	30.37698936	30.08955574	circ-0002465
control-17	28.94908714	29.19761086	GAPDH
control-17	29.33703041	29.19761086	GAPDH
control-17	29.30671883	29.19761086	GAPDH
control-18	27.77310371	27.81768799	GAPDH
control-18	28.18780518	27.81768799	GAPDH
control-18	27.49215698	27.81768799	GAPDH
control-19	25.48110962	25.3298645	GAPDH
control-19	25.56259155	25.3298645	GAPDH
control-19	24.94589424	25.3298645	GAPDH
control-20	24.25027466	24.46928406	GAPDH
control-20	24.35326004	24.46928406	GAPDH
control-20	24.80431747	24.46928406	GAPDH
control-21	28.83850479	29.44898033	GAPDH
control-21	29.55249214	29.44898033	GAPDH
control-21	29.95594215	29.44898033	GAPDH
control-22	24.11787415	23.97958374	GAPDH
control-22	24.5291481	23.97958374	GAPDH
control-22	23.29173279	23.97958374	GAPDH
control-23	25.42292595	25.70676994	GAPDH
control-23	25.8006916	25.70676994	GAPDH
control-23	25.89668846	25.70676994	GAPDH
control-24	24.67227364	25.03015327	GAPDH
control-24	25.13502121	25.03015327	GAPDH

control-24	25.28316689	25.03015327	GAPDH
stroke-17	23.73663902	23.6374054	GAPDH
stroke-17	23.37400818	23.6374054	GAPDH
stroke-17	23.80156708	23.6374054	GAPDH
stroke-18	25.80944824	25.36976051	GAPDH
stroke-18	25.16739082	25.36976051	GAPDH
stroke-18	25.13244247	25.36976051	GAPDH
stroke-19	25.1457386	25.50319862	GAPDH
stroke-19	25.46788025	25.50319862	GAPDH
stroke-19	25.89597893	25.50319862	GAPDH
stroke-20	23.80775452	23.75936317	GAPDH
stroke-20	24.3048172	23.75936317	GAPDH
stroke-20	23.1655159	23.75936317	GAPDH
stroke-21	24.44375801	24.54026031	GAPDH
stroke-21	24.70052719	24.54026031	GAPDH
stroke-21	24.47649956	24.54026031	GAPDH
stroke-22	23.68344688	22.18631554	GAPDH
stroke-22	21.7127037	22.18631554	GAPDH
stroke-22	21.16279602	22.18631554	GAPDH
stroke-23	23.90446663	23.97942352	GAPDH
stroke-23	23.16567993	23.97942352	GAPDH
stroke-23	24.8681221	23.97942352	GAPDH
stroke-24	24.87950325	25.03767967	GAPDH
stroke-24	25.12272453	25.03767967	GAPDH
stroke-24	25.11081123	25.03767967	GAPDH
control-17	33.19500732	33.03007126	circ-0002465
control-17	31.63611603	33.03007126	circ-0002465
control-17	34.25908279	33.03007126	circ-0002465
control-18	31.93916893	31.77598	circ-0002465
control-18	31.96018028	31.77598	circ-0002465
control-18	31.42859459	31.77598	circ-0002465
control-19	28.21405602	28.97886086	circ-0002465
control-19	29.88322258	28.97886086	circ-0002465
control-19	28.83930206	28.97886086	circ-0002465
control-20	29.51506805	29.45559692	circ-0002465
control-20	28.98643494	29.45559692	circ-0002465
control-20	29.86528397	29.45559692	circ-0002465
control-21	33.74800873	34.29346848	circ-0002465
control-21	34.00082779	34.29346848	circ-0002465
control-21	35.13157272	34.29346848	circ-0002465
control-22	28.24772644	28.91924095	circ-0002465
control-22	29.47133446	28.91924095	circ-0002465
control-22	29.03866386	28.91924095	circ-0002465
control-23	29.12859344	29.40195656	circ-0002465
control-23	29.25438118	29.40195656	circ-0002465
control-23	29.82289314	29.40195656	circ-0002465
control-24	28.22966957	28.29116631	circ-0002465
control-24	27.88504982	28.29116631	circ-0002465
control-24	28.75877953	28.29116631	circ-0002465
stroke-17	29.08116531	28.89151573	circ-0002465
stroke-17	28.8964901	28.89151573	circ-0002465
stroke-17	28.69688988	28.89151573	circ-0002465
stroke-18	30.37239456	31.37583351	circ-0002465
stroke-18	31.32005882	31.37583351	circ-0002465
stroke-18	32.43505478	31.37583351	circ-0002465
stroke-19	29.32694054	30.13388062	circ-0002465
stroke-19	30.83299637	30.13388062	circ-0002465
stroke-19	30.24170113	30.13388062	circ-0002465
stroke-20	29.62601471	28.71315193	circ-0002465
stroke-20	28.10028458	28.71315193	circ-0002465
stroke-20	28.41316032	28.71315193	circ-0002465
stroke-21	28.2462368	28.50326538	circ-0002465
stroke-21	28.33555412	28.50326538	circ-0002465
stroke-21	28.92800522	28.50326538	circ-0002465
stroke-22	25.73388672	26.64194489	circ-0002465

stroke-22	26.6782608	26.64194489	circ-0002465
stroke-22	27.51368523	26.64194489	circ-0002465
stroke-23	28.60551071	28.1728611	circ-0002465
stroke-23	28.55725288	28.1728611	circ-0002465
stroke-23	27.35581779	28.1728611	circ-0002465
stroke-24	29.12100983	29.1842289	circ-0002465
stroke-24	29.37849236	29.1842289	circ-0002465
stroke-24	29.05319023	29.1842289	circ-0002465
control-25	25.30481911	25.44786835	GAPDH
control-25	25.43247986	25.44786835	GAPDH
control-25	25.60630417	25.44786835	GAPDH
control-26	23.60297394	23.56755066	GAPDH
control-26	23.43074608	23.56755066	GAPDH
control-26	23.66893196	23.56755066	GAPDH
control-27	24.52764893	24.51243019	GAPDH
control-27	24.28105354	24.51243019	GAPDH
control-27	24.72859192	24.51243019	GAPDH
control-28	25.04073906	24.99892426	GAPDH
control-28	24.93611336	24.99892426	GAPDH
control-28	25.01992226	24.99892426	GAPDH
control-29	24.27246666	24.5294342	GAPDH
control-29	24.70700073	24.5294342	GAPDH
control-29	24.60883522	24.5294342	GAPDH
control-30	25.35090446	25.15704536	GAPDH
control-30	25.16349602	25.15704536	GAPDH
control-30	24.95673752	25.15704536	GAPDH
control-31	23.88335037	24.22380638	GAPDH
control-31	24.38490105	24.22380638	GAPDH
control-31	24.40316772	24.22380638	GAPDH
control-32	24.8608532	24.94484138	GAPDH
control-32	24.97536469	24.94484138	GAPDH
control-32	24.99830437	24.94484138	GAPDH
stroke-25	23.70881462	24.02713585	GAPDH
stroke-25	24.1415081	24.02713585	GAPDH
stroke-25	24.23108101	24.02713585	GAPDH
stroke-26	22.9226265	22.45311928	GAPDH
stroke-26	22.34535599	22.45311928	GAPDH
stroke-26	22.09137726	22.45311928	GAPDH
stroke-27	25.49575806	25.71344566	GAPDH
stroke-27	25.8449688	25.71344566	GAPDH
stroke-27	25.79960442	25.71344566	GAPDH
stroke-28	22.50871658	22.60425758	GAPDH
stroke-28	22.95963287	22.60425758	GAPDH
stroke-28	22.34441948	22.60425758	GAPDH
stroke-29	24.73519897	25.11142921	GAPDH
stroke-29	24.81212425	25.11142921	GAPDH
stroke-29	25.78696632	25.11142921	GAPDH
stroke-30	23.04039192	22.93328857	GAPDH
stroke-30	23.05605698	22.93328857	GAPDH
stroke-30	22.70341873	22.93328857	GAPDH
stroke-31	21.96428108	22.10832405	GAPDH
stroke-31	22.1443882	22.10832405	GAPDH
stroke-31	22.21630669	22.10832405	GAPDH
stroke-32	20.96019173	22.18644142	GAPDH
stroke-32	22.619627	22.18644142	GAPDH
stroke-32	22.97950935	22.18644142	GAPDH
control-25	27.86724091	26.90261078	circ-0002465
control-25	26.38318634	26.90261078	circ-0002465
control-25	26.45740318	26.90261078	circ-0002465
control-26	24.45399284	24.4001236	circ-0002465
control-26	24.22079277	24.4001236	circ-0002465
control-26	24.52558517	24.4001236	circ-0002465
control-27	26.99706841	27.32739449	circ-0002465
control-27	27.20311165	27.32739449	circ-0002465
control-27	27.7820015	27.32739449	circ-0002465

control-28	27.32508659	26.9041748	circ-0002465
control-28	26.89035225	26.9041748	circ-0002465
control-28	26.49708176	26.9041748	circ-0002465
control-29	27.77741814	27.0975666	circ-0002465
control-29	26.73068619	27.0975666	circ-0002465
control-29	26.7845974	27.0975666	circ-0002465
control-30	27.13654709	27.03691673	circ-0002465
control-30	27.13302231	27.03691673	circ-0002465
control-30	26.84117317	27.03691673	circ-0002465
control-31	26.87272644	26.93076515	circ-0002465
control-31	26.98042297	26.93076515	circ-0002465
control-31	26.93914795	26.93076515	circ-0002465
control-32	26.52950859	26.41366005	circ-0002465
control-32	26.51651955	26.41366005	circ-0002465
control-32	26.1949501	26.41366005	circ-0002465
stroke-25	28.66191673	28.31859398	circ-0002465
stroke-25	27.89312553	28.31859398	circ-0002465
stroke-25	28.40073395	28.31859398	circ-0002465
stroke-26	26.37397194	26.60219574	circ-0002465
stroke-26	26.68183708	26.60219574	circ-0002465
stroke-26	26.75078201	26.60219574	circ-0002465
stroke-27	34.16732025	32.4129982	circ-0002465
stroke-27	36.09153748	32.4129982	circ-0002465
stroke-27	26.9801445	32.4129982	circ-0002465
stroke-28	25.57251358	25.5241642	circ-0002465
stroke-28	25.36157036	25.5241642	circ-0002465
stroke-28	25.63841248	25.5241642	circ-0002465
stroke-29	26.71012306	26.70905113	circ-0002465
stroke-29	26.66895485	26.70905113	circ-0002465
stroke-29	26.74807358	26.70905113	circ-0002465
stroke-30	27.10251427	26.88416481	circ-0002465
stroke-30	27.01667786	26.88416481	circ-0002465
stroke-30	26.53330612	26.88416481	circ-0002465
stroke-31	24.62226295	24.26831818	circ-0002465
stroke-31	23.29580879	24.26831818	circ-0002465
stroke-31	24.88688087	24.26831818	circ-0002465
stroke-32	24.11989403	24.8059864	circ-0002465
stroke-32	25.12437057	24.8059864	circ-0002465
stroke-32	25.17369652	24.8059864	circ-0002465

sample	△CT		ΔΔCт	RQ
control-1	3.345715284	6.804044247	3.458328962	10.99159588
control-1	3.345715284	6.804044247	3.458328962	10.99159588
control-1	3.345715284	6.804044247	3.458328962	10.99159588
control-2	3.206092119	6.804044247	3.597952127	12.10853254
control-2	3.206092119	6.804044247	3.597952127	12.10853254
control-2	3.206092119	6.804044247	3.597952127	12.10853254
control-3	5.074839115	6.804044247	1.729205132	3.315450996
control-3	5.074839115	6.804044247	1.729205132	3.315450996
control-3	5.074839115	6.804044247	1.729205132	3.315450996
control-4	2.541051865	6.804044247	4.262992382	19.19944074
control-4	2.541051865	6.804044247	4.262992382	19.19944074
control-4	2.541051865	6.804044247	4.262992382	19.19944074
control-5	4.468437672	6.804044247	2.335606575	5.047631444
control-5	4.468437672	6.804044247	2.335606575	5.047631444
control-5	4.468437672	6.804044247	2.335606575	5.047631444
control-6	4.215999603	6.804044247	2.588044643	6.012831973
control-6	4.215999603	6.804044247	2.588044643	6.012831973
control-6	4.215999603	6.804044247	2.588044643	6.012831973
control-7	4.532814503	6.804044247	2.271229744	4.827344355
control-7	4.532814503	6.804044247	2.271229744	4.827344355
control-7	4.532814503	6.804044247	2.271229744	4.827344355
control-8	4.489276409	6.804044247	2.314767838	4.975245918

control-8	4.489276409	6.804044247	2.314767838	4.975245918
control-8	4.489276409	6.804044247	2.314767838	4.975245918
stroke-1	4.592529774	6.804044247	2.211514473	4.631612232
stroke-1	4.592529774	6.804044247	2.211514473	4.631612232
stroke-1	4.592529774	6.804044247	2.211514473	4.631612232
stroke-2	5.783895493	6.804044247	1.020148754	2.028128066
stroke-2	5.783895493	6.804044247	1.020148754	2.028128066
stroke-2	5.783895493	6.804044247	1.020148754	2.028128066
stroke-3	3.046279907	6.804044247	3.757764339	13.52694683
stroke-3	3.046279907	6.804044247	3.757764339	13.52694683
stroke-3	3.046279907	6.804044247	3.757764339	13.52694683
stroke-4	5.220332623	6.804044247	1.583711624	2.997400003
stroke-4	5.220332623	6.804044247	1.583711624	2.997400003
stroke-4	5.220332623	6.804044247	1.583711624	2.997400003
stroke-5	4.67515707	6.804044247	2.128887177	4.373799771
stroke-5	4.67515707	6.804044247	2.128887177	4.373799771
stroke-5	4.67515707	6.804044247	2.128887177	4.373799771
stroke-6	4.406322956	6.804044247	2.397721291	5.269701673
stroke-6	4.406322956	6.804044247	2.397721291	5.269701673
stroke-6	4.406322956	6.804044247	2.397721291	5.269701673
stroke-7	4.81234026	6.804044247	1.991703987	3.977064575
stroke-7	4.81234026	6.804044247	1.991703987	3.977064575
stroke-7	4.81234026	6.804044247	1.991703987	3.977064575
stroke-8	3.186045885	6.804044247	3.617998362	12.27795482
stroke-8	3.186045885	6.804044247	3.617998362	12.27795482
stroke-8	3.186045885	6.804044247	3.617998362	12.27795482
control9	2.871681929	6.804044247	3.932362318	15.26718652
control9	2.871681929	6.804044247	3.932362318	15.26718652
control9	2.871681929	6.804044247	3.932362318	15.26718652
control 10	3.148321152	6.804044247	3.655723095	12.60324293
control 10	3.148321152	6.804044247	3.655723095	12.60324293
control 10	3.148321152	6.804044247	3.655723095	12.60324293
control11	5.188875198	6.804044247	1.615169048	3.063474937
control11	5.188875198	6.804044247	1.615169048	3.063474937
control11	5.188875198	6.804044247	1.615169048	3.063474937
control 12	4.182418346	6.804044247	2.6216259	6.154432791
control 12	4.182418346	6.804044247	2.6216259	6.154432791
control 12	4.182418346	6.804044247	2.6216259	6.154432791
control 13	2.767314196	6.804044247	4.036730051	16.41257901
control 13	2.767314196	6.804044247	4.036730051	16.41257901
control 13	2.767314196	6.804044247	4.036730051	16.41257901
control 14	4.959344864	6.804044247	1.844699383	3.591780987
control 14	4.959344864	6.804044247	1.844699383	3.591780987
control 14	4.959344864	6.804044247	1.844699383	3.591780987
control 15	4.617807865	6.804044247	2.186236382	4.551166546
control 15	4.617807865	6.804044247	2.186236382	4.551166546
control 15	4.617807865	6.804044247	2.186236382	4.551166546
control 16	5.404774189	6.804044247	1.399270058	2.63768093
control 16	5.404774189	6.804044247	1.399270058	2.63768093
control 16	5.404774189	6.804044247	1.399270058	2.63768093
stroke-9	3.986255646	6.804044247	2.817788601	7.050808028
stroke-9	3.986255646	6.804044247	2.817788601	7.050808028
stroke-9	3.986255646	6.804044247	2.817788601	7.050808028
stroke-10	4.56879425	6.804044247	2.235249996	4.708442777
stroke-10	4.56879425	6.804044247	2.235249996	4.708442777
stroke-10	4.56879425	6.804044247	2.235249996	4.708442777
stroke-11	6.804044247	6.804044247	0	1
stroke-11	6.804044247	6.804044247	0	1
stroke-11	6.804044247	6.804044247	0	1
stroke-12	5.820761204	6.804044247	0.983283043	1.976959125
stroke-12	5.820761204	6.804044247	0.983283043	1.976959125
stroke-12	5.820761204	6.804044247	0.983283043	1.976959125
stroke-13	4.955394268	6.804044247	1.848649979	3.601629999
stroke-13	4.955394268	6.804044247	1.848649979	3.601629999
stroke-13	4.955394268	6.804044247	1.848649979	3.601629999

stroke-14	4.591873646	6.804044247	2.212170601	4.633719137
stroke-14	4.591873646	6.804044247	2.212170601	4.633719137
stroke-14	4.591873646	6.804044247	2.212170601	4.633719137
stroke-15	5.211851597	6.804044247	1.59219265	3.015072409
stroke-15	5.211851597	6.804044247	1.59219265	3.015072409
stroke-15	5.211851597	6.804044247	1.59219265	3.015072409
stroke-16	5.007183552	6.804044247	1.796860695	3.474633217
stroke-16	5.007183552	6.804044247	1.796860695	3.474633217
stroke-16	5.007183552	6.804044247	1.796860695	3.474633217
control-17	3.832456589	6.804044247	2.971587658	7.843989791
control-17	3.832456589	6.804044247	2.971587658	7.843989791
control-17	3.832456589	6.804044247	2.971587658	7.843989791
control-18	3.958292723	6.804044247	2.845751524	7.188802775
control-18	3.958292723	6.804044247	2.845751524	7.188802775
control-18	3.958292723	6.804044247	2.845751524	7.188802775
control-19	3.648995161	6.804044247	3.155049086	8.907676011
control-19	3.648995161	6.804044247	3.155049086	8.907676011
control-19	3.648995161	6.804044247	3.155049086	8.907676011
control-20	4.986311436	6.804044247	1.817732811	3.525267687
control-20	4.986311436	6.804044247	1.817732811	3.525267687
control-20	4.986311436	6.804044247	1.817732811	3.525267687
control-21	4.844490051	6.804044247	1.959554195	3.889417742
control-21	4.844490051	6.804044247	1.959554195	3.889417742
control-21	4.844490051	6.804044247	1.959554195	3.889417742
control-22	4.939656734	6.804044247	1.864387512	3.641133184
control-22	4.939656734	6.804044247	1.864387512	3.641133184
control-22	4.939656734	6.804044247	1.864387512	3.641133184
control-23	3.69518733	6.804044247	3.108856916	8.626987804
control-23	3.69518733	6.804044247	3.108856916	8.626987804
control-23	3.69518733	6.804044247	3.108856916	8.626987804
control-24	3.261012316	6.804044247	3.543031931	11.65625089
control-24	3.261012316	6.804044247	3.543031931	11.65625089
control-24	3.261012316	6.804044247	3.543031931	11.65625089
stroke-17	5.254110336	6.804044247	1.54993391	2.928037256
stroke-17	5.254110336	6.804044247	1.54993391	2.928037256
stroke-17	5.254110336	6.804044247	1.54993391	2.928037256
stroke-18	6.006075382	6.804044247	0.797968864	1.738651597
stroke-18	6.006075382	6.804044247	0.797968864	1.738651597
stroke-18	6.006075382	6.804044247	0.797968864	1.738651597
stroke-19	4.630680084	6.804044247	2.173364162	4.510740098
stroke-19	4.630680084	6.804044247	2.173364162	4.510740098
stroke-19	4.630680084	6.804044247	2.173364162	4.510740098
stroke-20	4.953790665	6.804044247	1.850253582	3.605635556
stroke-20	4.953790665	6.804044247	1.850253582	3.605635556
stroke-20	4.953790665	6.804044247	1.850253582	3.605635556
stroke-21	3.963003874	6.804044247	2.841040373	7.165365876
stroke-21	3.963003874	6.804044247	2.841040373	7.165365876
stroke-21	3.963003874	6.804044247	2.841040373	7.165365876
stroke-22	4.455628872	6.804044247	2.348415375	5.092645783
stroke-22	4.455628872	6.804044247	2.348415375	5.092645783
stroke-22	4.455628872	6.804044247	2.348415375	5.092645783
stroke-23	4.193437576	6.804044247	2.61060667	6.107604616
stroke-23	4.193437576	6.804044247	2.61060667	6.107604616
stroke-23	4.193437576	6.804044247	2.61060667	6.107604616
stroke-24	4.146551132	6.804044247	2.657493114	6.309357568
stroke-24	4.146551132	6.804044247	2.657493114	6.309357568
stroke-24	4.146551132	6.804044247	2.657493114	6.309357568
control-25	1.454742432	6.804044247	5.349301815	40.7662067
control-25	1.454742432	6.804044247	5.349301815	40.7662067
control-25	1.454742432	6.804044247	5.349301815	40.7662067
control-26	0.832572937	6.804044247	5.97147131	62.74685781
control-26	0.832572937	6.804044247	5.97147131	62.74685781
control-26	0.832572937	6.804044247	5.97147131	62.74685781
control-27	2.814962387	6.804044247	3.98908186	15.87937098
control-27	2.814962387	6.804044247	3.98908186	15.87937098

control-27	2.814962387	6.804044247	3.98908186	15.87937098
control-28	1.905248642	6.804044247	4.898795605	29.83214077
control-28	1.905248642	6.804044247	4.898795605	29.83214077
control-28	1.905248642	6.804044247	4.898795605	29.83214077
control-29	2.568133116	6.804044247	4.235911131	18.84240392
control-29	2.568133116	6.804044247	4.235911131	18.84240392
control-29	2.568133116	6.804044247	4.235911131	18.84240392
control-30	1.87986815	6.804044247	4.924176097	30.36160383
control-30	1.87986815	6.804044247	4.924176097	30.36160383
control-30	1.87986815	6.804044247	4.924176097	30.36160383
control-31	2.706959486	6.804044247	4.097084761	17.11375884
control-31	2.706959486	6.804044247	4.097084761	17.11375884
control-31	2.706959486	6.804044247	4.097084761	17.11375884
control-32	1.468818665	6.804044247	5.335225582	40.37038897
control-32	1.468818665	6.804044247	5.335225582	40.37038897
control-32	1.468818665	6.804044247	5.335225582	40.37038897
stroke-25	4.291457653	6.804044247	2.512586594	5.706422605
stroke-25	4.291457653	6.804044247	2.512586594	5.706422605
stroke-25	4.291457653	6.804044247	2.512586594	5.706422605
stroke-26	4.149076939	6.804044247	2.654967308	6.298321089
stroke-26	4.149076939	6.804044247	2.654967308	6.298321089
stroke-26	4.149076939	6.804044247	2.654967308	6.298321089
stroke-27	6.699556828	6.804044247	0.104487419	1.075112342
stroke-27	6.699556828	6.804044247	0.104487419	1.075112342
stroke-27	6.699556828	6.804044247	0.104487419	1.075112342
stroke-28	2.919909239	6.804044247	3.884135008	14.76526156
stroke-28	2.919909239	6.804044247	3.884135008	14.76526156
stroke-28	2.919909239	6.804044247	3.884135008	14.76526156
stroke-29	1.597620606	6.804044247	5.20642364	36.92237984
stroke-29	1.597620606	6.804044247	5.20642364	36.92237984
stroke-29	1.597620606	6.804044247	5.20642364	36.92237984
stroke-30	3.950876951	6.804044247	2.853167295	7.225849945
stroke-30	3.950876951	6.804044247	2.853167295	7.225849945
stroke-30	3.950876951	6.804044247	2.853167295	7.225849945
stroke-31	2.159992218	6.804044247	4.644052029	25.00339386
stroke-31	2.159992218	6.804044247	4.644052029	25.00339386
stroke-31	2.159992218	6.804044247	4.644052029	25.00339386
stroke-32	2.619544268	6.804044247	4.184499979	18.18276855
stroke-32	2.619544268	6.804044247	4.184499979	18.18276855
stroke-32	2.619544268	6.804044247	4.184499979	18.18276855

sample	RQ	sample	RQ
control-1	10.99159588	stroke-1	4.631612232
control-2	12.10853254	stroke-2	2.028128066
control-3	3.315450996	stroke-3	13.52694683
control-4	19.19944074	stroke-4	2.997400003
control-5	5.047631444	stroke-5	4.373799771
control-6	6.012831973	stroke-6	5.269701673
control-7	4.827344355	stroke-7	3.977064575
control-8	4.975245918	stroke-8	12.27795482
control-9	15.26718652	stroke-9	7.050808028
control-10	12.60324293	stroke-10	4.708442777
control-11	3.063474937	stroke-11	1
control-12	6.154432791	stroke-12	1.976959125
control-13	16.41257901	stroke-13	3.601629999
control-14	3.591780987	stroke-14	4.633719137
control-15	4.551166546	stroke-15	3.015072409
control-16	2.63768093	stroke-16	3.474633217
control-17	7.843989791	stroke-17	2.928037256
control-18	7.188802775	stroke-18	1.738651597
control-19	8.907676011	stroke-19	4.510740098
control-20	3.525267687	stroke-20	3.605635556
control-21	3.889417742	stroke-21	7.165365876

control-22	3.641133184	stroke-22	5.092645783
control-23	8.626987804	stroke-23	6.107604616
control-24	11.65625089	stroke-24	6.309357568
control-25	40.7662067	stroke-25	5.706422605
control-26	62.74685781	stroke-26	6.298321089
control-27	15.87937098	stroke-27	1.075112342
control-28	29.83214077	stroke-28	14.76526156
control-29	18.84240392	stroke-29	36.92237984
control-30	30.36160383	stroke-30	7.225849945
control-31	17.11375884	stroke-31	25.00339386
control-32	40.37038897	stroke-32	18.18276855

raw Ct data-circRNA-005548

sample	Raw Cт	Cт Mean	
control-1	25.54924202	25.5304184	GAPDH
control-1	25.21582222	25.5304184	GAPDH
control-1	25.82618904	25.5304184	GAPDH
control-2	26.18410683	26.18205833	GAPDH
control-2	26.32764816	26.18205833	GAPDH
control-2	26.03442192	26.18205833	GAPDH
control-3	25.60534668	25.69304848	GAPDH
control-3	25.82950401	25.69304848	GAPDH
control-3	25.64429855	25.69304848	GAPDH
control-4	26.16055679	25.89408112	GAPDH
control-4	25.5130291	25.89408112	GAPDH
control-4	26.00865746	25.89408112	GAPDH
control-5	26.62539101	26.80245972	GAPDH
control-5	26.93436241	26.80245972	GAPDH
control-5	26.84762764	26.80245972	GAPDH
control-6	25.94897652	25.77236938	GAPDH
control-6	25.78363991	25.77236938	GAPDH
control-6	25.58449554	25.77236938	GAPDH
control-7	25.91959572	25.63115883	GAPDH
control-7	26.07379532	25.63115883	GAPDH
control-7	24.90008545	25.63115883	GAPDH
control-8	27.01070786	26.91640282	GAPDH
control-8	26.8848629	26.91640282	GAPDH
control-8	26.8536377	26.91640282	GAPDH
stroke-1	23.47739601	24.01311684	GAPDH
stroke-1	24.05852127	24.01311684	GAPDH
stroke-1	24.50343513	24.01311684	GAPDH
stroke-2	28.49221039	28.04417419	GAPDH
stroke-2	27.84469986	28.04417419	GAPDH
stroke-2	27.79561424	28.04417419	GAPDH
stroke-3	24.66034317	24.91215515	GAPDH
stroke-3	24.92931557	24.91215515	GAPDH
stroke-3	25.14680099	24.91215515	GAPDH
stroke-4	26.82505226	28.24936676	GAPDH
stroke-4	26.71202469	28.24936676	GAPDH
stroke-4	31.21102142	28.24936676	GAPDH
stroke-5	24.5884552	24.8166256	GAPDH
stroke-5	25.039217	24.8166256	GAPDH
stroke-5	24.82220078	24.8166256	GAPDH
stroke-6	35.46171188	35.68018341	GAPDH
stroke-6	35.89865112	35.68018341	GAPDH
stroke-6	Undetermined	35.68018341	GAPDH
stroke-7	22.39485168	22.81052589	GAPDH
stroke-7	22.87345886	22.81052589	GAPDH
stroke-7	23.16327095	22.81052589	GAPDH
stroke-8	23.62879181	24.84118652	GAPDH
stroke-8	23.25596237	24.84118652	GAPDH
stroke-8	27.63880348	24.84118652	GAPDH
control-1	30.13471222	29.7931118	circ-0005548
control-1	29.37664986	29.7931118	circ-0005548

control-1	29.86797333	29.7931118	circ-0005548
control-2	29.92319489	29.46327209	circ-0005548
control-2	29.48360252	29.46327209	circ-0005548
control-2	28.98301697	29.46327209	circ-0005548
control-3	29.43703079	29.39609337	circ-0005548
control-3	29.71819496	29.39609337	circ-0005548
control-3	29.03305244	29.39609337	circ-0005548
control-4	28.53248405	28.74155426	circ-0005548
control-4	28.88143539	28.74155426	circ-0005548
control-4	28.81074333	28.74155426	circ-0005548
control-5	29.82089424	30.12084389	circ-0005548
control-5	30.30127335	30.12084389	circ-0005548
control-5	30.24036407	30.12084389	circ-0005548
control-6	28.93020439	28.93260002	circ-0005548
control-6	28.92459488	28.93260002	circ-0005548
control-6	28.94299889	28.93260002	circ-0005548
control-7	29.14531326	29.3142643	circ-0005548
control-7	29.33281326	29.3142643	circ-0005548
control-7	29.46466637	29.3142643	circ-0005548
control-8	30.54811668	30.06637001	circ-0005548
control-8	29.89681435	30.06637001	circ-0005548
control-8	29.75418091	30.06637001	circ-0005548
stroke-1	28.84966278	28.8271656	circ-0005548
stroke-1	29.40960693	28.8271656	circ-0005548
stroke-1	28.222229	28.8271656	circ-0005548
stroke-2	30.83957863	31.3448391	circ-0005548
stroke-2	31.21593285	31.3448391	circ-0005548
stroke-2	31.97900391	31.3448391	circ-0005548
stroke-3	28.89603233	29.23703957	circ-0005548
stroke-3	29.41209984	29.23703957	circ-0005548
stroke-3	29.40299034	29.23703957	circ-0005548
stroke-4	29.9642334	30.0290451	circ-0005548
stroke-4	29.97315979	30.0290451	circ-0005548
stroke-4	30.14974403	30.0290451	circ-0005548
stroke-5	29.09393501	29.22374535	circ-0005548
stroke-5	29.73144913	29.22374535	circ-0005548
stroke-5	28.8458519	29.22374535	circ-0005548
stroke-6	28.86117172	28.70638466	circ-0005548
stroke-6	28.98764992	28.70638466	circ-0005548
stroke-6	28.27033806	28.70638466	circ-0005548
stroke-7	28.2237339	28.06319237	circ-0005548
stroke-7	27.77008247	28.06319237	circ-0005548
stroke-7	28.19575691	28.06319237	circ-0005548
stroke-8	28.37052155	28.6648407	circ-0005548
stroke-8	28.76832771	28.6648407	circ-0005548
stroke-8	28.85567474	28.6648407	circ-0005548
control9	18.49805832	18.60410137	GAPDH
control9	18.71014023	18.60410137	GAPDH
control9	18.60410557	18.60410137	GAPDH
control 10	20.03706932	20.55056953	GAPDH
control 10	20.79405975	20.55056953	GAPDH
control 10	20.82057762	20.55056953	GAPDH
control11	20.23067474	19.85377502	GAPDH
control11	19.47781372	19.85377502	GAPDH
control11	19.85283852	19.85377502	GAPDH
control 12	18.5678978	19.01976585	GAPDH
control 12	19.01865768	19.01976585	GAPDH
control 12	19.47274017	19.01976585	GAPDH
control 13	18.53126717	18.68808746	GAPDH
control 13	19.43749428	18.68808746	GAPDH
control 13	18.09549904	18.68808746	GAPDH
control 14	20.79653358	20.88736534	GAPDH
control 14	20.72556114	20.88736534	GAPDH
control 14	21.13999748	20.88736534	GAPDH
control 15	19.49267006	19.57484627	GAPDH

control 15	19.41773605	19.57484627	GAPDH
control 15	19.81413841	19.57484627	GAPDH
control 16	18.50798607	19.14769745	GAPDH
control 16	19.35250473	19.14769745	GAPDH
control 16	19.58259773	19.14769745	GAPDH
stroke-9	25.51529503	24.94852448	GAPDH
stroke-9	24.22061348	24.94852448	GAPDH
stroke-9	25.10966301	24.94852448	GAPDH
stroke-10	24.22356224	24.27387238	GAPDH
stroke-10	24.11852646	24.27387238	GAPDH
stroke-10	24.47952843	24.27387238	GAPDH
stroke-11	21.25199699	21.2925396	GAPDH
stroke-11	21.21507645	21.2925396	GAPDH
stroke-11	21.41053963	21.2925396	GAPDH
stroke-12	21.31844139	21.29785156	GAPDH
stroke-12	21.33186531	21.29785156	GAPDH
stroke-12	21.24324799	21.29785156	GAPDH
stroke-13	21.75457382	21.79486084	GAPDH
stroke-13	21.78793907	21.79486084	GAPDH
stroke-13	21.84206963	21.79486084	GAPDH
stroke-14	20.01085663	19.99152946	GAPDH
stroke-14	19.27136803	19.99152946	GAPDH
stroke-14	20.69235992	19.99152946	GAPDH
stroke-15	20.33838272	20.65740776	GAPDH
stroke-15	21.03596497	20.65740776	GAPDH
stroke-15	20.5978775	20.65740776	GAPDH
stroke-16	21.27140236	21.15710258	GAPDH
stroke-16	20.84852409	21.15710258	GAPDH
stroke-16	21.3513813	21.15710258	GAPDH
control9	28.27529716	27.79139137	circ-0005548
control9	27.6286869	27.79139137	circ-0005548
control9	27.47019196	27.79139137	circ-0005548
control 10	29.32610321	29.172472	circ-0005548
control 10	28.98474884	29.172472	circ-0005548
control 10	29.20656586	29.172472	circ-0005548
control11	27.27981758	27.5242939	circ-0005548
control11	27.74784279	27.5242939	circ-0005548
control11	27.54522133	27.5242939	circ-0005548
control 12	27.03682899	27.23122406	circ-0005548
control 12	27.07709694	27.23122406	circ-0005548
control 12	27.57974434	27.23122406	circ-0005548
control 13	28.36922264	28.32983208	circ-0005548
control 13	27.95604706	28.32983208	circ-0005548
control 13	28.66422272	28.32983208	circ-0005548
control 14	27.96492386	27.70201111	circ-0005548
control 14	27.1071949	27.70201111	circ-0005548
control 14	28.03391838	27.70201111	circ-0005548
control 15	26.80350304	27.21355438	circ-0005548
control 15	27.17761612	27.21355438	circ-0005548
control 15	27.65954399	27.21355438	circ-0005548
control 16	27.34659576	27.57818604	circ-0005548
control 16	27.83520508	27.57818604	circ-0005548
control 16	27.55275345	27.57818604	circ-0005548
stroke-9	31.32762527	30.80535316	circ-0005548
stroke-9	30.64169312	30.80535316	circ-0005548
stroke-9	30.4467392	30.80535316	circ-0005548
stroke-10	27.89973068	28.98496437	circ-0005548
stroke-10	28.12163162	28.98496437	circ-0005548
stroke-10	30.9335289	28.98496437	circ-0005548
stroke-11	28.32009697	28.09488869	circ-0005548
stroke-11	28.06675529	28.09488869	circ-0005548
stroke-11	27.89781189	28.09488869	circ-0005548
stroke-12	28.87808609	28.9653492	circ-0005548
stroke-12	28.75458908	28.9653492	circ-0005548
stroke-12	29.26337433	28.9653492	circ-0005548

stroke-13	30.20879745	30.18146706	circ-0005548
stroke-13	30.82528305	30.18146706	circ-0005548
stroke-13	29.51032257	30.18146706	circ-0005548
stroke-14	28.57320976	28.7130127	circ-0005548
stroke-14	28.71076202	28.7130127	circ-0005548
stroke-14	28.85506821	28.7130127	circ-0005548
stroke-15	28.57791138	28.82722282	circ-0005548
stroke-15	28.75793839	28.82722282	circ-0005548
stroke-15	29.1458149	28.82722282	circ-0005548
stroke-16	28.82444763	28.85632324	circ-0005548
stroke-16	28.92103195	28.85632324	circ-0005548
stroke-16	28.82348824	28.85632324	circ-0005548
control-17	25.84146309	25.73897362	GAPDH
control-17	25.44108582	25.73897362	GAPDH
control-17	25.93436813	25.73897362	GAPDH
control-18	26.14860535	25.95986938	GAPDH
control-18	25.83952904	25.95986938	GAPDH
control-18	25.89147568	25.95986938	GAPDH
control-19	25.49755478	25.56768799	GAPDH
control-19	25.57378006	25.56768799	GAPDH
control-19	25.63172913	25.56768799	GAPDH
control-20	25.14019203	25.3269825	GAPDH
control-20	25.29609299	25.3269825	GAPDH
control-20	25.54466438	25.3269825	GAPDH
control-21	26.88145828	26.79045105	GAPDH
control-21	26.85717964	26.79045105	GAPDH
control-21	26.63271904	26.79045105	GAPDH
control-22	25.9525528	25.74632072	GAPDH
control-22	25.5992794	25.74632072	GAPDH
control-22	25.68712997	25.74632072	GAPDH
control-23	25.78086853	25.92199516	GAPDH
control-23	25.96206093	25.92199516	GAPDH
control-23	26.02305412	25.92199516	GAPDH
control-24	26.70739365	26.71579933	GAPDH
control-24	26.65486145	26.71579933	GAPDH
control-24	26.78514671	26.71579933	GAPDH
stroke-17	24.64425087	24.57648659	GAPDH
stroke-17	24.61152267	24.57648659	GAPDH
stroke-17	24.47368813	24.57648659	GAPDH
stroke-18	28.17127419	28.12898254	GAPDH
stroke-18	28.19966698	28.12898254	GAPDH
stroke-18	28.01600647	28.12898254	GAPDH
stroke-19	24.8534584	24.96452332	GAPDH
stroke-19	24.90655327	24.96452332	GAPDH
stroke-19	25.13355446	24.96452332	GAPDH
stroke-20	26.54661369	27.98045921	GAPDH
stroke-20	26.804636	27.98045921	GAPDH
stroke-20	30.59012794	27.98045921	GAPDH
stroke-21	24.88801956	24.81388855	GAPDH
stroke-21	24.85343933	24.81388855	GAPDH
stroke-21	24.70020866	24.81388855	GAPDH
stroke-22	23.66060257	23.50028038	GAPDH
stroke-22	23.86288452	23.50028038	GAPDH
stroke-22	22.97735405	23.50028038	GAPDH
stroke-23	23.52408409	23.7984848	GAPDH
stroke-23	23.82956886	23.7984848	GAPDH
stroke-23	24.04180145	23.7984848	GAPDH
stroke-24	25.91849899	26.92940331	GAPDH
stroke-24	25.98884392	26.92940331	GAPDH
stroke-24	28.88086128	26.92940331	GAPDH
control-17	29.31820107	29.43020439	circ-0005548
control-17	29.2917366	29.43020439	circ-0005548
control-17	29.6806736	29.43020439	circ-0005548
control-18	29.96067429	29.96424294	circ-0005548
control-18	30.00048256	29.96424294	circ-0005548

control-18	29.93156815	29.96424294	circ-0005548
control-19	29.35986519	29.45892143	circ-0005548
control-19	29.30387115	29.45892143	circ-0005548
control-19	29.71302605	29.45892143	circ-0005548
control-20	28.83373451	28.74543953	circ-0005548
control-20	28.76586723	28.74543953	circ-0005548
control-20	28.63671875	28.74543953	circ-0005548
control-21	30.94665146	30.76081848	circ-0005548
control-21	31.01482582	30.76081848	circ-0005548
control-21	30.32098389	30.76081848	circ-0005548
control-22	28.89760208	28.75770569	circ-0005548
control-22	28.74171448	28.75770569	circ-0005548
control-22	28.63380432	28.75770569	circ-0005548
control-23	28.95204544	28.93157005	circ-0005548
control-23	28.90785789	28.93157005	circ-0005548
control-23	28.93480301	28.93157005	circ-0005548
control-24	29.60251045	29.23786926	circ-0005548
control-24	29.57151604	29.23786926	circ-0005548
control-24	28.53958321	29.23786926	circ-0005548
stroke-17	29.64040184	29.0573349	circ-0005548
stroke-17	29.05752373	29.0573349	circ-0005548
stroke-17	28.47407913	29.0573349	circ-0005548
stroke-18	30.98116875	31.19766235	circ-0005548
stroke-18	30.97106171	31.19766235	circ-0005548
stroke-18	31.6407547	31.19766235	circ-0005548
stroke-19	29.54527283	29.63822746	circ-0005548
stroke-19	29.52233887	29.63822746	circ-0005548
stroke-19	29.84707069	29.63822746	circ-0005548
stroke-20	29.7865181	29.72163391	circ-0005548
stroke-20	29.68669128	29.72163391	circ-0005548
stroke-20	29.69169807	29.72163391	circ-0005548
stroke-21	29.87291718	29.92972755	circ-0005548
stroke-21	29.87873459	29.92972755	circ-0005548
stroke-21	30.0375309	29.92972755	circ-0005548
stroke-22	29.8132782	29.37895203	circ-0005548
stroke-22	29.19760895	29.37895203	circ-0005548
stroke-22	29.12596703	29.37895203	circ-0005548
stroke-23	29.4093132	29.35191345	circ-0005548
stroke-23	29.0681057	29.35191345	circ-0005548
stroke-23	29.57831573	29.35191345	circ-0005548
stroke-24	28.61583138	28.6761322	circ-0005548
stroke-24	28.5135498	28.6761322	circ-0005548
stroke-24	28.89901733	28.6761322	circ-0005548
control-25	24.67868423	25.00511932	GAPDH
control-25	25.12644196	25.00511932	GAPDH
control-25	25.21023178	25.00511932	GAPDH
control-26	22.17980957	22.68137932	GAPDH
control-26	23.07166672	22.68137932	GAPDH
control-26	22.79266167	22.68137932	GAPDH
control-27	23.39224625	24.72233009	GAPDH
control-27	25.39827919	24.72233009	GAPDH
control-27	25.37647057	24.72233009	GAPDH
control-28	24.03521538	24.23166084	GAPDH
control-28	23.88560486	24.23166084	GAPDH
control-28	24.77416039	24.23166084	GAPDH
control-29	23.53442383	23.73780823	GAPDH
control-29	23.91172791	23.73780823	GAPDH
control-29	23.76727295	23.73780823	GAPDH
control-30	25.26303864	24.47930336	GAPDH
control-30	23.9221344	24.47930336	GAPDH
control-30	24.25274086	24.47930336	GAPDH
control-31	22.96356583	23.54529762	GAPDH
control-31	23.40715981	23.54529762	GAPDH
control-31	24.26517105	23.54529762	GAPDH
control-32	24.89213753	25.24001312	GAPDH

control-32	25.58073616	25.24001312	GAPDH
control-32	25.24716949	25.24001312	GAPDH
stroke-25	24.10925293	24.26269722	GAPDH
stroke-25	24.95045662	24.26269722	GAPDH
stroke-25	23.72838593	24.26269722	GAPDH
stroke-26	22.71949768	22.87644386	GAPDH
stroke-26	22.84646416	22.87644386	GAPDH
stroke-26	23.06337547	22.87644386	GAPDH
stroke-27	24.76853752	25.5241909	GAPDH
stroke-27	25.82514572	25.5241909	GAPDH
stroke-27	25.97888756	25.5241909	GAPDH
stroke-28	21.53846169	21.51788521	GAPDH
stroke-28	21.19041252	21.51788521	GAPDH
stroke-28	21.82478523	21.51788521	GAPDH
stroke-29	25.44576836	25.83963013	GAPDH
stroke-29	26.39275169	25.83963013	GAPDH
stroke-29	25.68036652	25.83963013	GAPDH
stroke-30	22.82670784	22.95581818	GAPDH
stroke-30	22.99809837	22.95581818	GAPDH
stroke-30	23.04265022	22.95581818	GAPDH
stroke-31	23.24854279	22.61452293	GAPDH
stroke-31	21.68766785	22.61452293	GAPDH
stroke-31	22.90736008	22.61452293	GAPDH
stroke-32	22.25065231	23.17136574	GAPDH
stroke-32	24.14462852	23.17136574	GAPDH
stroke-32	23.11882019	23.17136574	GAPDH
control-25	28.18592072	28.40953827	circ-0005548
control-25	28.50474358	28.40953827	circ-0005548
control-25	28.53794861	28.40953827	circ-0005548
control-26	26.01140404	25.95163918	circ-0005548
control-26	25.89340782	25.95163918	circ-0005548
control-26	25.95010376	25.95163918	circ-0005548
control-27	27.56446838	25.48612976	circ-0005548
control-27	22.93903923	25.48612976	circ-0005548
control-27	25.95488358	25.48612976	circ-0005548
control-28	28.63087654	28.22242737	circ-0005548
control-28	28.14642715	28.22242737	circ-0005548
control-28	27.88998222	28.22242737	circ-0005548
control-29	27.48137474	26.4030056	circ-0005548
control-29	26.96049309	26.4030056	circ-0005548
control-29	24.76714897	26.4030056	circ-0005548
control-30	27.20843506	27.48822021	circ-0005548
control-30	27.90198898	27.48822021	circ-0005548
control-30	27.35423279	27.48822021	circ-0005548
control-31	28.00518608	27.95604134	circ-0005548
control-31	28.03863907	27.95604134	circ-0005548
control-31	27.82429886	27.95604134	circ-0005548
control-32	28.23088646	27.546278	circ-0005548
control-32	28.51524544	27.546278	circ-0005548
control-32	25.89269638	27.546278	circ-0005548
stroke-25	22.77723312	27.37389183	circ-0005548
stroke-25	29.82105255	27.37389183	circ-0005548
stroke-25	29.52338982	27.37389183	circ-0005548
stroke-26	27.66707993	27.66615868	circ-0005548
stroke-26	27.9228363	27.66615868	circ-0005548
stroke-26	27.40856171	27.66615868	circ-0005548
stroke-27	23.18893623	24.71518135	circ-0005548
stroke-27	22.33311462	24.71518135	circ-0005548
stroke-27	28.62349701	24.71518135	circ-0005548
stroke-28	26.18709373	26.12702179	circ-0005548
stroke-28	25.86688995	26.12702179	circ-0005548
stroke-28	26.32708168	26.12702179	circ-0005548
stroke-29	29.07029724	27.15695763	circ-0005548
stroke-29	29.14267731	27.15695763	circ-0005548
stroke-29	23.25790024	27.15695763	circ-0005548

stroke-30	27.14013481	24.42239189	circ-0005548	
stroke-30	23.75274277	24.42239189	circ-0005548	
stroke-30	22.37429237	24.42239189	circ-0005548	
stroke-31	22.36986351	23.99481964	circ-0005548	
stroke-31	23.54318619	23.99481964	circ-0005548	
stroke-31	26.07140541	23.99481964	circ-0005548	
stroke-32	26.64640617	26.95273399	circ-0005548	
stroke-32	27.1030674	26.95273399	circ-0005548	
stroke-32	27.10872459	26.95273399	circ-0005548	

sample	△CT		ΔΔCт	RQ
control-1	4.262693882	9.64174366	5.379049778	41.61552054
control-1	4.262693882	9.64174366	5.379049778	41.61552054
control-1	4.262693882	9.64174366	5.379049778	41.61552054
control-2	3.281212568	9.64174366	6.360531092	82.1695005
control-2	3.281212568	9.64174366	6.360531092	82.1695005
control-2	3.281212568	9.64174366	6.360531092	82.1695005
control-3	3.703042984	9.64174366	5.938700676	61.33763668
control-3	3.703042984	9.64174366	5.938700676	61.33763668
control-3	3.703042984	9.64174366	5.938700676	61.33763668
control-4	2.847473145	9.64174366	6.794270515	110.9888174
control-4	2.847473145	9.64174366	6.794270515	110.9888174
control-4	2.847473145	9.64174366	6.794270515	110.9888174
control-5	3.318383455	9.64174366	6.323360205	80.07945246
control-5	3.318383455	9.64174366	6.323360205	80.07945246
control-5	3.318383455	9.64174366	6.323360205	80.07945246
control-6	3.160228729	9.64174366	6.481514931	89.3573769
control-6	3.160228729	9.64174366	6.481514931	89.3573769
control-6	3.160228729	9.64174366	6.481514931	89.3573769
control-7	3.683105469	9.64174366	5.958638191	62.19118455
control-7	3.683105469	9.64174366	5.958638191	62.19118455
control-7	3.683105469	9.64174366	5.958638191	62.19118455
control-8	3.149967909	9.64174366	6.491775751	89.99517508
control-8	3.149967909	9.64174366	6.491775751	89.99517508
control-8	3.149967909	9.64174366	6.491775751	89.99517508
stroke-1	3.72	9.64174366	5.92174366	60.62091235
stroke-1	3.72	9.64174366	5.92174366	60.62091235
stroke-1	3.72	9.64174366	5.92174366	60.62091235
stroke-2	3.30066371	9.64174366	6.34107995	81.06908475
stroke-2	3.30066371	9.64174366	6.34107995	81.06908475
stroke-2	3.30066371	9.64174366	6.34107995	81.06908475
stroke-3	4.324887753	9.64174366	5.316855907	39.85961603
stroke-3	4.324887753	9.64174366	5.316855907	39.85961603
stroke-3	4.324887753	9.64174366	5.316855907	39.85961603
stroke-4	1.779679656	9.64174366	7.862064004	232.6575192
stroke-4	1.779679656	9.64174366	7.862064004	232.6575192
stroke-4	1.779679656	9.64174366	7.862064004	232.6575192
stroke-5	4.407121181	9.64174366	5.234622478	37.65116182
stroke-5	4.407121181	9.64174366	5.234622478	37.65116182
stroke-5	4.407121181	9.64174366	5.234622478	37.65116182
stroke-6	1	9.64174366	8.64174366	399.414711
stroke-6	1	9.64174366	8.64174366	399.414711
stroke-6	1	9.64174366	8.64174366	399.414711
stroke-7	5.252664089	9.64174366	4.389079571	20.95292238
stroke-7	5.252664089	9.64174366	4.389079571	20.95292238
stroke-7	5.252664089	9.64174366	4.389079571	20.95292238
stroke-8	3.823655367	9.64174366	5.818088293	56.4181828
stroke-8	3.823655367	9.64174366	5.818088293	56.4181828
stroke-8	3.823655367	9.64174366	5.818088293	56.4181828
control9	9.18729	14	4.81271	28.10412513
control9	9.18729	14	4.81271	28.10412513
control9	9.18729	14	4.81271	28.10412513
control 10	8.621903419	14	5.378096581	41.588034

control 10	8.621903419	14	5.378096581	41.588034
control 10	8.621903419	14	5.378096581	41.588034
control11	7.670518398	14	6.329481602	80.41995377
control11	7.670518398	14	6.329481602	80.41995377
control11	7.670518398	14	6.329481602	80.41995377
control 12	8.211458206	14	5.788541794	55.27448579
control 12	8.211458206	14	5.788541794	55.27448579
control 12	8.211458206	14	5.788541794	55.27448579
control 13	9.64174366	14	4.35825634	20.51001071
control 13	9.64174366	14	4.35825634	20.51001071
control 13	9.64174366	14	4.35825634	20.51001071
control 14	6.814648151	14	7.185351849	145.5480649
control 14	6.814648151	14	7.185351849	145.5480649
control 14	6.814648151	14	7.185351849	145.5480649
control 15	7.638706207	14	6.361293793	82.21295205
control 15	7.638706207	14	6.361293793	82.21295205
control 15	7.638706207	14	6.361293793	82.21295205
control 16	8.430488586	14	5.569511414	47.48866891
control 16	8.430488586	14	5.569511414	47.48866891
control 16	8.430488586	14	5.569511414	47.48866891
stroke-9	5.85682869	14	8.14317131	282.7084726
stroke-9	5.85682869	14	8.14317131	282.7084726
stroke-9	5.85682869	14	8.14317131	282.7084726
stroke-10	4.711091518	14	9.288908482	625.5183866
stroke-10	4.711091518	14	9.288908482	625.5183866
stroke-10	4.711091518	14	9.288908482	625.5183866
stroke-11	6.802350521	14	7.197649479	146.7940293
stroke-11	6.802350521	14	7.197649479	146.7940293
stroke-11	6.802350521	14	7.197649479	146.7940293
stroke-12	7.667498112	14	6.332501888	80.58848955
stroke-12	7.667498112	14	6.332501888	80.58848955
stroke-12	7.667498112	14	6.332501888	80.58848955
stroke-13	8.38660717	14	5.61339283	48.95528906
stroke-13	8.38660717	14	5.61339283	48.95528906
stroke-13	8.38660717	14	5.61339283	48.95528906
stroke-14	8.721485138	14	5.278514862	38.81425958
stroke-14	8.721485138	14	5.278514862	38.81425958
stroke-14	8.721485138	14	5.278514862	38.81425958
stroke-15	8.169813156	14	5.830186844	56.89329939
stroke-15	8.169813156	14	5.830186844	56.89329939
stroke-15	8.169813156	14	5.830186844	56.89329939
stroke-16	7.699220181	14	6.300779819	78.83584405
stroke-16	7.699220181	14	6.300779819	78.83584405
stroke-16	7.699220181	14	6.300779819	78.83584405
control-17	3.691231489	9.64174366	5.950512171	61.84187562
control-17	3.691231489	9.64174366	5.950512171	61.84187562
control-17	3.691231489	9.64174366	5.950512171	61.84187562
control-18	4.004371643	9.64174366	5.637372017	49.77577995
control-18	4.004371643	9.64174366	5.637372017	49.77577995
control-18	4.004371643	9.64174366	5.637372017	49.77577995
control-19	3.891232729	9.64174366	5.750510931	53.83643339
control-19	3.891232729	9.64174366	5.750510931	53.83643339
control-19	3.891232729	9.64174366	5.750510931	53.83643339
control-20	3.418457031	9.64174366	6.223286629	74.71296071
control-20	3.418457031	9.64174366	6.223286629	74.71296071
control-20	3.418457031	9.64174366	6.223286629	74.71296071
control-21	3.970368147	9.64174366	5.671375513	50.96290152
control-21	3.970368147	9.64174366	5.671375513	50.96290152
control-21	3.970368147	9.64174366	5.671375513	50.96290152
control-22	3.011386156	9.64174366	6.630357504	99.06870605
control-22	3.011386156	9.64174366	6.630357504	99.06870605
control-22	3.011386156	9.64174366	6.630357504	99.06870605
control-23	3.009574175	9.64174366	6.632169485	99.19321151
control-23	3.009574175	9.64174366	6.632169485	99.19321151
control-23	3.009574175	9.64174366	6.632169485	99.19321151

control-24	2.522069216	9.64174366	7.119674444	139.0706764
control-24	2.522069216	9.64174366	7.119674444	139.0706764
control-24	2.522069216	9.64174366	7.119674444	139.0706764
stroke-17	4.480847836	9.64174366	5.160895824	35.77539583
stroke-17	4.480847836	9.64174366	5.160895824	35.77539583
stroke-17	4.480847836	9.64174366	5.160895824	35.77539583
stroke-18	3.068679094	9.64174366	6.573064566	95.21154175
stroke-18	3.068679094	9.64174366	6.573064566	95.21154175
stroke-18	3.068679094	9.64174366	6.573064566	95.21154175
stroke-19	4.673705578	9.64174366	4.968038082	31.29885732
stroke-19	4.673705578	9.64174366	4.968038082	31.29885732
stroke-19	4.673705578	9.64174366	4.968038082	31.29885732
stroke-20	1.741176605	9.64174366	7.900567055	238.9503474
stroke-20	1.741176605	9.64174366	7.900567055	238.9503474
stroke-20	1.741176605	9.64174366	7.900567055	238.9503474
stroke-21	5.115838528	9.64174366	4.525905132	23.03738617
stroke-21	5.115838528	9.64174366	4.525905132	23.03738617
stroke-21	5.115838528	9.64174366	4.525905132	23.03738617
stroke-22	5.878671169	9.64174366	3.763072491	13.5768086
stroke-22	5.878671169	9.64174366	3.763072491	13.5768086
stroke-22	5.878671169	9.64174366	3.763072491	13.5768086
stroke-23	5.553426743	9.64174366	4.088316917	17.01006699
stroke-23	5.553426743	9.64174366	4.088316917	17.01006699
stroke-23	5.553426743	9.64174366	4.088316917	17.01006699
stroke-24	1.7467314	9.64174366	7.895012259	238.032088
stroke-24	1.7467314	9.64174366	7.895012259	238.032088
stroke-24	1.7467314	9.64174366	7.895012259	238.032088
control-25	3.40441823	9.64174366	6.23732543	75.44353804
control-25	3.40441823	9.64174366	6.23732543	75.44353804
control-25	3.40441823	9.64174366	6.23732543	75.44353804
control-26	3.270259142	9.64174366	6.371484518	82.79573328
control-26	3.270259142	9.64174366	6.371484518	82.79573328
control-26	3.270259142	9.64174366	6.371484518	82.79573328
control-27	2.84	9.64174366	6.80174366	111.5652298
control-27	2.84	9.64174366	6.80174366	111.5652298
control-27	2.84	9.64174366	6.80174366	111.5652298
control-28	3.990768433	9.64174366	5.650975227	50.24733679
control-28	3.990768433	9.64174366	5.650975227	50.24733679
control-28	3.990768433	9.64174366	5.650975227	50.24733679
control-29	3.75	9.64174366	5.89174366	59.37335202
control-29	3.75	9.64174366	5.89174366	59.37335202
control-29	3.75	9.64174366	5.89174366	59.37335202
control-30	3.008914232	9.64174366	6.632829428	99.23859657
control-30	3.008914232	9.64174366	6.632829428	99.23859657
control-30	3.008914232	9.64174366	6.632829428	99.23859657
control-31	4.410742283	9.64174366	5.231001377	37.55677755
control-31	4.410742283	9.64174366	5.231001377	37.55677755
control-31	4.410742283	9.64174366	5.231001377	37.55677755
control-32	3.27	9.64174366	6.37174366	82.81060668
control-32	3.27	9.64174366	6.37174366	82.81060668
control-32	3.27	9.64174366	6.37174366	82.81060668
stroke-25	3.111193419	9.64174366	6.530550241	92.44672081
stroke-25	3.111193419	9.64174366	6.530550241	92.44672081
stroke-25	3.111193419	9.64174366	6.530550241	92.44672081
stroke-26	4.789713383	9.64174366	4.852030277	28.88062938
stroke-26	4.789713383	9.64174366	4.852030277	28.88062938
stroke-26	4.789713383	9.64174366	4.852030277	28.88062938
stroke-27	-0.809007645	9.64174366	10.4507513	1399.553871
stroke-27	-0.809007645	9.64174366	10.4507513	1399.553871
stroke-27	-0.809007645	9.64174366	10.4507513	1399.553871
stroke-28	4.609135151	9.64174366	5.032608509	32.73151577
stroke-28	4.609135151	9.64174366	5.032608509	32.73151577
stroke-28	4.609135151	9.64174366	5.032608509	32.73151577
stroke-29	1.317329407	9.64174366	8.324414253	320.551923
stroke-29	1.317329407	9.64174366	8.324414253	320.551923

stroke-29	1.317329407	9.64174366	8.324414253	320.551923
stroke-30	1.466571212	9.64174366	8.175172448	289.0494362
stroke-30	1.466571212	9.64174366	8.175172448	289.0494362
stroke-30	1.466571212	9.64174366	8.175172448	289.0494362
stroke-31	1.3802948	9.64174366	8.26144886	306.8625676
stroke-31	1.3802948	9.64174366	8.26144886	306.8625676
stroke-31	1.3802948	9.64174366	8.26144886	306.8625676
stroke-32	3.781365633	9.64174366	5.860378027	58.09644686
stroke-32	3.781365633	9.64174366	5.860378027	58.09644686
stroke-32	3.781365633	9.64174366	5.860378027	58.09644686

sample	RQ	sample	RQ
control-1	41.61552054	stroke-1	60.62091235
control-2	82.1695005	stroke-2	81.06908475
control-3	61.33763668	stroke-3	39.85961603
control-4	110.9888174	stroke-4	232.6575192
control-5	80.07945246	stroke-5	37.65116182
control-6	89.3573769	stroke-6	399.414711
control-7	62.19118455	stroke-7	20.95292238
control-8	89.99517508	stroke-8	56.4181828
control-9	28.10412513	stroke-9	282.7084726
control-10	41.588034	stroke-10	625.5183866
control-11	80.41995377	stroke-11	146.7940293
control-12	55.27448579	stroke-12	80.58848955
control-13	20.51001071	stroke-13	48.95528906
control-14	145.5480649	stroke-14	38.81425958
control-15	82.21295205	stroke-15	56.89329939
control-16	47.48866891	stroke-16	78.83584405
control-17	61.84187562	stroke-17	35.77539583
control-18	49.77577995	stroke-18	95.21154175
control-19	53.83643339	stroke-19	31.29885732
control-20	74.71296071	stroke-20	238.9503474
control-21	50.96290152	stroke-21	23.03738617
control-22	99.06870605	stroke-22	13.5768086
control-23	99.19321151	stroke-23	17.01006699
control-24	139.0706764	stroke-24	238.032088
control-25	75.44353804	stroke-25	92.44672081
control-26	82.79573328	stroke-26	28.88062938
control-27	111.5652298	stroke-27	1399.553871
control-28	50.24733679	stroke-28	32.73151577
control-29	59.37335202	stroke-29	320.551923
control-30	99.23859657	stroke-30	289.0494362
control-31	37.55677755	stroke-31	306.8625676
control-32	82.81060668	stroke-32	58.09644686

Characteristic of AIS patients and healthy controls （PCR）

healthy controls	age	Gender	stroke patients	age	Gender
1	75	female	1	73	female
2	60	male	2	51	male
3	71	male	3	61	male
4	69	male	4	60	male
5	69	male	5	52	male
6	73	male	6	51	female
7	63	male	7	75	female
8	62	male	8	64	male
9	64	male	9	61	female
10	54	female	10	67	male
11	63	male	11	52	male
12	53	male	12	59	female
13	62	male	13	59	male
14	63	male	14	71	female
15	66	male	15	72	female
16	64	male	16	74	female

17	81	female	17	69	male
18	60	female	18	76	male
19	69	male	19	60	male
20	58	male	20	76	female
21	63	female	21	63	male
22	64	female	22	77	female
23	73	female	23	64	male
24	58	female	24	61	male
25	62	female	25	52	male
26	55	female	26	67	male
27	58	female	27	71	female
28	63	female	28	72	female
29	64	female	29	66	male
30	58	female	30	61	male
31	62	female	31	72	male
32	61	male	32	59	female

RNA-seq

sample	RIN values
control_1	9.4
control_2	7.7
control_3	9.2
stroke_1	8.3
stroke_2	9
stroke_3	9

miR-337-3p interacction with hsa_circ_0000607(vps13c)

miRNAid	miRNAname	geneID	geneName	geneType	chromosome	start
MIMAT0000754	hsa-miR-337-3p	NM_015378	VPS13D	circRNA	chr1	12428567
MIMAT0000754	hsa-miR-337-3p	NM_015378	VPS13D	circRNA	chr1	12569392
MIMAT0000754	hsa-miR-337-3p	NM_012231	PRDM2	circRNA	chr1	14095789
MIMAT0000754	hsa-miR-337-3p	NM_015207	OTUD3	circRNA	chr1	20235114
MIMAT0000754	hsa-miR-337-3p	NM_016287	HP1BP3	circRNA	chr1	21071278
MIMAT0000754	hsa-miR-337-3p	NM_020362	PITHD1	circRNA	chr1	24114243
MIMAT0000754	hsa-miR-337-3p	NM_001166006	EPB41	circRNA	chr1	29391851
MIMAT0000754	hsa-miR-337-3p	NM_001166006	EPB41	circRNA	chr1	29422908
MIMAT0000754	hsa-miR-337-3p	NM_001020658	PUM1	circRNA	chr1	31405807
MIMAT0000754	hsa-miR-337-3p	hsa_circ_0000046	hsa_circ_001242	circRNA	chr1	31995101
MIMAT0000754	hsa-miR-337-3p	NM_152493	ZNF362	circRNA	chr1	33765691
MIMAT0000754	hsa-miR-337-3p	NM_024772	ZMYM1	circRNA	chr1	35565808
MIMAT0000754	hsa-miR-337-3p	NM_005066	SFPQ	circRNA	chr1	35649503
MIMAT0000754	hsa-miR-337-3p	NM_005119	THRAP3	circRNA	chr1	36701961
MIMAT0000754	hsa-miR-337-3p	NM_005857	ZMPSTE24	circRNA	chr1	40758958
MIMAT0000754	hsa-miR-337-3p	NM_001172218	SCMH1	circRNA	chr1	41657155
MIMAT0000754	hsa-miR-337-3p	NM_024664	PPCS	circRNA	chr1	42925599
MIMAT0000754	hsa-miR-337-3p	NM_002482	NASP	circRNA	chr1	46083420
MIMAT0000754	hsa-miR-337-3p	NM_021639	GPBP1L1	circRNA	chr1	46093352
MIMAT0000754	hsa-miR-337-3p	NM_001004339	ZYG11A	circRNA	chr1	53329653
MIMAT0000754	hsa-miR-337-3p	NM_017526	LEPROT	circRNA	chr1	65898430
MIMAT0000754	hsa-miR-337-3p	NM_017526	LEPROT	circRNA	chr1	65898909
MIMAT0000754	hsa-miR-337-3p	NM_006417	IFI44	circRNA	chr1	79121047
MIMAT0000754	hsa-miR-337-3p	NM_001162536	RBMXL1	circRNA	chr1	89446400
MIMAT0000754	hsa-miR-337-3p	NM_001938	DR1	circRNA	chr1	93827442
MIMAT0000754	hsa-miR-337-3p	NM_015976	SNX7	circRNA	chr1	99150588
MIMAT0000754	hsa-miR-337-3p	NM_000028	AGL	circRNA	chr1	1E+08
MIMAT0000754	hsa-miR-337-3p	NM_001918	DBT	circRNA	chr1	1.01E+08
MIMAT0000754	hsa-miR-337-3p	NM_017619	RNPC3	circRNA	chr1	1.04E+08
MIMAT0000754	hsa-miR-337-3p	NM_001010935	RAP1A	circRNA	chr1	1.12E+08
MIMAT0000754	hsa-miR-337-3p	NM_017744	ST7L	circRNA	chr1	1.13E+08
MIMAT0000754	hsa-miR-337-3p	NM_138727	ST7L	circRNA	chr1	1.13E+08
MIMAT0000754	hsa-miR-337-3p	hsa_circ_0000107	hsa_circ_002024	circRNA	chr1	1.13E+08
MIMAT0000754	hsa-miR-337-3p	hsa_circ_0000108	hsa_circ_001020	circRNA	chr1	1.13E+08
MIMAT0000754	hsa-miR-337-3p	NM_018364	RSBN1	circRNA	chr1	1.14E+08

MIMAT0000754	hsa-miR-337-3p	NM_198459	DENND2C	circRNA	chr1	1.15E+08
MIMAT0000754	hsa-miR-337-3p	NM_002524	NRAS	circRNA	chr1	1.15E+08
MIMAT0000754	hsa-miR-337-3p	NM_006699	MAN1A2	circRNA	chr1	1.18E+08
MIMAT0000754	hsa-miR-337-3p	hsa_circ_0000116	hsa_circ_002089	circRNA	chr1	1.18E+08
MIMAT0000754	hsa-miR-337-3p	hsa_circ_0000117	hsa_circ_001812	circRNA	chr1	1.18E+08
MIMAT0000754	hsa-miR-337-3p	hsa_circ_0000118	hsa_circ_002173	circRNA	chr1	1.18E+08
MIMAT0000754	hsa-miR-337-3p	hsa_circ_0000119	hsa_circ_002057	circRNA	chr1	1.18E+08
MIMAT0000754	hsa-miR-337-3p	hsa_circ_0000120	hsa_circ_001022	circRNA	chr1	1.18E+08
MIMAT0000754	hsa-miR-337-3p	NM_017686	GDAP2	circRNA	chr1	1.18E+08
MIMAT0000754	hsa-miR-337-3p	NM_001037675	NBPF9	circRNA	chr1	1.45E+08
MIMAT0000754	hsa-miR-337-3p	NM_001039703	NBPF10	circRNA	chr1	1.45E+08
MIMAT0000754	hsa-miR-337-3p	NM_004892	SEC22B	circRNA	chr1	1.45E+08
MIMAT0000754	hsa-miR-337-3p	NM_001039703	NBPF10	circRNA	chr1	1.46E+08
MIMAT0000754	hsa-miR-337-3p	NM_001039703	NBPF10	circRNA	chr1	1.46E+08
MIMAT0000754	hsa-miR-337-3p	NM_014455	RNF115	circRNA	chr1	1.46E+08
MIMAT0000754	hsa-miR-337-3p	hsa_circ_0000125	hsa_circ_001024	circRNA	chr1	1.46E+08
MIMAT0000754	hsa-miR-337-3p	NM_001039703	NBPF10	circRNA	chr1	1.46E+08
MIMAT0000754	hsa-miR-337-3p	NM_001097616	GPR89C	circRNA	chr1	1.46E+08
MIMAT0000754	hsa-miR-337-3p	NM_004326	BCL9	circRNA	chr1	1.46E+08
MIMAT0000754	hsa-miR-337-3p	NM_005399	PRKAB2	circRNA	chr1	1.47E+08
MIMAT0000754	hsa-miR-337-3p	NM_001097616	GPR89C	circRNA	chr1	1.47E+08
MIMAT0000754	hsa-miR-337-3p	NM_004284	CHD1L	circRNA	chr1	1.47E+08
MIMAT0000754	hsa-miR-337-3p	NM_004326	BCL9	circRNA	chr1	1.47E+08
MIMAT0000754	hsa-miR-337-3p	NM_030920	ANP32E	circRNA	chr1	1.5E+08
MIMAT0000754	hsa-miR-337-3p	NM_021960	MCL1	circRNA	chr1	1.51E+08
MIMAT0000754	hsa-miR-337-3p	NM_020127	TUFT1	circRNA	chr1	1.52E+08
MIMAT0000754	hsa-miR-337-3p	NM_020672	S100A14	circRNA	chr1	1.54E+08
MIMAT0000754	hsa-miR-337-3p	NM_001098616	C1orf43	circRNA	chr1	1.54E+08
MIMAT0000754	hsa-miR-337-3p	NM_014847	UBAP2L	circRNA	chr1	1.54E+08
MIMAT0000754	hsa-miR-337-3p	NM_018489	ASH1L	circRNA	chr1	1.55E+08
MIMAT0000754	hsa-miR-337-3p	NM_014949	KIAA0907	circRNA	chr1	1.56E+08
MIMAT0000754	hsa-miR-337-3p	NM_016406	UFC1	circRNA	chr1	1.61E+08
MIMAT0000754	hsa-miR-337-3p	NM_199344	SFT2D2	circRNA	chr1	1.68E+08
MIMAT0000754	hsa-miR-337-3p	NM_022457	RFWD2	circRNA	chr1	1.76E+08
MIMAT0000754	hsa-miR-337-3p	NM_014810	CEP350	circRNA	chr1	1.8E+08
MIMAT0000754	hsa-miR-337-3p	NM_005819	STX6	circRNA	chr1	1.81E+08
MIMAT0000754	hsa-miR-337-3p	NR_023349	TSEN15	circRNA	chr1	1.84E+08
MIMAT0000754	hsa-miR-337-3p	NM_024529	CDC73	circRNA	chr1	1.93E+08
MIMAT0000754	hsa-miR-337-3p	NM_024529	CDC73	circRNA	chr1	1.93E+08
MIMAT0000754	hsa-miR-337-3p	NM_024529	CDC73	circRNA	chr1	1.93E+08
MIMAT0000754	hsa-miR-337-3p	NM_001195215	DENND1B	circRNA	chr1	1.97E+08
MIMAT0000754	hsa-miR-337-3p	NM_006618	KDM5B	circRNA	chr1	2.03E+08
MIMAT0000754	hsa-miR-337-3p	NM_006763	BTG2	circRNA	chr1	2.03E+08
MIMAT0000754	hsa-miR-337-3p	NM_002393	MDM4	circRNA	chr1	2.05E+08
MIMAT0000754	hsa-miR-337-3p	NM_015375	DSTYK	circRNA	chr1	2.05E+08
MIMAT0000754	hsa-miR-337-3p	NM_015375	DSTYK	circRNA	chr1	2.05E+08
MIMAT0000754	hsa-miR-337-3p	NM_014002	IKBKE	circRNA	chr1	2.07E+08
MIMAT0000754	hsa-miR-337-3p	NM_018566	YOD1	circRNA	chr1	2.07E+08
MIMAT0000754	hsa-miR-337-3p	NM_014053	FLVCR1	circRNA	chr1	2.13E+08
MIMAT0000754	hsa-miR-337-3p	NM_012424	RPS6KC1	circRNA	chr1	2.13E+08
MIMAT0000754	hsa-miR-337-3p	NM_005401	PTPN14	circRNA	chr1	2.15E+08
MIMAT0000754	hsa-miR-337-3p	NM_018060	IARS2	circRNA	chr1	2.2E+08
MIMAT0000754	hsa-miR-337-3p	TCONS_l2_000018	TCONS_l2_000018(	circRNA	chr1	2.23E+08
MIMAT0000754	hsa-miR-337-3p	TCONS_l2_000018	TCONS_l2_000018(	circRNA	chr1	2.23E+08
MIMAT0000754	hsa-miR-337-3p	TCONS_l2_000018	TCONS_l2_000018(	circRNA	chr1	2.24E+08
MIMAT0000754	hsa-miR-337-3p	NM_015176	FBXO28	circRNA	chr1	2.24E+08
MIMAT0000754	hsa-miR-337-3p	NR_033184	ZNF678	circRNA	chr1	2.28E+08
MIMAT0000754	hsa-miR-337-3p	NM_014236	GNPAT	circRNA	chr1	2.31E+08
MIMAT0000754	hsa-miR-337-3p	NM_014765	TOMM20	circRNA	chr1	2.35E+08
MIMAT0000754	hsa-miR-337-3p	NM_005465	AKT3	circRNA	chr1	2.44E+08
MIMAT0000754	hsa-miR-337-3p	NM_001167740	SMYD3	circRNA	chr1	2.46E+08
MIMAT0000754	hsa-miR-337-3p	NM_134421	HPCAL1	circRNA	chr2	10531492
MIMAT0000754	hsa-miR-337-3p	NM_024894	NOL10	circRNA	chr2	10742957
MIMAT0000754	hsa-miR-337-3p	NM_015317	PUM2	circRNA	chr2	20530798
MIMAT0000754	hsa-miR-337-3p	NM_147223	NCOA1	circRNA	chr2	24756119

MIMAT0000754	hsa-miR-337-3p	NM_007266	GPN1	circRNA	chr2	27873601
MIMAT0000754	hsa-miR-337-3p	NM_015955	MEMO1	circRNA	chr2	32145899
MIMAT0000754	hsa-miR-337-3p	NM_016441	CRIM1	circRNA	chr2	36776902
MIMAT0000754	hsa-miR-337-3p	NM_016441	CRIM1	circRNA	chr2	36778005
MIMAT0000754	hsa-miR-337-3p	NM_020744	MTA3	circRNA	chr2	42938893
MIMAT0000754	hsa-miR-337-3p	NM_001033557	PPM1B	circRNA	chr2	44428793
MIMAT0000754	hsa-miR-337-3p	NM_177968	PPM1B	circRNA	chr2	44428793
MIMAT0000754	hsa-miR-337-3p	NM_005400	PRKCE	circRNA	chr2	45914837
MIMAT0000754	hsa-miR-337-3p	NM_014614	PSME4	circRNA	chr2	54161771
MIMAT0000754	hsa-miR-337-3p	NM_006759	UGP2	circRNA	chr2	64112850
MIMAT0000754	hsa-miR-337-3p	NM_014755	SERTAD2	circRNA	chr2	64862824
MIMAT0000754	hsa-miR-337-3p	NM_181784	SPRED2	circRNA	chr2	65540089
MIMAT0000754	hsa-miR-337-3p	NM_017880	C2orf42	circRNA	chr2	70392693
MIMAT0000754	hsa-miR-337-3p	NM_022173	TIA1	circRNA	chr2	70436699
MIMAT0000754	hsa-miR-337-3p	NM_016297	PCYOX1	circRNA	chr2	70507694
MIMAT0000754	hsa-miR-337-3p	NM_144993	TET3	circRNA	chr2	74331455
MIMAT0000754	hsa-miR-337-3p	NM_015425	POLR1A	circRNA	chr2	86292586
MIMAT0000754	hsa-miR-337-3p	NM_144706	C2orf15	circRNA	chr2	99802262
MIMAT0000754	hsa-miR-337-3p	NM_002518	NPAS2	circRNA	chr2	1.01E+08
MIMAT0000754	hsa-miR-337-3p	NM_006267	RANBP2	circRNA	chr2	1.09E+08
MIMAT0000754	hsa-miR-337-3p	NM_182588	RGPD4	circRNA	chr2	1.09E+08
MIMAT0000754	hsa-miR-337-3p	NM_006267	RANBP2	circRNA	chr2	1.09E+08
MIMAT0000754	hsa-miR-337-3p	NM_182588	RGPD4	circRNA	chr2	1.09E+08
MIMAT0000754	hsa-miR-337-3p	NM_006267	RANBP2	circRNA	chr2	1.09E+08
MIMAT0000754	hsa-miR-337-3p	NM_182588	RGPD4	circRNA	chr2	1.09E+08
MIMAT0000754	hsa-miR-337-3p	NM_006267	RANBP2	circRNA	chr2	1.09E+08
MIMAT0000754	hsa-miR-337-3p	NM_182588	RGPD4	circRNA	chr2	1.09E+08
MIMAT0000754	hsa-miR-337-3p	NM_006267	RANBP2	circRNA	chr2	1.09E+08
MIMAT0000754	hsa-miR-337-3p	NM_182588	RGPD4	circRNA	chr2	1.09E+08
MIMAT0000754	hsa-miR-337-3p	NM_022336	EDAR	circRNA	chr2	1.1E+08
MIMAT0000754	hsa-miR-337-3p	NM_001037866	RGPD6	circRNA	chr2	1.13E+08
MIMAT0000754	hsa-miR-337-3p	NM_001164463	RGPD8	circRNA	chr2	1.13E+08
MIMAT0000754	hsa-miR-337-3p	NM_022662	ANAPC1	circRNA	chr2	1.13E+08
MIMAT0000754	hsa-miR-337-3p	NM_001037866	RGPD6	circRNA	chr2	1.13E+08
MIMAT0000754	hsa-miR-337-3p	NM_001164463	RGPD8	circRNA	chr2	1.13E+08
MIMAT0000754	hsa-miR-337-3p	NM_001037866	RGPD6	circRNA	chr2	1.13E+08
MIMAT0000754	hsa-miR-337-3p	NM_001164463	RGPD8	circRNA	chr2	1.13E+08
MIMAT0000754	hsa-miR-337-3p	NM_001037866	RGPD6	circRNA	chr2	1.13E+08
MIMAT0000754	hsa-miR-337-3p	NM_001164463	RGPD8	circRNA	chr2	1.13E+08
MIMAT0000754	hsa-miR-337-3p	NM_022662	ANAPC1	circRNA	chr2	1.13E+08
MIMAT0000754	hsa-miR-337-3p	NM_019014	POLR1B	circRNA	chr2	1.13E+08
MIMAT0000754	hsa-miR-337-3p	NM_025181	SLC35F5	circRNA	chr2	1.14E+08
MIMAT0000754	hsa-miR-337-3p	NM_025181	SLC35F5	circRNA	chr2	1.15E+08
MIMAT0000754	hsa-miR-337-3p	NM_001006622	WDR33	circRNA	chr2	1.29E+08
MIMAT0000754	hsa-miR-337-3p	NM_001006622	WDR33	circRNA	chr2	1.29E+08
MIMAT0000754	hsa-miR-337-3p	NM_001006623	WDR33	circRNA	chr2	1.29E+08
MIMAT0000754	hsa-miR-337-3p	NM_018383	WDR33	circRNA	chr2	1.29E+08
MIMAT0000754	hsa-miR-337-3p	NM_014607	UBXN4	circRNA	chr2	1.37E+08
MIMAT0000754	hsa-miR-337-3p	NM_015702	MMADHC	circRNA	chr2	1.5E+08
MIMAT0000754	hsa-miR-337-3p	NM_003628	PKP4	circRNA	chr2	1.59E+08
MIMAT0000754	hsa-miR-337-3p	NM_022826	43897	circRNA	chr2	1.61E+08
MIMAT0000754	hsa-miR-337-3p	NM_004482	GALNT3	circRNA	chr2	1.67E+08
MIMAT0000754	hsa-miR-337-3p	NM_024843	CYBRD1	circRNA	chr2	1.72E+08
MIMAT0000754	hsa-miR-337-3p	NM_002610	PDK1	circRNA	chr2	1.73E+08
MIMAT0000754	hsa-miR-337-3p	NM_002610	PDK1	circRNA	chr2	1.73E+08
MIMAT0000754	hsa-miR-337-3p	NM_133378	TTN	circRNA	chr2	1.79E+08
MIMAT0000754	hsa-miR-337-3p	NM_014905	GLS	circRNA	chr2	1.92E+08
MIMAT0000754	hsa-miR-337-3p	NM_015049	TRAK2	circRNA	chr2	2.02E+08
MIMAT0000754	hsa-miR-337-3p	NM_199229	RPE	circRNA	chr2	2.11E+08
MIMAT0000754	hsa-miR-337-3p	NM_001105537	ZNF142	circRNA	chr2	2.2E+08
MIMAT0000754	hsa-miR-337-3p	NM_004438	EPHA4	circRNA	chr2	2.22E+08
MIMAT0000754	hsa-miR-337-3p	NM_004504	AGFG1	circRNA	chr2	2.28E+08
MIMAT0000754	hsa-miR-337-3p	NM_138402	SP140L	circRNA	chr2	2.31E+08
MIMAT0000754	hsa-miR-337-3p	hsa_circ_0001105	hsa_circ_001735	circRNA	chr2	2.31E+08
MIMAT0000754	hsa-miR-337-3p	hsa_circ_0001106	hsa_circ_001283	circRNA	chr2	2.31E+08

MIMAT0000754	hsa-miR-337-3p	NM_182760	SUMF1	circRNA	chr3	4195095
MIMAT0000754	hsa-miR-337-3p	NM_182760	SUMF1	circRNA	chr3	4237517
MIMAT0000754	hsa-miR-337-3p	NM_001099952	ITPR1	circRNA	chr3	4722246
MIMAT0000754	hsa-miR-337-3p	NM_001168272	ITPR1	circRNA	chr3	4722246
MIMAT0000754	hsa-miR-337-3p	NM_003670	BHLHE40	circRNA	chr3	5025563
MIMAT0000754	hsa-miR-337-3p	NM_001080517	SETD5	circRNA	chr3	9478257
MIMAT0000754	hsa-miR-337-3p	NM_001145395	TSEN2	circRNA	chr3	12581058
MIMAT0000754	hsa-miR-337-3p	NM_033083	EAF1	circRNA	chr3	15482575
MIMAT0000754	hsa-miR-337-3p	NM_000060	BTD	circRNA	chr3	15670710
MIMAT0000754	hsa-miR-337-3p	NM_003615	SLC4A7	circRNA	chr3	27422857
MIMAT0000754	hsa-miR-337-3p	NM_182523	CMC1	circRNA	chr3	28296636
MIMAT0000754	hsa-miR-337-3p	NM_006371	CRTAP	circRNA	chr3	33171471
MIMAT0000754	hsa-miR-337-3p	NM_003149	STAC	circRNA	chr3	36588697
MIMAT0000754	hsa-miR-337-3p	NR_024024	ACAA1	circRNA	chr3	38164361
MIMAT0000754	hsa-miR-337-3p	NM_003420	ZNF35	circRNA	chr3	44692518
MIMAT0000754	hsa-miR-337-3p	NM_014966	DHX30	circRNA	chr3	47890982
MIMAT0000754	hsa-miR-337-3p	NM_001789	CDC25A	circRNA	chr3	48199589
MIMAT0000754	hsa-miR-337-3p	NM_001789	CDC25A	circRNA	chr3	48209347
MIMAT0000754	hsa-miR-337-3p	NM_004947	DOCK3	circRNA	chr3	51420219
MIMAT0000754	hsa-miR-337-3p	NM_000720	CACNA1D	circRNA	chr3	53699768
MIMAT0000754	hsa-miR-337-3p	NM_003392	WNT5A	circRNA	chr3	55501357
MIMAT0000754	hsa-miR-337-3p	NM_003392	WNT5A	circRNA	chr3	55513540
MIMAT0000754	hsa-miR-337-3p	NM_002841	PTPRG	circRNA	chr3	61594800
MIMAT0000754	hsa-miR-337-3p	NM_016206	VGLL3	circRNA	chr3	86991921
MIMAT0000754	hsa-miR-337-3p	NM_032359	C3orf26	circRNA	chr3	99575290
MIMAT0000754	hsa-miR-337-3p	NM_032359	C3orf26	circRNA	chr3	99625227
MIMAT0000754	hsa-miR-337-3p	NR_033437	UMPS	circRNA	chr3	1.24E+08
MIMAT0000754	hsa-miR-337-3p	NM_020733	HEG1	circRNA	chr3	1.25E+08
MIMAT0000754	hsa-miR-337-3p	NM_024628	SLC12A8	circRNA	chr3	1.25E+08
MIMAT0000754	hsa-miR-337-3p	NM_013336	SEC61A1	circRNA	chr3	1.28E+08
MIMAT0000754	hsa-miR-337-3p	NM_006506	RASA2	circRNA	chr3	1.41E+08
MIMAT0000754	hsa-miR-337-3p	NM_006506	RASA2	circRNA	chr3	1.41E+08
MIMAT0000754	hsa-miR-337-3p	NM_001080415	U2SURP	circRNA	chr3	1.43E+08
MIMAT0000754	hsa-miR-337-3p	NM_016094	COMMD2	circRNA	chr3	1.49E+08
MIMAT0000754	hsa-miR-337-3p	NM_024947	PHC3	circRNA	chr3	1.7E+08
MIMAT0000754	hsa-miR-337-3p	NM_005414	SKIL	circRNA	chr3	1.7E+08
MIMAT0000754	hsa-miR-337-3p	NM_024665	TBL1XR1	circRNA	chr3	1.77E+08
MIMAT0000754	hsa-miR-337-3p	NM_024665	TBL1XR1	circRNA	chr3	1.77E+08
MIMAT0000754	hsa-miR-337-3p	NM_022470	ZMAT3	circRNA	chr3	1.79E+08
MIMAT0000754	hsa-miR-337-3p	NM_152240	ZMAT3	circRNA	chr3	1.79E+08
MIMAT0000754	hsa-miR-337-3p	NM_021629	GNB4	circRNA	chr3	1.79E+08
MIMAT0000754	hsa-miR-337-3p	NM_004423	DVL3	circRNA	chr3	1.84E+08
MIMAT0000754	hsa-miR-337-3p	NM_004593	TRA2B	circRNA	chr3	1.86E+08
MIMAT0000754	hsa-miR-337-3p	NM_016306	DNAJB11	circRNA	chr3	1.86E+08
MIMAT0000754	hsa-miR-337-3p	NM_173216	ST6GAL1	circRNA	chr3	1.87E+08
MIMAT0000754	hsa-miR-337-3p	hsa_circ_0068511	hsa_circ_0068511	circRNA	chr3	1.89E+08
MIMAT0000754	hsa-miR-337-3p	hsa_circ_0068512	hsa_circ_0068512	circRNA	chr3	1.89E+08
MIMAT0000754	hsa-miR-337-3p	NM_012287	ACAP2	circRNA	chr3	1.95E+08
MIMAT0000754	hsa-miR-337-3p	NM_001145642	KIAA0226	circRNA	chr3	1.97E+08
MIMAT0000754	hsa-miR-337-3p	NM_005255	GAK	circRNA	chr4	905518
MIMAT0000754	hsa-miR-337-3p	NM_004249	RAB28	circRNA	chr4	13481087
MIMAT0000754	hsa-miR-337-3p	NM_001358	DHX15	circRNA	chr4	24571905
MIMAT0000754	hsa-miR-337-3p	NM_001085400	RELL1	circRNA	chr4	37612911
MIMAT0000754	hsa-miR-337-3p	NM_001085400	RELL1	circRNA	chr4	37613179
MIMAT0000754	hsa-miR-337-3p	NM_001204747	RFC1	circRNA	chr4	39289991
MIMAT0000754	hsa-miR-337-3p	NM_018177	N4BP2	circRNA	chr4	40158921
MIMAT0000754	hsa-miR-337-3p	NM_003215	TEC	circRNA	chr4	48138775
MIMAT0000754	hsa-miR-337-3p	NM_015030	FRYL	circRNA	chr4	48507580
MIMAT0000754	hsa-miR-337-3p	NM_001134937	FIP1L1	circRNA	chr4	54249993
MIMAT0000754	hsa-miR-337-3p	NM_030917	FIP1L1	circRNA	chr4	54249993
MIMAT0000754	hsa-miR-337-3p	hsa_circ_0001411	hsa_circ_002121	circRNA	chr4	54249993
MIMAT0000754	hsa-miR-337-3p	NM_004898	CLOCK	circRNA	chr4	56325336
MIMAT0000754	hsa-miR-337-3p	NM_000938	POLR2B	circRNA	chr4	57860888
MIMAT0000754	hsa-miR-337-3p	NM_032217	ANKRD17	circRNA	chr4	74038015
MIMAT0000754	hsa-miR-337-3p	NM_025074	FRAS1	circRNA	chr4	79464873

MIMAT0000754	hsa-miR-337-3p	NM_031370	HNRNPD	circRNA	chr4	83280776
MIMAT0000754	hsa-miR-337-3p	hsa_circ_0001420	hsa_circ_001161	circRNA	chr4	83280776
MIMAT0000754	hsa-miR-337-3p	NM_022154	SLC39A8	circRNA	chr4	1.03E+08
MIMAT0000754	hsa-miR-337-3p	NM_001127208	TET2	circRNA	chr4	1.06E+08
MIMAT0000754	hsa-miR-337-3p	NM_001031720	GSTCD	circRNA	chr4	1.07E+08
MIMAT0000754	hsa-miR-337-3p	NM_024751	GSTCD	circRNA	chr4	1.07E+08
MIMAT0000754	hsa-miR-337-3p	NM_001100389	TMEM192	circRNA	chr4	1.66E+08
MIMAT0000754	hsa-miR-337-3p	NM_004346	CASP3	circRNA	chr4	1.86E+08
MIMAT0000754	hsa-miR-337-3p	NM_007030	TPPP	circRNA	chr5	661182
MIMAT0000754	hsa-miR-337-3p	NM_015325	KIAA0947	circRNA	chr5	5461512
MIMAT0000754	hsa-miR-337-3p	NM_001040446	MTMR12	circRNA	chr5	32271981
MIMAT0000754	hsa-miR-337-3p	NM_006713	SUB1	circRNA	chr5	32603318
MIMAT0000754	hsa-miR-337-3p	NM_023073	C5orf42	circRNA	chr5	37183050
MIMAT0000754	hsa-miR-337-3p	NM_023073	C5orf42	circRNA	chr5	37226947
MIMAT0000754	hsa-miR-337-3p	NM_012382	TTC33	circRNA	chr5	40714290
MIMAT0000754	hsa-miR-337-3p	NM_002184	IL6ST	circRNA	chr5	55259205
MIMAT0000754	hsa-miR-337-3p	NM_001253699	ERBB2IP	circRNA	chr5	65321360
MIMAT0000754	hsa-miR-337-3p	NM_139168	SREK1	circRNA	chr5	65474680
MIMAT0000754	hsa-miR-337-3p	NM_001098728	GTF2H2C	circRNA	chr5	69707200
MIMAT0000754	hsa-miR-337-3p	NM_017411	SMN2	circRNA	chr5	69707200
MIMAT0000754	hsa-miR-337-3p	NR_033417	GTF2H2B	circRNA	chr5	69707200
MIMAT0000754	hsa-miR-337-3p	TCONS_l2_000234	TCONS_l2_000234(	circRNA	chr5	69707200
MIMAT0000754	hsa-miR-337-3p	NM_005909	MAP1B	circRNA	chr5	71502072
MIMAT0000754	hsa-miR-337-3p	NM_138782	FCHO2	circRNA	chr5	72311487
MIMAT0000754	hsa-miR-337-3p	NM_138782	FCHO2	circRNA	chr5	72384586
MIMAT0000754	hsa-miR-337-3p	NM_015566	FAM169A	circRNA	chr5	74149210
MIMAT0000754	hsa-miR-337-3p	NM_004272	HOMER1	circRNA	chr5	78752707
MIMAT0000754	hsa-miR-337-3p	NM_014639	TTC37	circRNA	chr5	94859435
MIMAT0000754	hsa-miR-337-3p	NM_012081	ELL2	circRNA	chr5	95221427
MIMAT0000754	hsa-miR-337-3p	NM_012081	ELL2	circRNA	chr5	95264457
MIMAT0000754	hsa-miR-337-3p	NM_001012761	RGMB	circRNA	chr5	98131527
MIMAT0000754	hsa-miR-337-3p	NM_015216	PPIP5K2	circRNA	chr5	1.03E+08
MIMAT0000754	hsa-miR-337-3p	NM_152624	DCP2	circRNA	chr5	1.12E+08
MIMAT0000754	hsa-miR-337-3p	NM_016144	COMMD10	circRNA	chr5	1.16E+08
MIMAT0000754	hsa-miR-337-3p	NM_014350	TNFAIP8	circRNA	chr5	1.19E+08
MIMAT0000754	hsa-miR-337-3p	NM_133372	FNIP1	circRNA	chr5	1.31E+08
MIMAT0000754	hsa-miR-337-3p	NM_014423	AFF4	circRNA	chr5	1.32E+08
MIMAT0000754	hsa-miR-337-3p	NM_001135586	C5orf24	circRNA	chr5	1.34E+08
MIMAT0000754	hsa-miR-337-3p	TCONS_00010097	TCONS_00010097	circRNA	chr5	1.35E+08
MIMAT0000754	hsa-miR-337-3p	TCONS_00010097	TCONS_00010097	circRNA	chr5	1.35E+08
MIMAT0000754	hsa-miR-337-3p	NM_001001419	SMAD5	circRNA	chr5	1.36E+08
MIMAT0000754	hsa-miR-337-3p	NM_001001419	SMAD5	circRNA	chr5	1.36E+08
MIMAT0000754	hsa-miR-337-3p	NM_181838	UBE2D2	circRNA	chr5	1.39E+08
MIMAT0000754	hsa-miR-337-3p	NM_017872	THG1L	circRNA	chr5	1.57E+08
MIMAT0000754	hsa-miR-337-3p	NM_022897	RANBP17	circRNA	chr5	1.7E+08
MIMAT0000754	hsa-miR-337-3p	NM_022897	RANBP17	circRNA	chr5	1.71E+08
MIMAT0000754	hsa-miR-337-3p	NM_005520	HNRNPH1	circRNA	chr5	1.79E+08
MIMAT0000754	hsa-miR-337-3p	NM_005520	HNRNPH1	circRNA	chr5	1.79E+08
MIMAT0000754	hsa-miR-337-3p	NM_001195291	SERPINB6	circRNA	chr6	2949240
MIMAT0000754	hsa-miR-337-3p	NM_016495	TBC1D7	circRNA	chr6	13300671
MIMAT0000754	hsa-miR-337-3p	NM_000332	ATXN1	circRNA	chr6	16303848
MIMAT0000754	hsa-miR-337-3p	NM_016255	FAM8A1	circRNA	chr6	17611364
MIMAT0000754	hsa-miR-337-3p	NM_001080480	MBOAT1	circRNA	chr6	20101988
MIMAT0000754	hsa-miR-337-3p	NM_007271	STK38	circRNA	chr6	36475306
MIMAT0000754	hsa-miR-337-3p	NM_152734	C6orf89	circRNA	chr6	36895184
MIMAT0000754	hsa-miR-337-3p	NM_001243186	PIM1	circRNA	chr6	37143010
MIMAT0000754	hsa-miR-337-3p	NM_002648	PIM1	circRNA	chr6	37143010
MIMAT0000754	hsa-miR-337-3p	NM_018322	C6orf64	circRNA	chr6	39073124
MIMAT0000754	hsa-miR-337-3p	NM_001184801	UBR2	circRNA	chr6	42594458
MIMAT0000754	hsa-miR-337-3p	NM_015349	KIAA0240	circRNA	chr6	42773139
MIMAT0000754	hsa-miR-337-3p	NM_001498	GCLC	circRNA	chr6	53372391
MIMAT0000754	hsa-miR-337-3p	NM_001123226	MTO1	circRNA	chr6	74183192
MIMAT0000754	hsa-miR-337-3p	NM_133645	MTO1	circRNA	chr6	74183192
MIMAT0000754	hsa-miR-337-3p	NM_004370	COL12A1	circRNA	chr6	75847193
MIMAT0000754	hsa-miR-337-3p	NM_004370	COL12A1	circRNA	chr6	75901937

MIMAT0000754	hsa-miR-337-3p	NM_001010844	IRAK1BP1	circRNA	chr6	79608151
MIMAT0000754	hsa-miR-337-3p	NM_031469	SH3BGRL2	circRNA	chr6	80409990
MIMAT0000754	hsa-miR-337-3p	NM_031469	SH3BGRL2	circRNA	chr6	80411146
MIMAT0000754	hsa-miR-337-3p	NM_015525	IBTK	circRNA	chr6	82912273
MIMAT0000754	hsa-miR-337-3p	NM_015021	ZNF292	circRNA	chr6	87920953
MIMAT0000754	hsa-miR-337-3p	NM_016021	UBE2J1	circRNA	chr6	90036727
MIMAT0000754	hsa-miR-337-3p	NM_198468	MMS22L	circRNA	chr6	97720606
MIMAT0000754	hsa-miR-337-3p	NM_001004317	LIN28B	circRNA	chr6	1.06E+08
MIMAT0000754	hsa-miR-337-3p	NM_006016	CD164	circRNA	chr6	1.1E+08
MIMAT0000754	hsa-miR-337-3p	NM_002912	REV3L	circRNA	chr6	1.12E+08
MIMAT0000754	hsa-miR-337-3p	NM_002912	REV3L	circRNA	chr6	1.12E+08
MIMAT0000754	hsa-miR-337-3p	NM_002912	REV3L	circRNA	chr6	1.12E+08
MIMAT0000754	hsa-miR-337-3p	NM_020399	GOPC	circRNA	chr6	1.18E+08
MIMAT0000754	hsa-miR-337-3p	NM_020755	SERINC1	circRNA	chr6	1.23E+08
MIMAT0000754	hsa-miR-337-3p	NM_001135648	PTPRK	circRNA	chr6	1.28E+08
MIMAT0000754	hsa-miR-337-3p	NM_004830	MED23	circRNA	chr6	1.32E+08
MIMAT0000754	hsa-miR-337-3p	NM_006620	HBS1L	circRNA	chr6	1.35E+08
MIMAT0000754	hsa-miR-337-3p	hsa_circ_0078031	hsa_circ_0078031	circRNA	chr6	1.42E+08
MIMAT0000754	hsa-miR-337-3p	NM_005389	PCMT1	circRNA	chr6	1.5E+08
MIMAT0000754	hsa-miR-337-3p	NM_020861	ZBTB2	circRNA	chr6	1.52E+08
MIMAT0000754	hsa-miR-337-3p	NM_012454	TIAM2	circRNA	chr6	1.55E+08
MIMAT0000754	hsa-miR-337-3p	NM_020245	TULP4	circRNA	chr6	1.59E+08
MIMAT0000754	hsa-miR-337-3p	NM_020823	TMEM181	circRNA	chr6	1.59E+08
MIMAT0000754	hsa-miR-337-3p	NM_014161	MRPL18	circRNA	chr6	1.6E+08
MIMAT0000754	hsa-miR-337-3p	NM_182552	WDR27	circRNA	chr6	1.7E+08
MIMAT0000754	hsa-miR-337-3p	TCONS_l2_000256	TCONS_l2_000256	circRNA	chr7	5105439
MIMAT0000754	hsa-miR-337-3p	TCONS_l2_000256	TCONS_l2_000256	circRNA	chr7	5238973
MIMAT0000754	hsa-miR-337-3p	NM_004227	CYTH3	circRNA	chr7	6204048
MIMAT0000754	hsa-miR-337-3p	NM_016265	ZNF12	circRNA	chr7	6728704
MIMAT0000754	hsa-miR-337-3p	NM_015132	SNX13	circRNA	chr7	17922232
MIMAT0000754	hsa-miR-337-3p	NM_182762	MACC1	circRNA	chr7	20179389
MIMAT0000754	hsa-miR-337-3p	NM_182762	MACC1	circRNA	chr7	20179405
MIMAT0000754	hsa-miR-337-3p	NM_015060	AVL9	circRNA	chr7	32794003
MIMAT0000754	hsa-miR-337-3p	NM_015060	AVL9	circRNA	chr7	32874474
MIMAT0000754	hsa-miR-337-3p	hsa_circ_0007400	hsa_circ_0007400	circRNA	chr7	32874474
MIMAT0000754	hsa-miR-337-3p	NM_004760	STK17A	circRNA	chr7	43670240
MIMAT0000754	hsa-miR-337-3p	NM_015332	NUDCD3	circRNA	chr7	44446731
MIMAT0000754	hsa-miR-337-3p	NM_022748	TNS3	circRNA	chr7	47578465
MIMAT0000754	hsa-miR-337-3p	NM_001042762	FIGNL1	circRNA	chr7	50511957
MIMAT0000754	hsa-miR-337-3p	NM_178558	ZNF680	circRNA	chr7	63980584
MIMAT0000754	hsa-miR-337-3p	NM_016220	ZNF107	circRNA	chr7	64146284
MIMAT0000754	hsa-miR-337-3p	NM_016220	ZNF107	circRNA	chr7	64151692
MIMAT0000754	hsa-miR-337-3p	NM_152626	ZNF92	circRNA	chr7	64820428
MIMAT0000754	hsa-miR-337-3p	NR_027393	INTS4L1	circRNA	chr7	64820428
MIMAT0000754	hsa-miR-337-3p	NR_033416	CCT6P3	circRNA	chr7	64820428
MIMAT0000754	hsa-miR-337-3p	TCONS_l2_000273	TCONS_l2_000273	circRNA	chr7	66038150
MIMAT0000754	hsa-miR-337-3p	NM_153033	KCTD7	circRNA	chr7	66222538
MIMAT0000754	hsa-miR-337-3p	NM_003388	CLIP2	circRNA	chr7	72687131
MIMAT0000754	hsa-miR-337-3p	NM_032999	GTF2I	circRNA	chr7	72687131
MIMAT0000754	hsa-miR-337-3p	NR_040582	STAG3L3	circRNA	chr7	72935100
MIMAT0000754	hsa-miR-337-3p	NM_003388	CLIP2	circRNA	chr7	73217299
MIMAT0000754	hsa-miR-337-3p	NM_032999	GTF2I	circRNA	chr7	73217299
MIMAT0000754	hsa-miR-337-3p	NM_003388	CLIP2	circRNA	chr7	73217388
MIMAT0000754	hsa-miR-337-3p	NM_032999	GTF2I	circRNA	chr7	73217388
MIMAT0000754	hsa-miR-337-3p	NM_001099415	POM121C	circRNA	chr7	75651104
MIMAT0000754	hsa-miR-337-3p	NM_005338	HIP1	circRNA	chr7	75651104
MIMAT0000754	hsa-miR-337-3p	NM_198467	RSBN1L	circRNA	chr7	77402546
MIMAT0000754	hsa-miR-337-3p	NM_021723	ADAM22	circRNA	chr7	87826276
MIMAT0000754	hsa-miR-337-3p	NM_012395	CDK14	circRNA	chr7	90837069
MIMAT0000754	hsa-miR-337-3p	NM_017667	CCDC132	circRNA	chr7	92921078
MIMAT0000754	hsa-miR-337-3p	NM_000089	COL1A2	circRNA	chr7	94057713
MIMAT0000754	hsa-miR-337-3p	NM_014916	LMTK2	circRNA	chr7	97835521
MIMAT0000754	hsa-miR-337-3p	NM_003439	ZKSCAN1	circRNA	chr7	99631114
MIMAT0000754	hsa-miR-337-3p	NM_003302	TRIP6	circRNA	chr7	1E+08
MIMAT0000754	hsa-miR-337-3p	NM_000602	SERPINE1	circRNA	chr7	1.01E+08

MIMAT0000754	hsa-miR-337-3p	NR_024199	CBLL1	circRNA	chr7	1.07E+08
MIMAT0000754	hsa-miR-337-3p	NM_001127500	MET	circRNA	chr7	1.16E+08
MIMAT0000754	hsa-miR-337-3p	NM_003941	WASL	circRNA	chr7	1.23E+08
MIMAT0000754	hsa-miR-337-3p	NM_176814	ZNF800	circRNA	chr7	1.27E+08
MIMAT0000754	hsa-miR-337-3p	NM_001199672	CALU	circRNA	chr7	1.28E+08
MIMAT0000754	hsa-miR-337-3p	hsa_circ_0001748	hsa_circ_002039	circRNA	chr7	1.33E+08
MIMAT0000754	hsa-miR-337-3p	NM_016019	LUC7L2	circRNA	chr7	1.39E+08
MIMAT0000754	hsa-miR-337-3p	TCONS_l2_000269	TCONS_l2_000269	circRNA	chr7	1.5E+08
MIMAT0000754	hsa-miR-337-3p	NM_020728	ESYT2	circRNA	chr7	1.59E+08
MIMAT0000754	hsa-miR-337-3p	NM_182643	DLC1	circRNA	chr8	12942317
MIMAT0000754	hsa-miR-337-3p	NM_016353	ZDHHC2	circRNA	chr8	17079776
MIMAT0000754	hsa-miR-337-3p	NM_016353	ZDHHC2	circRNA	chr8	17080023
MIMAT0000754	hsa-miR-337-3p	NM_001722	POLR3D	circRNA	chr8	22108425
MIMAT0000754	hsa-miR-337-3p	NM_004331	BNIP3L	circRNA	chr8	26268545
MIMAT0000754	hsa-miR-337-3p	NM_032664	FUT10	circRNA	chr8	33246884
MIMAT0000754	hsa-miR-337-3p	NM_004874	BAG4	circRNA	chr8	38069640
MIMAT0000754	hsa-miR-337-3p	NM_001134296	AP3M2	circRNA	chr8	42026844
MIMAT0000754	hsa-miR-337-3p	NM_024831	TGS1	circRNA	chr8	56737949
MIMAT0000754	hsa-miR-337-3p	NM_024790	CSPP1	circRNA	chr8	68002369
MIMAT0000754	hsa-miR-337-3p	NM_006421	ARFGEF1	circRNA	chr8	68150683
MIMAT0000754	hsa-miR-337-3p	NM_024721	ZFHX4	circRNA	chr8	77776945
MIMAT0000754	hsa-miR-337-3p	NM_001205263	RAD54B	circRNA	chr8	95403887
MIMAT0000754	hsa-miR-337-3p	NM_017890	VPS13B	circRNA	chr8	1E+08
MIMAT0000754	hsa-miR-337-3p	NR_027427	TATDN1	circRNA	chr8	1.26E+08
MIMAT0000754	hsa-miR-337-3p	TCONS_00015354	TCONS_00015354	circRNA	chr8	1.29E+08
MIMAT0000754	hsa-miR-337-3p	NM_001247996	ASAP1	circRNA	chr8	1.31E+08
MIMAT0000754	hsa-miR-337-3p	NM_001247996	ASAP1	circRNA	chr8	1.31E+08
MIMAT0000754	hsa-miR-337-3p	NM_001247996	ASAP1	circRNA	chr8	1.31E+08
MIMAT0000754	hsa-miR-337-3p	NM_001247996	ASAP1	circRNA	chr8	1.31E+08
MIMAT0000754	hsa-miR-337-3p	NM_001247996	ASAP1	circRNA	chr8	1.31E+08
MIMAT0000754	hsa-miR-337-3p	NM_001247996	ASAP1	circRNA	chr8	1.31E+08
MIMAT0000754	hsa-miR-337-3p	NM_012154	EIF2C2	circRNA	chr8	1.42E+08
MIMAT0000754	hsa-miR-337-3p	NM_001199649	PTK2	circRNA	chr8	1.42E+08
MIMAT0000754	hsa-miR-337-3p	NM_001199649	PTK2	circRNA	chr8	1.42E+08
MIMAT0000754	hsa-miR-337-3p	NM_153186	KANK1	circRNA	chr9	548582
MIMAT0000754	hsa-miR-337-3p	TCONS_l2_000291	TCONS_l2_000291	circRNA	chr9	2495206
MIMAT0000754	hsa-miR-337-3p	NM_001042413	GLIS3	circRNA	chr9	4266478
MIMAT0000754	hsa-miR-337-3p	NM_004170	SLC1A1	circRNA	chr9	4587218
MIMAT0000754	hsa-miR-337-3p	NM_016282	AK3	circRNA	chr9	4711432
MIMAT0000754	hsa-miR-337-3p	NM_004972	JAK2	circRNA	chr9	5127436
MIMAT0000754	hsa-miR-337-3p	NM_033222	PSIP1	circRNA	chr9	15464778
MIMAT0000754	hsa-miR-337-3p	NM_017645	HAUS6	circRNA	chr9	19087108
MIMAT0000754	hsa-miR-337-3p	NM_001539	DNAJA1	circRNA	chr9	33026547
MIMAT0000754	hsa-miR-337-3p	hsa_circ_0001859	hsa_circ_001783	circRNA	chr9	37089261
MIMAT0000754	hsa-miR-337-3p	NM_032226	ZCCHC7	circRNA	chr9	37130284
MIMAT0000754	hsa-miR-337-3p	NM_032226	ZCCHC7	circRNA	chr9	37176752
MIMAT0000754	hsa-miR-337-3p	NM_032226	ZCCHC7	circRNA	chr9	37192166
MIMAT0000754	hsa-miR-337-3p	NM_015110	SMC5	circRNA	chr9	72968363
MIMAT0000754	hsa-miR-337-3p	NM_001025780	FAM108B1	circRNA	chr9	74480417
MIMAT0000754	hsa-miR-337-3p	NM_033305	VPS13A	circRNA	chr9	79835236
MIMAT0000754	hsa-miR-337-3p	NM_152573	RASEF	circRNA	chr9	85672211
MIMAT0000754	hsa-miR-337-3p	hsa_circ_0008720	hsa_circ_0008720	circRNA	chr9	98747807
MIMAT0000754	hsa-miR-337-3p	NM_015469	NIPSNAP3A	circRNA	chr9	1.08E+08
MIMAT0000754	hsa-miR-337-3p	hsa_circ_0001878	hsa_circ_001203	circRNA	chr9	1.08E+08
MIMAT0000754	hsa-miR-337-3p	hsa_circ_0001879	hsa_circ_001867	circRNA	chr9	1.08E+08
MIMAT0000754	hsa-miR-337-3p	NM_080546	SLC44A1	circRNA	chr9	1.08E+08
MIMAT0000754	hsa-miR-337-3p	NM_032012	C9orf5	circRNA	chr9	1.12E+08
MIMAT0000754	hsa-miR-337-3p	NM_001735	C5	circRNA	chr9	1.24E+08
MIMAT0000754	hsa-miR-337-3p	NM_018201	TBC1D13	circRNA	chr9	1.32E+08
MIMAT0000754	hsa-miR-337-3p	NM_014506	TOR1B	circRNA	chr9	1.33E+08
MIMAT0000754	hsa-miR-337-3p	NM_013318	PRRC2B	circRNA	chr9	1.34E+08
MIMAT0000754	hsa-miR-337-3p	NM_033161	SURF4	circRNA	chr9	1.36E+08
MIMAT0000754	hsa-miR-337-3p	NM_020385	REXO4	circRNA	chr9	1.36E+08
MIMAT0000754	hsa-miR-337-3p	NM_001494	GDI2	circRNA	chr10	5842666
MIMAT0000754	hsa-miR-337-3p	NM_004808	NMT2	circRNA	chr10	15151760

MIMAT0000754	hsa-miR-337-3p	hsa_circ_0017904	hsa_circ_0017904	circRNA	chr10	17882673
MIMAT0000754	hsa-miR-337-3p	NM_005028	PIP4K2A	circRNA	chr10	22825255
MIMAT0000754	hsa-miR-337-3p	NM_005028	PIP4K2A	circRNA	chr10	22862253
MIMAT0000754	hsa-miR-337-3p	NM_014915	ANKRD26	circRNA	chr10	27352932
MIMAT0000754	hsa-miR-337-3p	NM_001128128	ZEB1	circRNA	chr10	31661701
MIMAT0000754	hsa-miR-337-3p	hsa_circ_0000227	hsa_circ_001214	circRNA	chr10	31661701
MIMAT0000754	hsa-miR-337-3p	NM_004521	KIF5B	circRNA	chr10	32299535
MIMAT0000754	hsa-miR-337-3p	NM_004521	KIF5B	circRNA	chr10	32300131
MIMAT0000754	hsa-miR-337-3p	NM_004521	KIF5B	circRNA	chr10	32322844
MIMAT0000754	hsa-miR-337-3p	NM_025209	EPC1	circRNA	chr10	32573715
MIMAT0000754	hsa-miR-337-3p	NM_145012	CCNY	circRNA	chr10	35860375
MIMAT0000754	hsa-miR-337-3p	NM_145011	ZNF25	circRNA	chr10	38240304
MIMAT0000754	hsa-miR-337-3p	NM_006955	ZNF33B	circRNA	chr10	43087885
MIMAT0000754	hsa-miR-337-3p	NM_007021	C10orf10	circRNA	chr10	45472498
MIMAT0000754	hsa-miR-337-3p	TCONS_l2_000030	TCONS_l2_000030	circRNA	chr10	48514675
MIMAT0000754	hsa-miR-337-3p	TCONS_l2_000039	TCONS_l2_000039	circRNA	chr10	48514675
MIMAT0000754	hsa-miR-337-3p	NM_147156	SGMS1	circRNA	chr10	52103537
MIMAT0000754	hsa-miR-337-3p	NM_147156	SGMS1	circRNA	chr10	52193268
MIMAT0000754	hsa-miR-337-3p	NM_032776	JMJD1C	circRNA	chr10	64943297
MIMAT0000754	hsa-miR-337-3p	NM_032776	JMJD1C	circRNA	chr10	65164938
MIMAT0000754	hsa-miR-337-3p	NM_004728	DDX21	circRNA	chr10	70743666
MIMAT0000754	hsa-miR-337-3p	NM_001142648	SAR1A	circRNA	chr10	71910093
MIMAT0000754	hsa-miR-337-3p	NM_001142595	P4HA1	circRNA	chr10	74834614
MIMAT0000754	hsa-miR-337-3p	NM_001142596	P4HA1	circRNA	chr10	74834614
MIMAT0000754	hsa-miR-337-3p	NM_001123	ADK	circRNA	chr10	75993327
MIMAT0000754	hsa-miR-337-3p	hsa_circ_0000249	hsa_circ_001838	circRNA	chr10	76214325
MIMAT0000754	hsa-miR-337-3p	hsa_circ_0000249	hsa_circ_001838	circRNA	chr10	76273892
MIMAT0000754	hsa-miR-337-3p	NM_032373	PCGF5	circRNA	chr10	93040192
MIMAT0000754	hsa-miR-337-3p	NM_032373	PCGF5	circRNA	chr10	93040626
MIMAT0000754	hsa-miR-337-3p	NM_003972	BTAF1	circRNA	chr10	93771127
MIMAT0000754	hsa-miR-337-3p	NM_014912	CPEB3	circRNA	chr10	93809755
MIMAT0000754	hsa-miR-337-3p	NM_014803	ZNF518A	circRNA	chr10	97919599
MIMAT0000754	hsa-miR-337-3p	NM_002629	PGAM1	circRNA	chr10	99192943
MIMAT0000754	hsa-miR-337-3p	NM_017902	HIF1AN	circRNA	chr10	1.02E+08
MIMAT0000754	hsa-miR-337-3p	NM_007373	SHOC2	circRNA	chr10	1.13E+08
MIMAT0000754	hsa-miR-337-3p	NM_001244949	GPAM	circRNA	chr10	1.14E+08
MIMAT0000754	hsa-miR-337-3p	NM_145206	VTI1A	circRNA	chr10	1.15E+08
MIMAT0000754	hsa-miR-337-3p	NM_153810	C10orf46	circRNA	chr10	1.2E+08
MIMAT0000754	hsa-miR-337-3p	NM_001033925	TIAL1	circRNA	chr10	1.21E+08
MIMAT0000754	hsa-miR-337-3p	NM_001033925	TIAL1	circRNA	chr10	1.21E+08
MIMAT0000754	hsa-miR-337-3p	NM_032182	FAM175B	circRNA	chr10	1.27E+08
MIMAT0000754	hsa-miR-337-3p	NM_021961	TEAD1	circRNA	chr11	12963554
MIMAT0000754	hsa-miR-337-3p	NM_021961	TEAD1	circRNA	chr11	12965974
MIMAT0000754	hsa-miR-337-3p	NM_001033506	CSTF3	circRNA	chr11	33163414
MIMAT0000754	hsa-miR-337-3p	NR_024625	API5	circRNA	chr11	43364575
MIMAT0000754	hsa-miR-337-3p	NM_016142	HSD17B12	circRNA	chr11	43861574
MIMAT0000754	hsa-miR-337-3p	NM_014342	MTCH2	circRNA	chr11	47639306
MIMAT0000754	hsa-miR-337-3p	NM_015231	NUP160	circRNA	chr11	47861931
MIMAT0000754	hsa-miR-337-3p	NR_026593	TMX2	circRNA	chr11	57505326
MIMAT0000754	hsa-miR-337-3p	NM_024811	CPSF7	circRNA	chr11	61188000
MIMAT0000754	hsa-miR-337-3p	NM_006185	NUMA1	circRNA	chr11	71773197
MIMAT0000754	hsa-miR-337-3p	NM_173582	PGM2L1	circRNA	chr11	74043813
MIMAT0000754	hsa-miR-337-3p	NM_018367	ACER3	circRNA	chr11	76621983
MIMAT0000754	hsa-miR-337-3p	NM_007166	PICALM	circRNA	chr11	85669912
MIMAT0000754	hsa-miR-337-3p	NM_144664	FAM76B	circRNA	chr11	95504013
MIMAT0000754	hsa-miR-337-3p	NM_144664	FAM76B	circRNA	chr11	95504126
MIMAT0000754	hsa-miR-337-3p	hsa_circ_0000353	hsa_circ_001720	circRNA	chr11	1.02E+08
MIMAT0000754	hsa-miR-337-3p	NM_001077691	ALG9	circRNA	chr11	1.12E+08
MIMAT0000754	hsa-miR-337-3p	NM_024740	ALG9	circRNA	chr11	1.12E+08
MIMAT0000754	hsa-miR-337-3p	NM_001197104	MLL	circRNA	chr11	1.18E+08
MIMAT0000754	hsa-miR-337-3p	NM_003105	SORL1	circRNA	chr11	1.22E+08
MIMAT0000754	hsa-miR-337-3p	NM_032873	UBASH3B	circRNA	chr11	1.23E+08
MIMAT0000754	hsa-miR-337-3p	NM_004879	EI24	circRNA	chr11	1.25E+08
MIMAT0000754	hsa-miR-337-3p	NM_001243597	CDON	circRNA	chr11	1.26E+08
MIMAT0000754	hsa-miR-337-3p	NM_001039661	TIRAP	circRNA	chr11	1.26E+08

MIMAT0000754	hsa-miR-337-3p	NM_006931	SLC2A3	circRNA	chr12	8073680
MIMAT0000754	hsa-miR-337-3p	NM_020734	RIMKLB	circRNA	chr12	8927785
MIMAT0000754	hsa-miR-337-3p	NM_030640	DUSP16	circRNA	chr12	12628688
MIMAT0000754	hsa-miR-337-3p	NM_177444	PPFIBP1	circRNA	chr12	27461356
MIMAT0000754	hsa-miR-337-3p	NM_177444	PPFIBP1	circRNA	chr12	27475531
MIMAT0000754	hsa-miR-337-3p	NM_018318	CCDC91	circRNA	chr12	28393821
MIMAT0000754	hsa-miR-337-3p	hsa_circ_0000386	hsa_circ_002027	circRNA	chr12	28393821
MIMAT0000754	hsa-miR-337-3p	NM_018318	CCDC91	circRNA	chr12	28702847
MIMAT0000754	hsa-miR-337-3p	NM_153634	CPNE8	circRNA	chr12	39047326
MIMAT0000754	hsa-miR-337-3p	NM_030674	SLC38A1	circRNA	chr12	46590894
MIMAT0000754	hsa-miR-337-3p	NM_001126103	RACGAP1	circRNA	chr12	50388096
MIMAT0000754	hsa-miR-337-3p	NM_138473	SP1	circRNA	chr12	53806478
MIMAT0000754	hsa-miR-337-3p	NM_138473	SP1	circRNA	chr12	53808374
MIMAT0000754	hsa-miR-337-3p	NM_018457	PRR13	circRNA	chr12	53847641
MIMAT0000754	hsa-miR-337-3p	NM_001127322	CBX5	circRNA	chr12	54632245
MIMAT0000754	hsa-miR-337-3p	NM_001127322	CBX5	circRNA	chr12	54632874
MIMAT0000754	hsa-miR-337-3p	NM_001029	RPS26	circRNA	chr12	56435882
MIMAT0000754	hsa-miR-337-3p	hsa_circ_0027246	hsa_circ_0027246	circRNA	chr12	57800287
MIMAT0000754	hsa-miR-337-3p	hsa_circ_0027246	hsa_circ_0027246	circRNA	chr12	57810167
MIMAT0000754	hsa-miR-337-3p	hsa_circ_0027246	hsa_circ_0027246	circRNA	chr12	57814776
MIMAT0000754	hsa-miR-337-3p	NM_031435	THAP2	circRNA	chr12	72074247
MIMAT0000754	hsa-miR-337-3p	NM_020841	OSBPL8	circRNA	chr12	76746129
MIMAT0000754	hsa-miR-337-3p	NM_014903	NAV3	circRNA	chr12	78582443
MIMAT0000754	hsa-miR-337-3p	NM_001135805	SYT1	circRNA	chr12	79843292
MIMAT0000754	hsa-miR-337-3p	NM_003297	NR2C1	circRNA	chr12	95415490
MIMAT0000754	hsa-miR-337-3p	NM_013320	HCFC2	circRNA	chr12	1.04E+08
MIMAT0000754	hsa-miR-337-3p	NM_013320	HCFC2	circRNA	chr12	1.04E+08
MIMAT0000754	hsa-miR-337-3p	NM_032148	SLC41A2	circRNA	chr12	1.05E+08
MIMAT0000754	hsa-miR-337-3p	NM_015335	MED13L	circRNA	chr12	1.16E+08
MIMAT0000754	hsa-miR-337-3p	NM_015335	MED13L	circRNA	chr12	1.17E+08
MIMAT0000754	hsa-miR-337-3p	NM_014730	MLEC	circRNA	chr12	1.21E+08
MIMAT0000754	hsa-miR-337-3p	NM_014730	MLEC	circRNA	chr12	1.21E+08
MIMAT0000754	hsa-miR-337-3p	NM_001170543	PGAM5	circRNA	chr12	1.33E+08
MIMAT0000754	hsa-miR-337-3p	NM_015114	ANKLE2	circRNA	chr12	1.33E+08
MIMAT0000754	hsa-miR-337-3p	TCONS_l2_000070	TCONS_l2_000070	circRNA	chr13	19870554
MIMAT0000754	hsa-miR-337-3p	TCONS_l2_000070	TCONS_l2_000070	circRNA	chr13	20138594
MIMAT0000754	hsa-miR-337-3p	TCONS_l2_000070	TCONS_l2_000070	circRNA	chr13	20264023
MIMAT0000754	hsa-miR-337-3p	NM_014572	LATS2	circRNA	chr13	21620315
MIMAT0000754	hsa-miR-337-3p	NM_153251	ZDHHC20	circRNA	chr13	22025232
MIMAT0000754	hsa-miR-337-3p	NM_014089	NUPL1	circRNA	chr13	25916446
MIMAT0000754	hsa-miR-337-3p	NM_175854	PAN3	circRNA	chr13	28743740
MIMAT0000754	hsa-miR-337-3p	NM_014887	N4BP2L2	circRNA	chr13	33083760
MIMAT0000754	hsa-miR-337-3p	NM_014887	N4BP2L2	circRNA	chr13	33110789
MIMAT0000754	hsa-miR-337-3p	NM_015087	SPG20	circRNA	chr13	36909357
MIMAT0000754	hsa-miR-337-3p	NM_005780	LHFP	circRNA	chr13	39917215
MIMAT0000754	hsa-miR-337-3p	NM_178009	DGKH	circRNA	chr13	42694693
MIMAT0000754	hsa-miR-337-3p	NM_015070	ZC3H13	circRNA	chr13	46543861
MIMAT0000754	hsa-miR-337-3p	NM_000321	RB1	circRNA	chr13	48951086
MIMAT0000754	hsa-miR-337-3p	NM_000321	RB1	circRNA	chr13	49055146
MIMAT0000754	hsa-miR-337-3p	NM_012141	INTS6	circRNA	chr13	51952481
MIMAT0000754	hsa-miR-337-3p	NM_019080	NDFIP2	circRNA	chr13	80127815
MIMAT0000754	hsa-miR-337-3p	NM_001130048	DOCK9	circRNA	chr13	99446716
MIMAT0000754	hsa-miR-337-3p	NM_007192	SUPT16H	circRNA	chr14	21826138
MIMAT0000754	hsa-miR-337-3p	hsa_circ_0000522	hsa_circ_000586	circRNA	chr14	21826138
MIMAT0000754	hsa-miR-337-3p	NM_032846	RAB2B	circRNA	chr14	21930543
MIMAT0000754	hsa-miR-337-3p	NM_014828	TOX4	circRNA	chr14	21956749
MIMAT0000754	hsa-miR-337-3p	NM_024328	THTPA	circRNA	chr14	24028295
MIMAT0000754	hsa-miR-337-3p	NM_001030055	ARHGAP5	circRNA	chr14	32561496
MIMAT0000754	hsa-miR-337-3p	NM_013448	BAZ1A	circRNA	chr14	35270356
MIMAT0000754	hsa-miR-337-3p	NM_017658	KLHL28	circRNA	chr14	45394939
MIMAT0000754	hsa-miR-337-3p	NM_004713	NEMF	circRNA	chr14	50064239
MIMAT0000754	hsa-miR-337-3p	NM_002408	MGAT2	circRNA	chr14	50090394
MIMAT0000754	hsa-miR-337-3p	NM_001130701	STYX	circRNA	chr14	53241579
MIMAT0000754	hsa-miR-337-3p	NM_145251	STYX	circRNA	chr14	53241579
MIMAT0000754	hsa-miR-337-3p	TCONS_l2_000077	TCONS_l2_000077	circRNA	chr14	53630284

MIMAT0000754	hsa-miR-337-3p	NM_005776	CNIH	circRNA	chr14	54894180
MIMAT0000754	hsa-miR-337-3p	NM_015589	SAMD4A	circRNA	chr14	55117052
MIMAT0000754	hsa-miR-337-3p	NM_015589	SAMD4A	circRNA	chr14	55133151
MIMAT0000754	hsa-miR-337-3p	NM_182926	KTN1	circRNA	chr14	56078737
MIMAT0000754	hsa-miR-337-3p	NM_001244189	KIAA0586	circRNA	chr14	58956882
MIMAT0000754	hsa-miR-337-3p	NM_017420	SIX4	circRNA	chr14	61180775
MIMAT0000754	hsa-miR-337-3p	NM_003082	SNAPC1	circRNA	chr14	62235354
MIMAT0000754	hsa-miR-337-3p	NM_182914	SYNE2	circRNA	chr14	64516416
MIMAT0000754	hsa-miR-337-3p	NM_021979	HSPA2	circRNA	chr14	65009801
MIMAT0000754	hsa-miR-337-3p	NM_016026	RDH11	circRNA	chr14	68143769
MIMAT0000754	hsa-miR-337-3p	NM_018373	SYNJ2BP	circRNA	chr14	70839821
MIMAT0000754	hsa-miR-337-3p	NM_014982	PCNX	circRNA	chr14	71577946
MIMAT0000754	hsa-miR-337-3p	hsa_circ_0002722	hsa_circ_0002722	circRNA	chr14	71868873
MIMAT0000754	hsa-miR-337-3p	hsa_circ_0000552	hsa_circ_000594	circRNA	chr14	71911112
MIMAT0000754	hsa-miR-337-3p	hsa_circ_0006783	hsa_circ_0006783	circRNA	chr14	71911112
MIMAT0000754	hsa-miR-337-3p	NM_000153	GALC	circRNA	chr14	88411907
MIMAT0000754	hsa-miR-337-3p	NM_018319	TDP1	circRNA	chr14	90489002
MIMAT0000754	hsa-miR-337-3p	NM_001002860	BTBD7	circRNA	chr14	93705735
MIMAT0000754	hsa-miR-337-3p	NM_003384	VRK1	circRNA	chr14	97326976
MIMAT0000754	hsa-miR-337-3p	hsa_circ_0000566	hsa_circ_002156	circRNA	chr14	97326976
MIMAT0000754	hsa-miR-337-3p	NM_005552	KLC1	circRNA	chr14	1.04E+08
MIMAT0000754	hsa-miR-337-3p	NM_032374	APOPT1	circRNA	chr14	1.04E+08
MIMAT0000754	hsa-miR-337-3p	NM_003257	TJP1	circRNA	chr15	30100883
MIMAT0000754	hsa-miR-337-3p	NM_181077	GOLGA8A	circRNA	chr15	34677305
MIMAT0000754	hsa-miR-337-3p	NM_014106	ZNF770	circRNA	chr15	35270895
MIMAT0000754	hsa-miR-337-3p	NM_170589	CASC5	circRNA	chr15	40947121
MIMAT0000754	hsa-miR-337-3p	NM_018145	FAM82A2	circRNA	chr15	41028396
MIMAT0000754	hsa-miR-337-3p	NM_007236	CHP	circRNA	chr15	41573862
MIMAT0000754	hsa-miR-337-3p	NM_015289	VPS39	circRNA	chr15	42492108
MIMAT0000754	hsa-miR-337-3p	NM_001159280	ADAL	circRNA	chr15	43645986
MIMAT0000754	hsa-miR-337-3p	NM_138423	CASC4	circRNA	chr15	44620921
MIMAT0000754	hsa-miR-337-3p	hsa_circ_0000595	hsa_circ_000851	circRNA	chr15	44620921
MIMAT0000754	hsa-miR-337-3p	NM_017672	TRPM7	circRNA	chr15	50906415
MIMAT0000754	hsa-miR-337-3p	NM_002748	MAPK6	circRNA	chr15	52358056
MIMAT0000754	hsa-miR-337-3p	NM_017610	RNF111	circRNA	chr15	59350727
MIMAT0000754	hsa-miR-337-3p	NM_004998	MYO1E	circRNA	chr15	59428617
MIMAT0000754	hsa-miR-337-3p	NM_001018088	VPS13C	circRNA	chr15	62300896
MIMAT0000754	hsa-miR-337-3p	hsa_circ_0000607	hsa_circ_001567	circRNA	chr15	62300896
MIMAT0000754	hsa-miR-337-3p	NM_003922	HERC1	circRNA	chr15	63935736
MIMAT0000754	hsa-miR-337-3p	NM_022048	CSNK1G1	circRNA	chr15	64592717
MIMAT0000754	hsa-miR-337-3p	NM_024817	THSD4	circRNA	chr15	72074785
MIMAT0000754	hsa-miR-337-3p	NM_005724	TSPAN3	circRNA	chr15	77338561
MIMAT0000754	hsa-miR-337-3p	NM_024776	PEAK1	circRNA	chr15	77404519
MIMAT0000754	hsa-miR-337-3p	NM_016332	SEPX1	circRNA	chr16	1988323
MIMAT0000754	hsa-miR-337-3p	NM_000548	TSC2	circRNA	chr16	2110719
MIMAT0000754	hsa-miR-337-3p	NM_014287	NOMO1	circRNA	chr16	16176414
MIMAT0000754	hsa-miR-337-3p	NM_014287	NOMO1	circRNA	chr16	16234411
MIMAT0000754	hsa-miR-337-3p	NM_015092	SMG1	circRNA	chr16	18847255
MIMAT0000754	hsa-miR-337-3p	NM_003366	UQCRC2	circRNA	chr16	21976782
MIMAT0000754	hsa-miR-337-3p	hsa_circ_0038902	hsa_circ_0038902	circRNA	chr16	29467313
MIMAT0000754	hsa-miR-337-3p	NM_001031827	BOLA2	circRNA	chr16	29814971
MIMAT0000754	hsa-miR-337-3p	NR_002453	LOC595101	circRNA	chr16	29814971
MIMAT0000754	hsa-miR-337-3p	TCONS_l2_000104	TCONS_l2_000104[illegible]	circRNA	chr16	29814971
MIMAT0000754	hsa-miR-337-3p	NM_003414	ZNF267	circRNA	chr16	31926048
MIMAT0000754	hsa-miR-337-3p	NM_015247	CYLD	circRNA	chr16	50783732
MIMAT0000754	hsa-miR-337-3p	NM_016284	CNOT1	circRNA	chr16	58590859
MIMAT0000754	hsa-miR-337-3p	NM_206999	CNOT1	circRNA	chr16	58590859
MIMAT0000754	hsa-miR-337-3p	NM_001018159	NAE1	circRNA	chr16	66839894
MIMAT0000754	hsa-miR-337-3p	NM_020786	PDP2	circRNA	chr16	66921056
MIMAT0000754	hsa-miR-337-3p	NM_001076785	SLC7A6	circRNA	chr16	68331372
MIMAT0000754	hsa-miR-337-3p	NM_001076785	SLC7A6	circRNA	chr16	68335551
MIMAT0000754	hsa-miR-337-3p	NM_015020	PHLPP2	circRNA	chr16	71679313
MIMAT0000754	hsa-miR-337-3p	NM_015020	PHLPP2	circRNA	chr16	71681786
MIMAT0000754	hsa-miR-337-3p	NR_027264	GLG1	circRNA	chr16	74481873
MIMAT0000754	hsa-miR-337-3p	NM_005153	USP10	circRNA	chr16	84753229

MIMAT0000754	hsa-miR-337-3p	NM_022818	MAP1LC3B	circRNA	chr16	87438299
MIMAT0000754	hsa-miR-337-3p	NM_001080779	MYO1C	circRNA	chr17	1368696
MIMAT0000754	hsa-miR-337-3p	NM_002532	NUP88	circRNA	chr17	5319918
MIMAT0000754	hsa-miR-337-3p	hsa_circ_0000739	hsa_circ_001080	circRNA	chr17	5319918
MIMAT0000754	hsa-miR-337-3p	NM_020787	ZNF624	circRNA	chr17	16525167
MIMAT0000754	hsa-miR-337-3p	NM_014964	EPN2	circRNA	chr17	19238423
MIMAT0000754	hsa-miR-337-3p	NM_024857	ATAD5	circRNA	chr17	29041619
MIMAT0000754	hsa-miR-337-3p	NR_015341	LRRC37BP1	circRNA	chr17	29041619
MIMAT0000754	hsa-miR-337-3p	NM_024857	ATAD5	circRNA	chr17	29098493
MIMAT0000754	hsa-miR-337-3p	NR_015341	LRRC37BP1	circRNA	chr17	29098493
MIMAT0000754	hsa-miR-337-3p	NM_015355	SUZ12	circRNA	chr17	29109364
MIMAT0000754	hsa-miR-337-3p	NM_024857	ATAD5	circRNA	chr17	29109364
MIMAT0000754	hsa-miR-337-3p	NR_015341	LRRC37BP1	circRNA	chr17	29109364
MIMAT0000754	hsa-miR-337-3p	NM_015355	SUZ12	circRNA	chr17	29192769
MIMAT0000754	hsa-miR-337-3p	NM_024857	ATAD5	circRNA	chr17	29192769
MIMAT0000754	hsa-miR-337-3p	NR_015341	LRRC37BP1	circRNA	chr17	29192769
MIMAT0000754	hsa-miR-337-3p	hsa_circ_0000756	hsa_circ_001390	circRNA	chr17	29192769
MIMAT0000754	hsa-miR-337-3p	NM_015355	SUZ12	circRNA	chr17	29431769
MIMAT0000754	hsa-miR-337-3p	NM_015355	SUZ12	circRNA	chr17	29547060
MIMAT0000754	hsa-miR-337-3p	NM_015355	SUZ12	circRNA	chr17	29584909
MIMAT0000754	hsa-miR-337-3p	NM_015355	SUZ12	circRNA	chr17	29606765
MIMAT0000754	hsa-miR-337-3p	NM_015355	SUZ12	circRNA	chr17	29896033
MIMAT0000754	hsa-miR-337-3p	NM_001012241	MSL1	circRNA	chr17	38290856
MIMAT0000754	hsa-miR-337-3p	NM_001012241	MSL1	circRNA	chr17	38291729
MIMAT0000754	hsa-miR-337-3p	NM_139276	STAT3	circRNA	chr17	40465347
MIMAT0000754	hsa-miR-337-3p	NM_005533	IFI35	circRNA	chr17	41166371
MIMAT0000754	hsa-miR-337-3p	NM_007300	BRCA1	circRNA	chr17	41246095
MIMAT0000754	hsa-miR-337-3p	NM_031858	NBR1	circRNA	chr17	41363026
MIMAT0000754	hsa-miR-337-3p	NM_030753	WNT3	circRNA	chr17	44196441
MIMAT0000754	hsa-miR-337-3p	hsa_circ_0044187	hsa_circ_0044187	circRNA	chr17	44196441
MIMAT0000754	hsa-miR-337-3p	hsa_circ_0044189	hsa_circ_0044189	circRNA	chr17	44196441
MIMAT0000754	hsa-miR-337-3p	hsa_circ_0044190	hsa_circ_0044190	circRNA	chr17	44196441
MIMAT0000754	hsa-miR-337-3p	hsa_circ_0044191	hsa_circ_0044191	circRNA	chr17	44196441
MIMAT0000754	hsa-miR-337-3p	NM_030753	WNT3	circRNA	chr17	44242213
MIMAT0000754	hsa-miR-337-3p	hsa_circ_0044187	hsa_circ_0044187	circRNA	chr17	44242213
MIMAT0000754	hsa-miR-337-3p	hsa_circ_0044189	hsa_circ_0044189	circRNA	chr17	44242213
MIMAT0000754	hsa-miR-337-3p	hsa_circ_0044190	hsa_circ_0044190	circRNA	chr17	44242213
MIMAT0000754	hsa-miR-337-3p	hsa_circ_0044191	hsa_circ_0044191	circRNA	chr17	44242213
MIMAT0000754	hsa-miR-337-3p	NM_001145365	ZNF652	circRNA	chr17	47367911
MIMAT0000754	hsa-miR-337-3p	NM_001145365	ZNF652	circRNA	chr17	47374256
MIMAT0000754	hsa-miR-337-3p	NM_030802	FAM117A	circRNA	chr17	47788294
MIMAT0000754	hsa-miR-337-3p	NM_001130527	SPAG9	circRNA	chr17	49059925
MIMAT0000754	hsa-miR-337-3p	NM_001130528	SPAG9	circRNA	chr17	49059925
MIMAT0000754	hsa-miR-337-3p	NM_001251971	SPAG9	circRNA	chr17	49059925
MIMAT0000754	hsa-miR-337-3p	hsa_circ_0000785	hsa_circ_001488	circRNA	chr17	49059925
MIMAT0000754	hsa-miR-337-3p	NM_005121	MED13	circRNA	chr17	60062071
MIMAT0000754	hsa-miR-337-3p	NM_006852	TLK2	circRNA	chr17	60651314
MIMAT0000754	hsa-miR-337-3p	NM_025185	TANC2	circRNA	chr17	61500186
MIMAT0000754	hsa-miR-337-3p	NM_020198	CCDC47	circRNA	chr17	61845317
MIMAT0000754	hsa-miR-337-3p	NM_002737	PRKCA	circRNA	chr17	64510268
MIMAT0000754	hsa-miR-337-3p	NM_002737	PRKCA	circRNA	chr17	64605133
MIMAT0000754	hsa-miR-337-3p	NM_015462	NOL11	circRNA	chr17	65717495
MIMAT0000754	hsa-miR-337-3p	NM_004459	BPTF	circRNA	chr17	65907252
MIMAT0000754	hsa-miR-337-3p	NM_182641	BPTF	circRNA	chr17	65907252
MIMAT0000754	hsa-miR-337-3p	NM_182641	BPTF	circRNA	chr17	65959986
MIMAT0000754	hsa-miR-337-3p	NM_020761	RPTOR	circRNA	chr17	78705484
MIMAT0000754	hsa-miR-337-3p	NM_016538	SIRT7	circRNA	chr17	79873001
MIMAT0000754	hsa-miR-337-3p	NM_001012716	C18orf56	circRNA	chr18	649915
MIMAT0000754	hsa-miR-337-3p	NM_006868	RAB31	circRNA	chr18	9740256
MIMAT0000754	hsa-miR-337-3p	NM_006868	RAB31	circRNA	chr18	9765872
MIMAT0000754	hsa-miR-337-3p	NM_001243425	TTC39C	circRNA	chr18	21658146
MIMAT0000754	hsa-miR-337-3p	NM_018439	IMPACT	circRNA	chr18	22030808
MIMAT0000754	hsa-miR-337-3p	hsa_circ_0000840	hsa_circ_000637	circRNA	chr18	22030808
MIMAT0000754	hsa-miR-337-3p	NM_016097	IER3IP1	circRNA	chr18	44681447
MIMAT0000754	hsa-miR-337-3p	NM_021127	PMAIP1	circRNA	chr18	57570659

MIMAT0000754	hsa-miR-337-3p	NM_005483	CHAF1A	circRNA	chr19	4443140
MIMAT0000754	hsa-miR-337-3p	NM_001145160	TPM4	circRNA	chr19	16212566
MIMAT0000754	hsa-miR-337-3p	NM_003290	TPM4	circRNA	chr19	16212566
MIMAT0000754	hsa-miR-337-3p	NM_031218	ZNF93	circRNA	chr19	20026155
MIMAT0000754	hsa-miR-337-3p	NM_001159293	ZNF737	circRNA	chr19	20736553
MIMAT0000754	hsa-miR-337-3p	NM_001076675	ZNF626	circRNA	chr19	20829123
MIMAT0000754	hsa-miR-337-3p	NR_027130	ZNF738	circRNA	chr19	21558108
MIMAT0000754	hsa-miR-337-3p	NM_001076678	ZNF493	circRNA	chr19	21587998
MIMAT0000754	hsa-miR-337-3p	NM_145326	ZNF493	circRNA	chr19	21587998
MIMAT0000754	hsa-miR-337-3p	NM_173531	ZNF100	circRNA	chr19	21927781
MIMAT0000754	hsa-miR-337-3p	NM_003423	ZNF43	circRNA	chr19	22001935
MIMAT0000754	hsa-miR-337-3p	NM_001098626	ZNF98	circRNA	chr19	22586226
MIMAT0000754	hsa-miR-337-3p	NM_020855	ZNF492	circRNA	chr19	22836176
MIMAT0000754	hsa-miR-337-3p	TCONS_l2_000133	TCONS_l2_000133	circRNA	chr19	23332220
MIMAT0000754	hsa-miR-337-3p	NR_045208	LOC100130342	circRNA	chr19	35150383
MIMAT0000754	hsa-miR-337-3p	NM_001003962	CAPNS1	circRNA	chr19	36640762
MIMAT0000754	hsa-miR-337-3p	NM_001749	CAPNS1	circRNA	chr19	36640762
MIMAT0000754	hsa-miR-337-3p	hsa_circ_0000934	hsa_circ_002015	circRNA	chr19	40585626
MIMAT0000754	hsa-miR-337-3p	NM_001199324	ZNF615	circRNA	chr19	52495826
MIMAT0000754	hsa-miR-337-3p	NM_178523	ZNF616	circRNA	chr19	52627190
MIMAT0000754	hsa-miR-337-3p	NM_144684	ZNF480	circRNA	chr19	52825546
MIMAT0000754	hsa-miR-337-3p	NM_032423	ZNF528	circRNA	chr19	52909251
MIMAT0000754	hsa-miR-337-3p	NM_001105553	ZNF83	circRNA	chr19	53122203
MIMAT0000754	hsa-miR-337-3p	NM_001242531	ZNF83	circRNA	chr19	53122203
MIMAT0000754	hsa-miR-337-3p	NR_036599	ZNF28	circRNA	chr19	53311274
MIMAT0000754	hsa-miR-337-3p	NM_199132	ZNF468	circRNA	chr19	53352354
MIMAT0000754	hsa-miR-337-3p	NM_207333	ZNF320	circRNA	chr19	53391394
MIMAT0000754	hsa-miR-337-3p	NM_001008401	ZNF761	circRNA	chr19	53952856
MIMAT0000754	hsa-miR-337-3p	NM_001172773	ZNF548	circRNA	chr19	57911770
MIMAT0000754	hsa-miR-337-3p	NM_138347	ZNF551	circRNA	chr19	58212766
MIMAT0000754	hsa-miR-337-3p	NM_014480	ZNF544	circRNA	chr19	58771045
MIMAT0000754	hsa-miR-337-3p	NM_177559	CSNK2A1	circRNA	chr20	464094
MIMAT0000754	hsa-miR-337-3p	NM_181527	NAA20	circRNA	chr20	20013224
MIMAT0000754	hsa-miR-337-3p	NM_000099	CST3	circRNA	chr20	23614136
MIMAT0000754	hsa-miR-337-3p	NM_016436	PHF20	circRNA	chr20	34537017
MIMAT0000754	hsa-miR-337-3p	NM_002895	RBL1	circRNA	chr20	35649084
MIMAT0000754	hsa-miR-337-3p	NM_181802	UBE2C	circRNA	chr20	44445468
MIMAT0000754	hsa-miR-337-3p	NM_001316	CSE1L	circRNA	chr20	47707476
MIMAT0000754	hsa-miR-337-3p	hsa_circ_0001168	hsa_circ_000906	circRNA	chr20	47707476
MIMAT0000754	hsa-miR-337-3p	NM_018683	RNF114	circRNA	chr20	48569717
MIMAT0000754	hsa-miR-337-3p	NM_017843	BCAS4	circRNA	chr20	49493054
MIMAT0000754	hsa-miR-337-3p	NM_015339	ADNP	circRNA	chr20	49507235
MIMAT0000754	hsa-miR-337-3p	NM_020673	RAB22A	circRNA	chr20	56935245
MIMAT0000754	hsa-miR-337-3p	NM_003185	TAF4	circRNA	chr20	60549897
MIMAT0000754	hsa-miR-337-3p	NM_033081	DIDO1	circRNA	chr20	61541149
MIMAT0000754	hsa-miR-337-3p	NM_080796	DIDO1	circRNA	chr20	61541149
MIMAT0000754	hsa-miR-337-3p	NM_006948	HSPA13	circRNA	chr21	15744100
MIMAT0000754	hsa-miR-337-3p	NM_003489	NRIP1	circRNA	chr21	16365587
MIMAT0000754	hsa-miR-337-3p	TCONS_l2_000171	TCONS_l2_000171	circRNA	chr21	16365587
MIMAT0000754	hsa-miR-337-3p	NM_004540	NCAM2	circRNA	chr21	22652514
MIMAT0000754	hsa-miR-337-3p	NM_000484	APP	circRNA	chr21	27253407
MIMAT0000754	hsa-miR-337-3p	NM_206866	BACH1	circRNA	chr21	30718930
MIMAT0000754	hsa-miR-337-3p	NM_000628	IL10RB	circRNA	chr21	34648901
MIMAT0000754	hsa-miR-337-3p	NM_001136005	GART	circRNA	chr21	34897118
MIMAT0000754	hsa-miR-337-3p	NM_175085	GART	circRNA	chr21	34897118
MIMAT0000754	hsa-miR-337-3p	NM_138927	SON	circRNA	chr21	34924409
MIMAT0000754	hsa-miR-337-3p	NM_006933	SLC5A3	circRNA	chr21	35470995
MIMAT0000754	hsa-miR-337-3p	NM_032476	MRPS6	circRNA	chr21	35478811
MIMAT0000754	hsa-miR-337-3p	NM_032476	MRPS6	circRNA	chr21	35497725
MIMAT0000754	hsa-miR-337-3p	NM_033656	BRWD1	circRNA	chr21	40567166
MIMAT0000754	hsa-miR-337-3p	NM_015241	MICAL3	circRNA	chr22	18482571
MIMAT0000754	hsa-miR-337-3p	NM_014941	MORC2	circRNA	chr22	31363517
MIMAT0000754	hsa-miR-337-3p	NM_019843	EIF4ENIF1	circRNA	chr22	31835494
MIMAT0000754	hsa-miR-337-3p	NR_027780	HMGXB4	circRNA	chr22	35691457
MIMAT0000754	hsa-miR-337-3p	NM_022098	XPNPEP3	circRNA	chr22	41350640

MIMAT0000754	hsa-miR-337-3p	NM_025204	TRABD	circRNA	chr22	50637221
MIMAT0000754	hsa-miR-337-3p	NM_001130923	RABL2B	circRNA	chr22	51208410
MIMAT0000754	hsa-miR-337-3p	hsa_circ_0089761	hsa_circ_0089761	circRNA	chrM	12851
MIMAT0000754	hsa-miR-337-3p	hsa_circ_0089763	hsa_circ_0089763	circRNA	chrM	12851
MIMAT0000754	hsa-miR-337-3p	NM_001039091	PRPS2	circRNA	chrX	12841776
MIMAT0000754	hsa-miR-337-3p	NM_004586	RPS6KA3	circRNA	chrX	20171447
MIMAT0000754	hsa-miR-337-3p	NM_004586	RPS6KA3	circRNA	chrX	20173186
MIMAT0000754	hsa-miR-337-3p	NM_000444	PHEX	circRNA	chrX	22237158
MIMAT0000754	hsa-miR-337-3p	NM_005765	ATP6AP2	circRNA	chrX	40464957
MIMAT0000754	hsa-miR-337-3p	NM_004229	MED14	circRNA	chrX	40534526
MIMAT0000754	hsa-miR-337-3p	NM_014735	PHF16	circRNA	chrX	46845132
MIMAT0000754	hsa-miR-337-3p	NM_018486	HDAC8	circRNA	chrX	71787827
MIMAT0000754	hsa-miR-337-3p	TCONS_00016926	TCONS_00016926	circRNA	chrX	73042378
MIMAT0000754	hsa-miR-337-3p	NM_003022	SH3BGRL	circRNA	chrX	80488944
MIMAT0000754	hsa-miR-337-3p	NM_001113490	AMOT	circRNA	chrX	1.12E+08
MIMAT0000754	hsa-miR-337-3p	TCONS_00017083	TCONS_00017083	circRNA	chrX	1.15E+08
MIMAT0000754	hsa-miR-337-3p	NM_001560	IL13RA1	circRNA	chrX	1.18E+08
MIMAT0000754	hsa-miR-337-3p	NM_013995	LAMP2	circRNA	chrX	1.2E+08
MIMAT0000754	hsa-miR-337-3p	NM_001167	XIAP	circRNA	chrX	1.23E+08
MIMAT0000754	hsa-miR-337-3p	NM_021183	RAP2C	circRNA	chrX	1.31E+08
MIMAT0000754	hsa-miR-337-3p	NM_173470	MMGT1	circRNA	chrX	1.35E+08
MIMAT0000754	hsa-miR-337-3p	NM_001164803	RBMX	circRNA	chrX	1.36E+08
MIMAT0000754	hsa-miR-337-3p	NM_000252	MTM1	circRNA	chrX	1.5E+08
MIMAT0000754	hsa-miR-337-3p	NM_024332	BRCC3	circRNA	chrX	1.54E+08
MIMAT0000754	hsa-miR-337-3p	NM_003372	VBP1	circRNA	chrX	1.54E+08

miR-337-3p was predicted to target Bcl2

miRNAid	miRNAname	geneID	geneName	geneType	chromosome	narrowStart
MIMAT0000062	hsa-let-7a-5p	ENSG00000171791	BCL2	protein_coding	chr18	60792463
MIMAT0000063	hsa-let-7b-5p	ENSG00000171791	BCL2	protein_coding	chr18	60792463
MIMAT0000064	hsa-let-7c-5p	ENSG00000171791	BCL2	protein_coding	chr18	60792463
MIMAT0000065	hsa-let-7d-5p	ENSG00000171791	BCL2	protein_coding	chr18	60792463
MIMAT0000066	hsa-let-7e-5p	ENSG00000171791	BCL2	protein_coding	chr18	60792463
MIMAT0000067	hsa-let-7f-5p	ENSG00000171791	BCL2	protein_coding	chr18	60792463
MIMAT0000068	hsa-miR-15a-5p	ENSG00000171791	BCL2	protein_coding	chr18	60793253
MIMAT0000068	hsa-miR-15a-5p	ENSG00000171791	BCL2	protein_coding	chr18	60793323
MIMAT0000068	hsa-miR-15a-5p	ENSG00000171791	BCL2	protein_coding	chr18	60794055
MIMAT0000068	hsa-miR-15a-5p	ENSG00000171791	BCL2	protein_coding	chr18	60795399
MIMAT0000069	hsa-miR-16-5p	ENSG00000171791	BCL2	protein_coding	chr18	60793253
MIMAT0000069	hsa-miR-16-5p	ENSG00000171791	BCL2	protein_coding	chr18	60793323
MIMAT0000069	hsa-miR-16-5p	ENSG00000171791	BCL2	protein_coding	chr18	60794055
MIMAT0000069	hsa-miR-16-5p	ENSG00000171791	BCL2	protein_coding	chr18	60795399
MIMAT0000070	hsa-miR-17-5p	ENSG00000171791	BCL2	protein_coding	chr18	60790674
MIMAT0000070	hsa-miR-17-5p	ENSG00000171791	BCL2	protein_coding	chr18	60792739
MIMAT0000075	hsa-miR-20a-5p	ENSG00000171791	BCL2	protein_coding	chr18	60790674
MIMAT0000075	hsa-miR-20a-5p	ENSG00000171791	BCL2	protein_coding	chr18	60792739
MIMAT0000076	hsa-miR-21-5p	ENSG00000171791	BCL2	protein_coding	chr18	60795133
MIMAT0000076	hsa-miR-21-5p	ENSG00000171791	BCL2	protein_coding	chr18	60985831
MIMAT0000077	hsa-miR-22-3p	ENSG00000171791	BCL2	protein_coding	chr18	60791624
MIMAT0000078	hsa-miR-23a-3p	ENSG00000171791	BCL2	protein_coding	chr18	60791441
MIMAT0000078	hsa-miR-23a-3p	ENSG00000171791	BCL2	protein_coding	chr18	60792191
MIMAT0000078	hsa-miR-23a-3p	ENSG00000171791	BCL2	protein_coding	chr18	60792428
MIMAT0000081	hsa-miR-25-3p	ENSG00000171791	BCL2	protein_coding	chr18	60791607
MIMAT0000084	hsa-miR-27a-3p	ENSG00000171791	BCL2	protein_coding	chr18	60794487
MIMAT0000084	hsa-miR-27a-3p	ENSG00000171791	BCL2	protein_coding	chr18	60795343
MIMAT0000087	hsa-miR-30a-5p	ENSG00000171791	BCL2	protein_coding	chr18	60793366
MIMAT0000090	hsa-miR-32-5p	ENSG00000171791	BCL2	protein_coding	chr18	60791607
MIMAT0000092	hsa-miR-92a-3p	ENSG00000171791	BCL2	protein_coding	chr18	60791607
MIMAT0000093	hsa-miR-93-5p	ENSG00000171791	BCL2	protein_coding	chr18	60790674
MIMAT0000093	hsa-miR-93-5p	ENSG00000171791	BCL2	protein_coding	chr18	60792739
MIMAT0000095	hsa-miR-96-5p	ENSG00000171791	BCL2	protein_coding	chr18	60793313
MIMAT0000095	hsa-miR-96-5p	ENSG00000171791	BCL2	protein_coding	chr18	60795542
MIMAT0000095	hsa-miR-96-5p	ENSG00000171791	BCL2	protein_coding	chr18	60795648

MIMAT0000096	hsa-miR-98-5p	ENSG00000171791	BCL2	protein_coding	chr18	60792463
MIMAT0000099	hsa-miR-101-3p	ENSG00000171791	BCL2	protein_coding	chr18	60791290
MIMAT0000101	hsa-miR-103a-3p	ENSG00000171791	BCL2	protein_coding	chr18	60793324
MIMAT0000103	hsa-miR-106a-5p	ENSG00000171791	BCL2	protein_coding	chr18	60790674
MIMAT0000103	hsa-miR-106a-5p	ENSG00000171791	BCL2	protein_coding	chr18	60792739
MIMAT0000104	hsa-miR-107	ENSG00000171791	BCL2	protein_coding	chr18	60793324
MIMAT0000222	hsa-miR-192-5p	ENSG00000171791	BCL2	protein_coding	chr18	60793137
MIMAT0000226	hsa-miR-196a-5p	ENSG00000171791	BCL2	protein_coding	chr18	60793888
MIMAT0000227	hsa-miR-197-3p	ENSG00000171791	BCL2	protein_coding	chr18	60794577
MIMAT0000231	hsa-miR-199a-5p	ENSG00000171791	BCL2	protein_coding	chr18	60791185
MIMAT0000231	hsa-miR-199a-5p	ENSG00000171791	BCL2	protein_coding	chr18	60791327
MIMAT0000231	hsa-miR-199a-5p	ENSG00000171791	BCL2	protein_coding	chr18	60793865
MIMAT0000241	hsa-miR-208a-3p	ENSG00000171791	BCL2	protein_coding	chr18	60791401
MIMAT0000242	hsa-miR-129-5p	ENSG00000171791	BCL2	protein_coding	chr18	60794328
MIMAT0000244	hsa-miR-30c-5p	ENSG00000171791	BCL2	protein_coding	chr18	60793366
MIMAT0000245	hsa-miR-30d-5p	ENSG00000171791	BCL2	protein_coding	chr18	60793366
MIMAT0000250	hsa-miR-139-5p	ENSG00000171791	BCL2	protein_coding	chr18	60791122
MIMAT0000250	hsa-miR-139-5p	ENSG00000171791	BCL2	protein_coding	chr18	60794357
MIMAT0000250	hsa-miR-139-5p	ENSG00000171791	BCL2	protein_coding	chr18	60794527
MIMAT0000250	hsa-miR-139-5p	ENSG00000171791	BCL2	protein_coding	chr18	60794626
MIMAT0000251	hsa-miR-147a	ENSG00000171791	BCL2	protein_coding	chr18	60795702
MIMAT0000251	hsa-miR-147a	ENSG00000171791	BCL2	protein_coding	chr18	60795706
MIMAT0000251	hsa-miR-147a	ENSG00000171791	BCL2	protein_coding	chr18	60795721
MIMAT0000251	hsa-miR-147a	ENSG00000171791	BCL2	protein_coding	chr18	60795736
MIMAT0000252	hsa-miR-7-5p	ENSG00000171791	BCL2	protein_coding	chr18	60794189
MIMAT0000255	hsa-miR-34a-5p	ENSG00000171791	BCL2	protein_coding	chr18	60791270
MIMAT0000255	hsa-miR-34a-5p	ENSG00000171791	BCL2	protein_coding	chr18	60795650
MIMAT0000256	hsa-miR-181a-5p	ENSG00000171791	BCL2	protein_coding	chr18	60792956
MIMAT0000256	hsa-miR-181a-5p	ENSG00000171791	BCL2	protein_coding	chr18	60794101
MIMAT0000257	hsa-miR-181b-5p	ENSG00000171791	BCL2	protein_coding	chr18	60792956
MIMAT0000257	hsa-miR-181b-5p	ENSG00000171791	BCL2	protein_coding	chr18	60794101
MIMAT0000258	hsa-miR-181c-5p	ENSG00000171791	BCL2	protein_coding	chr18	60792956
MIMAT0000258	hsa-miR-181c-5p	ENSG00000171791	BCL2	protein_coding	chr18	60794101
MIMAT0000259	hsa-miR-182-5p	ENSG00000171791	BCL2	protein_coding	chr18	60793313
MIMAT0000259	hsa-miR-182-5p	ENSG00000171791	BCL2	protein_coding	chr18	60795542
MIMAT0000259	hsa-miR-182-5p	ENSG00000171791	BCL2	protein_coding	chr18	60795648
MIMAT0000261	hsa-miR-183-5p	ENSG00000171791	BCL2	protein_coding	chr18	60790642
MIMAT0000263	hsa-miR-199b-5p	ENSG00000171791	BCL2	protein_coding	chr18	60791185
MIMAT0000263	hsa-miR-199b-5p	ENSG00000171791	BCL2	protein_coding	chr18	60791327
MIMAT0000263	hsa-miR-199b-5p	ENSG00000171791	BCL2	protein_coding	chr18	60793865
MIMAT0000265	hsa-miR-204-5p	ENSG00000171791	BCL2	protein_coding	chr18	60795643
MIMAT0000266	hsa-miR-205-5p	ENSG00000171791	BCL2	protein_coding	chr18	60794574
MIMAT0000268	hsa-miR-211-5p	ENSG00000171791	BCL2	protein_coding	chr18	60795643
MIMAT0000271	hsa-miR-214-3p	ENSG00000171791	BCL2	protein_coding	chr18	60795398
MIMAT0000272	hsa-miR-215-5p	ENSG00000171791	BCL2	protein_coding	chr18	60793137
MIMAT0000273	hsa-miR-216a-5p	ENSG00000171791	BCL2	protein_coding	chr18	60793952
MIMAT0000273	hsa-miR-216a-5p	ENSG00000171791	BCL2	protein_coding	chr18	60795197
MIMAT0000274	hsa-miR-217	ENSG00000171791	BCL2	protein_coding	chr18	60791551
MIMAT0000275	hsa-miR-218-5p	ENSG00000171791	BCL2	protein_coding	chr18	60791202
MIMAT0000275	hsa-miR-218-5p	ENSG00000171791	BCL2	protein_coding	chr18	60794523
MIMAT0000276	hsa-miR-219a-5p	ENSG00000171791	BCL2	protein_coding	chr18	60791348
MIMAT0000278	hsa-miR-221-3p	ENSG00000171791	BCL2	protein_coding	chr18	60792386
MIMAT0000278	hsa-miR-221-3p	ENSG00000171791	BCL2	protein_coding	chr18	60794526
MIMAT0000279	hsa-miR-222-3p	ENSG00000171791	BCL2	protein_coding	chr18	60792386
MIMAT0000279	hsa-miR-222-3p	ENSG00000171791	BCL2	protein_coding	chr18	60794526
MIMAT0000281	hsa-miR-224-5p	ENSG00000171791	BCL2	protein_coding	chr18	60794461
MIMAT0000318	hsa-miR-200b-3p	ENSG00000171791	BCL2	protein_coding	chr18	60793032
MIMAT0000318	hsa-miR-200b-3p	ENSG00000171791	BCL2	protein_coding	chr18	60793165
MIMAT0000318	hsa-miR-200b-3p	ENSG00000171791	BCL2	protein_coding	chr18	60795143
MIMAT0000414	hsa-let-7g-5p	ENSG00000171791	BCL2	protein_coding	chr18	60792463
MIMAT0000415	hsa-let-7i-5p	ENSG00000171791	BCL2	protein_coding	chr18	60792463
MIMAT0000417	hsa-miR-15b-5p	ENSG00000171791	BCL2	protein_coding	chr18	60793253
MIMAT0000417	hsa-miR-15b-5p	ENSG00000171791	BCL2	protein_coding	chr18	60793323
MIMAT0000417	hsa-miR-15b-5p	ENSG00000171791	BCL2	protein_coding	chr18	60794055
MIMAT0000417	hsa-miR-15b-5p	ENSG00000171791	BCL2	protein_coding	chr18	60795399

MIMAT0000418	hsa-miR-23b-3p	ENSG00000171791	BCL2	protein_coding	chr18	60791441
MIMAT0000418	hsa-miR-23b-3p	ENSG00000171791	BCL2	protein_coding	chr18	60792191
MIMAT0000418	hsa-miR-23b-3p	ENSG00000171791	BCL2	protein_coding	chr18	60792428
MIMAT0000419	hsa-miR-27b-3p	ENSG00000171791	BCL2	protein_coding	chr18	60794487
MIMAT0000419	hsa-miR-27b-3p	ENSG00000171791	BCL2	protein_coding	chr18	60795343
MIMAT0000420	hsa-miR-30b-5p	ENSG00000171791	BCL2	protein_coding	chr18	60793366
MIMAT0000423	hsa-miR-125b-5p	ENSG00000171791	BCL2	protein_coding	chr18	60792304
MIMAT0000423	hsa-miR-125b-5p	ENSG00000171791	BCL2	protein_coding	chr18	60793433
MIMAT0000425	hsa-miR-130a-3p	ENSG00000171791	BCL2	protein_coding	chr18	60792741
MIMAT0000425	hsa-miR-130a-3p	ENSG00000171791	BCL2	protein_coding	chr18	60793021
MIMAT0000431	hsa-miR-140-5p	ENSG00000171791	BCL2	protein_coding	chr18	60795551
MIMAT0000432	hsa-miR-141-3p	ENSG00000171791	BCL2	protein_coding	chr18	60790864
MIMAT0000435	hsa-miR-143-3p	ENSG00000171791	BCL2	protein_coding	chr18	60791332
MIMAT0000435	hsa-miR-143-3p	ENSG00000171791	BCL2	protein_coding	chr18	60794034
MIMAT0000436	hsa-miR-144-3p	ENSG00000171791	BCL2	protein_coding	chr18	60791290
MIMAT0000437	hsa-miR-145-5p	ENSG00000171791	BCL2	protein_coding	chr18	60791326
MIMAT0000439	hsa-miR-153-3p	ENSG00000171791	BCL2	protein_coding	chr18	60791336
MIMAT0000439	hsa-miR-153-3p	ENSG00000171791	BCL2	protein_coding	chr18	60792743
MIMAT0000439	hsa-miR-153-3p	ENSG00000171791	BCL2	protein_coding	chr18	60795794
MIMAT0000439	hsa-miR-153-3p	ENSG00000171791	BCL2	protein_coding	chr18	60795827
MIMAT0000440	hsa-miR-191-5p	ENSG00000171791	BCL2	protein_coding	chr18	60791619
MIMAT0000441	hsa-miR-9-5p	ENSG00000171791	BCL2	protein_coding	chr18	60795646
MIMAT0000442	hsa-miR-9-3p	ENSG00000171791	BCL2	protein_coding	chr18	60793250
MIMAT0000442	hsa-miR-9-3p	ENSG00000171791	BCL2	protein_coding	chr18	60794052
MIMAT0000442	hsa-miR-9-3p	ENSG00000171791	BCL2	protein_coding	chr18	60794469
MIMAT0000443	hsa-miR-125a-5p	ENSG00000171791	BCL2	protein_coding	chr18	60792304
MIMAT0000443	hsa-miR-125a-5p	ENSG00000171791	BCL2	protein_coding	chr18	60793433
MIMAT0000445	hsa-miR-126-3p	ENSG00000171791	BCL2	protein_coding	chr18	60791667
MIMAT0000447	hsa-miR-134-5p	ENSG00000171791	BCL2	protein_coding	chr18	60790650
MIMAT0000447	hsa-miR-134-5p	ENSG00000171791	BCL2	protein_coding	chr18	60792532
MIMAT0000448	hsa-miR-136-5p	ENSG00000171791	BCL2	protein_coding	chr18	60791305
MIMAT0000448	hsa-miR-136-5p	ENSG00000171791	BCL2	protein_coding	chr18	60793297
MIMAT0000450	hsa-miR-149-5p	ENSG00000171791	BCL2	protein_coding	chr18	60793455
MIMAT0000451	hsa-miR-150-5p	ENSG00000171791	BCL2	protein_coding	chr18	60794659
MIMAT0000451	hsa-miR-150-5p	ENSG00000171791	BCL2	protein_coding	chr18	60795242
MIMAT0000455	hsa-miR-185-5p	ENSG00000171791	BCL2	protein_coding	chr18	60792479
MIMAT0000456	hsa-miR-186-5p	ENSG00000171791	BCL2	protein_coding	chr18	60790974
MIMAT0000456	hsa-miR-186-5p	ENSG00000171791	BCL2	protein_coding	chr18	60790990
MIMAT0000456	hsa-miR-186-5p	ENSG00000171791	BCL2	protein_coding	chr18	60795359
MIMAT0000461	hsa-miR-195-5p	ENSG00000171791	BCL2	protein_coding	chr18	60793253
MIMAT0000461	hsa-miR-195-5p	ENSG00000171791	BCL2	protein_coding	chr18	60793323
MIMAT0000461	hsa-miR-195-5p	ENSG00000171791	BCL2	protein_coding	chr18	60794055
MIMAT0000461	hsa-miR-195-5p	ENSG00000171791	BCL2	protein_coding	chr18	60795399
MIMAT0000510	hsa-miR-320a	ENSG00000171791	BCL2	protein_coding	chr18	60790847
MIMAT0000510	hsa-miR-320a	ENSG00000171791	BCL2	protein_coding	chr18	60790978
MIMAT0000510	hsa-miR-320a	ENSG00000171791	BCL2	protein_coding	chr18	60792280
MIMAT0000510	hsa-miR-320a	ENSG00000171791	BCL2	protein_coding	chr18	60793850
MIMAT0000617	hsa-miR-200c-3p	ENSG00000171791	BCL2	protein_coding	chr18	60793032
MIMAT0000617	hsa-miR-200c-3p	ENSG00000171791	BCL2	protein_coding	chr18	60793165
MIMAT0000617	hsa-miR-200c-3p	ENSG00000171791	BCL2	protein_coding	chr18	60795143
MIMAT0000646	hsa-miR-155-5p	ENSG00000171791	BCL2	protein_coding	chr18	60794133
MIMAT0000646	hsa-miR-155-5p	ENSG00000171791	BCL2	protein_coding	chr18	60794141
MIMAT0000646	hsa-miR-155-5p	ENSG00000171791	BCL2	protein_coding	chr18	60794277
MIMAT0000680	hsa-miR-106b-5p	ENSG00000171791	BCL2	protein_coding	chr18	60790674
MIMAT0000680	hsa-miR-106b-5p	ENSG00000171791	BCL2	protein_coding	chr18	60792739
MIMAT0000682	hsa-miR-200a-3p	ENSG00000171791	BCL2	protein_coding	chr18	60790864
MIMAT0000684	hsa-miR-302a-3p	ENSG00000171791	BCL2	protein_coding	chr18	60790675
MIMAT0000684	hsa-miR-302a-3p	ENSG00000171791	BCL2	protein_coding	chr18	60792740
MIMAT0000684	hsa-miR-302a-3p	ENSG00000171791	BCL2	protein_coding	chr18	60793020
MIMAT0000686	hsa-miR-34c-5p	ENSG00000171791	BCL2	protein_coding	chr18	60791270
MIMAT0000686	hsa-miR-34c-5p	ENSG00000171791	BCL2	protein_coding	chr18	60795650
MIMAT0000688	hsa-miR-301a-3p	ENSG00000171791	BCL2	protein_coding	chr18	60792741
MIMAT0000688	hsa-miR-301a-3p	ENSG00000171791	BCL2	protein_coding	chr18	60793021
MIMAT0000690	hsa-miR-296-5p	ENSG00000171791	BCL2	protein_coding	chr18	60791065
MIMAT0000690	hsa-miR-296-5p	ENSG00000171791	BCL2	protein_coding	chr18	60794600

MIMAT0000691	hsa-miR-130b-3p	ENSG00000171791	BCL2	protein_coding	chr18	60792741
MIMAT0000691	hsa-miR-130b-3p	ENSG00000171791	BCL2	protein_coding	chr18	60793021
MIMAT0000692	hsa-miR-30e-5p	ENSG00000171791	BCL2	protein_coding	chr18	60793366
MIMAT0000707	hsa-miR-363-3p	ENSG00000171791	BCL2	protein_coding	chr18	60791607
MIMAT0000710	hsa-miR-365a-3p	ENSG00000171791	BCL2	protein_coding	chr18	60794142
MIMAT0000710	hsa-miR-365a-3p	ENSG00000171791	BCL2	protein_coding	chr18	60794278
MIMAT0000715	hsa-miR-302b-3p	ENSG00000171791	BCL2	protein_coding	chr18	60790675
MIMAT0000715	hsa-miR-302b-3p	ENSG00000171791	BCL2	protein_coding	chr18	60792740
MIMAT0000715	hsa-miR-302b-3p	ENSG00000171791	BCL2	protein_coding	chr18	60793020
MIMAT0000717	hsa-miR-302c-3p	ENSG00000171791	BCL2	protein_coding	chr18	60790675
MIMAT0000717	hsa-miR-302c-3p	ENSG00000171791	BCL2	protein_coding	chr18	60792740
MIMAT0000717	hsa-miR-302c-3p	ENSG00000171791	BCL2	protein_coding	chr18	60793020
MIMAT0000718	hsa-miR-302d-3p	ENSG00000171791	BCL2	protein_coding	chr18	60790675
MIMAT0000718	hsa-miR-302d-3p	ENSG00000171791	BCL2	protein_coding	chr18	60792740
MIMAT0000718	hsa-miR-302d-3p	ENSG00000171791	BCL2	protein_coding	chr18	60793020
MIMAT0000719	hsa-miR-367-3p	ENSG00000171791	BCL2	protein_coding	chr18	60791607
MIMAT0000721	hsa-miR-369-3p	ENSG00000171791	BCL2	protein_coding	chr18	60790955
MIMAT0000721	hsa-miR-369-3p	ENSG00000171791	BCL2	protein_coding	chr18	60791482
MIMAT0000721	hsa-miR-369-3p	ENSG00000171791	BCL2	protein_coding	chr18	60793382
MIMAT0000723	hsa-miR-371a-3p	ENSG00000171791	BCL2	protein_coding	chr18	60790676
MIMAT0000724	hsa-miR-372-3p	ENSG00000171791	BCL2	protein_coding	chr18	60790675
MIMAT0000724	hsa-miR-372-3p	ENSG00000171791	BCL2	protein_coding	chr18	60792740
MIMAT0000724	hsa-miR-372-3p	ENSG00000171791	BCL2	protein_coding	chr18	60793020
MIMAT0000726	hsa-miR-373-3p	ENSG00000171791	BCL2	protein_coding	chr18	60790675
MIMAT0000726	hsa-miR-373-3p	ENSG00000171791	BCL2	protein_coding	chr18	60792740
MIMAT0000726	hsa-miR-373-3p	ENSG00000171791	BCL2	protein_coding	chr18	60793020
MIMAT0000727	hsa-miR-374a-5p	ENSG00000171791	BCL2	protein_coding	chr18	60790954
MIMAT0000727	hsa-miR-374a-5p	ENSG00000171791	BCL2	protein_coding	chr18	60792350
MIMAT0000727	hsa-miR-374a-5p	ENSG00000171791	BCL2	protein_coding	chr18	60794131
MIMAT0000727	hsa-miR-374a-5p	ENSG00000171791	BCL2	protein_coding	chr18	60985835
MIMAT0000735	hsa-miR-380-3p	ENSG00000171791	BCL2	protein_coding	chr18	60793364
MIMAT0000735	hsa-miR-380-3p	ENSG00000171791	BCL2	protein_coding	chr18	60793705
MIMAT0000735	hsa-miR-380-3p	ENSG00000171791	BCL2	protein_coding	chr18	60794382
MIMAT0000735	hsa-miR-380-3p	ENSG00000171791	BCL2	protein_coding	chr18	60795622
MIMAT0000736	hsa-miR-381-3p	ENSG00000171791	BCL2	protein_coding	chr18	60790857
MIMAT0000736	hsa-miR-381-3p	ENSG00000171791	BCL2	protein_coding	chr18	60794009
MIMAT0000738	hsa-miR-383-5p	ENSG00000171791	BCL2	protein_coding	chr18	60792019
MIMAT0000738	hsa-miR-383-5p	ENSG00000171791	BCL2	protein_coding	chr18	60795426
MIMAT0000751	hsa-miR-330-3p	ENSG00000171791	BCL2	protein_coding	chr18	60793849
MIMAT0000753	hsa-miR-342-3p	ENSG00000171791	BCL2	protein_coding	chr18	60790584
MIMAT0000753	hsa-miR-342-3p	ENSG00000171791	BCL2	protein_coding	chr18	60793980
MIMAT0000753	hsa-miR-342-3p	ENSG00000171791	BCL2	protein_coding	chr18	60794486
MIMAT0000754	hsa-miR-337-3p	ENSG00000171791	BCL2	protein_coding	chr18	60795150
MIMAT0000755	hsa-miR-323a-3p	ENSG00000171791	BCL2	protein_coding	chr18	60792109
MIMAT0000756	hsa-miR-326	ENSG00000171791	BCL2	protein_coding	chr18	60791650
MIMAT0000760	hsa-miR-331-3p	ENSG00000171791	BCL2	protein_coding	chr18	60792572
MIMAT0000761	hsa-miR-324-5p	ENSG00000171791	BCL2	protein_coding	chr18	60791323
MIMAT0000762	hsa-miR-324-3p	ENSG00000171791	BCL2	protein_coding	chr18	60792569
MIMAT0000763	hsa-miR-338-3p	ENSG00000171791	BCL2	protein_coding	chr18	60795397
MIMAT0000765	hsa-miR-335-5p	ENSG00000171791	BCL2	protein_coding	chr18	60793053
MIMAT0000765	hsa-miR-335-5p	ENSG00000171791	BCL2	protein_coding	chr18	60793941
MIMAT0000772	hsa-miR-345-5p	ENSG00000171791	BCL2	protein_coding	chr18	60792038
MIMAT0000772	hsa-miR-345-5p	ENSG00000171791	BCL2	protein_coding	chr18	60793293
MIMAT0000773	hsa-miR-346	ENSG00000171791	BCL2	protein_coding	chr18	60795447
MIMAT0001075	hsa-miR-384	ENSG00000171791	BCL2	protein_coding	chr18	60794204
MIMAT0001075	hsa-miR-384	ENSG00000171791	BCL2	protein_coding	chr18	60795149
MIMAT0001080	hsa-miR-196b-5p	ENSG00000171791	BCL2	protein_coding	chr18	60793888
MIMAT0001341	hsa-miR-424-5p	ENSG00000171791	BCL2	protein_coding	chr18	60793253
MIMAT0001341	hsa-miR-424-5p	ENSG00000171791	BCL2	protein_coding	chr18	60793323
MIMAT0001341	hsa-miR-424-5p	ENSG00000171791	BCL2	protein_coding	chr18	60794055
MIMAT0001341	hsa-miR-424-5p	ENSG00000171791	BCL2	protein_coding	chr18	60795399
MIMAT0001413	hsa-miR-20b-5p	ENSG00000171791	BCL2	protein_coding	chr18	60790674
MIMAT0001413	hsa-miR-20b-5p	ENSG00000171791	BCL2	protein_coding	chr18	60792739
MIMAT0001532	hsa-miR-448	ENSG00000171791	BCL2	protein_coding	chr18	60791336
MIMAT0001532	hsa-miR-448	ENSG00000171791	BCL2	protein_coding	chr18	60792743

MIMAT0001532	hsa-miR-448	ENSG00000171791	BCL2	protein_coding	chr18	60795794
MIMAT0001532	hsa-miR-448	ENSG00000171791	BCL2	protein_coding	chr18	60795827
MIMAT0001536	hsa-miR-429	ENSG00000171791	BCL2	protein_coding	chr18	60793032
MIMAT0001536	hsa-miR-429	ENSG00000171791	BCL2	protein_coding	chr18	60793165
MIMAT0001536	hsa-miR-429	ENSG00000171791	BCL2	protein_coding	chr18	60795143
MIMAT0001541	hsa-miR-449a	ENSG00000171791	BCL2	protein_coding	chr18	60791270
MIMAT0001541	hsa-miR-449a	ENSG00000171791	BCL2	protein_coding	chr18	60795650
MIMAT0001625	hsa-miR-431-5p	ENSG00000171791	BCL2	protein_coding	chr18	60792494
MIMAT0001627	hsa-miR-433-3p	ENSG00000171791	BCL2	protein_coding	chr18	60791344
MIMAT0001635	hsa-miR-452-5p	ENSG00000171791	BCL2	protein_coding	chr18	60793072
MIMAT0001635	hsa-miR-452-5p	ENSG00000171791	BCL2	protein_coding	chr18	60795685
MIMAT0001639	hsa-miR-409-3p	ENSG00000171791	BCL2	protein_coding	chr18	60791684
MIMAT0001639	hsa-miR-409-3p	ENSG00000171791	BCL2	protein_coding	chr18	60792485
MIMAT0001639	hsa-miR-409-3p	ENSG00000171791	BCL2	protein_coding	chr18	60794164
MIMAT0001639	hsa-miR-409-3p	ENSG00000171791	BCL2	protein_coding	chr18	60794372
MIMAT0002171	hsa-miR-410-3p	ENSG00000171791	BCL2	protein_coding	chr18	60792432
MIMAT0002171	hsa-miR-410-3p	ENSG00000171791	BCL2	protein_coding	chr18	60794130
MIMAT0002175	hsa-miR-485-5p	ENSG00000171791	BCL2	protein_coding	chr18	60793056
MIMAT0002176	hsa-miR-485-3p	ENSG00000171791	BCL2	protein_coding	chr18	60793461
MIMAT0002805	hsa-miR-489-3p	ENSG00000171791	BCL2	protein_coding	chr18	60793869
MIMAT0002810	hsa-miR-202-5p	ENSG00000171791	BCL2	protein_coding	chr18	60795150
MIMAT0002813	hsa-miR-493-5p	ENSG00000171791	BCL2	protein_coding	chr18	60790873
MIMAT0002813	hsa-miR-493-5p	ENSG00000171791	BCL2	protein_coding	chr18	60791477
MIMAT0002813	hsa-miR-493-5p	ENSG00000171791	BCL2	protein_coding	chr18	60792106
MIMAT0002813	hsa-miR-493-5p	ENSG00000171791	BCL2	protein_coding	chr18	60793037
MIMAT0002813	hsa-miR-493-5p	ENSG00000171791	BCL2	protein_coding	chr18	60794290
MIMAT0002814	hsa-miR-432-5p	ENSG00000171791	BCL2	protein_coding	chr18	60791028
MIMAT0002814	hsa-miR-432-5p	ENSG00000171791	BCL2	protein_coding	chr18	60793258
MIMAT0002816	hsa-miR-494-3p	ENSG00000171791	BCL2	protein_coding	chr18	60791496
MIMAT0002816	hsa-miR-494-3p	ENSG00000171791	BCL2	protein_coding	chr18	60793845
MIMAT0002817	hsa-miR-495-3p	ENSG00000171791	BCL2	protein_coding	chr18	60790670
MIMAT0002817	hsa-miR-495-3p	ENSG00000171791	BCL2	protein_coding	chr18	60790843
MIMAT0002817	hsa-miR-495-3p	ENSG00000171791	BCL2	protein_coding	chr18	60790894
MIMAT0002817	hsa-miR-495-3p	ENSG00000171791	BCL2	protein_coding	chr18	60790919
MIMAT0002817	hsa-miR-495-3p	ENSG00000171791	BCL2	protein_coding	chr18	60790961
MIMAT0002817	hsa-miR-495-3p	ENSG00000171791	BCL2	protein_coding	chr18	60792420
MIMAT0002817	hsa-miR-495-3p	ENSG00000171791	BCL2	protein_coding	chr18	60793193
MIMAT0002817	hsa-miR-495-3p	ENSG00000171791	BCL2	protein_coding	chr18	60793847
MIMAT0002817	hsa-miR-495-3p	ENSG00000171791	BCL2	protein_coding	chr18	60794020
MIMAT0002820	hsa-miR-497-5p	ENSG00000171791	BCL2	protein_coding	chr18	60793253
MIMAT0002820	hsa-miR-497-5p	ENSG00000171791	BCL2	protein_coding	chr18	60793323
MIMAT0002820	hsa-miR-497-5p	ENSG00000171791	BCL2	protein_coding	chr18	60794055
MIMAT0002820	hsa-miR-497-5p	ENSG00000171791	BCL2	protein_coding	chr18	60795399
MIMAT0002821	hsa-miR-181d-5p	ENSG00000171791	BCL2	protein_coding	chr18	60792956
MIMAT0002821	hsa-miR-181d-5p	ENSG00000171791	BCL2	protein_coding	chr18	60794101
MIMAT0002824	hsa-miR-498	ENSG00000171791	BCL2	protein_coding	chr18	60794169
MIMAT0002825	hsa-miR-520e	ENSG00000171791	BCL2	protein_coding	chr18	60790675
MIMAT0002825	hsa-miR-520e	ENSG00000171791	BCL2	protein_coding	chr18	60792740
MIMAT0002825	hsa-miR-520e	ENSG00000171791	BCL2	protein_coding	chr18	60793020
MIMAT0002826	hsa-miR-515-5p	ENSG00000171791	BCL2	protein_coding	chr18	60791427
MIMAT0002826	hsa-miR-515-5p	ENSG00000171791	BCL2	protein_coding	chr18	60794455
MIMAT0002828	hsa-miR-519e-5p	ENSG00000171791	BCL2	protein_coding	chr18	60791427
MIMAT0002828	hsa-miR-519e-5p	ENSG00000171791	BCL2	protein_coding	chr18	60794455
MIMAT0002832	hsa-miR-519c-3p	ENSG00000171791	BCL2	protein_coding	chr18	60790675
MIMAT0002832	hsa-miR-519c-3p	ENSG00000171791	BCL2	protein_coding	chr18	60792740
MIMAT0002832	hsa-miR-519c-3p	ENSG00000171791	BCL2	protein_coding	chr18	60793020
MIMAT0002833	hsa-miR-520a-5p	ENSG00000171791	BCL2	protein_coding	chr18	60791275
MIMAT0002834	hsa-miR-520a-3p	ENSG00000171791	BCL2	protein_coding	chr18	60790675
MIMAT0002834	hsa-miR-520a-3p	ENSG00000171791	BCL2	protein_coding	chr18	60792740
MIMAT0002834	hsa-miR-520a-3p	ENSG00000171791	BCL2	protein_coding	chr18	60793020
MIMAT0002835	hsa-miR-526b-5p	ENSG00000171791	BCL2	protein_coding	chr18	60790836
MIMAT0002835	hsa-miR-526b-5p	ENSG00000171791	BCL2	protein_coding	chr18	60791155
MIMAT0002836	hsa-miR-526b-3p	ENSG00000171791	BCL2	protein_coding	chr18	60790674
MIMAT0002836	hsa-miR-526b-3p	ENSG00000171791	BCL2	protein_coding	chr18	60792739
MIMAT0002837	hsa-miR-519b-3p	ENSG00000171791	BCL2	protein_coding	chr18	60790675

MIMAT0002837	hsa-miR-519b-3p	ENSG00000171791	BCL2	protein_coding	chr18	60792740
MIMAT0002837	hsa-miR-519b-3p	ENSG00000171791	BCL2	protein_coding	chr18	60793020
MIMAT0002838	hsa-miR-525-5p	ENSG00000171791	BCL2	protein_coding	chr18	60791275
MIMAT0002842	hsa-miR-518f-3p	ENSG00000171791	BCL2	protein_coding	chr18	60795484
MIMAT0002843	hsa-miR-520b	ENSG00000171791	BCL2	protein_coding	chr18	60790675
MIMAT0002843	hsa-miR-520b	ENSG00000171791	BCL2	protein_coding	chr18	60792740
MIMAT0002843	hsa-miR-520b	ENSG00000171791	BCL2	protein_coding	chr18	60793020
MIMAT0002844	hsa-miR-518b	ENSG00000171791	BCL2	protein_coding	chr18	60795484
MIMAT0002846	hsa-miR-520c-3p	ENSG00000171791	BCL2	protein_coding	chr18	60790675
MIMAT0002846	hsa-miR-520c-3p	ENSG00000171791	BCL2	protein_coding	chr18	60792740
MIMAT0002846	hsa-miR-520c-3p	ENSG00000171791	BCL2	protein_coding	chr18	60793020
MIMAT0002848	hsa-miR-518c-3p	ENSG00000171791	BCL2	protein_coding	chr18	60795484
MIMAT0002849	hsa-miR-524-5p	ENSG00000171791	BCL2	protein_coding	chr18	60790858
MIMAT0002853	hsa-miR-519d-3p	ENSG00000171791	BCL2	protein_coding	chr18	60790674
MIMAT0002853	hsa-miR-519d-3p	ENSG00000171791	BCL2	protein_coding	chr18	60792739
MIMAT0002855	hsa-miR-520d-5p	ENSG00000171791	BCL2	protein_coding	chr18	60790858
MIMAT0002856	hsa-miR-520d-3p	ENSG00000171791	BCL2	protein_coding	chr18	60790675
MIMAT0002856	hsa-miR-520d-3p	ENSG00000171791	BCL2	protein_coding	chr18	60792740
MIMAT0002856	hsa-miR-520d-3p	ENSG00000171791	BCL2	protein_coding	chr18	60793020
MIMAT0002858	hsa-miR-520g-3p	ENSG00000171791	BCL2	protein_coding	chr18	60790673
MIMAT0002858	hsa-miR-520g-3p	ENSG00000171791	BCL2	protein_coding	chr18	60794107
MIMAT0002858	hsa-miR-520g-3p	ENSG00000171791	BCL2	protein_coding	chr18	60794459
MIMAT0002859	hsa-miR-516b-5p	ENSG00000171791	BCL2	protein_coding	chr18	60791059
MIMAT0002859	hsa-miR-516b-5p	ENSG00000171791	BCL2	protein_coding	chr18	60791651
MIMAT0002863	hsa-miR-518a-3p	ENSG00000171791	BCL2	protein_coding	chr18	60795484
MIMAT0002864	hsa-miR-518d-3p	ENSG00000171791	BCL2	protein_coding	chr18	60795484
MIMAT0002867	hsa-miR-520h	ENSG00000171791	BCL2	protein_coding	chr18	60790673
MIMAT0002867	hsa-miR-520h	ENSG00000171791	BCL2	protein_coding	chr18	60794107
MIMAT0002867	hsa-miR-520h	ENSG00000171791	BCL2	protein_coding	chr18	60794459
MIMAT0002868	hsa-miR-522-3p	ENSG00000171791	BCL2	protein_coding	chr18	60792436
MIMAT0002868	hsa-miR-522-3p	ENSG00000171791	BCL2	protein_coding	chr18	60794123
MIMAT0002868	hsa-miR-522-3p	ENSG00000171791	BCL2	protein_coding	chr18	60794536
MIMAT0002869	hsa-miR-519a-3p	ENSG00000171791	BCL2	protein_coding	chr18	60790675
MIMAT0002869	hsa-miR-519a-3p	ENSG00000171791	BCL2	protein_coding	chr18	60792740
MIMAT0002869	hsa-miR-519a-3p	ENSG00000171791	BCL2	protein_coding	chr18	60793020
MIMAT0002870	hsa-miR-499a-5p	ENSG00000171791	BCL2	protein_coding	chr18	60791401
MIMAT0002874	hsa-miR-503-5p	ENSG00000171791	BCL2	protein_coding	chr18	60793253
MIMAT0002874	hsa-miR-503-5p	ENSG00000171791	BCL2	protein_coding	chr18	60793323
MIMAT0002874	hsa-miR-503-5p	ENSG00000171791	BCL2	protein_coding	chr18	60794055
MIMAT0002874	hsa-miR-503-5p	ENSG00000171791	BCL2	protein_coding	chr18	60795399
MIMAT0002877	hsa-miR-513a-5p	ENSG00000171791	BCL2	protein_coding	chr18	60794487
MIMAT0002877	hsa-miR-513a-5p	ENSG00000171791	BCL2	protein_coding	chr18	60795343
MIMAT0002880	hsa-miR-508-3p	ENSG00000171791	BCL2	protein_coding	chr18	60791348
MIMAT0002881	hsa-miR-509-3p	ENSG00000171791	BCL2	protein_coding	chr18	60793333
MIMAT0002882	hsa-miR-510-5p	ENSG00000171791	BCL2	protein_coding	chr18	60791108
MIMAT0002882	hsa-miR-510-5p	ENSG00000171791	BCL2	protein_coding	chr18	60793899
MIMAT0002882	hsa-miR-510-5p	ENSG00000171791	BCL2	protein_coding	chr18	60795005
MIMAT0002883	hsa-miR-514a-3p	ENSG00000171791	BCL2	protein_coding	chr18	60793358
MIMAT0003150	hsa-miR-455-5p	ENSG00000171791	BCL2	protein_coding	chr18	60794522
MIMAT0003161	hsa-miR-493-3p	ENSG00000171791	BCL2	protein_coding	chr18	60790817
MIMAT0003161	hsa-miR-493-3p	ENSG00000171791	BCL2	protein_coding	chr18	60790949
MIMAT0003161	hsa-miR-493-3p	ENSG00000171791	BCL2	protein_coding	chr18	60791519
MIMAT0003215	hsa-miR-552-3p	ENSG00000171791	BCL2	protein_coding	chr18	60794489
MIMAT0003218	hsa-miR-92b-3p	ENSG00000171791	BCL2	protein_coding	chr18	60791607
MIMAT0003220	hsa-miR-556-5p	ENSG00000171791	BCL2	protein_coding	chr18	60791051
MIMAT0003241	hsa-miR-576-5p	ENSG00000171791	BCL2	protein_coding	chr18	60792967
MIMAT0003241	hsa-miR-576-5p	ENSG00000171791	BCL2	protein_coding	chr18	60793247
MIMAT0003241	hsa-miR-576-5p	ENSG00000171791	BCL2	protein_coding	chr18	60794180
MIMAT0003242	hsa-miR-577	ENSG00000171791	BCL2	protein_coding	chr18	60793945
MIMAT0003242	hsa-miR-577	ENSG00000171791	BCL2	protein_coding	chr18	60794119
MIMAT0003242	hsa-miR-577	ENSG00000171791	BCL2	protein_coding	chr18	60794295
MIMAT0003242	hsa-miR-577	ENSG00000171791	BCL2	protein_coding	chr18	60794368
MIMAT0003244	hsa-miR-579-3p	ENSG00000171791	BCL2	protein_coding	chr18	60791692
MIMAT0003244	hsa-miR-579-3p	ENSG00000171791	BCL2	protein_coding	chr18	60794464
MIMAT0003247	hsa-miR-582-5p	ENSG00000171791	BCL2	protein_coding	chr18	60791123

MIMAT0003247	hsa-miR-582-5p	ENSG00000171791	BCL2	protein_coding	chr18	60791289
MIMAT0003247	hsa-miR-582-5p	ENSG00000171791	BCL2	protein_coding	chr18	60792163
MIMAT0003247	hsa-miR-582-5p	ENSG00000171791	BCL2	protein_coding	chr18	60793039
MIMAT0003247	hsa-miR-582-5p	ENSG00000171791	BCL2	protein_coding	chr18	60794358
MIMAT0003247	hsa-miR-582-5p	ENSG00000171791	BCL2	protein_coding	chr18	60794627
MIMAT0003255	hsa-miR-588	ENSG00000171791	BCL2	protein_coding	chr18	60790696
MIMAT0003255	hsa-miR-588	ENSG00000171791	BCL2	protein_coding	chr18	60793862
MIMAT0003255	hsa-miR-588	ENSG00000171791	BCL2	protein_coding	chr18	60794546
MIMAT0003258	hsa-miR-590-5p	ENSG00000171791	BCL2	protein_coding	chr18	60795133
MIMAT0003289	hsa-miR-620	ENSG00000171791	BCL2	protein_coding	chr18	60790704
MIMAT0003289	hsa-miR-620	ENSG00000171791	BCL2	protein_coding	chr18	60792478
MIMAT0003294	hsa-miR-625-5p	ENSG00000171791	BCL2	protein_coding	chr18	60987019
MIMAT0003296	hsa-miR-627-5p	ENSG00000171791	BCL2	protein_coding	chr18	60795264
MIMAT0003311	hsa-miR-641	ENSG00000171791	BCL2	protein_coding	chr18	60791373
MIMAT0003311	hsa-miR-641	ENSG00000171791	BCL2	protein_coding	chr18	60792002
MIMAT0003311	hsa-miR-641	ENSG00000171791	BCL2	protein_coding	chr18	60793198
MIMAT0003311	hsa-miR-641	ENSG00000171791	BCL2	protein_coding	chr18	60795567
MIMAT0003312	hsa-miR-642a-5p	ENSG00000171791	BCL2	protein_coding	chr18	60794621
MIMAT0003312	hsa-miR-642a-5p	ENSG00000171791	BCL2	protein_coding	chr18	60795329
MIMAT0003320	hsa-miR-650	ENSG00000171791	BCL2	protein_coding	chr18	60795299
MIMAT0003321	hsa-miR-651-5p	ENSG00000171791	BCL2	protein_coding	chr18	60792213
MIMAT0003321	hsa-miR-651-5p	ENSG00000171791	BCL2	protein_coding	chr18	60794439
MIMAT0003327	hsa-miR-449b-5p	ENSG00000171791	BCL2	protein_coding	chr18	60791270
MIMAT0003327	hsa-miR-449b-5p	ENSG00000171791	BCL2	protein_coding	chr18	60795650
MIMAT0003328	hsa-miR-653-5p	ENSG00000171791	BCL2	protein_coding	chr18	60791234
MIMAT0003330	hsa-miR-654-5p	ENSG00000171791	BCL2	protein_coding	chr18	60794492
MIMAT0003331	hsa-miR-655-3p	ENSG00000171791	BCL2	protein_coding	chr18	60794113
MIMAT0003332	hsa-miR-656-3p	ENSG00000171791	BCL2	protein_coding	chr18	60790613
MIMAT0003332	hsa-miR-656-3p	ENSG00000171791	BCL2	protein_coding	chr18	60793215
MIMAT0003332	hsa-miR-656-3p	ENSG00000171791	BCL2	protein_coding	chr18	60795797
MIMAT0003338	hsa-miR-660-5p	ENSG00000171791	BCL2	protein_coding	chr18	60791573
MIMAT0003389	hsa-miR-542-3p	ENSG00000171791	BCL2	protein_coding	chr18	60793868
MIMAT0003389	hsa-miR-542-3p	ENSG00000171791	BCL2	protein_coding	chr18	60794362
MIMAT0003393	hsa-miR-425-5p	ENSG00000171791	BCL2	protein_coding	chr18	60794414
MIMAT0003879	hsa-miR-758-3p	ENSG00000171791	BCL2	protein_coding	chr18	60794446
MIMAT0003881	hsa-miR-668-3p	ENSG00000171791	BCL2	protein_coding	chr18	60791008
MIMAT0003881	hsa-miR-668-3p	ENSG00000171791	BCL2	protein_coding	chr18	60794227
MIMAT0003885	hsa-miR-454-3p	ENSG00000171791	BCL2	protein_coding	chr18	60792741
MIMAT0003885	hsa-miR-454-3p	ENSG00000171791	BCL2	protein_coding	chr18	60793021
MIMAT0003948	hsa-miR-770-5p	ENSG00000171791	BCL2	protein_coding	chr18	60790907
MIMAT0003948	hsa-miR-770-5p	ENSG00000171791	BCL2	protein_coding	chr18	60795017
MIMAT0004185	hsa-miR-802	ENSG00000171791	BCL2	protein_coding	chr18	60790909
MIMAT0004502	hsa-miR-28-3p	ENSG00000171791	BCL2	protein_coding	chr18	60790619
MIMAT0004593	hsa-miR-130a-5p	ENSG00000171791	BCL2	protein_coding	chr18	60791441
MIMAT0004593	hsa-miR-130a-5p	ENSG00000171791	BCL2	protein_coding	chr18	60792191
MIMAT0004593	hsa-miR-130a-5p	ENSG00000171791	BCL2	protein_coding	chr18	60792428
MIMAT0004594	hsa-miR-132-5p	ENSG00000171791	BCL2	protein_coding	chr18	60794543
MIMAT0004597	hsa-miR-140-3p	ENSG00000171791	BCL2	protein_coding	chr18	60791670
MIMAT0004597	hsa-miR-140-3p	ENSG00000171791	BCL2	protein_coding	chr18	60792007
MIMAT0004597	hsa-miR-140-3p	ENSG00000171791	BCL2	protein_coding	chr18	60794074
MIMAT0004675	hsa-miR-219a-2-3	ENSG00000171791	BCL2	protein_coding	chr18	60793737
MIMAT0004679	hsa-miR-296-3p	ENSG00000171791	BCL2	protein_coding	chr18	60987024
MIMAT0004682	hsa-miR-361-3p	ENSG00000171791	BCL2	protein_coding	chr18	60794301
MIMAT0004682	hsa-miR-361-3p	ENSG00000171791	BCL2	protein_coding	chr18	60795314
MIMAT0004682	hsa-miR-361-3p	ENSG00000171791	BCL2	protein_coding	chr18	60795349
MIMAT0004687	hsa-miR-371a-5p	ENSG00000171791	BCL2	protein_coding	chr18	60790914
MIMAT0004687	hsa-miR-371a-5p	ENSG00000171791	BCL2	protein_coding	chr18	60793955
MIMAT0004690	hsa-miR-379-3p	ENSG00000171791	BCL2	protein_coding	chr18	60793364
MIMAT0004690	hsa-miR-379-3p	ENSG00000171791	BCL2	protein_coding	chr18	60793705
MIMAT0004690	hsa-miR-379-3p	ENSG00000171791	BCL2	protein_coding	chr18	60794382
MIMAT0004690	hsa-miR-379-3p	ENSG00000171791	BCL2	protein_coding	chr18	60795622
MIMAT0004692	hsa-miR-340-5p	ENSG00000171791	BCL2	protein_coding	chr18	60791457
MIMAT0004692	hsa-miR-340-5p	ENSG00000171791	BCL2	protein_coding	chr18	60792198
MIMAT0004692	hsa-miR-340-5p	ENSG00000171791	BCL2	protein_coding	chr18	60792433
MIMAT0004692	hsa-miR-340-5p	ENSG00000171791	BCL2	protein_coding	chr18	60795096

MIMAT0004693	hsa-miR-330-5p	ENSG00000171791	BCL2	protein_coding	chr18	60791650
MIMAT0004763	hsa-miR-488-3p	ENSG00000171791	BCL2	protein_coding	chr18	60792278
MIMAT0004774	hsa-miR-501-3p	ENSG00000171791	BCL2	protein_coding	chr18	60793828
MIMAT0004775	hsa-miR-502-3p	ENSG00000171791	BCL2	protein_coding	chr18	60793828
MIMAT0004780	hsa-miR-532-3p	ENSG00000171791	BCL2	protein_coding	chr18	60794659
MIMAT0004780	hsa-miR-532-3p	ENSG00000171791	BCL2	protein_coding	chr18	60795242
MIMAT0004784	hsa-miR-455-3p	ENSG00000171791	BCL2	protein_coding	chr18	60791272
MIMAT0004785	hsa-miR-545-5p	ENSG00000171791	BCL2	protein_coding	chr18	60790907
MIMAT0004785	hsa-miR-545-5p	ENSG00000171791	BCL2	protein_coding	chr18	60795652
MIMAT0004797	hsa-miR-582-3p	ENSG00000171791	BCL2	protein_coding	chr18	60790701
MIMAT0004797	hsa-miR-582-3p	ENSG00000171791	BCL2	protein_coding	chr18	60794185
MIMAT0004805	hsa-miR-616-3p	ENSG00000171791	BCL2	protein_coding	chr18	60791526
MIMAT0004805	hsa-miR-616-3p	ENSG00000171791	BCL2	protein_coding	chr18	60794463
MIMAT0004807	hsa-miR-624-3p	ENSG00000171791	BCL2	protein_coding	chr18	60790628
MIMAT0004809	hsa-miR-628-5p	ENSG00000171791	BCL2	protein_coding	chr18	60791641
MIMAT0004809	hsa-miR-628-5p	ENSG00000171791	BCL2	protein_coding	chr18	60794217
MIMAT0004813	hsa-miR-411-3p	ENSG00000171791	BCL2	protein_coding	chr18	60793364
MIMAT0004813	hsa-miR-411-3p	ENSG00000171791	BCL2	protein_coding	chr18	60793705
MIMAT0004813	hsa-miR-411-3p	ENSG00000171791	BCL2	protein_coding	chr18	60794382
MIMAT0004813	hsa-miR-411-3p	ENSG00000171791	BCL2	protein_coding	chr18	60795622
MIMAT0004814	hsa-miR-654-3p	ENSG00000171791	BCL2	protein_coding	chr18	60791646
MIMAT0004814	hsa-miR-654-3p	ENSG00000171791	BCL2	protein_coding	chr18	60794638
MIMAT0004903	hsa-miR-300	ENSG00000171791	BCL2	protein_coding	chr18	60790857
MIMAT0004903	hsa-miR-300	ENSG00000171791	BCL2	protein_coding	chr18	60794009
MIMAT0004909	hsa-miR-450b-5p	ENSG00000171791	BCL2	protein_coding	chr18	60791320
MIMAT0004909	hsa-miR-450b-5p	ENSG00000171791	BCL2	protein_coding	chr18	60792244
MIMAT0004909	hsa-miR-450b-5p	ENSG00000171791	BCL2	protein_coding	chr18	60795825
MIMAT0004911	hsa-miR-874-3p	ENSG00000171791	BCL2	protein_coding	chr18	60792302
MIMAT0004916	hsa-miR-888-5p	ENSG00000171791	BCL2	protein_coding	chr18	60790913
MIMAT0004916	hsa-miR-888-5p	ENSG00000171791	BCL2	protein_coding	chr18	60792259
MIMAT0004919	hsa-miR-541-5p	ENSG00000171791	BCL2	protein_coding	chr18	60987054
MIMAT0004920	hsa-miR-541-3p	ENSG00000171791	BCL2	protein_coding	chr18	60794492
MIMAT0004921	hsa-miR-889-3p	ENSG00000171791	BCL2	protein_coding	chr18	60793145
MIMAT0004921	hsa-miR-889-3p	ENSG00000171791	BCL2	protein_coding	chr18	60793383
MIMAT0004921	hsa-miR-889-3p	ENSG00000171791	BCL2	protein_coding	chr18	60795338
MIMAT0004924	hsa-miR-876-5p	ENSG00000171791	BCL2	protein_coding	chr18	60791102
MIMAT0004924	hsa-miR-876-5p	ENSG00000171791	BCL2	protein_coding	chr18	60794442
MIMAT0004924	hsa-miR-876-5p	ENSG00000171791	BCL2	protein_coding	chr18	60795558
MIMAT0004947	hsa-miR-885-5p	ENSG00000171791	BCL2	protein_coding	chr18	60790680
MIMAT0004947	hsa-miR-885-5p	ENSG00000171791	BCL2	protein_coding	chr18	60791575
MIMAT0004947	hsa-miR-885-5p	ENSG00000171791	BCL2	protein_coding	chr18	60793299
MIMAT0004952	hsa-miR-665	ENSG00000171791	BCL2	protein_coding	chr18	60794284
MIMAT0004953	hsa-miR-873-5p	ENSG00000171791	BCL2	protein_coding	chr18	60795320
MIMAT0004954	hsa-miR-543	ENSG00000171791	BCL2	protein_coding	chr18	60791488
MIMAT0004954	hsa-miR-543	ENSG00000171791	BCL2	protein_coding	chr18	60794086
MIMAT0004955	hsa-miR-374b-5p	ENSG00000171791	BCL2	protein_coding	chr18	60790954
MIMAT0004955	hsa-miR-374b-5p	ENSG00000171791	BCL2	protein_coding	chr18	60794131
MIMAT0004958	hsa-miR-301b-3p	ENSG00000171791	BCL2	protein_coding	chr18	60792741
MIMAT0004958	hsa-miR-301b-3p	ENSG00000171791	BCL2	protein_coding	chr18	60793021
MIMAT0004959	hsa-miR-216b-5p	ENSG00000171791	BCL2	protein_coding	chr18	60793952
MIMAT0004959	hsa-miR-216b-5p	ENSG00000171791	BCL2	protein_coding	chr18	60795197
MIMAT0004960	hsa-miR-208b-3p	ENSG00000171791	BCL2	protein_coding	chr18	60791401
MIMAT0004985	hsa-miR-942-5p	ENSG00000171791	BCL2	protein_coding	chr18	60793917
MIMAT0004985	hsa-miR-942-5p	ENSG00000171791	BCL2	protein_coding	chr18	60795383
MIMAT0005788	hsa-miR-513b-5p	ENSG00000171791	BCL2	protein_coding	chr18	60790608
MIMAT0005788	hsa-miR-513b-5p	ENSG00000171791	BCL2	protein_coding	chr18	60793798
MIMAT0005788	hsa-miR-513b-5p	ENSG00000171791	BCL2	protein_coding	chr18	60793981
MIMAT0005789	hsa-miR-513c-5p	ENSG00000171791	BCL2	protein_coding	chr18	60793954
MIMAT0005792	hsa-miR-320b	ENSG00000171791	BCL2	protein_coding	chr18	60790847
MIMAT0005792	hsa-miR-320b	ENSG00000171791	BCL2	protein_coding	chr18	60790978
MIMAT0005792	hsa-miR-320b	ENSG00000171791	BCL2	protein_coding	chr18	60792280
MIMAT0005792	hsa-miR-320b	ENSG00000171791	BCL2	protein_coding	chr18	60793850
MIMAT0005793	hsa-miR-320c	ENSG00000171791	BCL2	protein_coding	chr18	60790847
MIMAT0005793	hsa-miR-320c	ENSG00000171791	BCL2	protein_coding	chr18	60790978
MIMAT0005793	hsa-miR-320c	ENSG00000171791	BCL2	protein_coding	chr18	60792280

MIMAT0005793	hsa-miR-320c	ENSG00000171791	BCL2	protein_coding	chr18	60793850
MIMAT0005795	hsa-miR-1323	ENSG00000171791	BCL2	protein_coding	chr18	60790916
MIMAT0005795	hsa-miR-1323	ENSG00000171791	BCL2	protein_coding	chr18	60790963
MIMAT0005795	hsa-miR-1323	ENSG00000171791	BCL2	protein_coding	chr18	60793202
MIMAT0005796	hsa-miR-1271-5p	ENSG00000171791	BCL2	protein_coding	chr18	60793313
MIMAT0005796	hsa-miR-1271-5p	ENSG00000171791	BCL2	protein_coding	chr18	60795542
MIMAT0005796	hsa-miR-1271-5p	ENSG00000171791	BCL2	protein_coding	chr18	60795648
MIMAT0005797	hsa-miR-1301-3p	ENSG00000171791	BCL2	protein_coding	chr18	60793023
MIMAT0005797	hsa-miR-1301-3p	ENSG00000171791	BCL2	protein_coding	chr18	60795762
MIMAT0005798	hsa-miR-1185-5p	ENSG00000171791	BCL2	protein_coding	chr18	60793262
MIMAT0005800	hsa-miR-1298-5p	ENSG00000171791	BCL2	protein_coding	chr18	60793407
MIMAT0005800	hsa-miR-1298-5p	ENSG00000171791	BCL2	protein_coding	chr18	60793423
MIMAT0005824	hsa-miR-1179	ENSG00000171791	BCL2	protein_coding	chr18	60792405
MIMAT0005824	hsa-miR-1179	ENSG00000171791	BCL2	protein_coding	chr18	60794079
MIMAT0005877	hsa-miR-1286	ENSG00000171791	BCL2	protein_coding	chr18	60793316
MIMAT0005885	hsa-miR-1295a	ENSG00000171791	BCL2	protein_coding	chr18	60793995
MIMAT0005919	hsa-miR-548o-3p	ENSG00000171791	BCL2	protein_coding	chr18	60790916
MIMAT0005919	hsa-miR-548o-3p	ENSG00000171791	BCL2	protein_coding	chr18	60790963
MIMAT0005919	hsa-miR-548o-3p	ENSG00000171791	BCL2	protein_coding	chr18	60793202
MIMAT0005923	hsa-miR-1269a	ENSG00000171791	BCL2	protein_coding	chr18	60795306
MIMAT0005924	hsa-miR-1270	ENSG00000171791	BCL2	protein_coding	chr18	60790704
MIMAT0005924	hsa-miR-1270	ENSG00000171791	BCL2	protein_coding	chr18	60792478
MIMAT0005930	hsa-miR-1276	ENSG00000171791	BCL2	protein_coding	chr18	60790806
MIMAT0005930	hsa-miR-1276	ENSG00000171791	BCL2	protein_coding	chr18	60795276
MIMAT0005931	hsa-miR-302e	ENSG00000171791	BCL2	protein_coding	chr18	60790675
MIMAT0005931	hsa-miR-302e	ENSG00000171791	BCL2	protein_coding	chr18	60792740
MIMAT0005931	hsa-miR-302e	ENSG00000171791	BCL2	protein_coding	chr18	60793020
MIMAT0005951	hsa-miR-1307-3p	ENSG00000171791	BCL2	protein_coding	chr18	60795118
MIMAT0005952	hsa-miR-1321	ENSG00000171791	BCL2	protein_coding	chr18	60795297
MIMAT0006764	hsa-miR-320d	ENSG00000171791	BCL2	protein_coding	chr18	60790847
MIMAT0006764	hsa-miR-320d	ENSG00000171791	BCL2	protein_coding	chr18	60790978
MIMAT0006764	hsa-miR-320d	ENSG00000171791	BCL2	protein_coding	chr18	60792280
MIMAT0006764	hsa-miR-320d	ENSG00000171791	BCL2	protein_coding	chr18	60793850
MIMAT0007885	hsa-miR-1911-5p	ENSG00000171791	BCL2	protein_coding	chr18	60794097
MIMAT0007888	hsa-miR-1913	ENSG00000171791	BCL2	protein_coding	chr18	60792569
MIMAT0009198	hsa-miR-224-3p	ENSG00000171791	BCL2	protein_coding	chr18	60792435
MIMAT0009198	hsa-miR-224-3p	ENSG00000171791	BCL2	protein_coding	chr18	60794122
MIMAT0009198	hsa-miR-224-3p	ENSG00000171791	BCL2	protein_coding	chr18	60794535
MIMAT0010357	hsa-miR-670-5p	ENSG00000171791	BCL2	protein_coding	chr18	60793432
MIMAT0014980	hsa-miR-3118	ENSG00000171791	BCL2	protein_coding	chr18	60790649
MIMAT0014980	hsa-miR-3118	ENSG00000171791	BCL2	protein_coding	chr18	60792531
MIMAT0014983	hsa-miR-3121-3p	ENSG00000171791	BCL2	protein_coding	chr18	60792231
MIMAT0014983	hsa-miR-3121-3p	ENSG00000171791	BCL2	protein_coding	chr18	60793984
MIMAT0014983	hsa-miR-3121-3p	ENSG00000171791	BCL2	protein_coding	chr18	60795655
MIMAT0014990	hsa-miR-3127-5p	ENSG00000171791	BCL2	protein_coding	chr18	60793901
MIMAT0014990	hsa-miR-3127-5p	ENSG00000171791	BCL2	protein_coding	chr18	60795068
MIMAT0015008	hsa-miR-3140-3p	ENSG00000171791	BCL2	protein_coding	chr18	60792945
MIMAT0015008	hsa-miR-3140-3p	ENSG00000171791	BCL2	protein_coding	chr18	60793927
MIMAT0015008	hsa-miR-3140-3p	ENSG00000171791	BCL2	protein_coding	chr18	60795111
MIMAT0015016	hsa-miR-3145-3p	ENSG00000171791	BCL2	protein_coding	chr18	60793337
MIMAT0015016	hsa-miR-3145-3p	ENSG00000171791	BCL2	protein_coding	chr18	60795088
MIMAT0015016	hsa-miR-3145-3p	ENSG00000171791	BCL2	protein_coding	chr18	60795636
MIMAT0015037	hsa-miR-3163	ENSG00000171791	BCL2	protein_coding	chr18	60791457
MIMAT0015037	hsa-miR-3163	ENSG00000171791	BCL2	protein_coding	chr18	60792415
MIMAT0015037	hsa-miR-3163	ENSG00000171791	BCL2	protein_coding	chr18	60793376
MIMAT0015037	hsa-miR-3163	ENSG00000171791	BCL2	protein_coding	chr18	60794345
MIMAT0015038	hsa-miR-3164	ENSG00000171791	BCL2	protein_coding	chr18	60790650
MIMAT0015038	hsa-miR-3164	ENSG00000171791	BCL2	protein_coding	chr18	60792532
MIMAT0015042	hsa-miR-3167	ENSG00000171791	BCL2	protein_coding	chr18	60791101
MIMAT0015042	hsa-miR-3167	ENSG00000171791	BCL2	protein_coding	chr18	60794441
MIMAT0015042	hsa-miR-3167	ENSG00000171791	BCL2	protein_coding	chr18	60795557
MIMAT0015069	hsa-miR-3187-3p	ENSG00000171791	BCL2	protein_coding	chr18	60790696
MIMAT0015069	hsa-miR-3187-3p	ENSG00000171791	BCL2	protein_coding	chr18	60793862
MIMAT0015069	hsa-miR-3187-3p	ENSG00000171791	BCL2	protein_coding	chr18	60794546
MIMAT0015078	hsa-miR-3194-5p	ENSG00000171791	BCL2	protein_coding	chr18	60795411

MIMAT0015085	hsa-miR-3200-3p	ENSG00000171791	BCL2	protein_coding	chr18	60790930
MIMAT0015085	hsa-miR-3200-3p	ENSG00000171791	BCL2	protein_coding	chr18	60791026
MIMAT0015085	hsa-miR-3200-3p	ENSG00000171791	BCL2	protein_coding	chr18	60791227
MIMAT0016870	hsa-miR-4319	ENSG00000171791	BCL2	protein_coding	chr18	60792304
MIMAT0016870	hsa-miR-4319	ENSG00000171791	BCL2	protein_coding	chr18	60793433
MIMAT0016894	hsa-miR-4262	ENSG00000171791	BCL2	protein_coding	chr18	60792955
MIMAT0016894	hsa-miR-4262	ENSG00000171791	BCL2	protein_coding	chr18	60794100
MIMAT0016895	hsa-miR-2355-5p	ENSG00000171791	BCL2	protein_coding	chr18	60790984
MIMAT0016895	hsa-miR-2355-5p	ENSG00000171791	BCL2	protein_coding	chr18	60794255
MIMAT0016895	hsa-miR-2355-5p	ENSG00000171791	BCL2	protein_coding	chr18	60795387
MIMAT0016895	hsa-miR-2355-5p	ENSG00000171791	BCL2	protein_coding	chr18	60795418
MIMAT0017950	hsa-miR-2355-3p	ENSG00000171791	BCL2	protein_coding	chr18	60791350
MIMAT0017950	hsa-miR-2355-3p	ENSG00000171791	BCL2	protein_coding	chr18	60791366
MIMAT0017989	hsa-miR-3612	ENSG00000171791	BCL2	protein_coding	chr18	60795299
MIMAT0018000	hsa-miR-23c	ENSG00000171791	BCL2	protein_coding	chr18	60791441
MIMAT0018000	hsa-miR-23c	ENSG00000171791	BCL2	protein_coding	chr18	60792191
MIMAT0018000	hsa-miR-23c	ENSG00000171791	BCL2	protein_coding	chr18	60792428
MIMAT0018003	hsa-miR-3622a-5	ENSG00000171791	BCL2	protein_coding	chr18	60794904
MIMAT0018003	hsa-miR-3622a-5	ENSG00000171791	BCL2	protein_coding	chr18	60795503
MIMAT0018119	hsa-miR-3690	ENSG00000171791	BCL2	protein_coding	chr18	60792270
MIMAT0018119	hsa-miR-3690	ENSG00000171791	BCL2	protein_coding	chr18	60794316
MIMAT0018192	hsa-miR-3918	ENSG00000171791	BCL2	protein_coding	chr18	60793902
MIMAT0018192	hsa-miR-3918	ENSG00000171791	BCL2	protein_coding	chr18	60794605
MIMAT0018192	hsa-miR-3918	ENSG00000171791	BCL2	protein_coding	chr18	60795069
MIMAT0018192	hsa-miR-3918	ENSG00000171791	BCL2	protein_coding	chr18	60795353
MIMAT0018443	hsa-miR-374c-5p	ENSG00000171791	BCL2	protein_coding	chr18	60794112
MIMAT0018937	hsa-miR-378g	ENSG00000171791	BCL2	protein_coding	chr18	60792376
MIMAT0018937	hsa-miR-378g	ENSG00000171791	BCL2	protein_coding	chr18	60793060
MIMAT0018943	hsa-miR-4428	ENSG00000171791	BCL2	protein_coding	chr18	60987008
MIMAT0019064	hsa-miR-4525	ENSG00000171791	BCL2	protein_coding	chr18	60987010
MIMAT0019214	hsa-miR-3173-5p	ENSG00000171791	BCL2	protein_coding	chr18	60792303
MIMAT0019694	hsa-miR-4637	ENSG00000171791	BCL2	protein_coding	chr18	60792113
MIMAT0019699	hsa-miR-4640-5p	ENSG00000171791	BCL2	protein_coding	chr18	60792378
MIMAT0019699	hsa-miR-4640-5p	ENSG00000171791	BCL2	protein_coding	chr18	60793049
MIMAT0019699	hsa-miR-4640-5p	ENSG00000171791	BCL2	protein_coding	chr18	60794495
MIMAT0019761	hsa-miR-4677-3p	ENSG00000171791	BCL2	protein_coding	chr18	60792530
MIMAT0019761	hsa-miR-4677-3p	ENSG00000171791	BCL2	protein_coding	chr18	60793171
MIMAT0019798	hsa-miR-4701-5p	ENSG00000171791	BCL2	protein_coding	chr18	60790696
MIMAT0019798	hsa-miR-4701-5p	ENSG00000171791	BCL2	protein_coding	chr18	60793862
MIMAT0019798	hsa-miR-4701-5p	ENSG00000171791	BCL2	protein_coding	chr18	60794546
MIMAT0019845	hsa-miR-4726-5p	ENSG00000171791	BCL2	protein_coding	chr18	60792378
MIMAT0019845	hsa-miR-4726-5p	ENSG00000171791	BCL2	protein_coding	chr18	60793049
MIMAT0019845	hsa-miR-4726-5p	ENSG00000171791	BCL2	protein_coding	chr18	60794495
MIMAT0019853	hsa-miR-4731-5p	ENSG00000171791	BCL2	protein_coding	chr18	60793059
MIMAT0019868	hsa-miR-4739	ENSG00000171791	BCL2	protein_coding	chr18	60795297
MIMAT0019899	hsa-miR-4756-5p	ENSG00000171791	BCL2	protein_coding	chr18	60795297
MIMAT0019909	hsa-miR-4761-3p	ENSG00000171791	BCL2	protein_coding	chr18	60795162
MIMAT0019909	hsa-miR-4761-3p	ENSG00000171791	BCL2	protein_coding	chr18	60795354
MIMAT0019924	hsa-miR-4770	ENSG00000171791	BCL2	protein_coding	chr18	60791332
MIMAT0019924	hsa-miR-4770	ENSG00000171791	BCL2	protein_coding	chr18	60794034
MIMAT0020541	hsa-miR-5047	ENSG00000171791	BCL2	protein_coding	chr18	60793023
MIMAT0020541	hsa-miR-5047	ENSG00000171791	BCL2	protein_coding	chr18	60795762
MIMAT0021043	hsa-miR-5010-5p	ENSG00000171791	BCL2	protein_coding	chr18	60987010
MIMAT0022270	hsa-miR-5579-3p	ENSG00000171791	BCL2	protein_coding	chr18	60795132
MIMAT0022287	hsa-miR-5586-5p	ENSG00000171791	BCL2	protein_coding	chr18	60791325
MIMAT0022287	hsa-miR-5586-5p	ENSG00000171791	BCL2	protein_coding	chr18	60791586
MIMAT0022287	hsa-miR-5586-5p	ENSG00000171791	BCL2	protein_coding	chr18	60793873
MIMAT0022287	hsa-miR-5586-5p	ENSG00000171791	BCL2	protein_coding	chr18	60795514
MIMAT0022479	hsa-miR-5688	ENSG00000171791	BCL2	protein_coding	chr18	60790670
MIMAT0022479	hsa-miR-5688	ENSG00000171791	BCL2	protein_coding	chr18	60790843
MIMAT0022479	hsa-miR-5688	ENSG00000171791	BCL2	protein_coding	chr18	60790894
MIMAT0022479	hsa-miR-5688	ENSG00000171791	BCL2	protein_coding	chr18	60790919
MIMAT0022479	hsa-miR-5688	ENSG00000171791	BCL2	protein_coding	chr18	60790961
MIMAT0022479	hsa-miR-5688	ENSG00000171791	BCL2	protein_coding	chr18	60792420
MIMAT0022479	hsa-miR-5688	ENSG00000171791	BCL2	protein_coding	chr18	60793193

MIMAT0022479	hsa-miR-5688	ENSG00000171791	BCL2	protein_coding	chr18	60793847
MIMAT0022479	hsa-miR-5688	ENSG00000171791	BCL2	protein_coding	chr18	60794020
MIMAT0022697	hsa-miR-382-3p	ENSG00000171791	BCL2	protein_coding	chr18	60793406
MIMAT0022698	hsa-miR-345-3p	ENSG00000171791	BCL2	protein_coding	chr18	60792304
MIMAT0022698	hsa-miR-345-3p	ENSG00000171791	BCL2	protein_coding	chr18	60793433
MIMAT0022717	hsa-miR-873-3p	ENSG00000171791	BCL2	protein_coding	chr18	60790706
MIMAT0022717	hsa-miR-873-3p	ENSG00000171791	BCL2	protein_coding	chr18	60793896
MIMAT0022724	hsa-miR-1277-5p	ENSG00000171791	BCL2	protein_coding	chr18	60793145
MIMAT0022724	hsa-miR-1277-5p	ENSG00000171791	BCL2	protein_coding	chr18	60795048
MIMAT0022736	hsa-miR-642b-5p	ENSG00000171791	BCL2	protein_coding	chr18	60791190
MIMAT0022736	hsa-miR-642b-5p	ENSG00000171791	BCL2	protein_coding	chr18	60793430
MIMAT0022736	hsa-miR-642b-5p	ENSG00000171791	BCL2	protein_coding	chr18	60794253
MIMAT0022736	hsa-miR-642b-5p	ENSG00000171791	BCL2	protein_coding	chr18	60794566
MIMAT0022736	hsa-miR-642b-5p	ENSG00000171791	BCL2	protein_coding	chr18	60795226
MIMAT0022834	hsa-miR-365b-3p	ENSG00000171791	BCL2	protein_coding	chr18	60794141
MIMAT0022834	hsa-miR-365b-3p	ENSG00000171791	BCL2	protein_coding	chr18	60794278
MIMAT0023713	hsa-miR-6088	ENSG00000171791	BCL2	protein_coding	chr18	60791332
MIMAT0023713	hsa-miR-6088	ENSG00000171791	BCL2	protein_coding	chr18	60794034
MIMAT0027571	hsa-miR-6835-3p	ENSG00000171791	BCL2	protein_coding	chr18	60793250
MIMAT0027578	hsa-miR-6838-5p	ENSG00000171791	BCL2	protein_coding	chr18	60793322
